# Multiscale Characterizations of Surface Anisotropies

**DOI:** 10.3390/ma13133028

**Published:** 2020-07-07

**Authors:** Tomasz Bartkowiak, Johan Berglund, Christopher A. Brown

**Affiliations:** 1 Institute of Mechanical Technology, Poznan University of Technology, 60-965 Poznań, Poland; 2 Department of Manufacturing, RISE Research Institutes of Sweden, SE-43153 Mölndal, Sweden; johan.berglund@ri.se; 3 Department of Industrial and Materials Science, Chalmers University of Technology, SE-41296 Gothenburg, Sweden; 4 Surface Metrology Lab, Worcester Polytechnic Institute, Worcester, MA 01609, USA; brown@wpi.edu

**Keywords:** surface texture, anisotropy, multiscale

## Abstract

Anisotropy can influence surface function and can be an indication of processing. These influences and indications include friction, wetting, and microwear. This article studies two methods for multiscale quantification and visualization of anisotropy. One uses multiscale curvature tensor analysis and shows anisotropy in horizontal coordinates i.e., topocentric. The other uses multiple bandpass filters (also known as sliding bandpass filters) applied prior to calculating anisotropy parameters, texture aspect ratios (Str) and texture directions (Std), showing anisotropy in horizontal directions only. Topographies were studied on two milled steel surfaces, one convex with an evident large scale, cylindrical form anisotropy, the other nominally flat with smaller scale anisotropies; a µEDMed surface, an example of an isotropic surface; and an additively manufactured surface with pillar-like features. Curvature tensors contain the two principal curvatures, i.e., maximum and minimum curvatures, which are orthogonal, and their directions, at each location. Principal directions are plotted for each calculated location on each surface, at each scale considered. Histograms in horizontal coordinates show altitude and azimuth angles of principal curvatures, elucidating dominant texture directions at each scale. Str and Std do not show vertical components, i.e., altitudes, of anisotropy. Changes of anisotropy with scale categorically failed to be detected by traditional characterization methods used conventionally. These multiscale methods show clearly in several representations that anisotropy changes with scale on actual surface measurements with markedly different anisotropies.

## 1. Introduction

The objective of this paper is to present and study two new multiscale methods for determining anisotropy, i.e., lay or directionality of topographies. One method, based on curvature tensors, is primarily geometric and naturally multiscale. The other method, based on auto correlation and Fourier analyses, usually are applied to large single ranges of scales (ISO25178 [1]) and are implemented as multiscale here by using multiple bandpass filters to cover a multitude of scales. This study compares visual impressions of anisotropy from height maps of four surfaces, selected for their distinctive anisotropic characteristics, with representations of anisotropies by results of these two methods.

This is important because anisotropy at different scales can be an indicator of processing and performance. These indications can be valuable for product and process design and analysis, and for analyzing wear phenomena in engineering, anthropology, and archeology [2]. Currently, we are aware of no multiscale methods for analyzing anisotropies in national or international standards. Many topographically related phenomena are scale specific. Processing can influence different scales differently, and performance can be influenced at different scales differently. Knowing specific scales of interaction for phenomena can improve design and analyses of products and processes and augment the sophistication of many kinds of scientific analyses.

Anisotropy can be an ambiguous term in material science and surface metrology. It is generally related to properties which change with direction [3]. In surface metrology specifically, anisotropy relates to topographic characterization parameters changing with direction of observation, measurement, or calculation. Historically “lay” was used to describe a property of machined surfaces [4]. Lay, apart from its mathematical definition, is intuitively recognized visually. It is perceived as a dominant direction, or directions, in which machining marks seem to the eye to line up with each other. Processes like surface grinding and shaping can produce a surface where a profile parallel to the lay will appear to be nearly smooth. Face turning produces a surface with circular marks, while milling and honing processes produce surfaces with complex patterns. Roughing and finishing operations can create lays in more than one direction. Additively manufactured surfaces contain multidirectional features like hills (with peaks), dales (with pits), ridgelines, courses, and saddle points which directions cannot be described with 2D only [5]. Freeform surfaces present new challenges for topographic characterization, because they should not be required to rely on just one datum [6]. Because curvatures are spatial derivatives of slopes, they do not require a datum. This is an important advantage of curvature for characterization of free forms and internal cavities.

Anisotropies affect functional behaviors of surfaces. Summits which are long and narrow are likely to have different load-bearing properties than symmetrical summits [7] and similarly, leakage between contacting surfaces is likely to be influenced by the direction of the anisotropy [8]. It becomes important, therefore, to find, if possible, some quantitative ways of characterizing anisotropy. There are really two different problems: to find the direction or directions of the anisotropy, and to quantify the degree of anisotropy [9]. Anisotropic features can have different geometrical dimensions or sizes, which means that they can be detectable and/or inspectable at specific scales of observation or calculation, justify application of multiscale methods.

In this paper, “scale” refers to a narrow band of spatial frequencies or wavelengths, and multiscale refers to analyses done systematically over a range of scales [2]. Two multiscale methods are used here. One method uses 3D multiscale curvature tensor analysis. The other uses sliding bandpass filtering [2] prior to the calculation of the texture aspect ratio (Str) and texture direction (Std), measures of anisotropy from ISO 25178 [1]. Sliding bandpass filtering is a multiscale analysis method where bandpass filters are applied to isolate narrow bandwidths in scale at regular, even adjacent or overlapping intervals, to cover a wide variety of scales at which characterization parameters can be computed.

Curvature is a geometric property that naturally varies with scales of observation or calculation, as do length, area, and slope. These can be referred to as multiscale geometric analyses [2]. Curvatures on surfaces can be characterized as second order tensors which vary as functions of scale and position. Curvature tensors can be calculated from areal topographic measurements, i.e., surfaces height maps where heights *z* vary with position *x* and *y*, (*z* = *z*(*x,y*)). This kind of areal, multiscale curvature analysis has been recently developed to better understand surface topographies, or textures in mechanical engineering (ASME B46.1 [4]), how they are created and how they perform, based on how they interact with processing, the environment, or other surfaces. Scales of calculation for curvature tensors here are determined by the sizes of the regions over which the height measurements are selected for calculations of curvature to be made.

Topographies are commonly characterized by simplistic height parameters, such as, average roughness, the mean of the absolute values of heights measured from a mean line, Ra (for profiles), or a mean plane, Sa (for surfaces). Typically, roughness characterization analyses are applied to topographic measurements after removing form and waviness with Gaussian filters [1]. The conventional bandwidths for characterizing roughness are large. Sliding bandpass filtering uses different cut-offs to isolate narrow bandwidths. The central spatial frequencies or wavelengths for these narrow bandwidths can be systematically varied over a range of scales. Such multiple, or sliding, bandpass filters can be used to determine scale dependence of conventional texture characterization parameters for finding strong correlations and confident discriminations [2].

Average roughness, like Sa and Ra, is usually most sensitive to the longest wavelengths remaining after filtering form and waviness. This is because amplitudes tend to increase with wavelengths in these scale ranges for many surfaces. Topographic characterizations are valuable for assisting process or product design. When they help to establish correlations, first, between manufacturing processes and the resulting topographies, and, second, between topographies and performance, such as, adhesion or wetting. Conventional height parameters, with conventional filtering, use large bandwidths for waviness and roughness. Because of this coarse treatment of scale, they often fail to find strong correlations with processing parameters or measures of performance. Relations with specific textures, scale-sensitive processing, and performance phenomena, can be specific to certain fine, or narrow, ranges in scale. However, conventional parameters, which show weak correlations with processing and performance when used with traditional filtering, have been shown to correlate strongly when narrow band-pass filters are applied at appropriate scales [10,11]. Conventional height parameters do not provide any insights into the geometric nature of features, their horizontal spacings, or sequences of heights.

Motif analysis was successful in characterizing functional properties of surfaces, especially in friction and contact problems [12,13,14]. In those studies, parameters were used both to identify and separate anisotropic components by appropriate anisotropic filtering and characterization of surface motifs. Today, motif parameters are less used, although conclusions regarding the relationship between function and specification remain crucial.

Another approach for quantitative, multiscale characterization uses PSD (power spectral density), which is based on Fourier transformations. This approach treats signals as combinations of sinusoidal harmonics with different phases, amplitudes, and frequencies. Michalski used angular diagrams and contour maps of PSD to show that anisotropy on gear teeth flanks discriminates kinds of processing [15]. Jacobs et al. presented three important drawbacks to PSD and proposed strategies to mitigate them, and find aperiodicity, tilt, AFM (atomic force microscope) tip shape, and instrument noise [16]. PSD is commonly used to characterize machined surfaces to determine dominance of feeds, versus tool vibrations, and tool edge wear and lubricants [17], to predict the surface roughness in single point diamond turning [18,19]. On machined and worn surfaces dominant frequencies of PSD indicate anisotropy, and multiscale analysis of morphologies inside wear scars show regularly distributed PSD functions with tendencies to reduce maximum wavelengths with decreasing scale [20].

Multiscale curvature characterization of profiles have been correlated with fatigue life with an R^2^ of 0.96 [21], clearly discriminated progressions of edge rounding by mass finishing [22], and found variations in stitching of profiles in measurements of aspheric lenses [23].

Multiscale curvature tensor analyses described complex new types of textures created by additive manufacturing [24], clearly showed fine-scale topographic effects of treating FDM parts with acetone vapor, elucidated differences in topographies of conventionally machined parts [25], and compared microgeometries between milling and grinding and contact interactions to determine relations between coefficient of friction and multiscale curvature [26]. In addition to characterizing principle curvatures, tensor analyses determine their directions, which is essential for characterizing anisotropy.

Anisotropy is important because it can significantly affect interactions between surfaces and phenomena that influence, or are influenced by, topographies. Tribological contacts in sheet forming are dependent on the orientation of microgrooves that influence friction [27]. Characterization of anisotropy is important for understanding topographies of honed piston liner [28]. Anisotropy also influences leakage in ball valves [29].

Fourier transforms and autocorrelation functions (ACF) are commonly used to characterize anisotropy over one scale range. Fourier spectra in polar coordinates show power spectrum in all directions. Directions with the largest amplitudes in these power spectra show anisotropy. Autocorrelations are used for periodic or pseudo-periodic motifs. Anisotropy at one scale can be characterized by surface texture ratios (Str, ISO 25178), which are ratios of lengths of fastest decay of ACF in any direction to lengths of slowest decay of ACF in any direction.

Sliding bandpass filtering can be used for multiscale analyses of traditional parameters. Std, texture direction, is the angle where the angular spectrum is the largest. Str, the texture aspect ratio, characterizes the uniformity of surface textures, determined by the ratio of the autocorrelation decay distances in the directions where the auto-correlation function decays to 0.2, by default, the fastest and the slowest. It is one for isotropic surfaces and zero for highly anisotropic surfaces. To calculate these parameters as a function of scale, measurement data are first filtered multiple times with narrow bands, stepping through a range of wavelengths, then calculating Str and Std for all the wavelengths, thereby creating multiscale characterization of anisotropy. Neither of these methods intrinsically provides multiscale characterizations or describes vertical components of anisotropy.

Thomas et al. applied structure functions, or topothesies, to analyze anisotropy [9]. Topothesy was invariant with orientation for isotropic surfaces. For a strongly anisotropic surface, topothesy was shown to be the same in every direction, except parallel to the lay, where it changes dramatically.

Area-scale and length-scale analyses, two multiscale geometric analyses, stem from fractal geometry. Length-scale has been used to analyze anisotropy in anthropology, to discriminate different kinds of dental microwear of fossil hominis to indicate diet [30,31], and in archeology for multiscale discrimination of types of use wear on stone tools with high confidence [32]. Area-scale has been used for finding strong correlations with loading during use at a particular scale [33].

The current paper includes an introduction of a multiscale curvature tensor analysis method, focusing on its potential to indicate anisotropy. Additionally included is a description of a multiscale bandpass filter. Examples of four measured surfaces with textures that provide different kinds of anisotropy are used to compare characterization techniques. These examples are brought, first, as a test for detecting evident anisotropy, and second, to characterize anisotropy and with respect to different scales of observation. The latter cannot be analyzed with conventional methods that characterize at only one scale or large ranges of scales. Multiscale analyses and characterizations have been shown to be important for understanding interaction with topographies and for establishing strong correlations and confident discriminations [2]. Curvature tensors can be helpful in identifying anisotropy by analyzing principal directions. This is not yet in ISO or ASME standards.

## 2. Materials and Methods

### 2.1. Surfaces and Measurements

Four surfaces are studied. Renderings of these surfaces are shown in Figure 2. These are selected to exemplify certain distinct types of anisotropy at different scales. These surfaces are intended to elicit a range of quantitative and qualitative characterization results, that differ scales and that facilitate comparisons and contrasts. These surfaces and their measurements are described below.

MilledC—a convex cylindrical form created by ball-nose end-milling of tool steel. This process creates strongly anisotropic topographies at scales of its cylindrical form and of the stepover between passes in milling. It was measured with a coherence scanning interferometry (CSI), white light interferometer, Wyko RSTPlus (Veeco Instruments, Plainview, NY, USA) with 10× lens. The measured region was 580 × 430 µm, and *x*- and *y*-sampling intervals were 790 and 910 nm respectively.

MilledF—a flat form also created by ball-nose end-milling of tool steel. This process was also expected to create topographies with anisotropies that are strong near the scales of the stepover, although it lacks the larger scale cylindrical form of MilledC. It was also measured with a CSI white light interferometer, Wyko RSTPlus, although with 5×, a magnification lens, giving a measurement region of 1.2 mm (*x*) and 0.9 mm (*y*) and sampling intervals of 1.7 µm (*x*) and 1.9 µm (*y*), which were, also in contrast to MilledC, resampled to 2 µm in both *x* and *y* before any analysis was done.

µEDMed—was created on 316 L stainless steel by µEDM (micro electo-discharge machining) with a discharge energy of 18 nJ. This process creates nominally isotropic topographies at scales larger than the discharge craters. These surfaces were machined by SmalTec (Lisle, IL, USA, www.smaltec.com) using a hydrocarbon-based oil for its dielectric fluid. The surface was measured with a scanning laser confocal microscope equipped with a 405 nm wavelength laser and a 100× objective lens with a numerical aperture of 0.95. The measured regions consist of 1024 × 1024 height samples over 125 × 125 µm, for a sampling interval of 125 nm. The measurements were processed using form removal and modal outlier filtering [34].

L-PBFed—was created using laser powder bed fusion (L-PBF) with 316 L stainless steel powder in a Solutions SLM 125 HL machine. This process creates topographies with subtle anisotropies that are challenging to detect. The measurement was made using CSI (coherence scanning interferometry) with white light on a Sensofar S neox (Sensofar, Barcelona, Spain) instrument with a 50× objective, perpendicular to a wall that had been formed vertically. The measured region was 516 × 516 µm and the sampling interval was 260 nm in both *x* and *y* directions.

Original sampling intervals used in multiscale analysis, related to all four surfaces are shown in Table 1, with scales presented in curvature-related figures.

### 2.2. Multiscale Curvature Tensor Calculations

Anisotropy is analyzed by using curvature tensors to determine the most prominent directions for ridges and valleys. Curvature tensors are calculated from the measured topographies by a 3D normal-based method, advancing Theisel’s work [35]. Topographic measurements are tiled virtually with triangular patches from which normals are calculated. Each triangle is a right-angled isosceles in its (*x*,*y*) projection. Scales of calculation are the lengths of the triangle’s legs, or catheti [10]. Progressive down-sampling is used to increase scales of calculation regularly for multiscale analysis.

**Figure 1:**
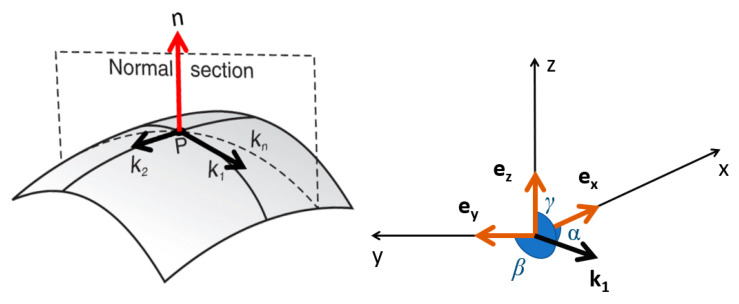
Relations between curvature principal directions **k_1_** and **k_2_** and normal vector and visualization of geometrical direction angles for principal direction **k_1_** in spherical coordinates.

For each group of three tiles, curvature tensors are calculated. The principal directions, **k_1_** and **k_2_** are indicative of anisotropy. A principal direction vector can be decomposed into direction cosines, and then to three directional angles: *α*, *β*, and *γ*, between **k_1_** and the global coordinate system in which the measured topographies are described: **e***_x_*, **e***_y_*, and **e***_z_* (Figure 1). Distributions of *α*, *β*, and *γ*, over all triangular patches, can be plotted for each scale in spherical coordinates. For anisotropic surfaces, an evident peak or peaks in the distribution is expected to appear, indicating the dominant directions. Whereas, for more isotropic surfaces, more uniform distributions are expected. These results can also be visualized in a horizontal coordinate system, also known as topocentric, bfigurey expressing the orientation of **k_1_** in angular coordinates: altitude, or elevation, and azimuth [36]. The reason why a spherical coordinate system is not used is because the polar angle, in that case, is measured from a fixed zenith direction (*z*-axis), whereas in the horizontal coordinate system, elevation is measured from a reference plane, associated with a horizon, which is more intuitive. When principal directions of maximal curvature are coplanar with datum, the elevation is zero, whereas the polar angle would be 90 degrees.

The script that allows calculation of multiscale curvature tensor, including principal directions at each scale and location was created using Mathematica 12 (Wolfram Research, Oxfordshire, UK) computational software. This script is available upon request.

### 2.3. Bandpass Filtering for Multiscale Analyses

Manipulations of datasets, bandpass filtering, and calculations of characterization parameters were performed with MountainsMap^®^ 7.4 software (DigitalSurf, Besançon, France). Non-measured heights in the measurements were filled-in with using smart shape interpolation.

#### Step 1: Defining spatial frequency bands (scales) for bandpass filters and filtering.

Bandpass filters divide topographic data into different scales of observation, i.e., spatial frequency or wavelength bands, reciprocals of each other, for calculating ISO 25178 topographic characterization parameters.

Multiscale bandpass filtering, in this study, uses a sequence of robust Gaussian, combining low-pass and high-pass filters, so that only narrow bands of scales between are left. Mean values of upper and lower nesting indices of bands, centers of cutoff wavelengths, are used to represent scales of observation, or calculation. The bands overlap each other. Nesting indexes are selected with ratios of 1 to 2. There is a 50% overlap between the bands as shown in Table 2. The smallest nesting index possible, using a robust Gaussian filter, is three times the sampling interval (the larger one, if the sampling is different in *x* and *y*). The longest nesting index possible is the length of the shortest edge of the measurement region. Berglund et al. [37] used a similar method in their approach B, although it was plotted differently and they also used Sq for characterizations, which is not used here. Table 2 shows bandpass filter values with the lowest and highest wavelengths used for nesting indices represented by dashes. Note that low-pass and high-pass refer to spatial frequencies, the inverse of wavelengths.

The nature of scales in bandpass filtering differ slightly from those in multiscale curvature analysis. The latter scales are given as multiples of sampling intervals, achieved by down sampling, acting like step functions. The former uses robust Gaussian filters for bandpass filtering, which are not step functions. Wavelengths slightly beyond low and high cut-off wavelengths are included in a bandpass, although with diminishing amplitudes around the cut-offs.

#### Step 2: Calculating conventional topographic characterization parameters.

Texture aspect ratios (Str), texture direction (Std) from ISO 25178-2 are calculated separately for each bandpass scale.

#### Step 3: Creating polar plots from conventional topographic characterization parameters.

Polar plots represent anisotropy symmetrically. Two points are plotted at each scale, with the angle based on Std and the magnitude based Str Distances from the centers are the complement of Str (1 − Str). The angles are Std and its straight angle (St + 180). This provides symmetry, which makes these plots easier to interpret. Using the complement of the texture aspect ratio (1 − Str) portrays stronger anisotropies as larger, i.e., as the magnitudes of the anisotropy with a maximum of one.

## 3. Results

### 3.1. Visual Impressions of Anisotropy

**Figure 2:**
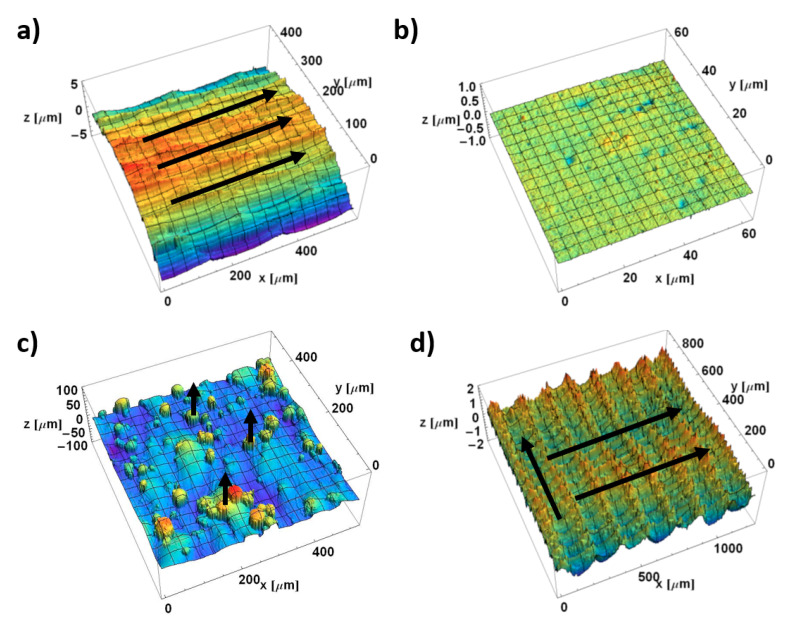
Renderings of measured topographies of (**a**) MilledC, (**b**) µEDMed, (**c**) L-PBFed, and (**d**) MilledF. Please note that black arrows indicate visual impression of apparent anisotropy.

**Figure 3:**
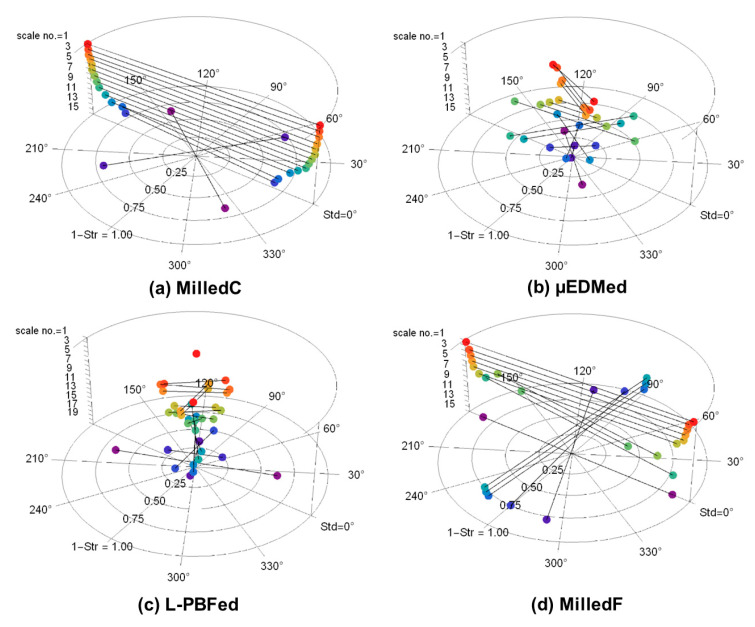
Polar plots showing dominant directions of anisotropy with texture direction (Std) in degrees, magnitude with the complement of the texture aspect ratios (1 − Str), and scales vertically with scale number corresponding to the band numbers from Table 2, with 1 the smallest scale as the highest, calculated for: (**a**) MilledC, (**b**) µEDMed, (**c**) L-PBFed, and (**d**) MilledF.

**Figure 4:**
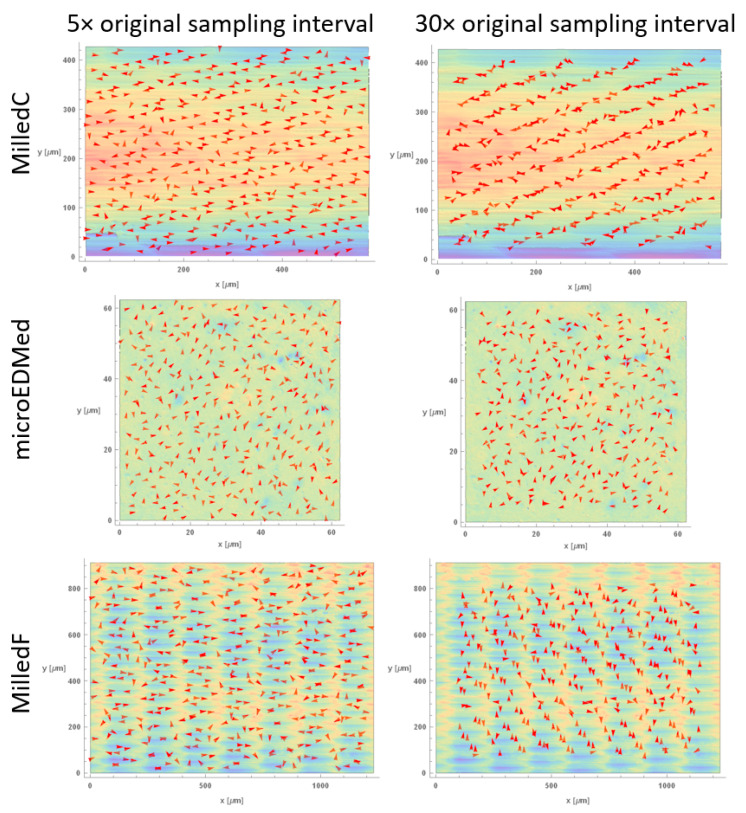
Directions of maximum principal curvatures calculated for MilledC, µEDMed, and MilledF at two scales: 5× and 30× the original sampling interval, plotted together with color-coded height maps. Please note that red arrows indicate a direction of maximum curvature at a given location.

**Figure 5:**
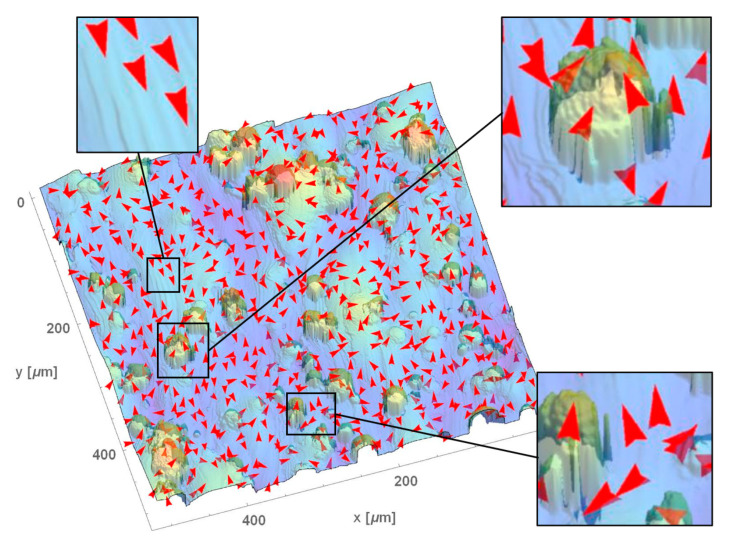
Directions of maximum principal curvature calculated for L-PBFed at the original sampling interval. Please note that red arrows indicate a direction of maximum curvature at a given location.

**Figure 6:**
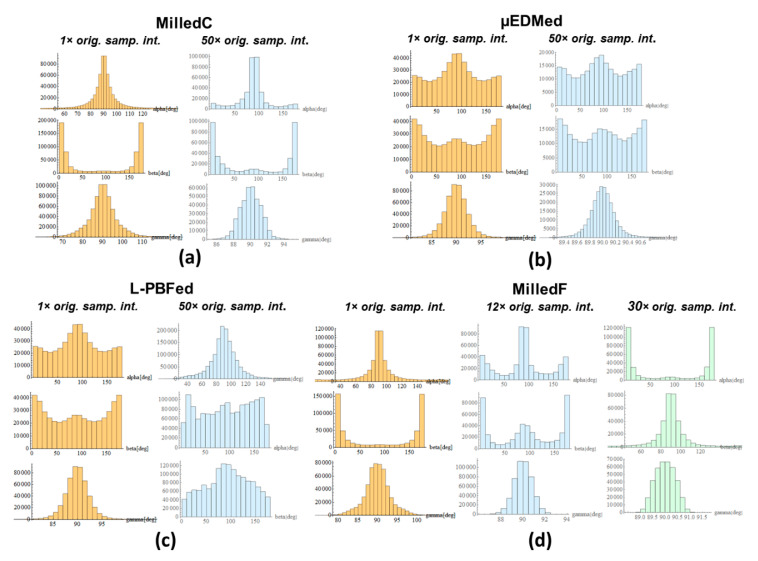
2D histograms created from direction cosines of maximum curvature calculated for (**a**) MilledC, (**b**) µEDMed, (**c**) L-PBFed, and (**d**) MilledF at the indicated scales.

**Figure 7:**
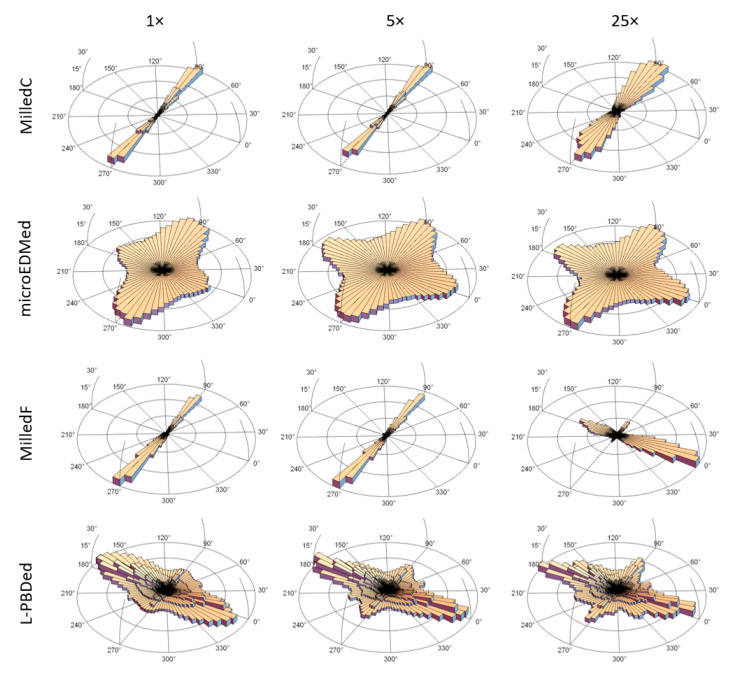
Linear histograms of maximum principal curvature directions in horizontal coordinate system (HCS), i.e., topocentric coordinates at three different scales.

**Figure 8:**
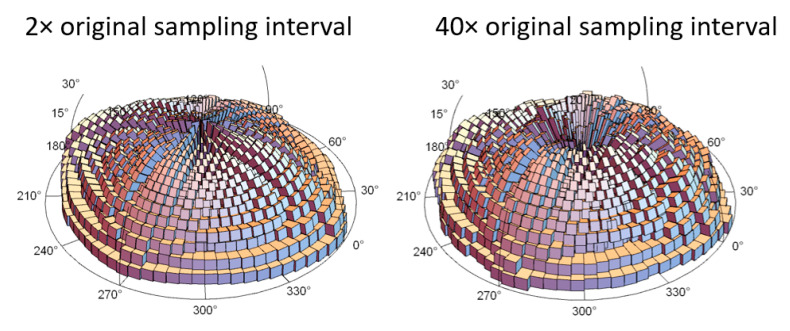
Logarithmic histograms of maximum principal curvature directions in horizontal coordinate system (HCS), i.e., topocentric coordinates at two different scales for the L-PBFed surface.

Visual impressions of anisotropy of all four surfaces can be made from the colored height maps, renderings of their topographic measurements in Figure 2. These impressions are to be compared with results of the two kinds of multiscale analyses described above, curvature tensors and bandpass texture aspect ratios (Str) and texture directions (Std), which are shown in Figure 3, Figure 4, Figure 5, Figure 6, Figure 7 and Figure 8.

Both milled surfaces are clearly anisotropic (Figure 2a,d). Black arrows indicate apparent directionality of characteristic features. MilledC (Figure 2a) exhibits fine scale ridges and valleys that tend to align with the *x*-axis. Its cylindrical form was not removed to show the anisotropy at larger scales. MilledF (Figure 2d) changes with scale. There are troughs and ridges of different scales. At larger scales, they are oriented in *x* and most clearly discernible by subtle variations in color. At smaller scales, they are oriented, as stripes, along *y* and, at even smaller scales, clearly discernible by the distinct repeated cusp shapes of a circular tool nose directed parallel to the *x*-axis.

The EDMed surface appears isotropic (Figure 2b). There are small sized craters created by low-energy electric discharges that appear to be randomly distributed on the surface. Note that the peak-to-valley roughness, as indicated by the vertical scale, is less than the others.

The L-PBF-ed surface (Figure 2c) has subtle ridges and valleys parallel to the *y* axis, aligned with the laser scanning direction used to fuse the powders. It also shows columnar features with steep slopes from partly fused powder particles, indicating a vertical component to the anisotropy. Details on steep slopes cannot be measured with conventional instruments, because the illumination and observation direction are nearly parallel to the sides of the columns.

Renderings of bandpass filtered surfaces that were used to calculate Std and Str for all analyzed bands and surfaces are shown in Supplementary Materials.

### 3.2. Multiscale Characterizations of Surface Anisotropies by Bandpass Filtering

Figure 3 presents polar plots showing anisotropy. A bandpass filter was used for the multiscale decomposition, as described above. The plots show dominant directions, Std, and their magnitudes, as complements of texture aspect ratios (1 − Str), in polar coordinates, for each scale in *z*. The *x* axis in Figure 2 corresponds to 0° in these polar plots. For strongly anisotropic surfaces, the complement of Str is close to one, the full radius of the plots. Central wavelengths of the bandpass filters (Table 2) indicating scales, are shown as band numbers, and increase downwards from smallest to largest. These polar plots facilitate analyses of scale-dependent anisotropies.

The polar plot for MilledC (Figure 3a) shows strong anisotropy at 0° for all scales except the two largest. Anisotropy (1 − Str) decreases with increasing scale, suggesting that there are larger, more directionally varied features. Fine scale features are strongly anisotropic. The two largest scales show dominant directions changing from 0° to 50° and to 143°, respectively. Those last bands are the widest, therefore, they include the widest spectrum of feature sizes, including waviness and cylindrical form. Waviness, that can be seen, might be detected by the Std. In addition, in band 15, there is a slope along the *x*-axis. This due to a misalignment, the surface is not perfectly perpendicular to the *z*-axis of the microscope. This could affect the change in Std from 50° to 143°. The direction along *x*-axis is also significant in this band, but not dominating according to Std and Str.

The polar plot for µEDMed (Figure 3b) shows relatively small magnitudes and directions that change with scale. The least anisotropy is noted for scales of 1.688 and 36 µm and the strongest for scales of 4.5 and 6.75 µm. The scales for which the texture is the most anisotropic cover the diameters of the most evident discharge craters of similar directionality. Those features stand off the isotropic background which corresponds to increasing magnitudes.

The polar plot for L-PBFed (Figure 3c) also shows relatively small anisotropy and directions that change with scale. Apart from the largest scale, the largest magnitude of anisotropy varies between 0.06 to 0.36. The largest scale of analysis, band 19 with a low-pass cut-off of 384.0 µm, can be associated with the direction of laser scanning motion during the sintering process. The direction of solidified material is dominant and other differently oriented features do not play key roles for that scale of calculation. Characterization of anisotropies of pillar features is not possible using this method.

The polar plot for MilledF (Figure 3d) shows strong anisotropy around 0° at the finest scales. This changes dramatically for larger scales, which are 90° shifts for scales from 144 to 522 µm. For the largest scales, there is a return to 0°. For all scales, the complements of the texture aspect ratios are greater than 0.84, indicating strong anisotropy.

### 3.3. Multiscale Characterizations of Surface Anisotropie by the Direction of Maximum Curvature

Figure 4 and Figure 5 show **k_1_**, directions of maximum curvatures, with arrows on height maps surfaces at two scales, five and twenty times their original sampling intervals. Other scales are included in Supplementary Materials. Arrows are plotted for a limited number of regions to improve perceptions. Regions indicating ridges and grooves, that are clearly anisotropic, are visible for MilledC in all scales. This is evident in the similar orientation of **k_1_** for every location. In contrast, for µEDMed, orientations of principal direction of maximum principal curvature vary with region as expected for isotropic surface. For MilledF finer scale orientations of **k_1_** are mostly aligned in *x*, corresponding to anisotropic features created by cutting tool rotation and interactions between the tool edge and workpiece material. For larger scales, arrows are indicative of the bi-directional pattern created by the feed. Visualization of **k_1_** for L-PBFed topography indicate different types of directional features. At the finest scale, wrinkle-like features created by solidifying melt pools can be seen, as well as vertical grooves on the pillar like structures, and valleys between them.

Distributions of angles for directions of maximum curvatures, at each calculation scale, are plotted as 2D histograms in Figure 6 at their original sampling intervals and at forty times their original sampling intervals. More uniform distributions indicate more isotropy, whereas distributions with distinctive single or multiple nonuniformities in their distributions indicate stronger anisotropies in one or more directions.

Multiscale curvature approaches facilitate better characterization of surface anisotropy at multiple scales. This can be done by analyzing 2D histograms of direction angles. Anisotropy is evident if those distributions are unimodal, i.e., a single distinctive peak is present. Stronger anisotropies correspond to smaller standard deviation of those distributions. This effect is visible for MilledC (Figure 6a). For all scales analyzed, distributions of direction angles *α*, *β*, *γ* possess corresponding unique modes at 90°, 0°, or 180° and 90°. With increasing scales, distributions become more concentrated around these values. This could indicate that fine scale features are caused by irregularities in chip formation. This includes built-up edge on the tool that is continuously generated and removed during machining and heterogeneity of material microstructures. All those effects create randomly oriented microfeatures. For larger scales, the cylindrical form and waviness, which are directionality consistent, become more dominant.

For isotropic surfaces, the distributions of direction angles are, in contrast to anisotropic textures, multimodal and more uniform. This effect is visible for µEDMed surface for distributions of *α* and *β* for all scales (see example data in Figure 6b). For isotropic surface, distributions of *α* and *β* are similar. The distribution of *γ* can indicate how the texture orientation differs from *xy*-plane. Ideal vertical features should be characterized by *γ* = 0 degree or *γ* = 180 degree. In case of EDMed topography, distribution of *γ* are highly concentrated at 90 degrees, indicating features mostly oriented in the *xy* plane. The opposite effect can be seen for the L-PBFed surface, where *γ* distributions are more dispersed (Figure 6c). Distributions of *α* and *β* indicate isotropy. Some local anisotropies of *β* equal to 90 degrees, corresponding to laser beam path directions during sintering.

MilledF (Figure 6d) surface shows an anisotropic character that is represented by unimodal distribution of *α*, *β*, and *γ*, like MilledC. This is observed between scales between 1× and 9× as well 35× and 40× original sampling interval. Clear bimodal distributions of *α*, *β* are noted between scales 10× and 25× original sampling interval. The shift in dominant direction by 90 degrees can be seen between scales 26× and 34×, the original sampling interval.

Anisotropy can also be visualized in three-dimensional distributions in the horizontal coordinate system (HCS), i.e., topocentric coordinates. In this method, the entire hemisphere is divided into bins of angular resolutions (5 by 5 degrees Figure 7). Each **k_1_**, is expressed in the HCS and associated certain bin. More **k_1_** vectors in a direction indicates more anisotropy. Some are shown in Figure 7, other scales are in the Supplementary Materials. An azimuth angle of 90 or 270 degrees corresponds to the alignment with *x*-axis, and 0 or 180 degrees to *y*-axis.

Distribution plots in HCS for MilledC confirm its anisotropic characteristics. The dominant orientation of principal direction **k_1_** is aligned with 90 and 270 degree azimuths for all scales, while elevation is mostly below 5 degrees. Some fine scale features appear to be inclined at slightly steeper angles, although this is only visible for scales less than 7.846 µm (10× original sampling interval). These features are located around the cylindrical form, which was intentionally not removed to test this effect. Regardless of those fine scale features, the nature of MilledC anisotropy is generally two-dimensional.

The distribution of **k_1_** for µEDMed examples is evidently more dispersed than MilledC. The dominant azimuths of 0 and 180 degrees, as well as 90 and 270 degrees, relate to the scanning direction of the electrodes during electric discharge machining. Elevation is always less than 5 degrees and is related to general flatness of the topography with some slightly inclined slopes of the discharge craters. This confirms the isotropic nature of µEDMed topographies.

Orientation of **k_1_** in HCS for MilledF show how its anisotropy depends on scale. Between 2 and 18 µm, principal directions of maximum curvatures are aligned with 90 and 270 degree azimuths. Between 20 and 50 µm a second dominant direction is present for azimuth between 0 and 180 degrees. At 60 µm, the first dominant direction is not visible, whereas at 80 µm, the second is not evident. For larger scales, both dominant directions are present. The elevation for all scales analyzed is always close to 0 degrees. This is due to the fact that the form (or the general shape) was removed prior to the analysis.

Other characteristics can be seen for the L-PBFed example. In this case, dominant azimuths of **k_1_** are 0 and 180 degrees, as well as 90 and 270 degrees, although dispersion is visibly greater than for MilledC and MilledF. The elevation is found to be up to 90 degrees for the finest scales, when distribution is presented in a logarithmic scale for magnitude (Figure 8), and it is related to the curvature of pillar like features. Clearly the anisotropy of this surface topography is 3D and scale-dependent.

### 3.4. Conventional Approach Based on Fourier Transform in Polar Coordinates

**Figure 9:**
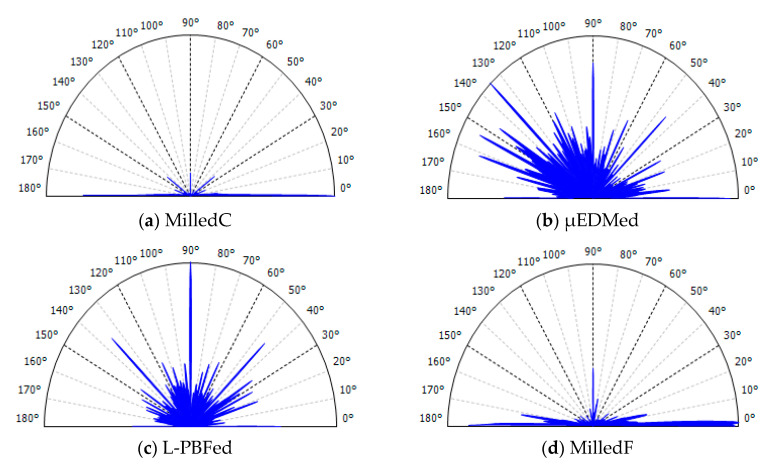
Rosette plots created with a conventional, non-multiscale method using Fourier spectra in polar coordinates, for (**a**) MilledC, (**b**) µEDMed, (**c**) SLMed, and (**d**) MilledF.

Rosette plots created from MilledC and µEDMed topographies can indicate anisotropy and isotropy, which is consistent with indications from bandpass filtering and curvature tensor methods (Figure 9a,b). The limitation of the conventional approach, based on Fourier transform in polar coordinates, becomes more evident for the other two surfaces. For L-PBFed, laser path direction is distinct. No identification and characterization of 3D features is available (Figure 9c). Variation of directionality with scale for MilledF is not shown in Figure 9d, as it appears to be a strongly anisotropic surface, with some weak deviations in perpendicular directions. These results are consistent with expectations based on visual examination of their topographic maps, and intuitive estimations of dominant directions.

## 4. Discussion

Two new, different methods using multiscale analyses and characterization for quantification and visualization of anisotropy are studied here and tested for their ability to elucidate anisotropies. The analysis algorithms for calculating multiscale characterization parameters for determining anisotropy are described. Results of applications to four measured surfaces, manufactured to have distinctly different anisotropies, are examined critically.

Detecting anisotropies by inspection is important, although not a substitute for algorithms, which can automate detection, remove bias, subjectivity, and tedium of multiple inspections, and might, by its consistency, provide insights that would not be detectable by inspection. Inspection should be used to verify results of new algorithms before they are used to investigate surfaces whose significant structures are revealed only by multiscale curvature analyses.

Validation of these methods for elucidating anisotropies as functions of scale is an issue with a philosophical component. Numerical validation of new characterizations of this kind is problematic because they extend current possibilities beyond current experience. There are no other tests that can produce essentially similar results. Over time, the value of new characterizations might be established, and this could be another kind of validation. For that to happen, papers must be published to disseminate this new knowledge. In an initial work, as here, new methods can be compared with each other. Here, several kinds of representations are used to facilitate these comparisons. Consequently, it is learned that they are consistent with each other. They fulfill reasonable expectations for discriminating measurements from topographies with anisotropies that are known to be different.

These two methods are compared in several different kinds of representations, which are intended to reveal anisotropic properties. These elucidate differences between each surface, with impressions from qualitative visual inspection, and with conventional rosette plots of Fourier analyses. One new multiscale method uses multiple bandpass filters (also known as sliding bandpass filtering) which are applied to measured topographic data prior to calculating conventional anisotropy characterization parameters, texture aspect ratios (Str), and texture directions (Std). These detect anisotropy in two dimensions, parallel to a datum plane. The other uses multiscale curvature tensor analysis to characterize topographies with principal curvatures and their orientation in three dimensions, at each location and multiple scales. The main limitations of sliding bandpass filtering is that it only allows characterization of a single dominant direction at each scale. However, it detects changes in anisotropy with scale, which is not possible with conventional non-multiscale approach using autocorrelation functions, for example. That approach can only be used for 2D anisotropy analysis conducted at a nominal scale, i.e., original sampling interval.

The anisotropies detected by these two multiscale methods correspond well to those that are evident by visual inspection of height maps, and in a more limited sense, to conventional rosette plots of Fourier spectra. Visual inspections can detect changes in anisotropy with scale, whereas conventional rosette plots of Fourier spectra are limited because they cannot.

Determining specific scales of anisotropy is important because, according to Brown et al. [2], some topographically dependent phenomena have certain scales, or narrow scale ranges, over which they interact with topographies. These scales can be advantageous or disadvantageous for certain kinds of performance. Anisotropies can be created at different scales. It is important to be able to recognize these scales for product and process design. They can also be important in physical anthropology and forensics [30].

Multiscale curvature tensor analysis provides the most knowledge about anisotropy of any of the methods studied here. Results of this analysis characterize anisotropy so that it can be represented in horizontal coordinate systems i.e., topocentric coordinates, which show both horizontal and vertical components, providing a true three-dimensional representation of anisotropy.

Multiscale curvature tensor analyses provide characterization and visualization of directionality of subregions. This method, unlike conventional and sliding bandpass filtering, can also be used to characterize features which are directed perpendicularly from nominal or datum plane. This is particularly important for characterizing freeform surfaces and surface manufactured additively by laser fusion. The later can have especially intricate topographies at fine scales due to partly fused particles.

The term “scale” is used in this study differently in the two multiscale analyses. Therefore, a direct quantitative comparison might not be possible. From a qualitative perspective, they both indicate anisotropy changes with scales similarly. In sliding bandpass filtering, scale refers to central wavelengths and widths of bands [11,37]. In geometric multiscale methods, scale is associated with geometrical parameters like length, area, filled volumes or areas, and curvature [1]. In this multiscale curvature analysis, scale is linked with the size of a triangular patch [10] into which the original mesh is divided. The curvature tensor analysis uses what is essentially down sampling. The scale is more specific and tied to multiples of sampling intervals, i.e., pixel sizes. From a qualitative perspective, they both similarly indicate anisotropy changes with scales. The two different manifestations of scale exacerbate precise quantitative comparison of results from these two different methods. However, these two methods similarly indicate the changes in anisotropy with scale in all four analyzed examples.

A limitation of multiscale analysis is its computation complexity, so it can be time-consuming. Maleki et al. showed that curvature tensor calculation required the most time when compared to other existing methods [38]. On the other hand, that study concluded that it performed the best, together with the Bigerelle–Nowicki method, in terms of quality for the analyzed test scenarios.

Additively manufactured surfaces present new challenges for characterizations. The surfaces of metal PBF (powder bed fusion) components are typically highly irregular, with steep sided and re-entrant features [5]. Relevant surface features exist at a wide range of scale, therefore a scale-based characterization becomes of great importance. Being datum-independent, curvature seems to be a prospective candidate for the analysis of AM surfaces in terms of anisotropy. Further research will focus on characterizing complex 3D, freeform structures, and measurements by microCT.

Multiscale curvature tensor analyses are a valuable tool for elucidating changes in anisotropy caused by processing, and for indicating performance, such as sealing, lubrication, and friction. These also require appropriate statistical analyses, which could describe the complexity of the anisotropy expressed as histograms of maximum principal curvature directions in horizontal coordinate system. Potential candidates for visualization should also portray bivariate characters of distributions, as both azimuth and elevation angles are considered together. Potential candidates include measures of distribution, i.e., modes, means and medians, dispersion and associated, such as bivariate mean deviation, total variation, or generalized variance. Higher moments like bivariate skewness and kurtosis can also be relevant [39]. Tracking changes with scale, should be additional tools to enumerate effects of surface processing on anisotropy. This is especially important in physical anthropology, paleontology, and archaeology, where directions of topographic features found in artifacts indicate their function [2,30,31]. Sophisticated indications of anisotropy by multiscale curvature tensors can improve manufacturing processes diagnostics, e.g., in the detection and characterization of tool wear [40], and help to understand its impact on performance of resulting topographies [41].

The main advantage of conventional analyses is that they are incorporated in commercial software, and therefore used extensively by the industry and academia. New characterization methods, like multiscale, will be welcomed, if they add value by advancing the understanding of the relations between topographies and phenomena. This could be facilitated by more intuitive, automated, and easy-to-use software developed and distributed for industrial use. Some efforts have already been made by including multiscale profile and areal analyses and bandpass filtration (profiles only), as easy-to-use features in commercial software. Multiscale curvature analyses, and bandpass filtering for areal datasets prior to calculating traditional parameters, should follow the same path.

## 5. Conclusions

Two new multiscale methods for quantification and visualization of anisotropy are described, examined critically, and compared logically each other, with impressions from qualitative visual inspection, and with conventional rosette plots of Fourier analyses. One new multiscale method uses multiple bandpass filters (also known as sliding bandpass filtering) which are applied to measured topographic data prior to calculating conventional anisotropy characterization parameters, texture aspect ratios (Str), and texture directions (Std). These detect anisotropy in two dimensions, parallel to a datum plane. The other uses multiscale curvature tensor analysis to characterize topographies with principal curvatures and their orientation in three dimensions, at each location and multiple scales.

The anisotropies detected by these two multiscale methods correspond well to those that are evident by visual inspection of height maps, and in a more limited sense, to conventional rosette plots of Fourier spectra. Visual inspections can detect changes in anisotropy with scale, whereas conventional rosette plots of Fourier spectra are limited because they cannot.

Both these new, multiscale methods can show clearly that anisotropy can change with scale on actual surfaces with markedly different anisotropies.

Changes of anisotropy with scale categorically cannot be detected by traditional characterization methods used conventionally, e.g., Fourier spectra.

Multiscale curvature tensor analysis shows anisotropy in horizontal coordinate systems (HCS), i.e., topocentric, with both horizontal and vertical components, which are a true, three-dimensional representations of anisotropy.

With the bandpass approach, polar plots elucidate anisotropy at specific scales. Multiple plots, at different scales, can be combined and used to show the multiscale nature of different sorts of anisotropies. These polar plots show orientations of anisotropy of texture directions (Std) in degrees, magnitudes in the radial direction are derived from complements of texture aspect ratios (1 − Str), and scales are shown vertically. However, only a single dominant direction can be indicated at each scale.

Directions of principal curvatures superimposed on height maps also elucidate anisotropies at specific scales. Different scales show the multiscale nature of different sorts of anisotropies.

Histograms, showing frequency distributions, created from direction cosines of maximum principal curvatures are another way of elucidating anisotropies as a function of scale. These can either be plotted conventionally in cartesian coordinates, or with hemispherical-type histograms of maximum principal curvatures directions using horizontal coordinate systems (HCS), i.e., topocentric coordinates.

## Figures and Tables

**Table 1:** Original sampling intervals used in the multiscale analysis.

Surface	MilledC	MilledF	µEDMed	L-PBFed
Original sampling interval [µm]	0.790	2.000	0.125	0.260
5× original sampling interval [µm]	3.950	10.000	0.625	1.300
20× original sampling interval [µm]	15.800	40.000	2.500	5.200
25× original sampling interval [µm]	19.750	50.000	3.125	6.500
40× original sampling interval [µm]	31.600	80.000	5.000	10.400

**Table 2:** Wavelengths of the nesting indices, center, low-pass, and high-pass for multiscale (also known as sliding) bandpass filtering, all in [µm].

	MilledC			µEDMed			L-PBFed			MilledF		
No.	Center	Low	High	Center	Low	High	Center	Low	High	Center	Low	High
1	3.0	-	4.0	0.422	-	0.563	1.1	-	1.5	6	-	8
2	4.5	3.0	6.0	0.563	0.375	0.750	1.5	1.0	2.0	9	6	12
3	6.0	4.0	8.0	0.844	0.563	1.125	2.3	1.5	3.0	12	8	16
4	9.0	6.0	12.0	1.125	0.750	1.500	3.0	2.0	4.0	18	12	24
5	12.0	8.0	16.0	1.688	1.125	2.250	4.5	3.0	6.0	24	16	32
6	18.0	12.0	24.0	2.250	1.500	3.000	6.0	4.0	8.0	36	24	48
7	24.0	16.0	32.0	3.375	2.250	4.500	9.0	6.0	12.0	48	32	64
8	36.0	24.0	48.0	4.500	3.000	6.000	12.0	8.0	16.0	72	48	96
9	48.0	32.0	64.0	6.750	4.500	9.000	18.0	12.0	24.0	96	64	128
10	72.0	48.0	96.0	9.000	6.000	12.000	24.0	16.0	32.0	144	96	192
11	96.0	64.0	128.0	13.500	9.000	18.000	36.0	24.0	48.0	192	128	256
12	144.0	96.0	192.0	18.000	12.000	24.000	48.0	32.0	64.0	270	192	348
13	192.0	128.0	256.0	27.000	18.000	36.000	72.0	48.0	96.0	384	256	512
14	270.0	192.0	348.0	36.000	24.000	48.000	96.0	64.0	128.0	522	348	696
15	384.0	256.0	-	48.000	32.000	-	144.0	96.0	192.0	768	512	-
16	N/A	N/A	N/A	N/A	N/A	N/A	192.0	128.0	256.0	N/A	N/A	N/A
17	N/A	N/A	N/A	N/A	N/A	N/A	288.0	192.0	384.0	N/A	N/A	N/A
18	N/A	N/A	N/A	N/A	N/A	N/A	384.0	256.0	512.0	N/A	N/A	N/A
19	N/A	N/A	N/A	N/A	N/A	N/A	576.0	384.0	-	N/A	N/A	N/A

## Supplementary Materials

The following are available online at https://www.mdpi.com/1996-1944/13/13/3028/s1, as a supplement to this study, authors include the bandpass filtered surfaces created by robust Gaussian filtration as described in the paper. Additional histograms of maximum principal curvature directions in horizontal coordinate system (HCS), together with plots representing directions of maximum principal curvatures, calculated for all four analyzed surfaces, are also included in the supplement.
