# Supplementary figures and images for: Multiscale Characterizations of Surface Anisotropies (part 2 of 2)
Source: Materials (Basel). 2020 Jul 7;13(13):3028. doi: 10.3390/ma13133028 (PMC7372363; doi:10.3390/ma13133028)

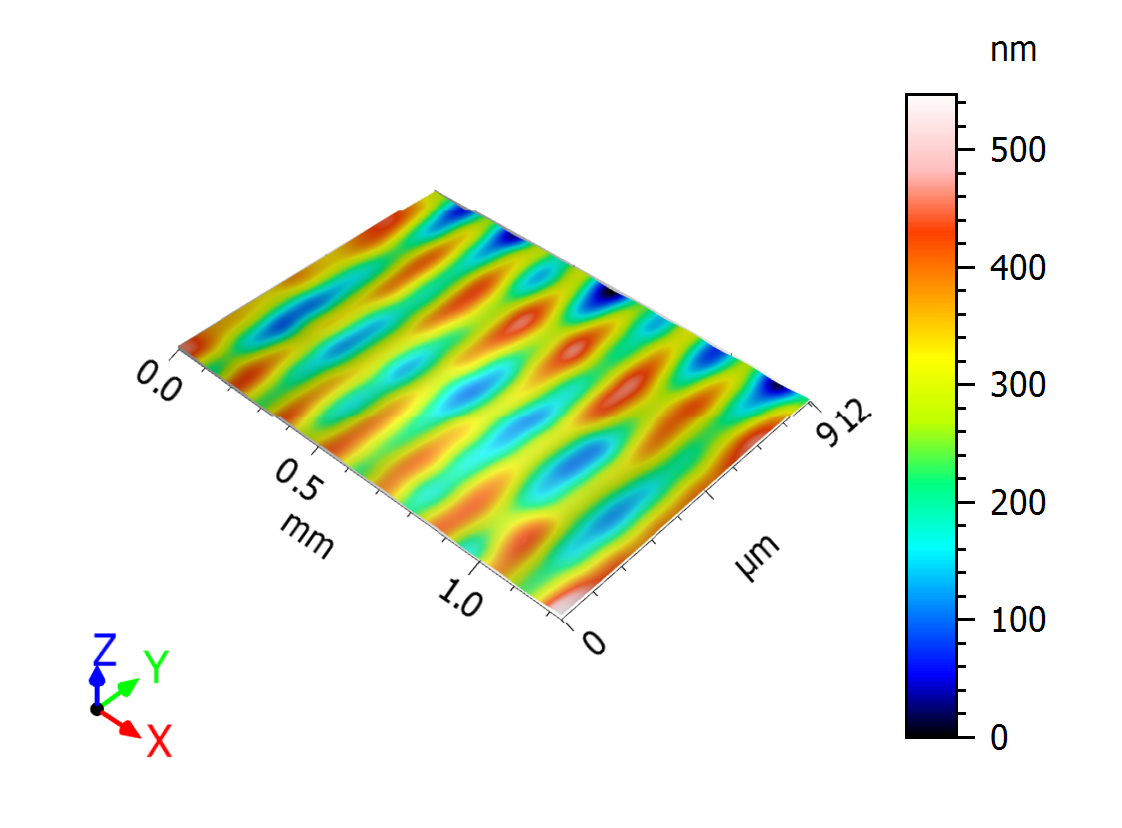

Supplement: Supplementary file 1 [file materials-13-03028-s001.zip › supplementary data/Bandpass filtering/3d_images_filtered_surfaces/MilledF_Number=12_CentralWavelength=270.png]

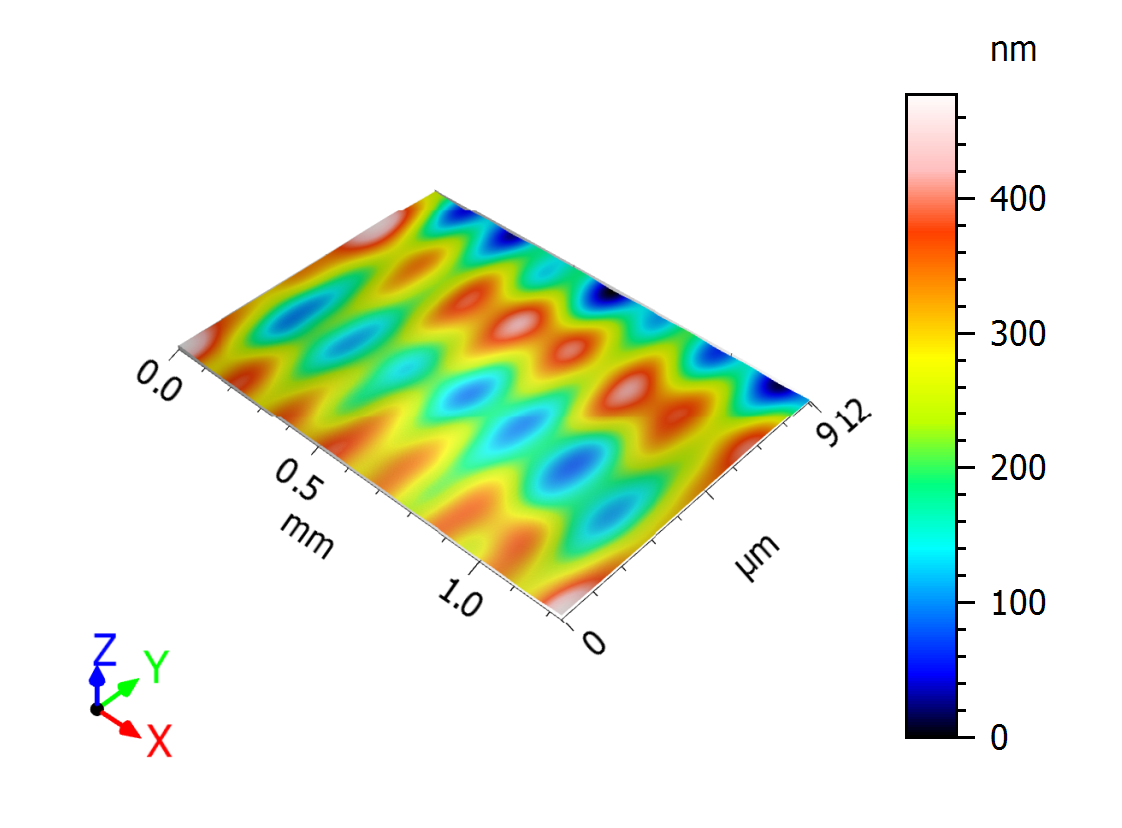

Supplement: Supplementary file 1 [file materials-13-03028-s001.zip › supplementary data/Bandpass filtering/3d_images_filtered_surfaces/MilledF_Number=13_CentralWavelength=384.png]

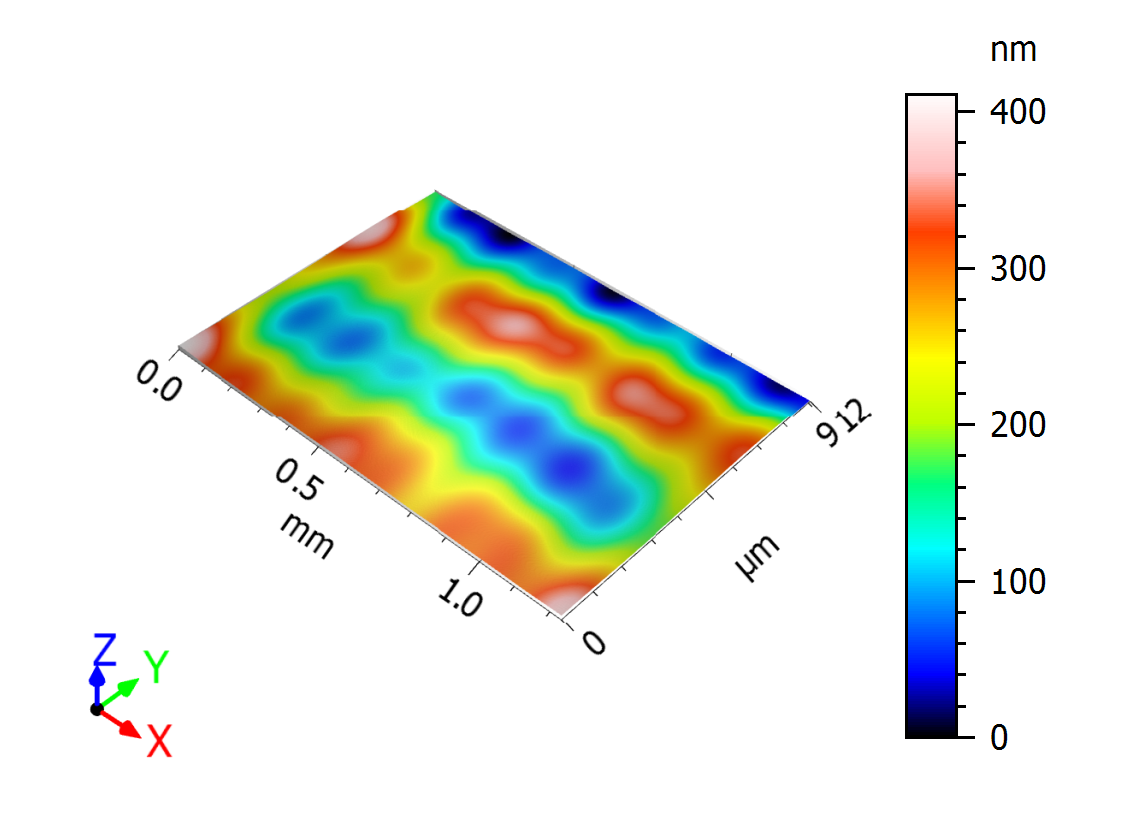

Supplement: Supplementary file 1 [file materials-13-03028-s001.zip › supplementary data/Bandpass filtering/3d_images_filtered_surfaces/MilledF_Number=14_CentralWavelength=522.png]

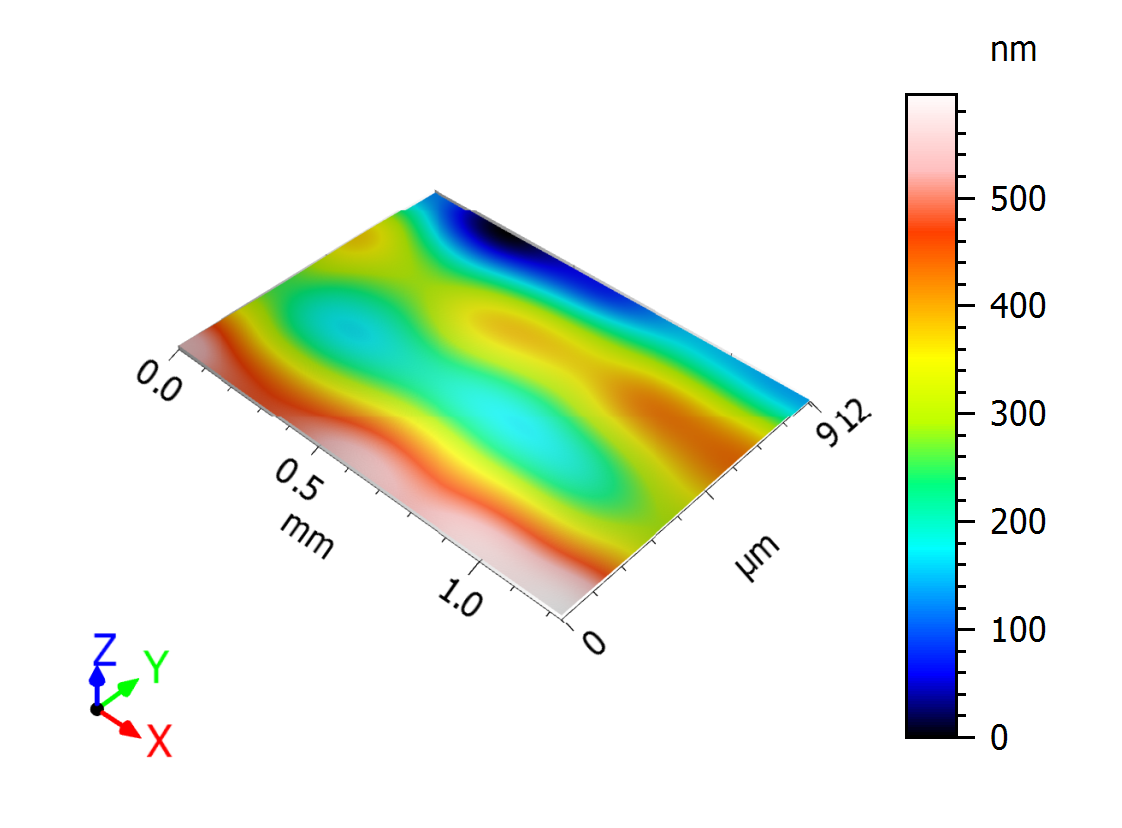

Supplement: Supplementary file 1 [file materials-13-03028-s001.zip › supplementary data/Bandpass filtering/3d_images_filtered_surfaces/MilledF_Number=15_CentralWavelength=768.png]

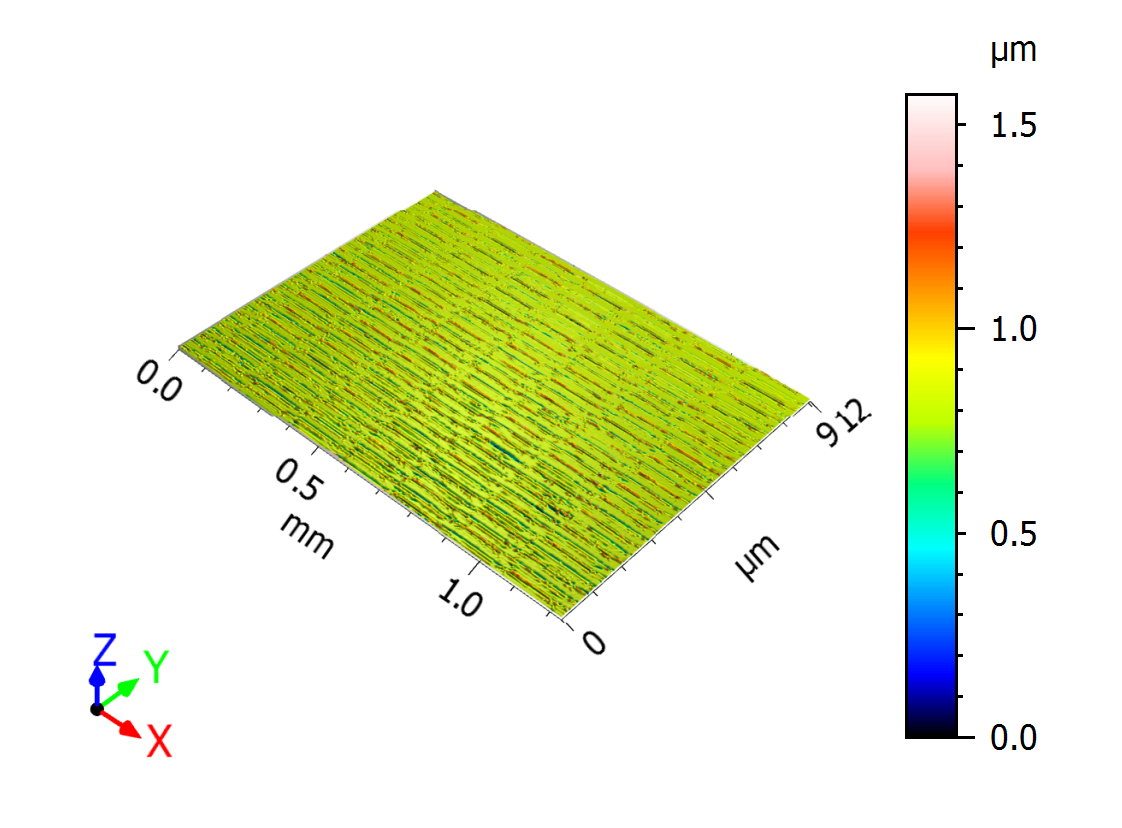

Supplement: Supplementary file 1 [file materials-13-03028-s001.zip › supplementary data/Bandpass filtering/3d_images_filtered_surfaces/MilledF_Number=1_CentralWavelength=6.png]

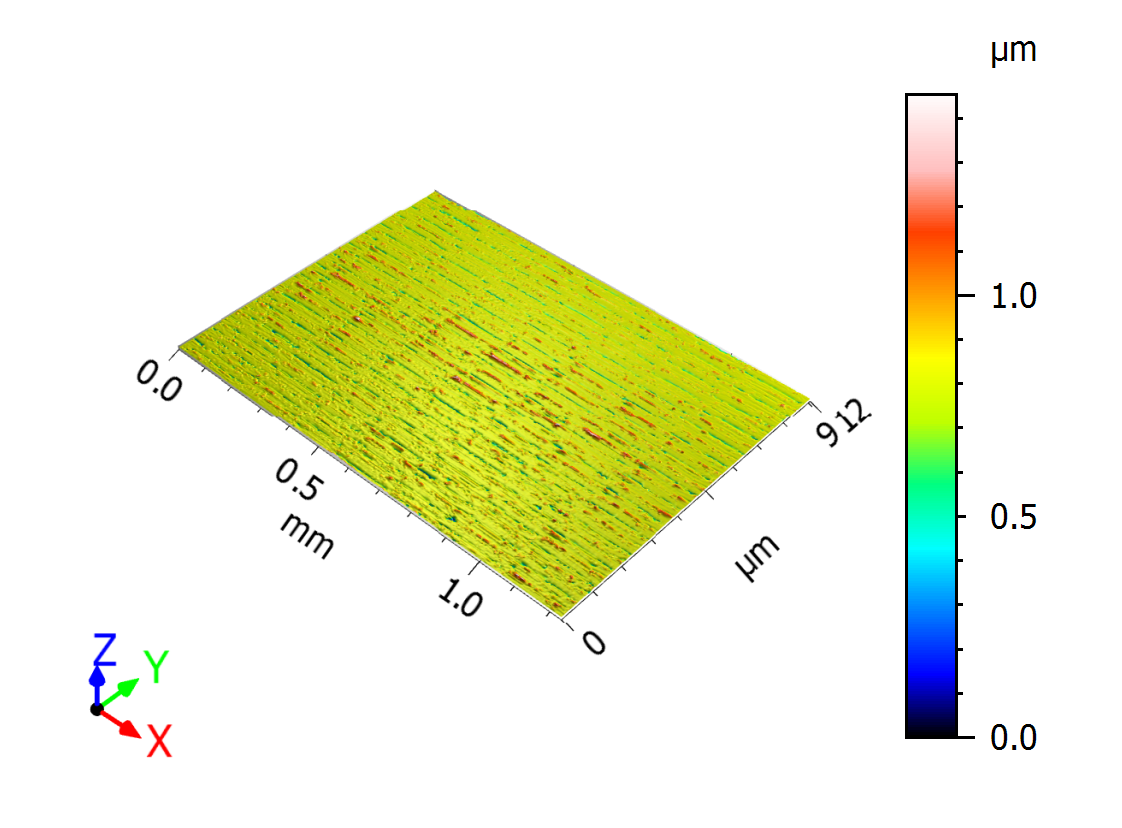

Supplement: Supplementary file 1 [file materials-13-03028-s001.zip › supplementary data/Bandpass filtering/3d_images_filtered_surfaces/MilledF_Number=2_CentralWavelength=9.png]

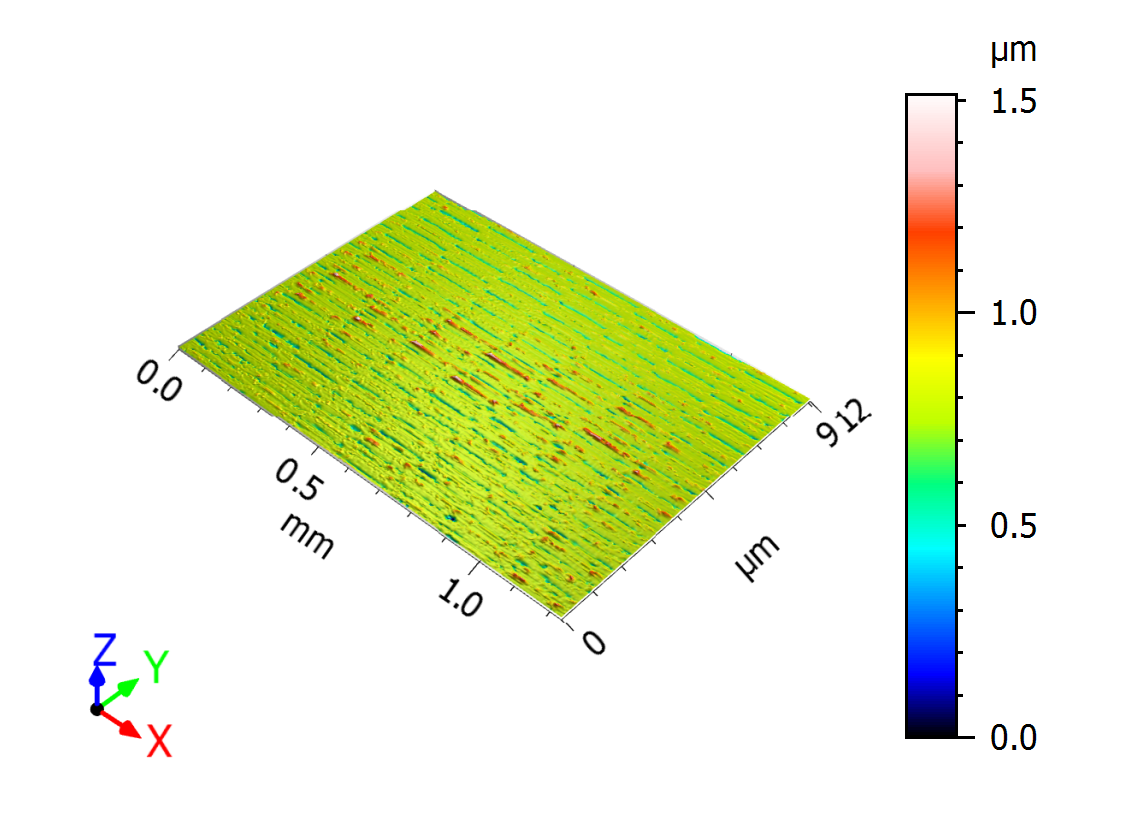

Supplement: Supplementary file 1 [file materials-13-03028-s001.zip › supplementary data/Bandpass filtering/3d_images_filtered_surfaces/MilledF_Number=3_CentralWavelength=12.png]

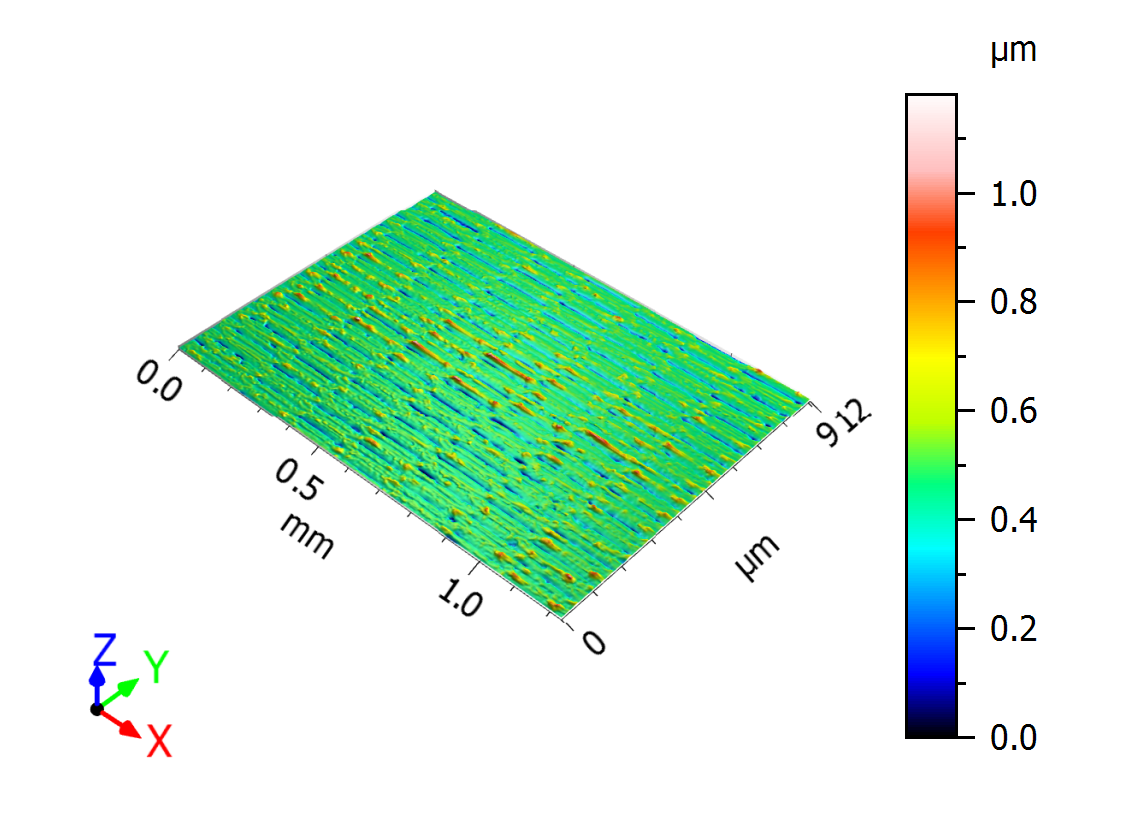

Supplement: Supplementary file 1 [file materials-13-03028-s001.zip › supplementary data/Bandpass filtering/3d_images_filtered_surfaces/MilledF_Number=4_CentralWavelength=18.png]

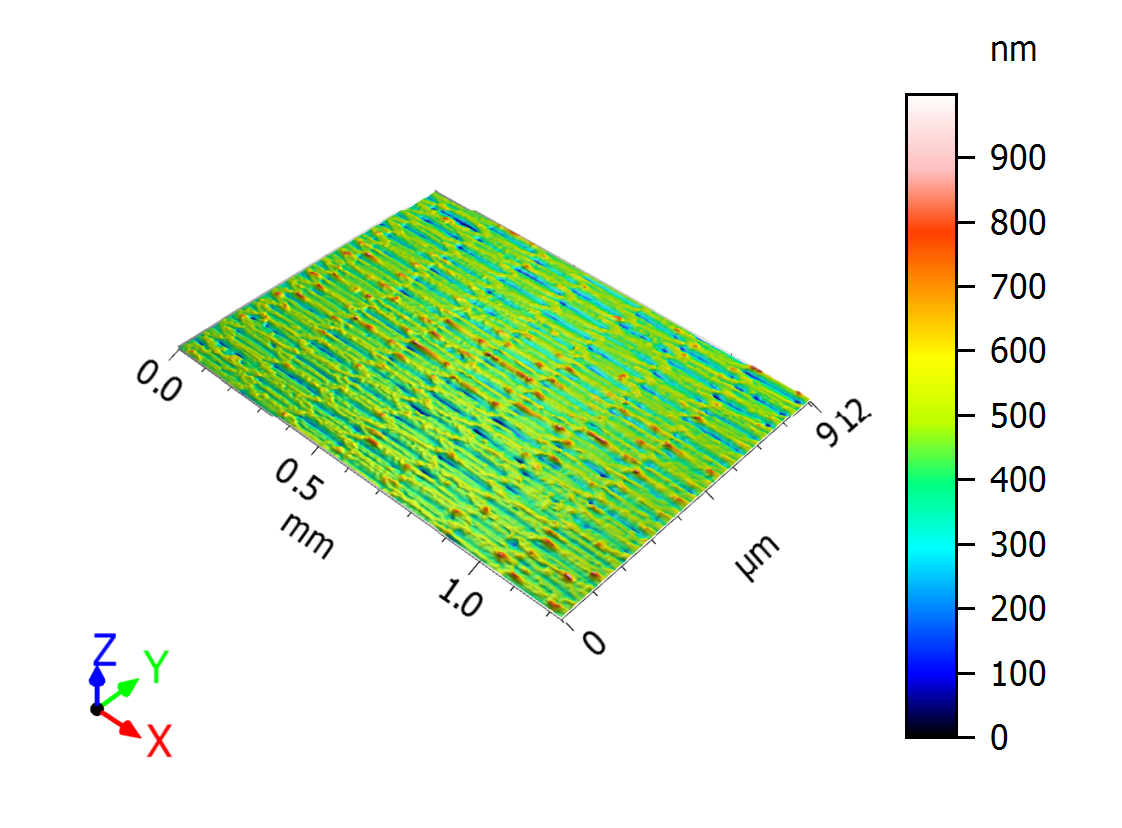

Supplement: Supplementary file 1 [file materials-13-03028-s001.zip › supplementary data/Bandpass filtering/3d_images_filtered_surfaces/MilledF_Number=5_CentralWavelength=24.png]

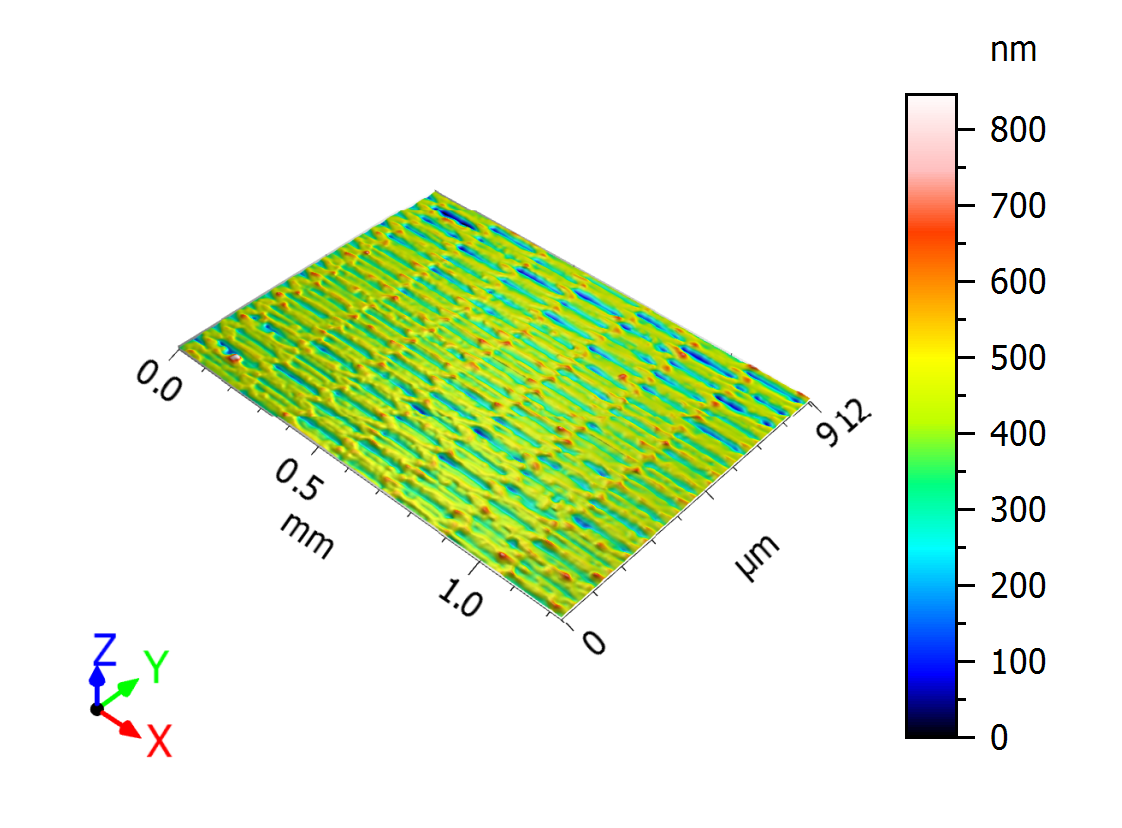

Supplement: Supplementary file 1 [file materials-13-03028-s001.zip › supplementary data/Bandpass filtering/3d_images_filtered_surfaces/MilledF_Number=6_CentralWavelength=36.png]

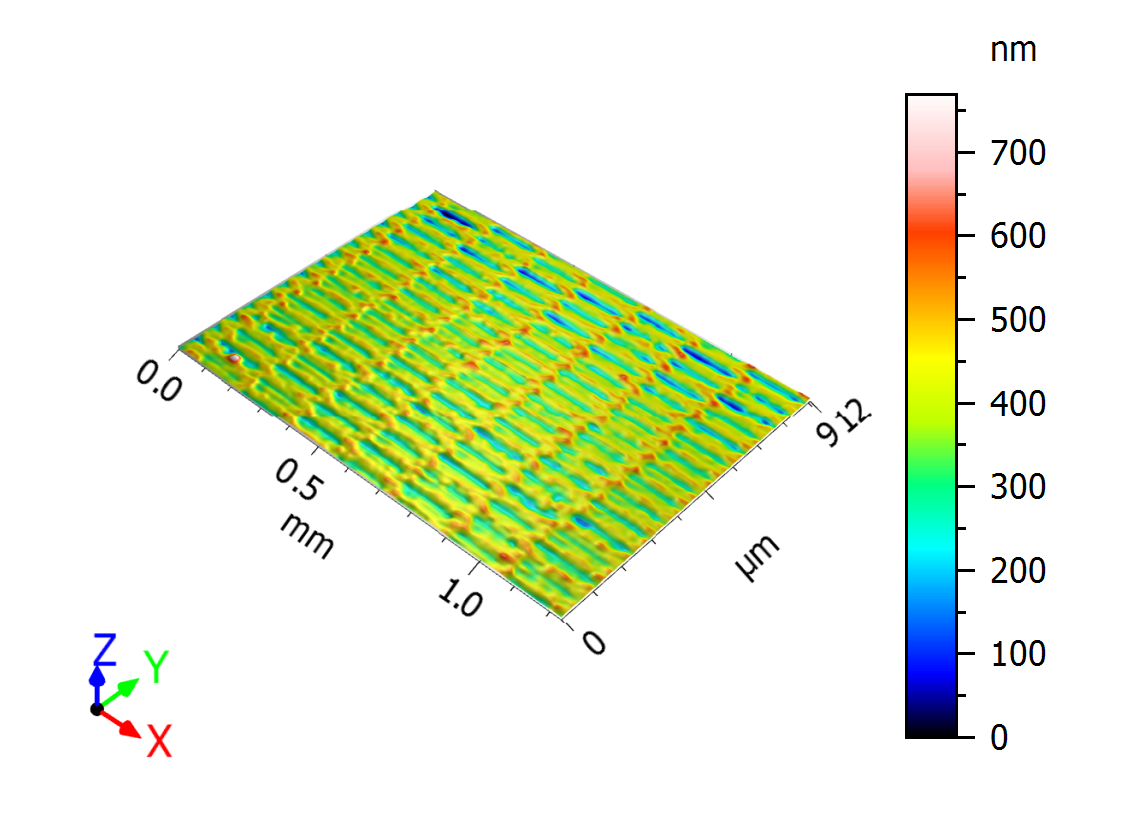

Supplement: Supplementary file 1 [file materials-13-03028-s001.zip › supplementary data/Bandpass filtering/3d_images_filtered_surfaces/MilledF_Number=7_CentralWavelength=48.png]

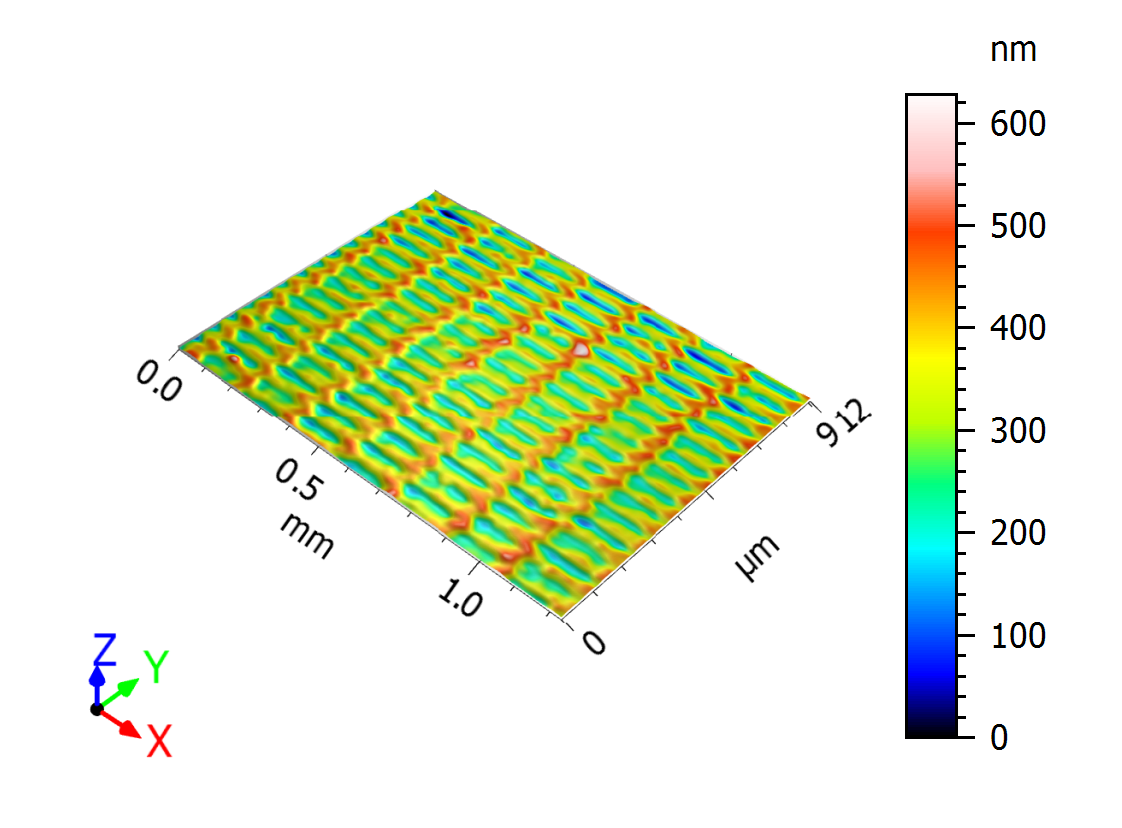

Supplement: Supplementary file 1 [file materials-13-03028-s001.zip › supplementary data/Bandpass filtering/3d_images_filtered_surfaces/MilledF_Number=8_CentralWavelength=72.png]

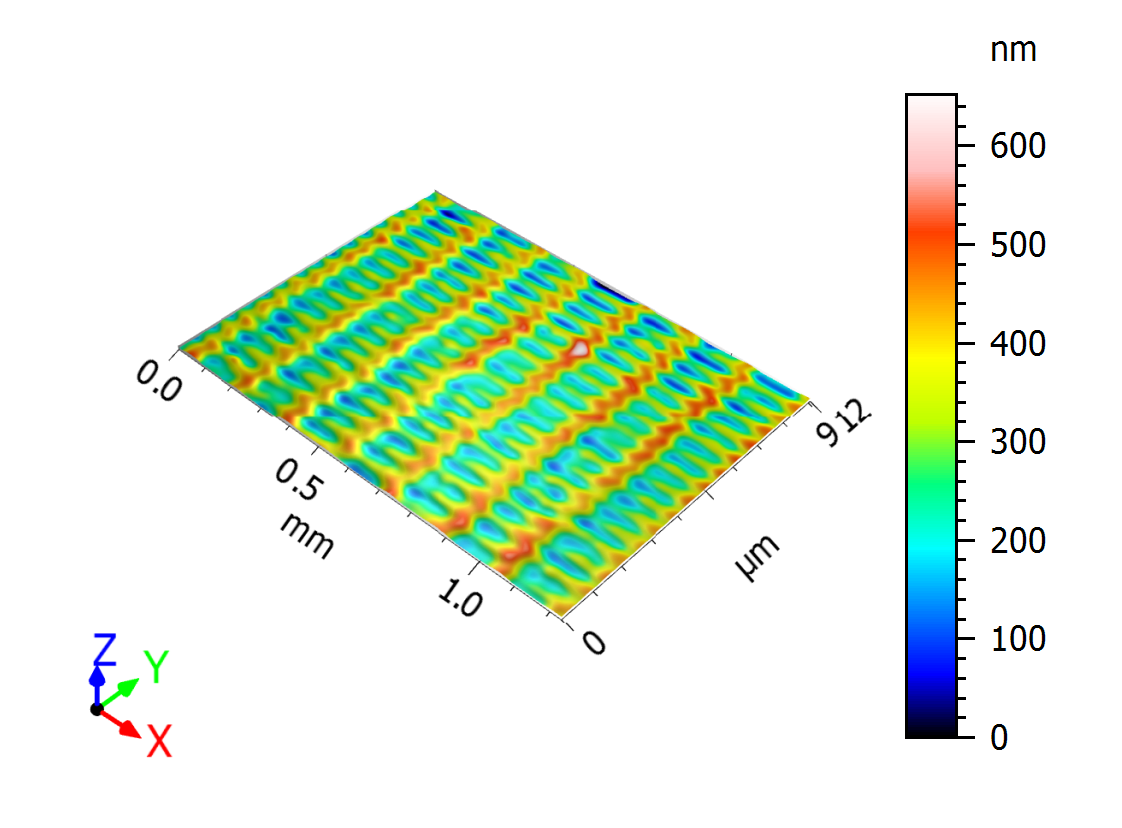

Supplement: Supplementary file 1 [file materials-13-03028-s001.zip › supplementary data/Bandpass filtering/3d_images_filtered_surfaces/MilledF_Number=9_CentralWavelength=96.png]

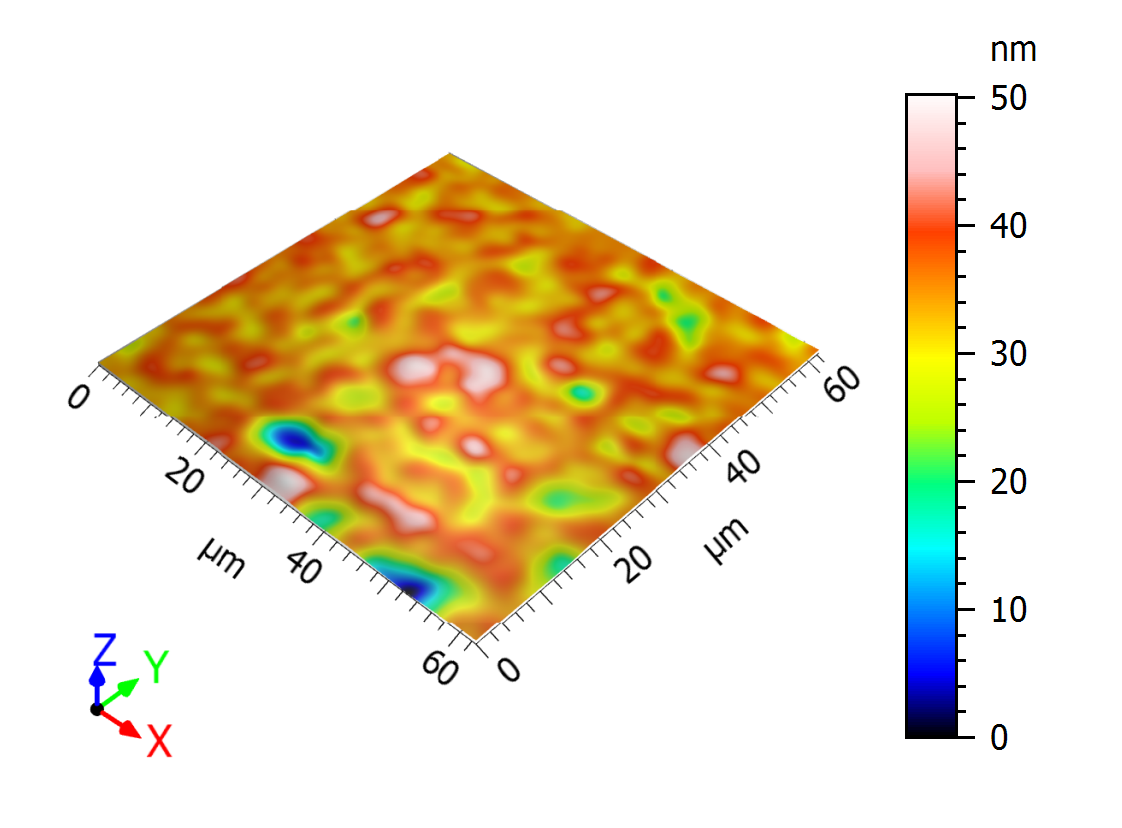

Supplement: Supplementary file 1 [file materials-13-03028-s001.zip › supplementary data/Bandpass filtering/3d_images_filtered_surfaces/μEDMed_Number=10_CentralWavelength=9.png]

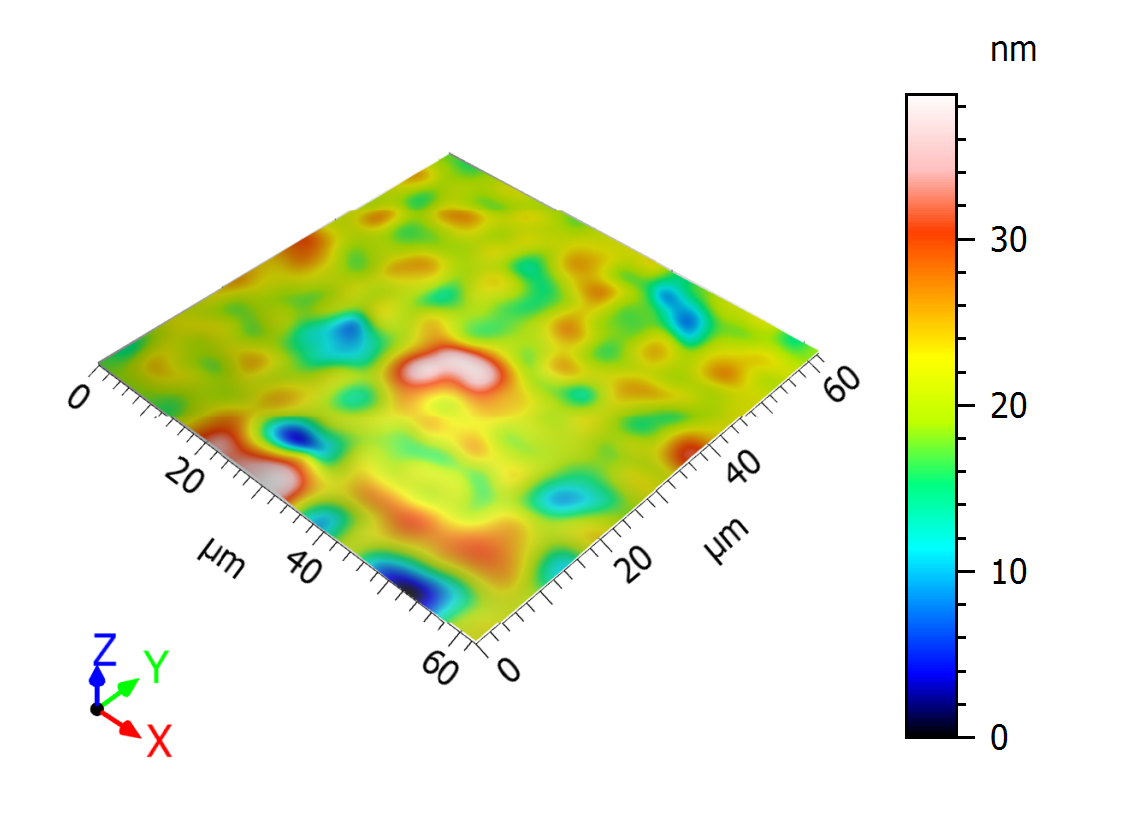

Supplement: Supplementary file 1 [file materials-13-03028-s001.zip › supplementary data/Bandpass filtering/3d_images_filtered_surfaces/μEDMed_Number=11_CentralWavelength=13.5.png]

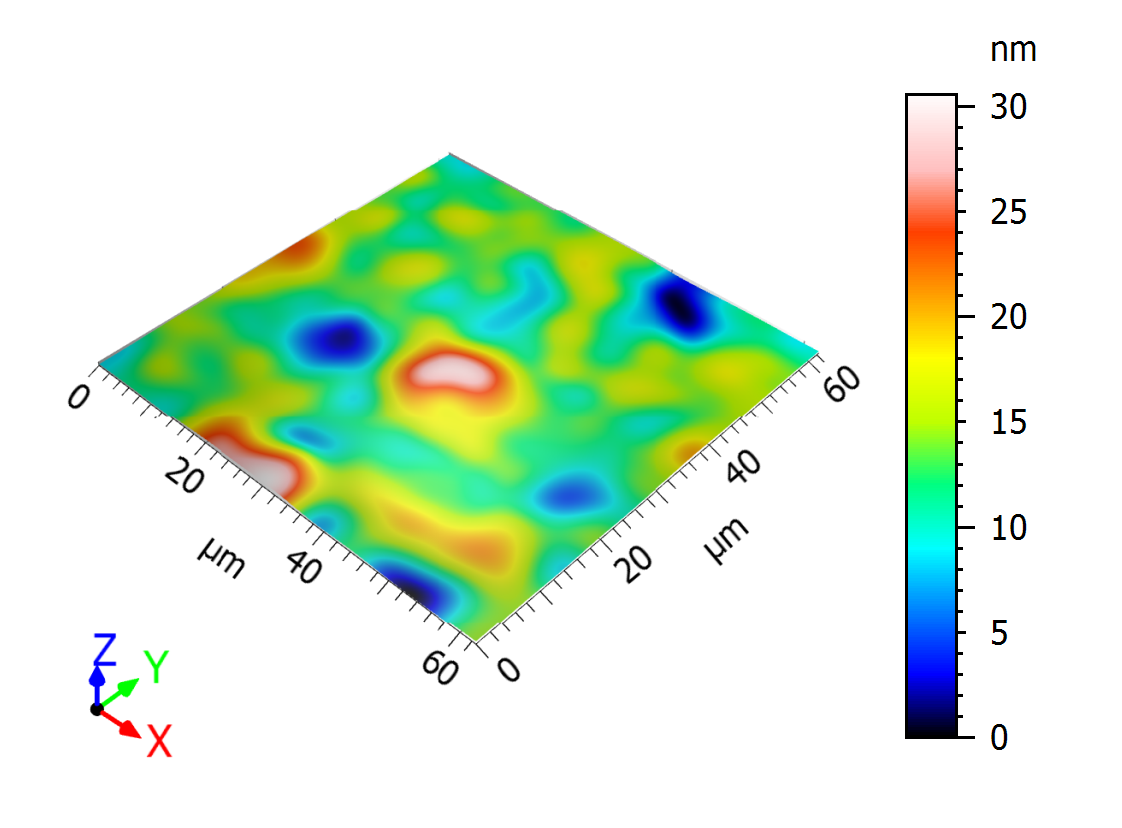

Supplement: Supplementary file 1 [file materials-13-03028-s001.zip › supplementary data/Bandpass filtering/3d_images_filtered_surfaces/μEDMed_Number=12_CentralWavelength=18.png]

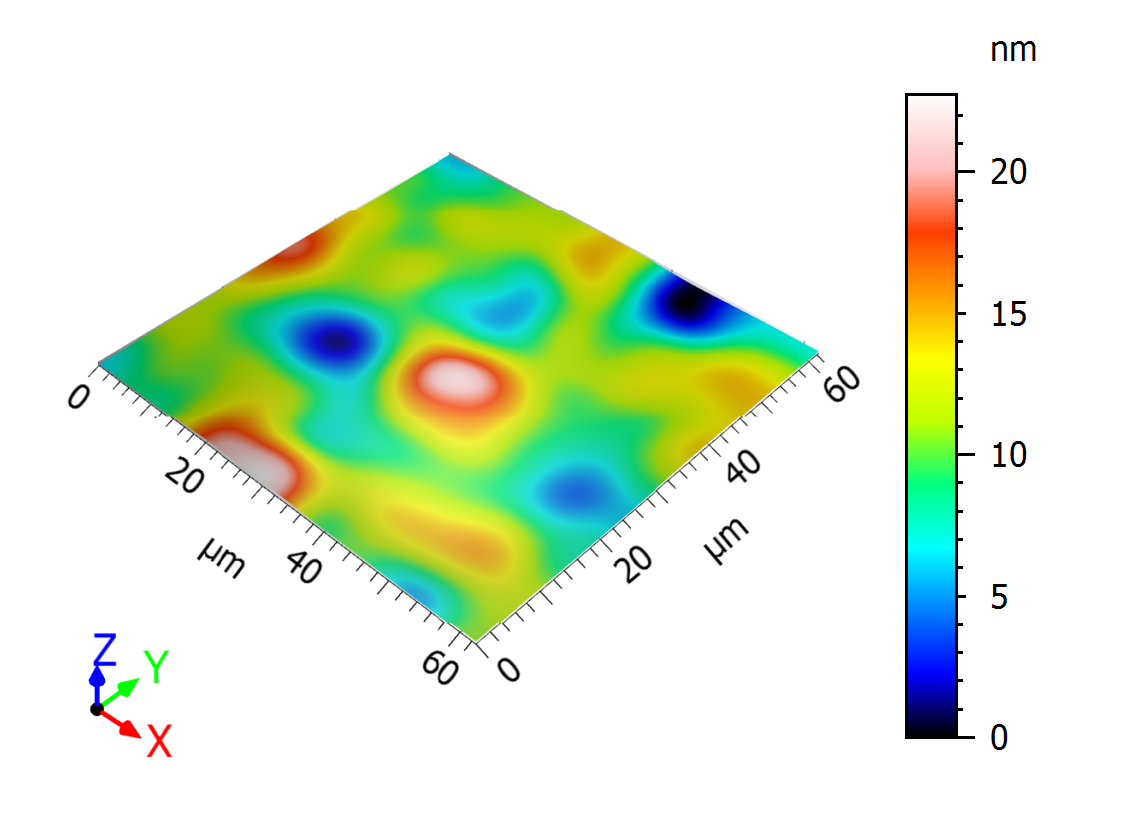

Supplement: Supplementary file 1 [file materials-13-03028-s001.zip › supplementary data/Bandpass filtering/3d_images_filtered_surfaces/μEDMed_Number=13_CentralWavelength=27.png]

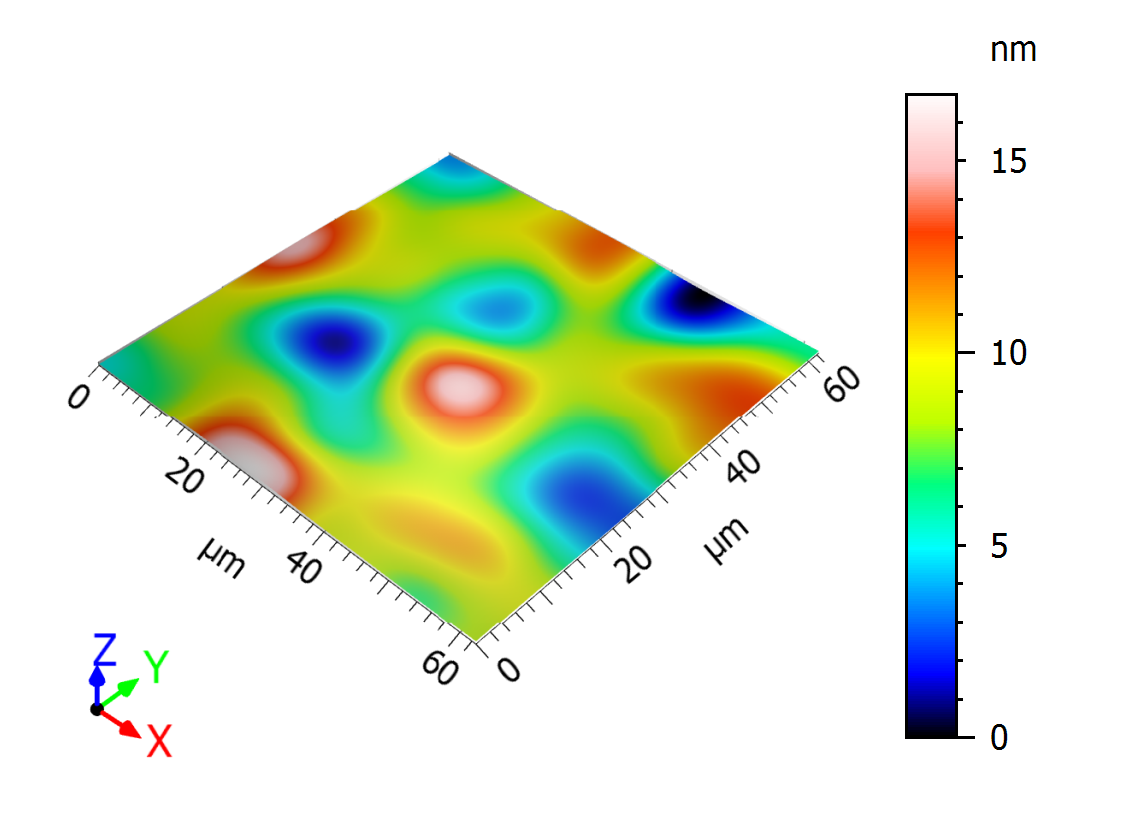

Supplement: Supplementary file 1 [file materials-13-03028-s001.zip › supplementary data/Bandpass filtering/3d_images_filtered_surfaces/μEDMed_Number=14_CentralWavelength=36.png]

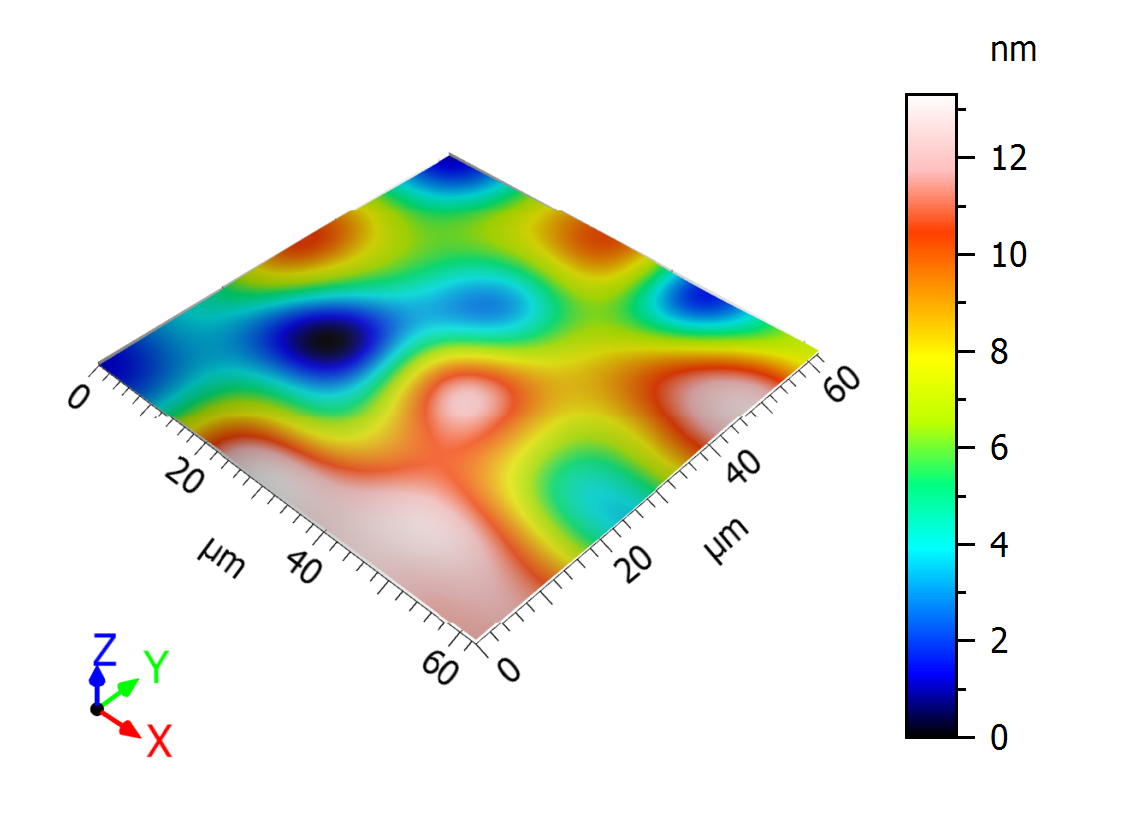

Supplement: Supplementary file 1 [file materials-13-03028-s001.zip › supplementary data/Bandpass filtering/3d_images_filtered_surfaces/μEDMed_Number=15_CentralWavelength=48.png]

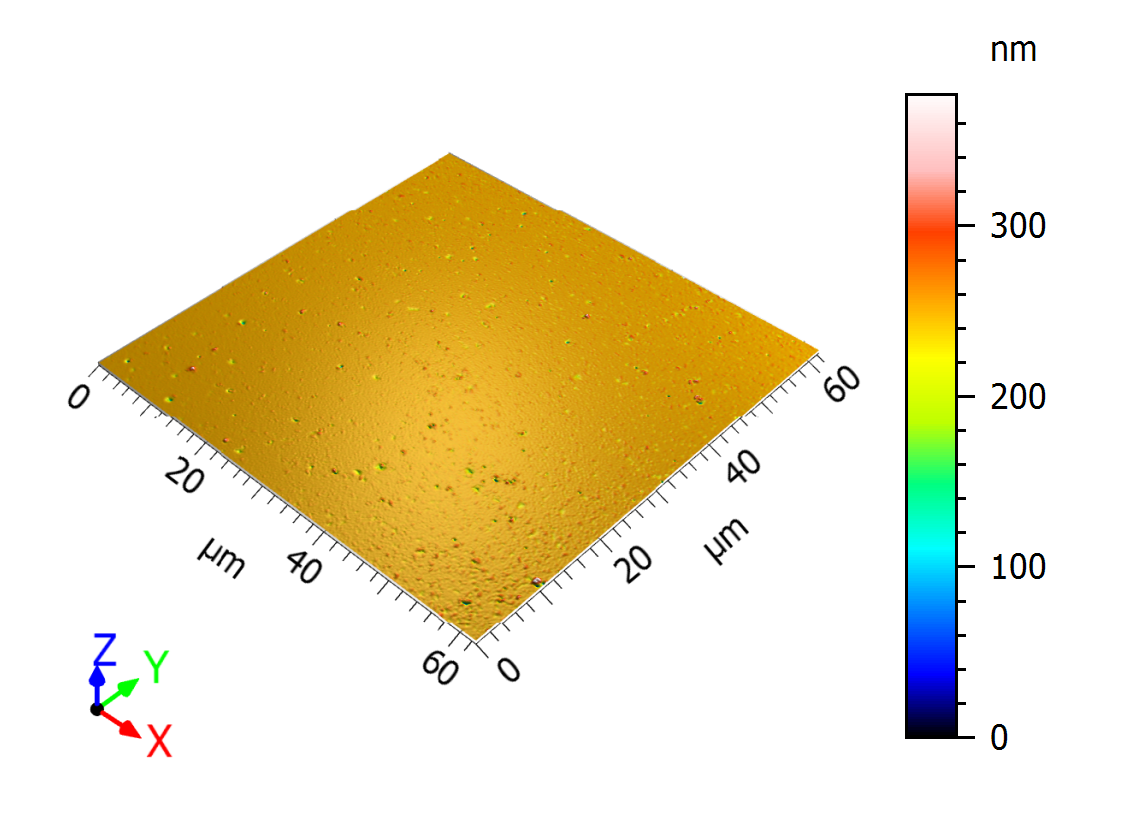

Supplement: Supplementary file 1 [file materials-13-03028-s001.zip › supplementary data/Bandpass filtering/3d_images_filtered_surfaces/μEDMed_Number=1_CentralWavelength=0.421875.png]

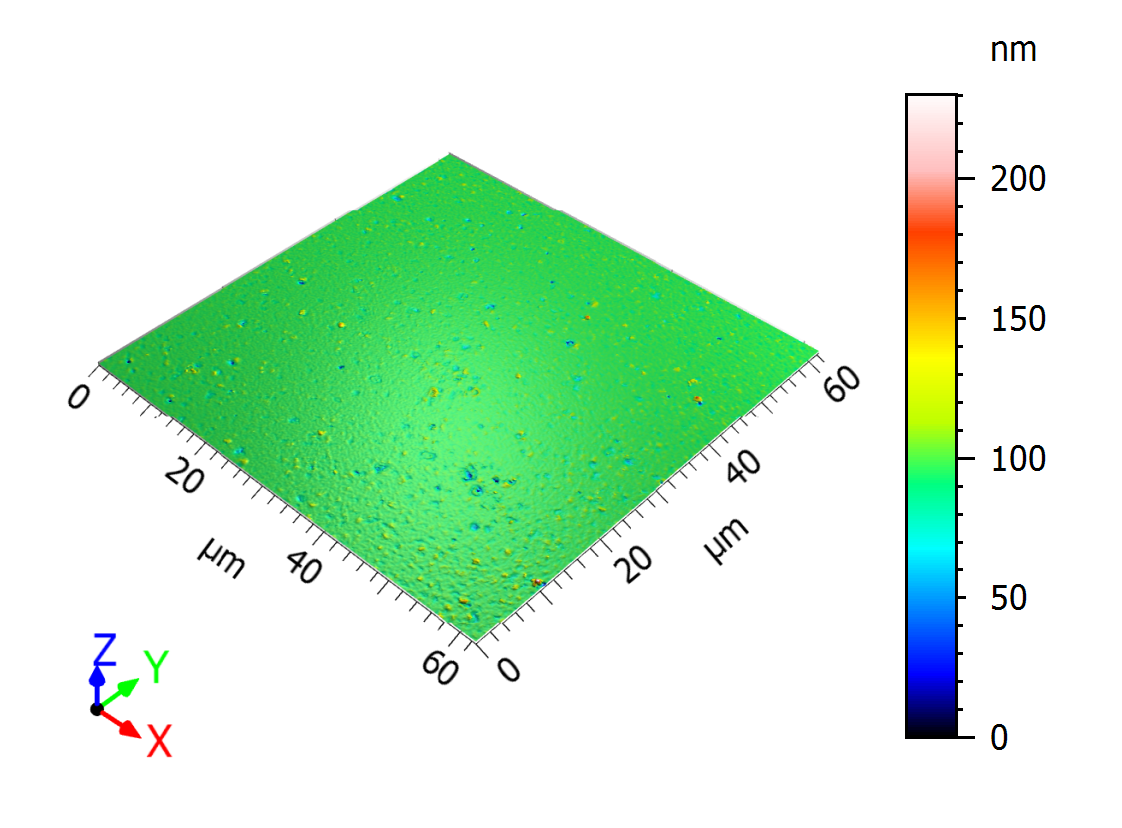

Supplement: Supplementary file 1 [file materials-13-03028-s001.zip › supplementary data/Bandpass filtering/3d_images_filtered_surfaces/μEDMed_Number=2_CentralWavelength=0.5625.png]

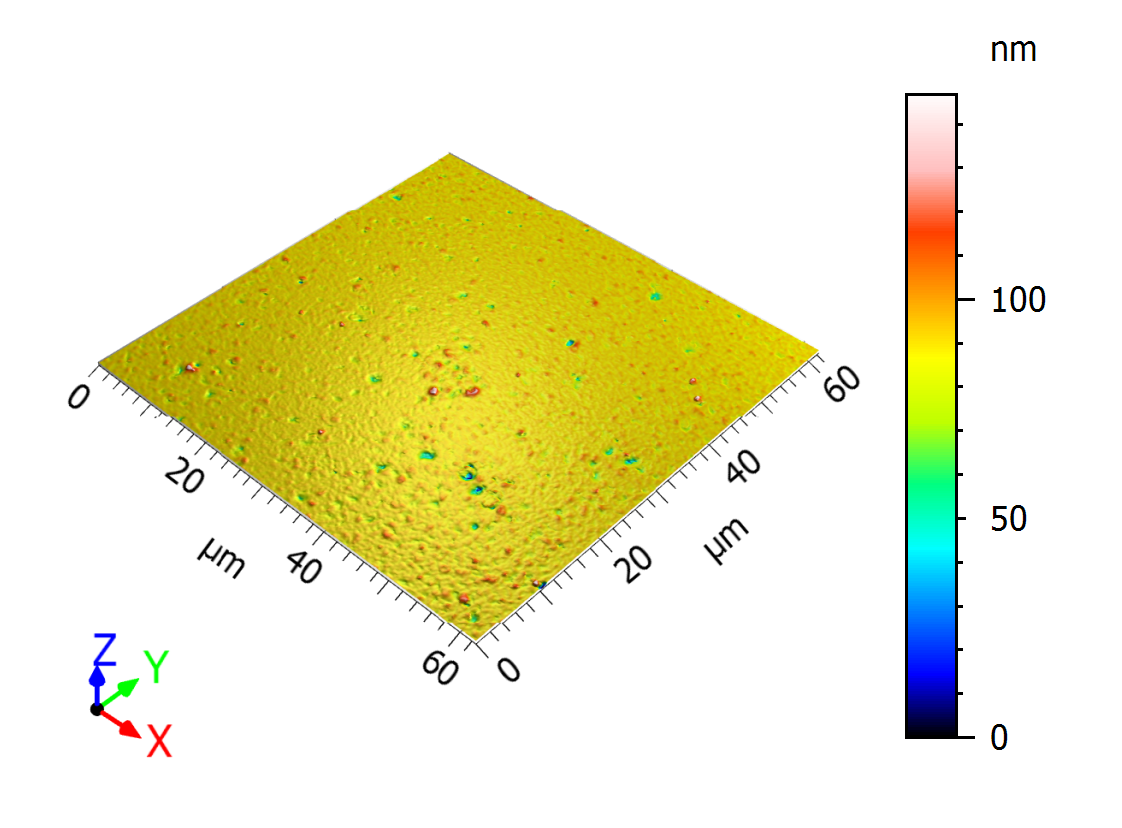

Supplement: Supplementary file 1 [file materials-13-03028-s001.zip › supplementary data/Bandpass filtering/3d_images_filtered_surfaces/μEDMed_Number=3_CentralWavelength=0.84375.png]

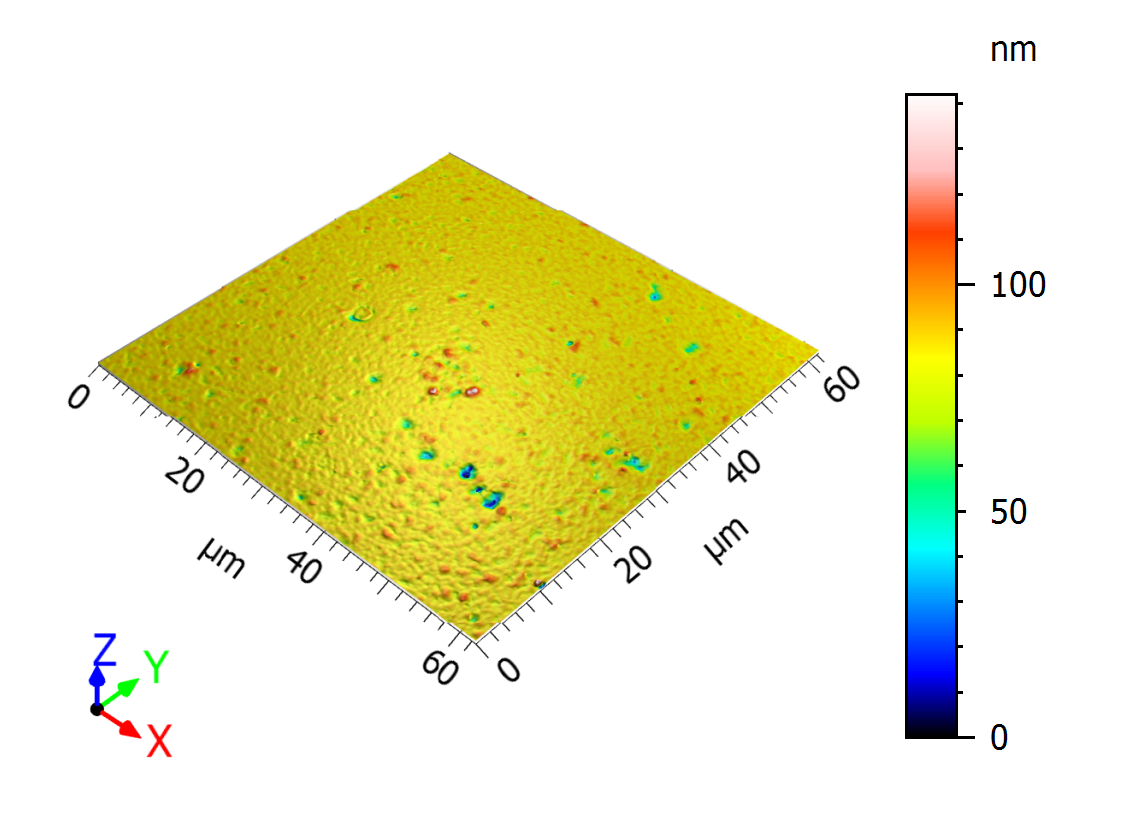

Supplement: Supplementary file 1 [file materials-13-03028-s001.zip › supplementary data/Bandpass filtering/3d_images_filtered_surfaces/μEDMed_Number=4_CentralWavelength=1.125.png]

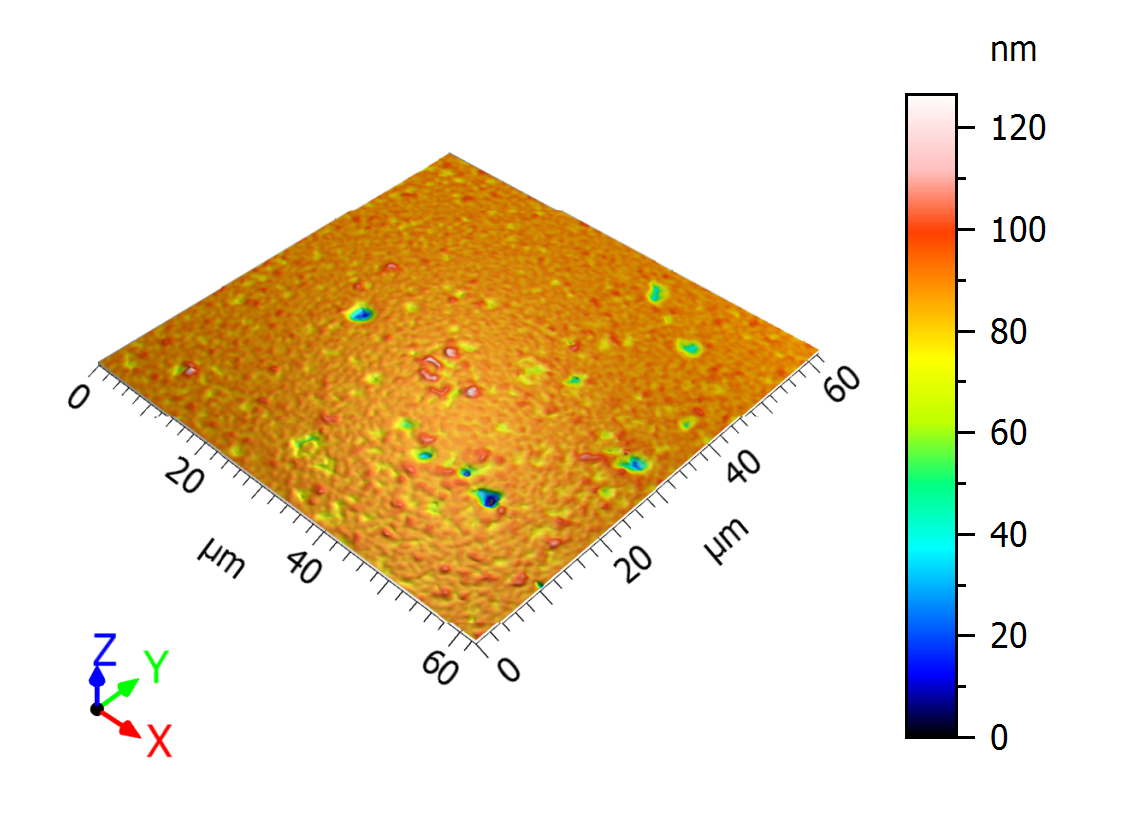

Supplement: Supplementary file 1 [file materials-13-03028-s001.zip › supplementary data/Bandpass filtering/3d_images_filtered_surfaces/μEDMed_Number=5_CentralWavelength=1.6875.png]

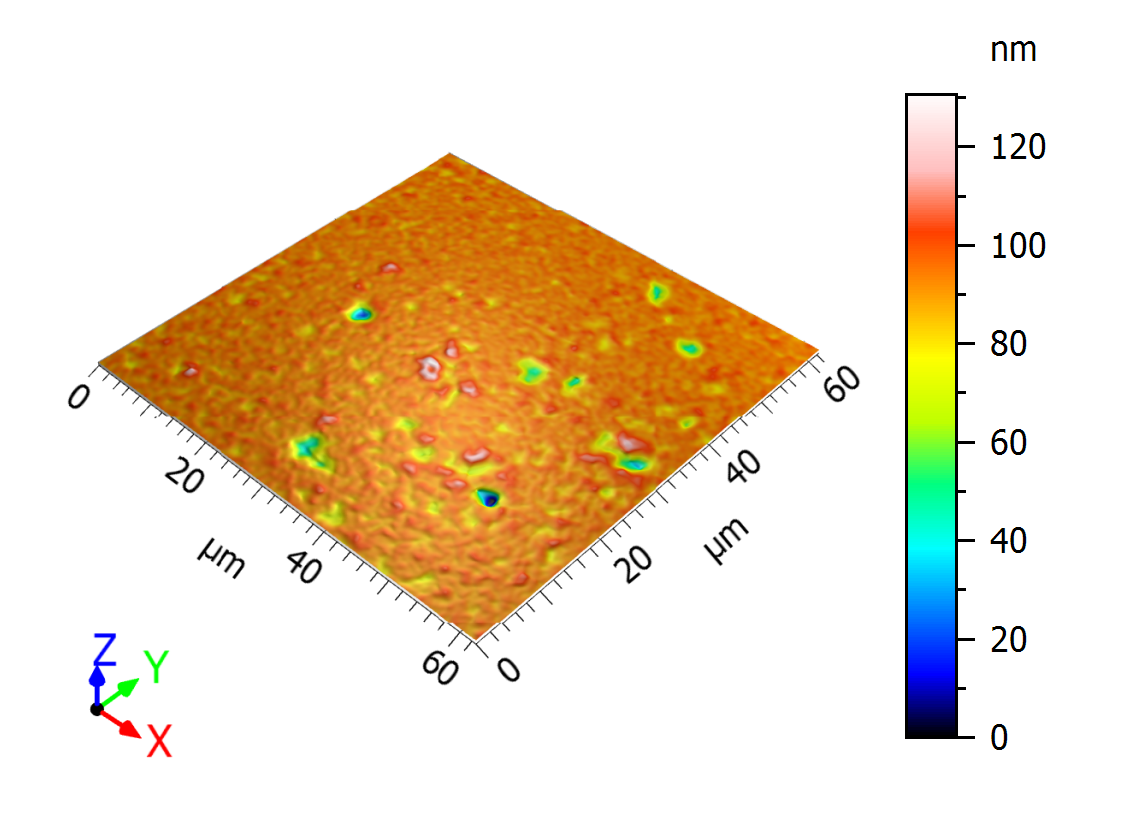

Supplement: Supplementary file 1 [file materials-13-03028-s001.zip › supplementary data/Bandpass filtering/3d_images_filtered_surfaces/μEDMed_Number=6_CentralWavelength=2.25.png]

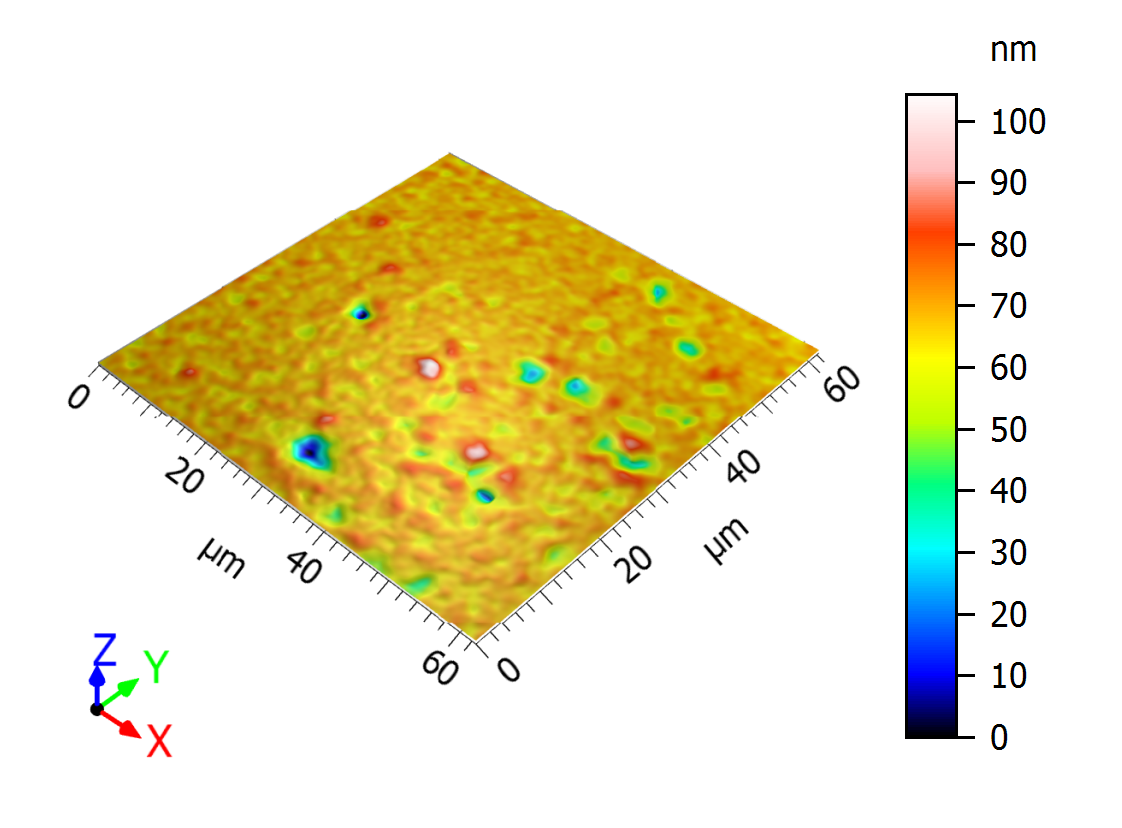

Supplement: Supplementary file 1 [file materials-13-03028-s001.zip › supplementary data/Bandpass filtering/3d_images_filtered_surfaces/μEDMed_Number=7_CentralWavelength=3.375.png]

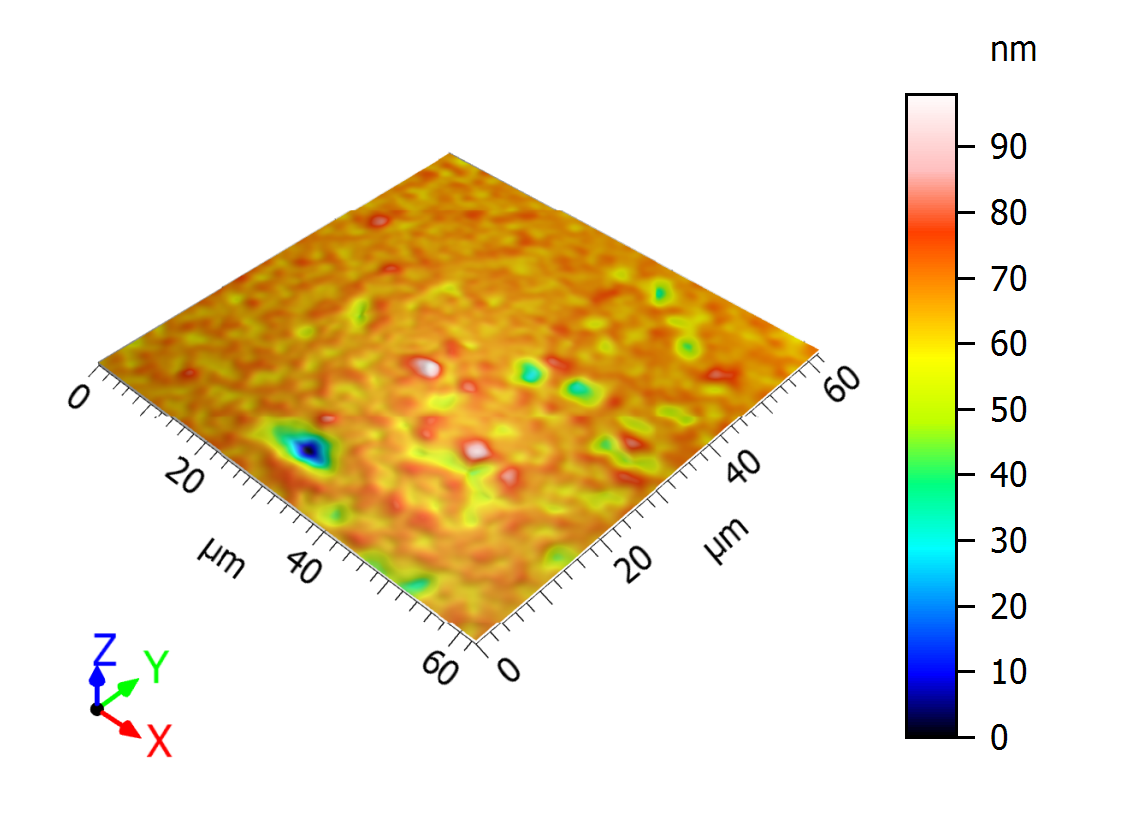

Supplement: Supplementary file 1 [file materials-13-03028-s001.zip › supplementary data/Bandpass filtering/3d_images_filtered_surfaces/μEDMed_Number=8_CentralWavelength=4.5.png]

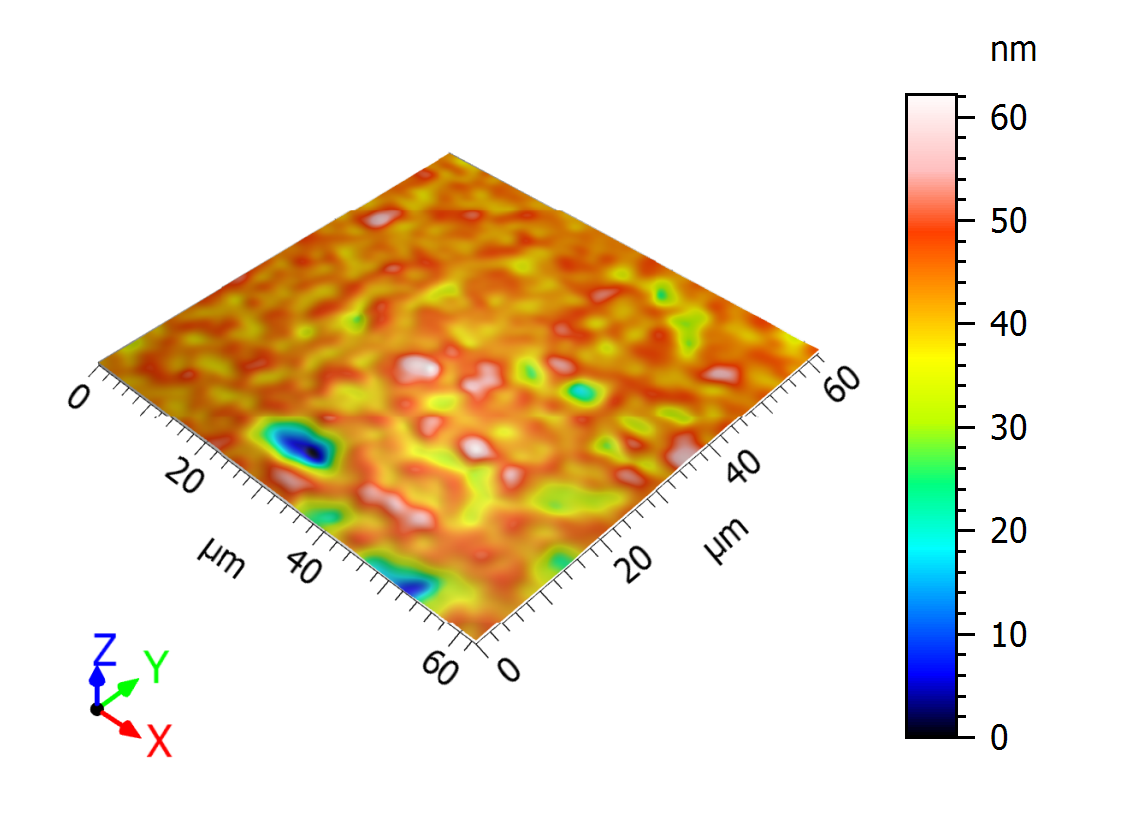

Supplement: Supplementary file 1 [file materials-13-03028-s001.zip › supplementary data/Bandpass filtering/3d_images_filtered_surfaces/μEDMed_Number=9_CentralWavelength=6.75.png]

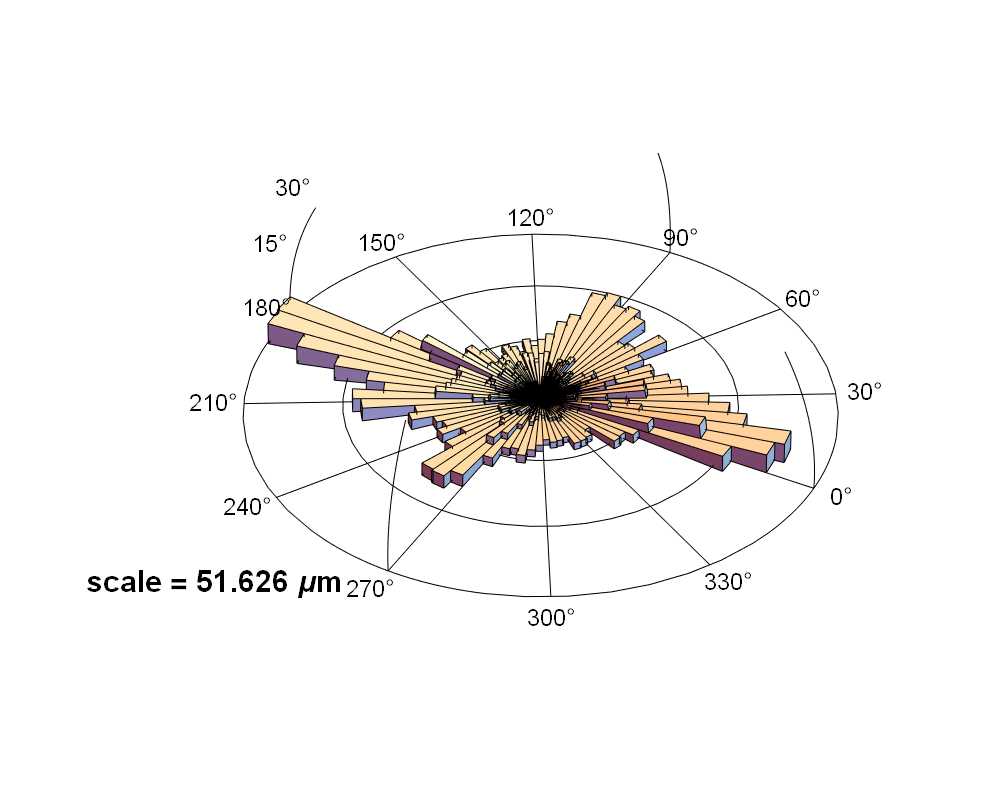

Supplement: Supplementary file 1 [file materials-13-03028-s001.zip › supplementary data/Multiscale curvature analysis/3D rosetta plots/L-PBFed_100withlabel.tiff]

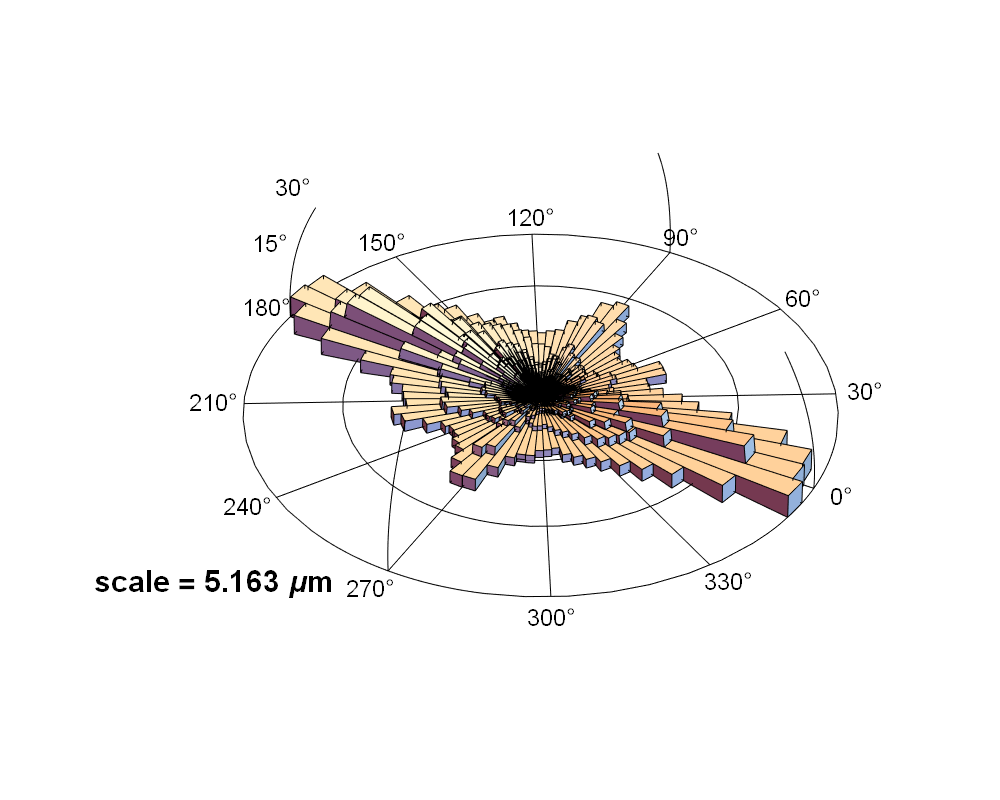

Supplement: Supplementary file 1 [file materials-13-03028-s001.zip › supplementary data/Multiscale curvature analysis/3D rosetta plots/L-PBFed_10withlabel.tiff]

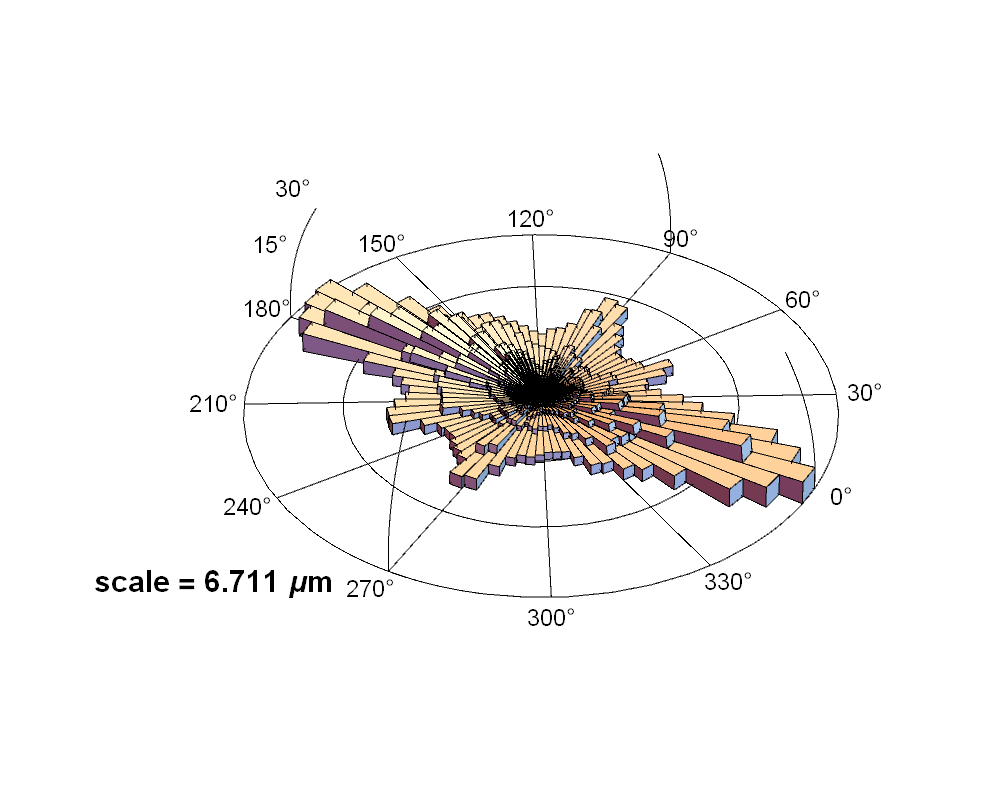

Supplement: Supplementary file 1 [file materials-13-03028-s001.zip › supplementary data/Multiscale curvature analysis/3D rosetta plots/L-PBFed_13withlabel.tiff]

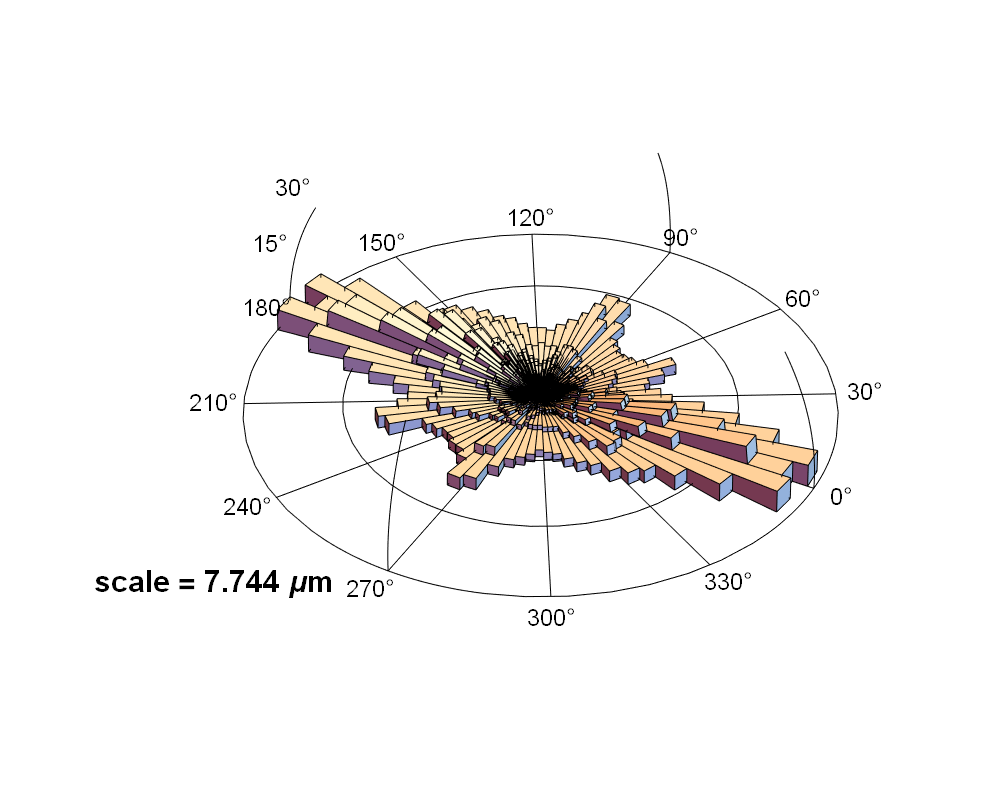

Supplement: Supplementary file 1 [file materials-13-03028-s001.zip › supplementary data/Multiscale curvature analysis/3D rosetta plots/L-PBFed_15withlabel.tiff]

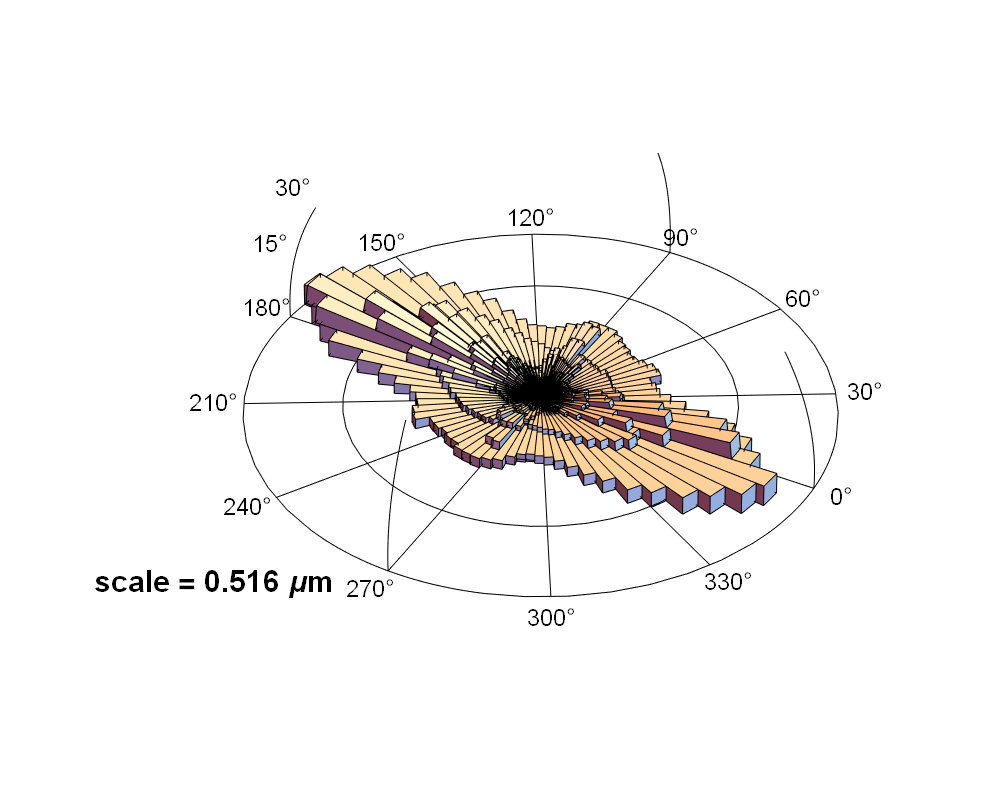

Supplement: Supplementary file 1 [file materials-13-03028-s001.zip › supplementary data/Multiscale curvature analysis/3D rosetta plots/L-PBFed_1withlabel.tiff]

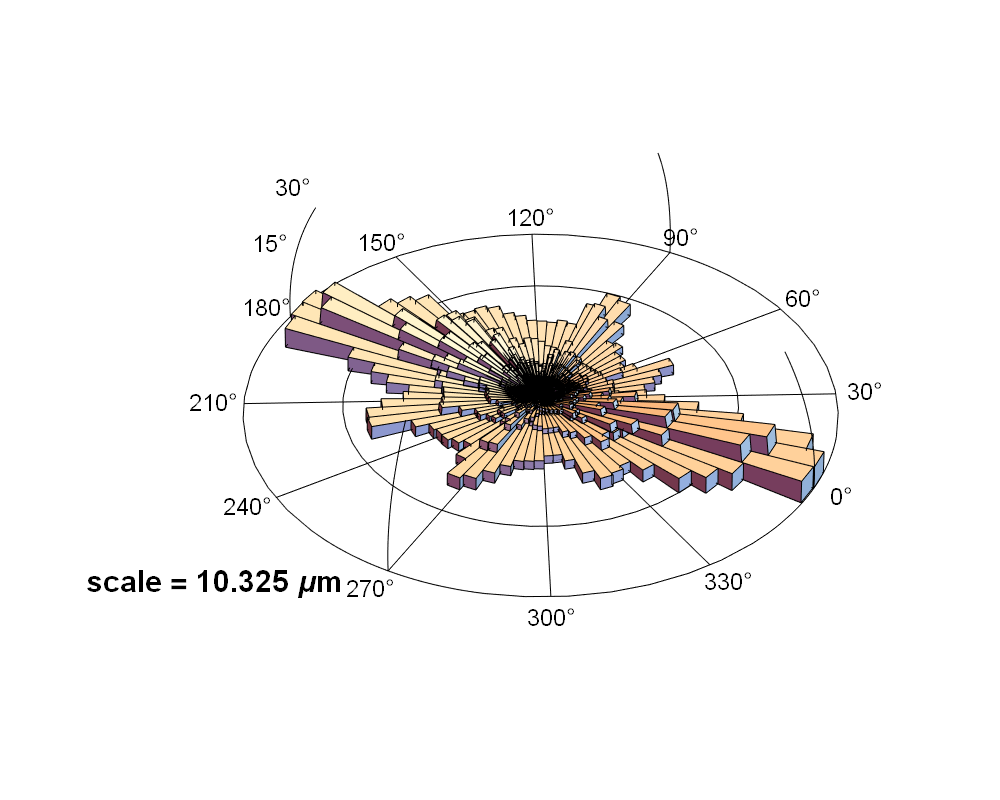

Supplement: Supplementary file 1 [file materials-13-03028-s001.zip › supplementary data/Multiscale curvature analysis/3D rosetta plots/L-PBFed_20withlabel.tiff]

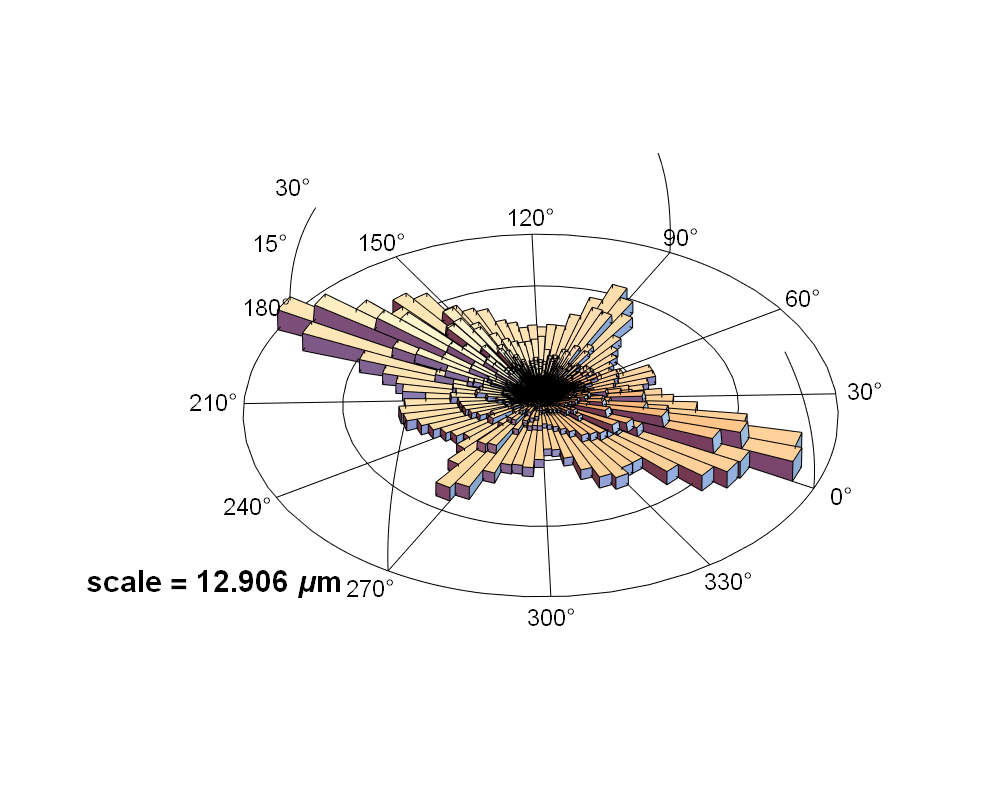

Supplement: Supplementary file 1 [file materials-13-03028-s001.zip › supplementary data/Multiscale curvature analysis/3D rosetta plots/L-PBFed_25withlabel.tiff]

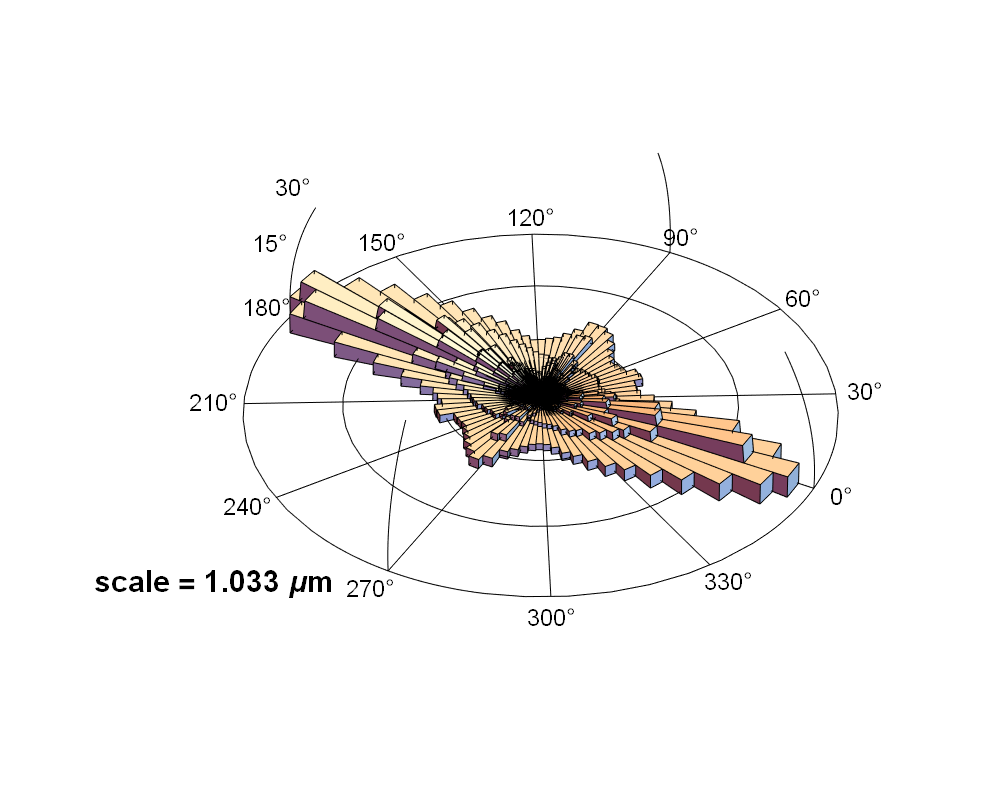

Supplement: Supplementary file 1 [file materials-13-03028-s001.zip › supplementary data/Multiscale curvature analysis/3D rosetta plots/L-PBFed_2withlabel.tiff]

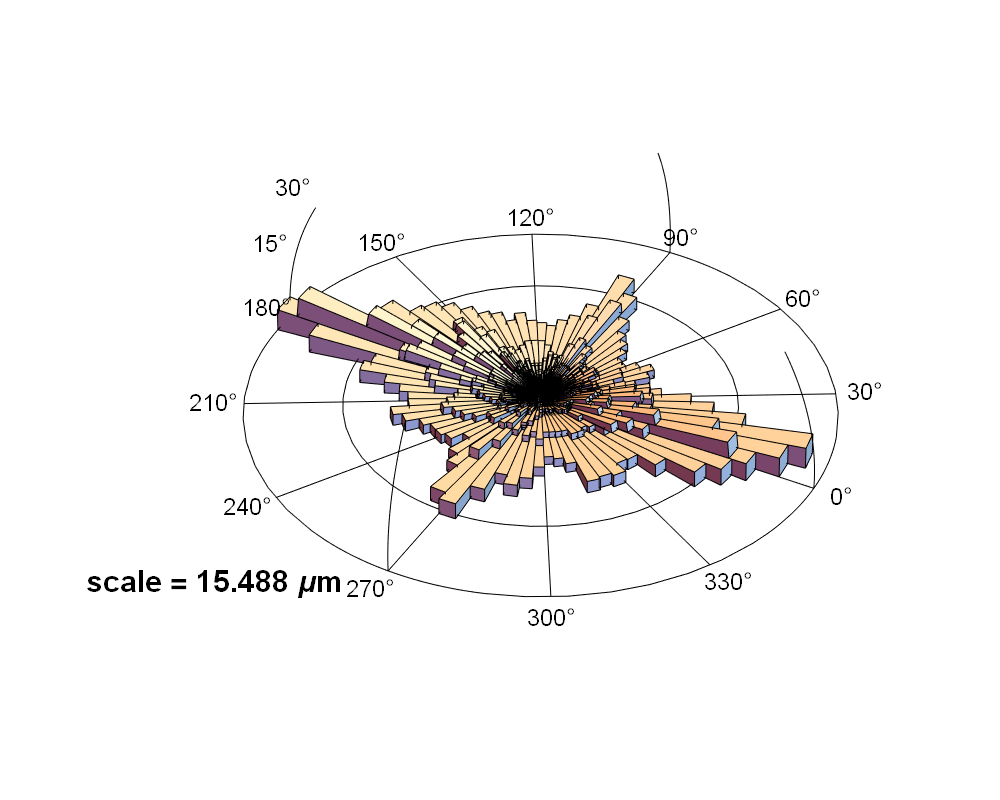

Supplement: Supplementary file 1 [file materials-13-03028-s001.zip › supplementary data/Multiscale curvature analysis/3D rosetta plots/L-PBFed_30withlabel.tiff]

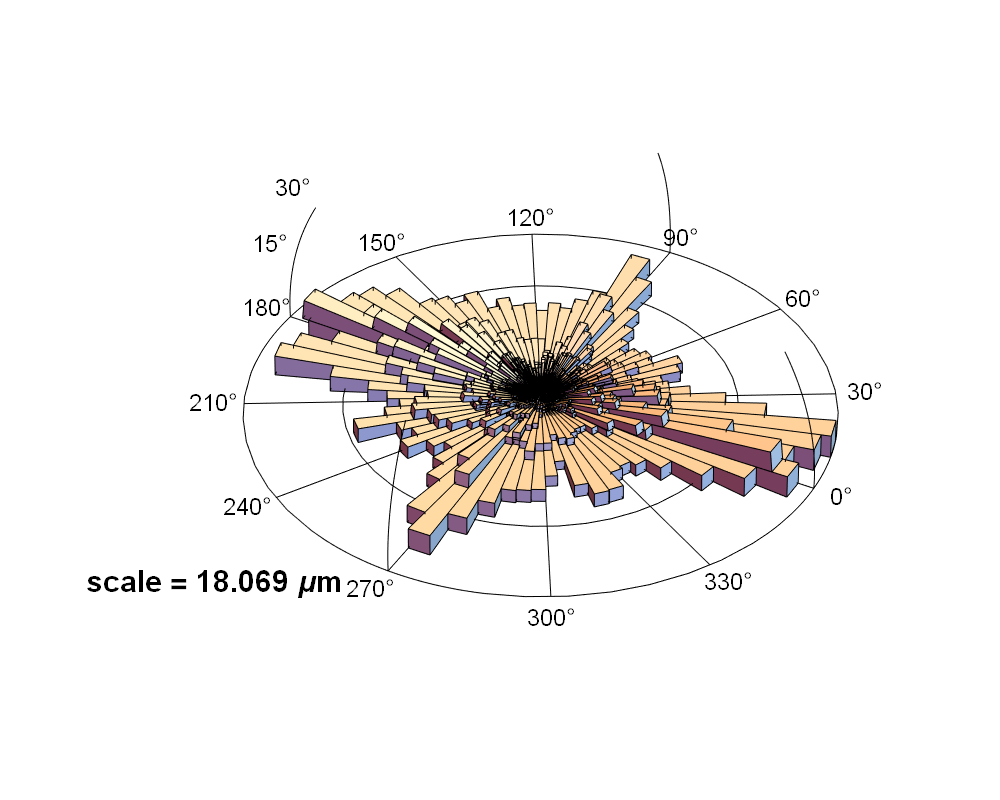

Supplement: Supplementary file 1 [file materials-13-03028-s001.zip › supplementary data/Multiscale curvature analysis/3D rosetta plots/L-PBFed_35withlabel.tiff]

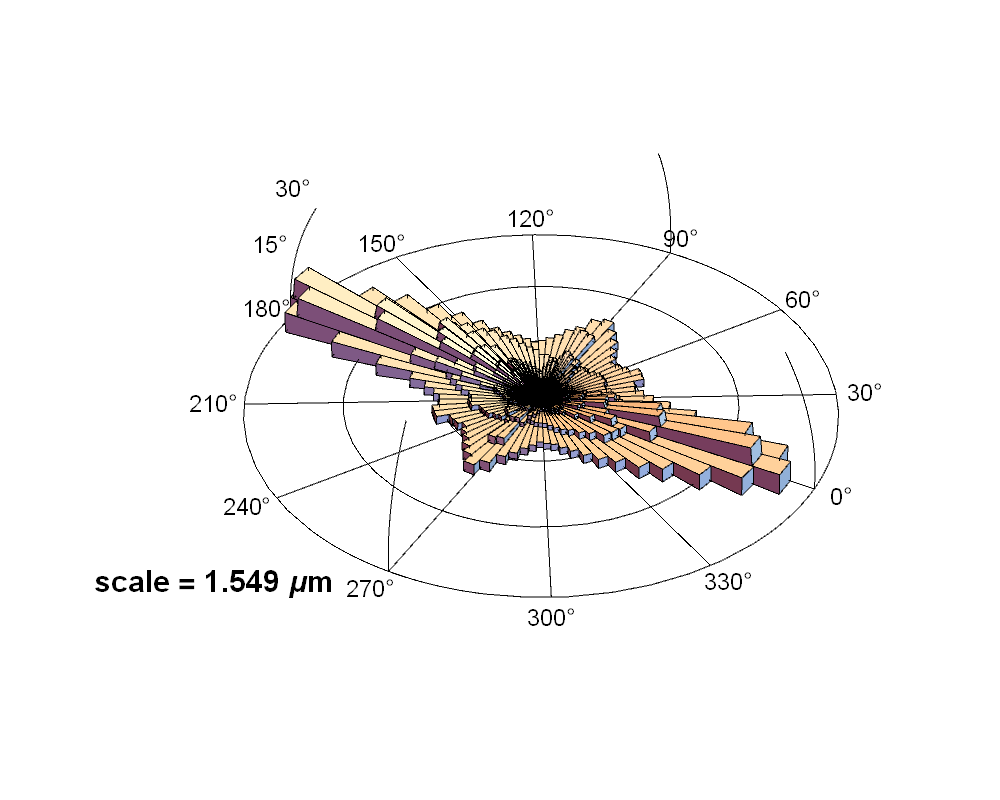

Supplement: Supplementary file 1 [file materials-13-03028-s001.zip › supplementary data/Multiscale curvature analysis/3D rosetta plots/L-PBFed_3withlabel.tiff]

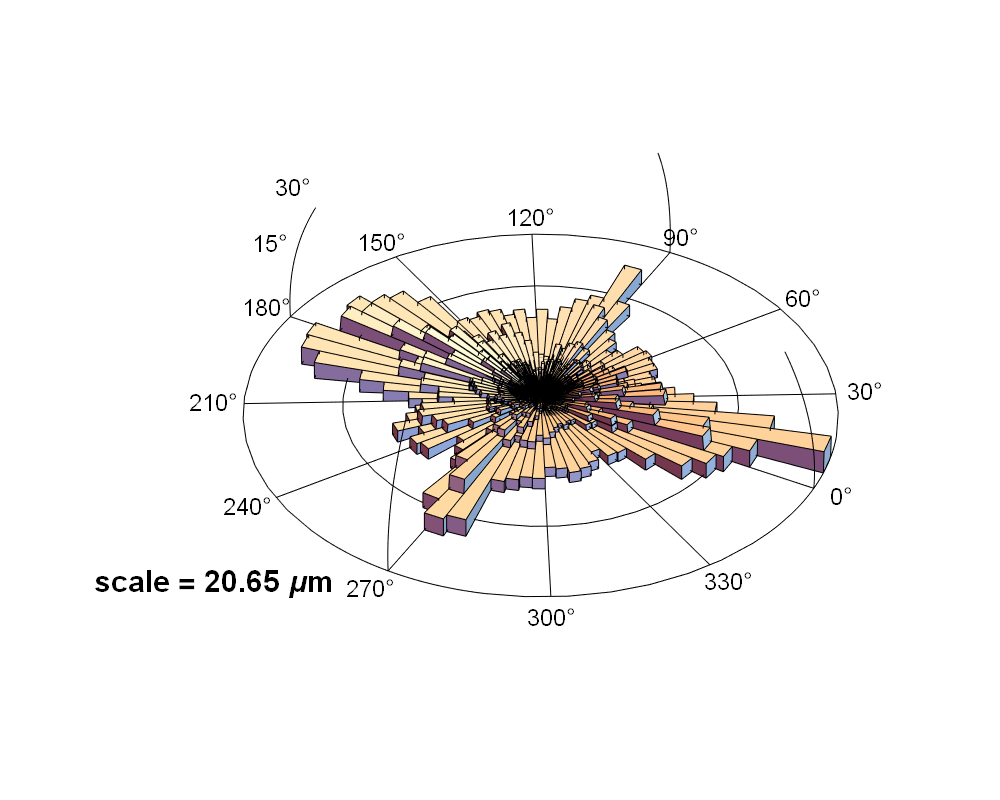

Supplement: Supplementary file 1 [file materials-13-03028-s001.zip › supplementary data/Multiscale curvature analysis/3D rosetta plots/L-PBFed_40withlabel.tiff]

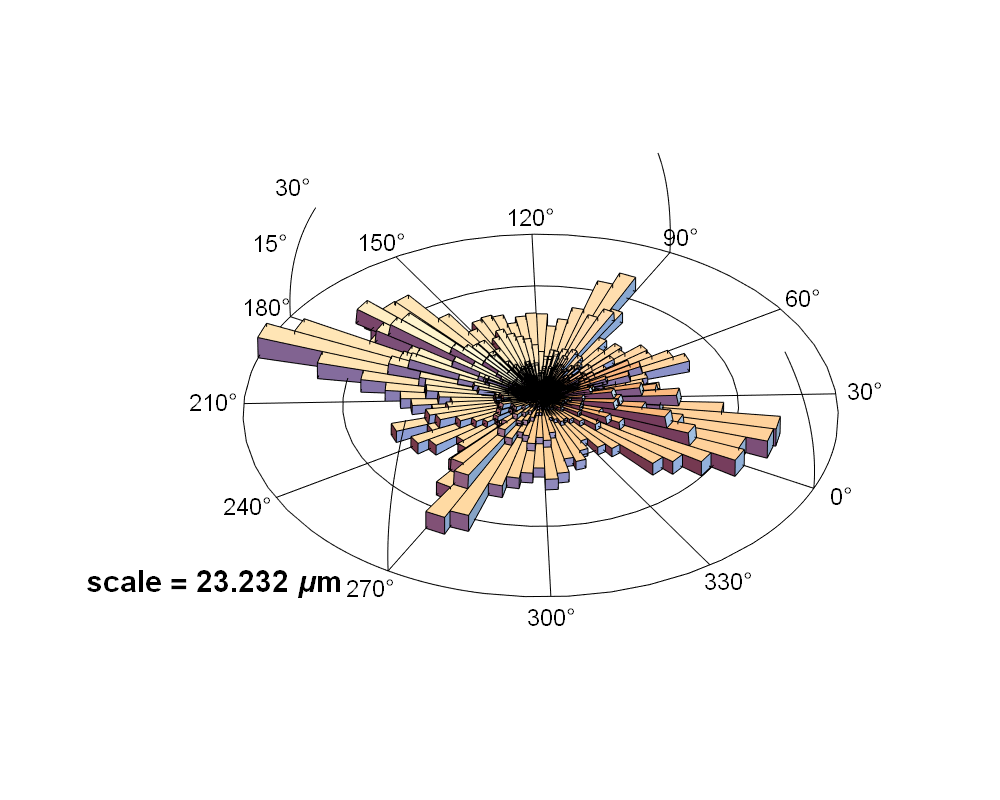

Supplement: Supplementary file 1 [file materials-13-03028-s001.zip › supplementary data/Multiscale curvature analysis/3D rosetta plots/L-PBFed_45withlabel.tiff]

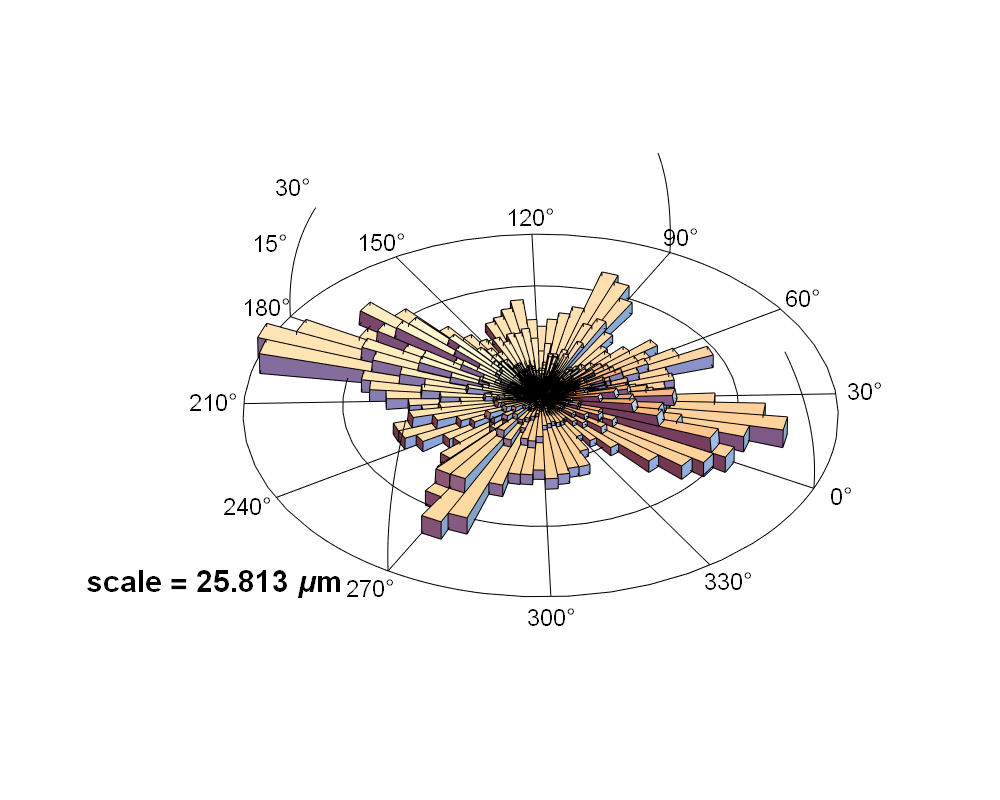

Supplement: Supplementary file 1 [file materials-13-03028-s001.zip › supplementary data/Multiscale curvature analysis/3D rosetta plots/L-PBFed_50withlabel.tiff]

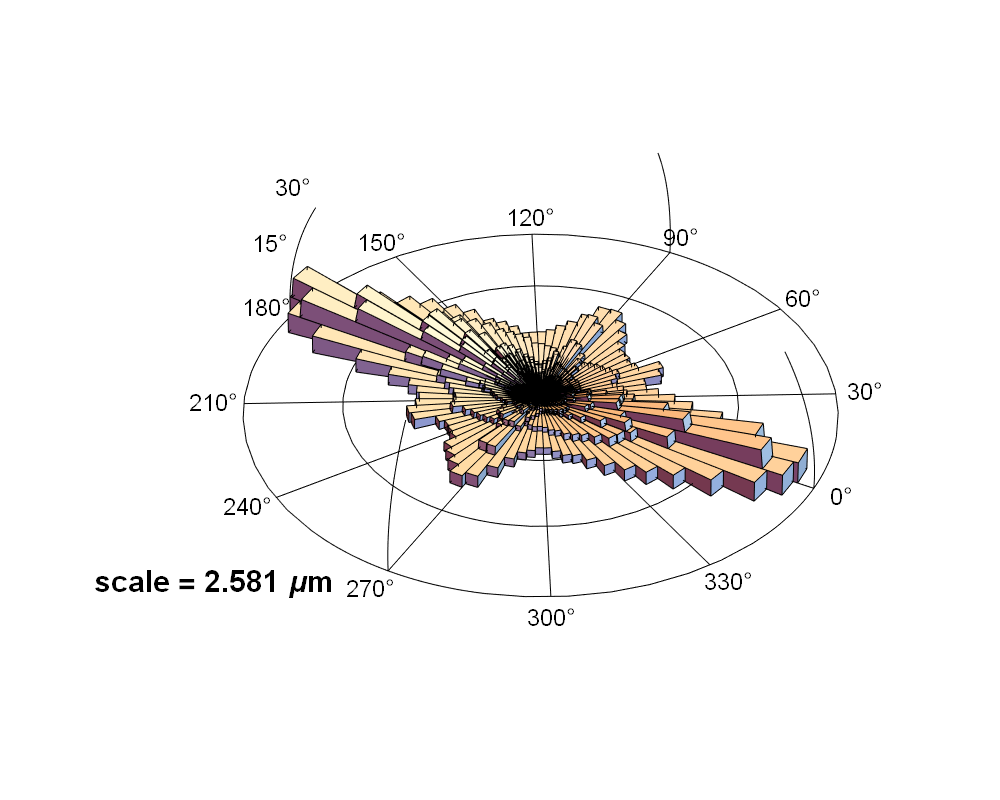

Supplement: Supplementary file 1 [file materials-13-03028-s001.zip › supplementary data/Multiscale curvature analysis/3D rosetta plots/L-PBFed_5withlabel.tiff]

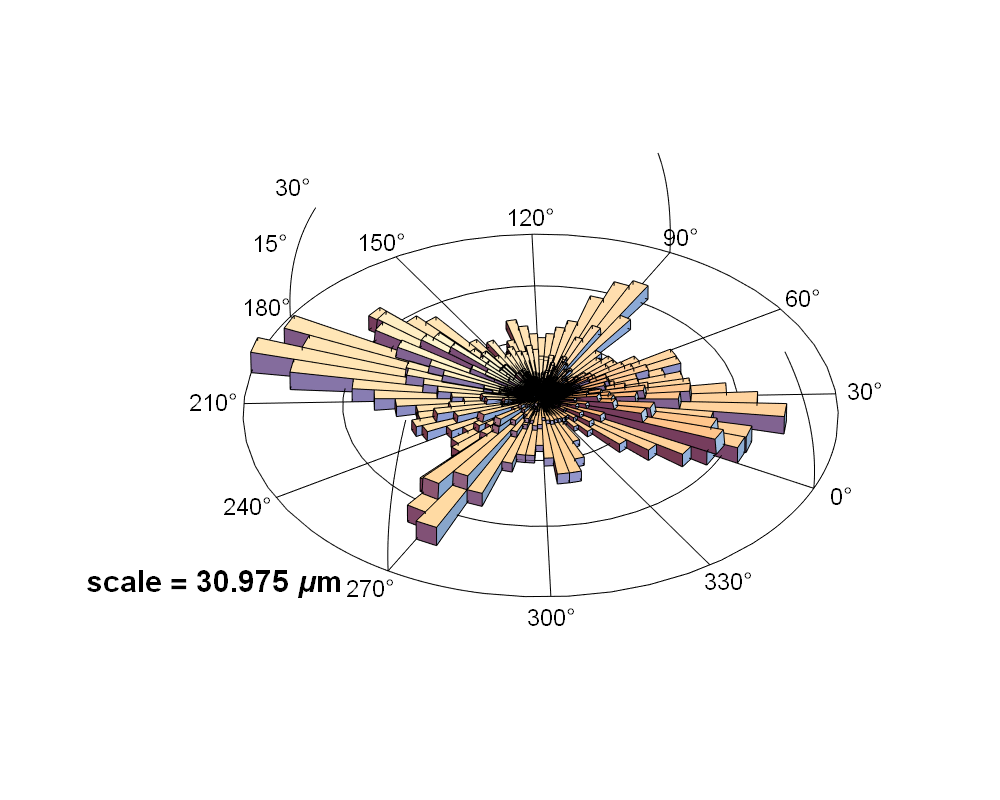

Supplement: Supplementary file 1 [file materials-13-03028-s001.zip › supplementary data/Multiscale curvature analysis/3D rosetta plots/L-PBFed_60withlabel.tiff]

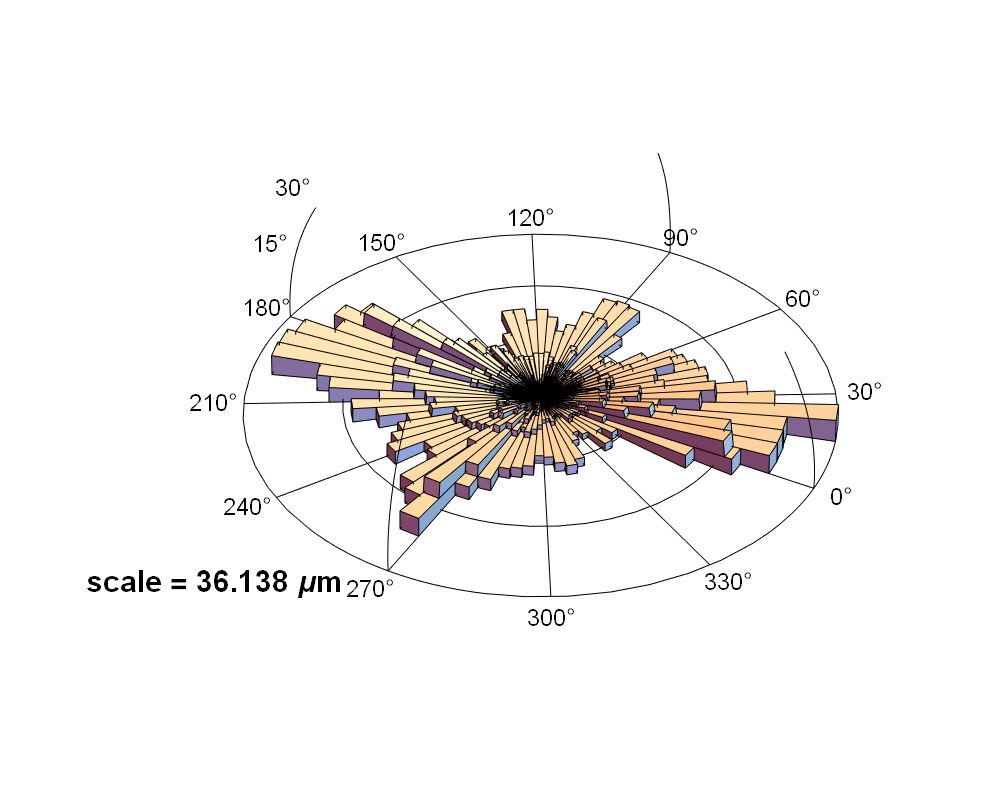

Supplement: Supplementary file 1 [file materials-13-03028-s001.zip › supplementary data/Multiscale curvature analysis/3D rosetta plots/L-PBFed_70withlabel.tiff]

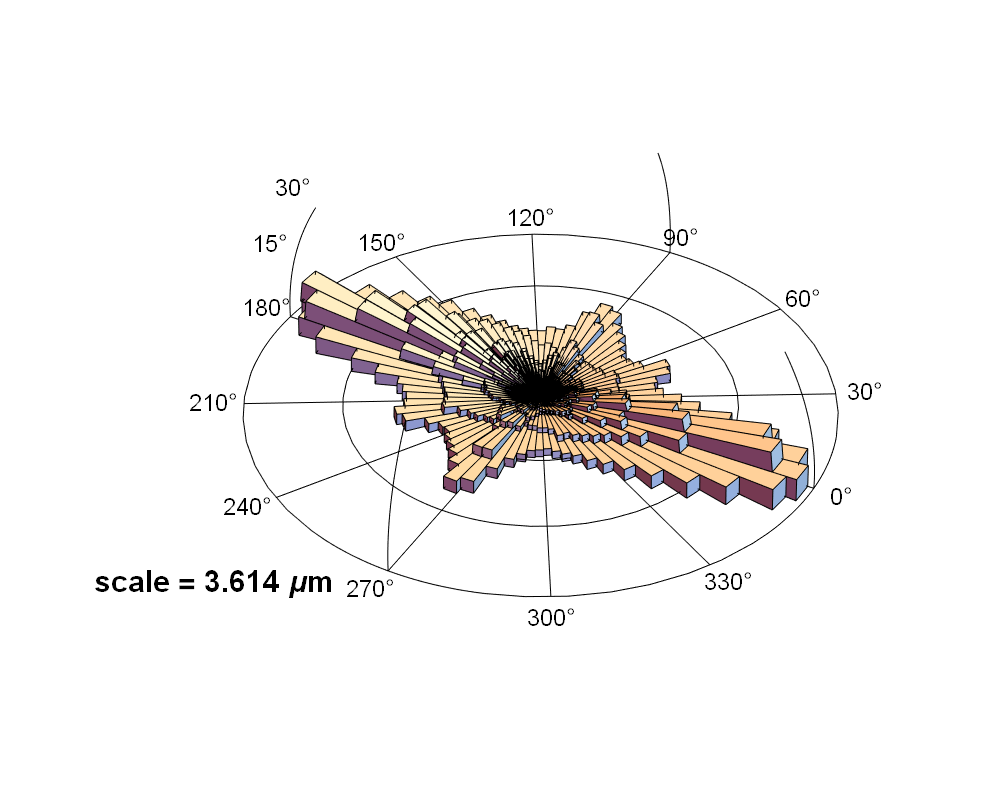

Supplement: Supplementary file 1 [file materials-13-03028-s001.zip › supplementary data/Multiscale curvature analysis/3D rosetta plots/L-PBFed_7withlabel.tiff]

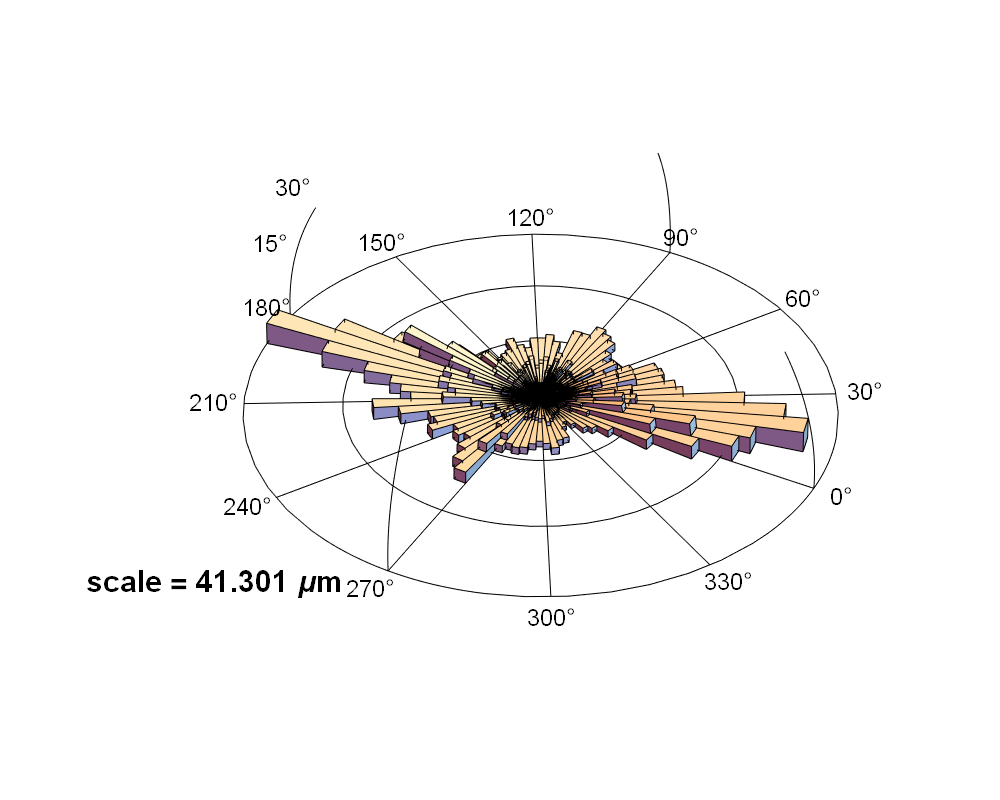

Supplement: Supplementary file 1 [file materials-13-03028-s001.zip › supplementary data/Multiscale curvature analysis/3D rosetta plots/L-PBFed_80withlabel.tiff]

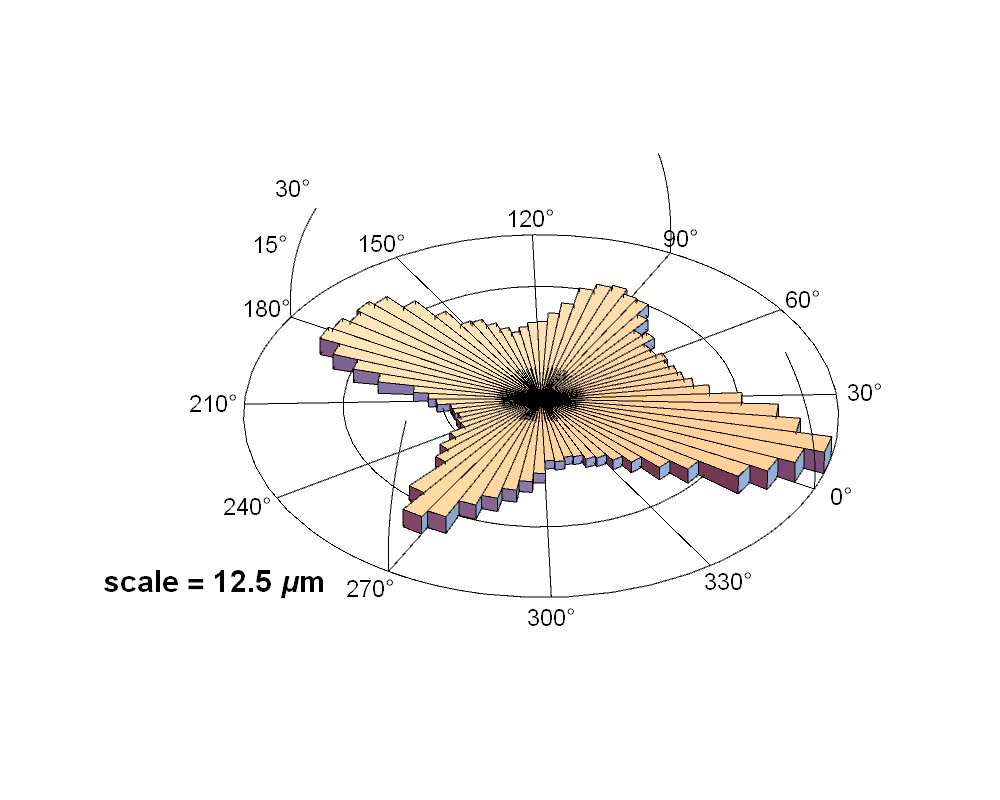

Supplement: Supplementary file 1 [file materials-13-03028-s001.zip › supplementary data/Multiscale curvature analysis/3D rosetta plots/microEDMed_100withlabel.tiff]

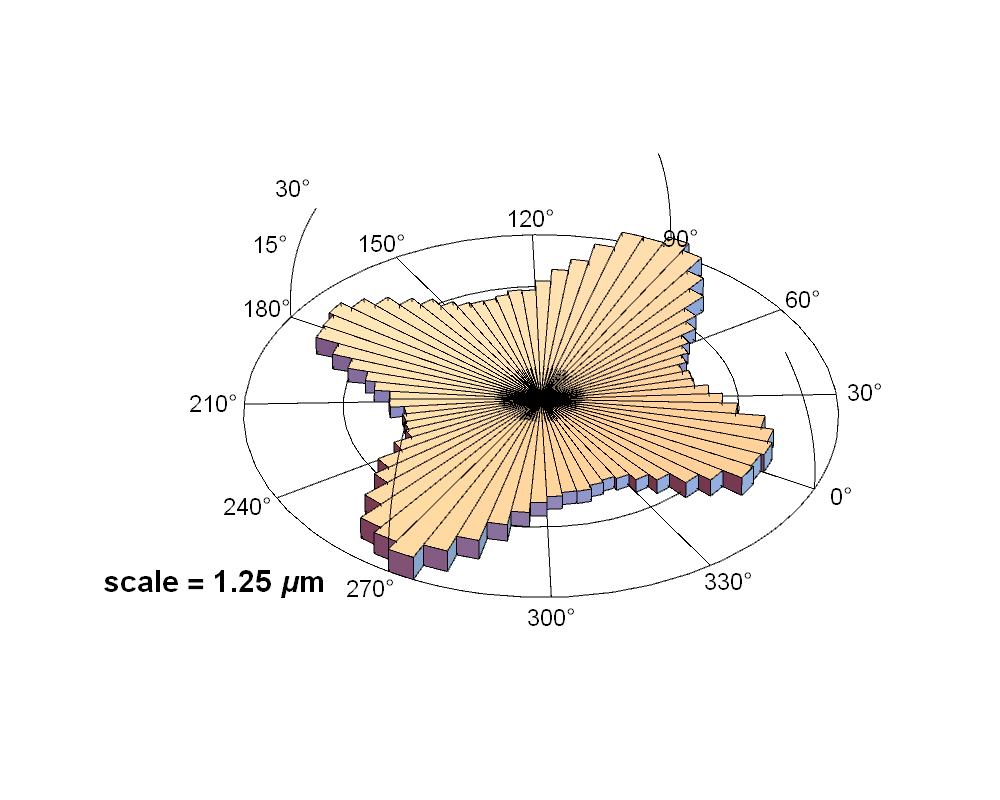

Supplement: Supplementary file 1 [file materials-13-03028-s001.zip › supplementary data/Multiscale curvature analysis/3D rosetta plots/microEDMed_10withlabel.tiff]

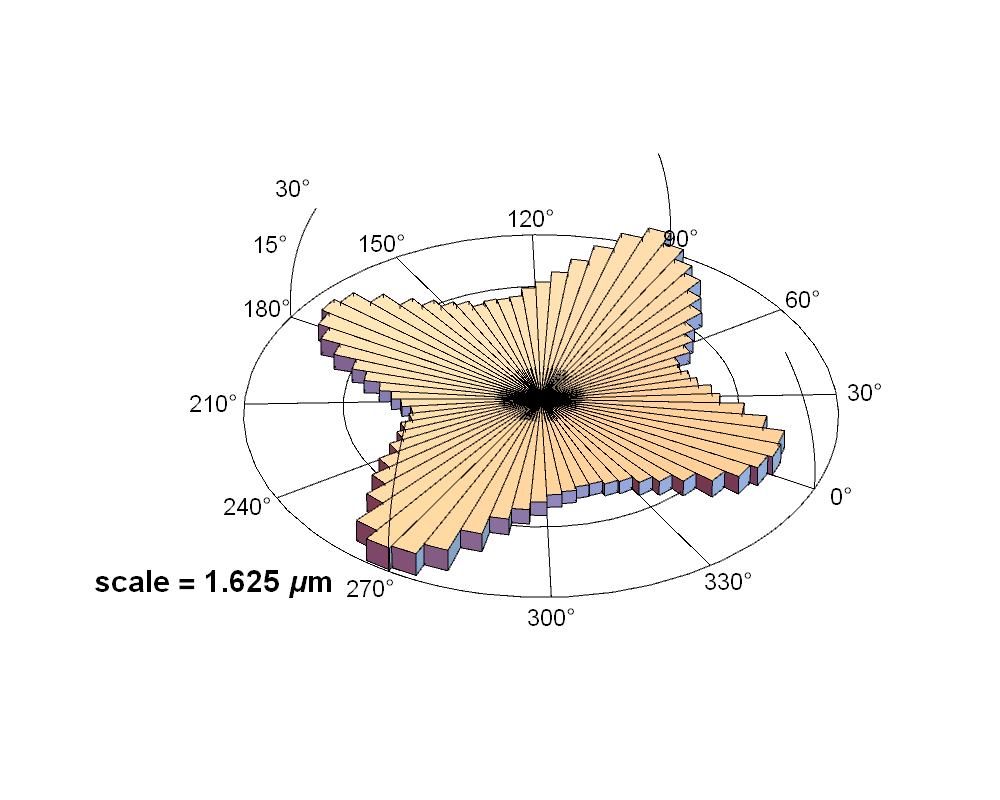

Supplement: Supplementary file 1 [file materials-13-03028-s001.zip › supplementary data/Multiscale curvature analysis/3D rosetta plots/microEDMed_13withlabel.tiff]

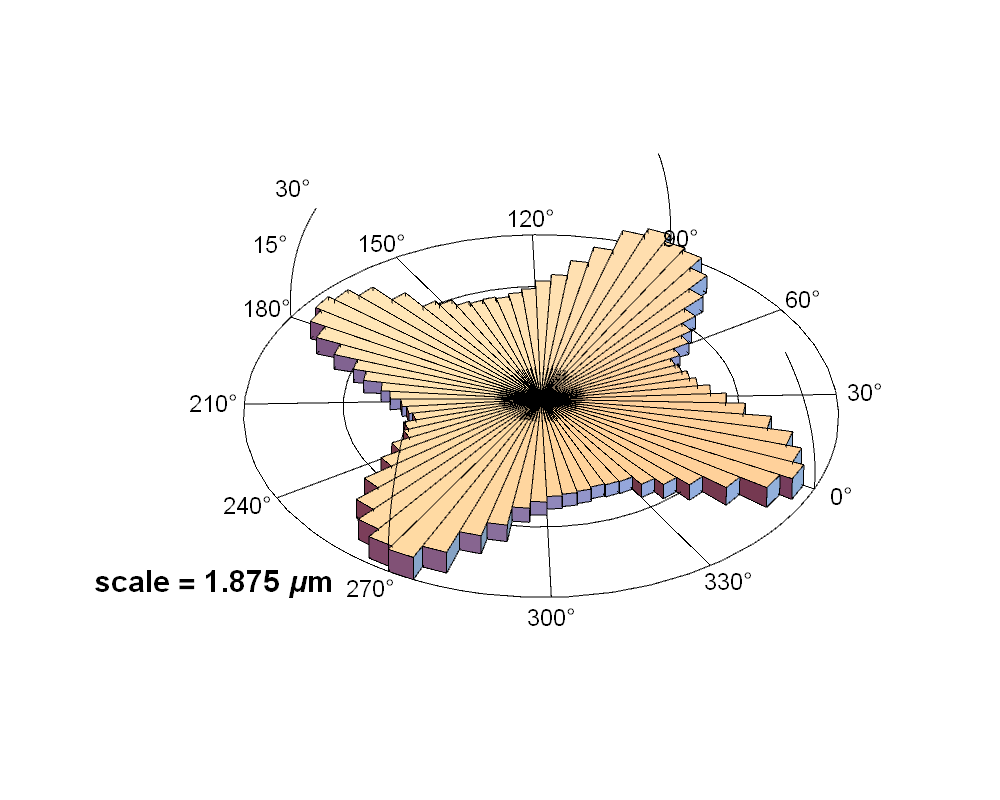

Supplement: Supplementary file 1 [file materials-13-03028-s001.zip › supplementary data/Multiscale curvature analysis/3D rosetta plots/microEDMed_15withlabel.tiff]

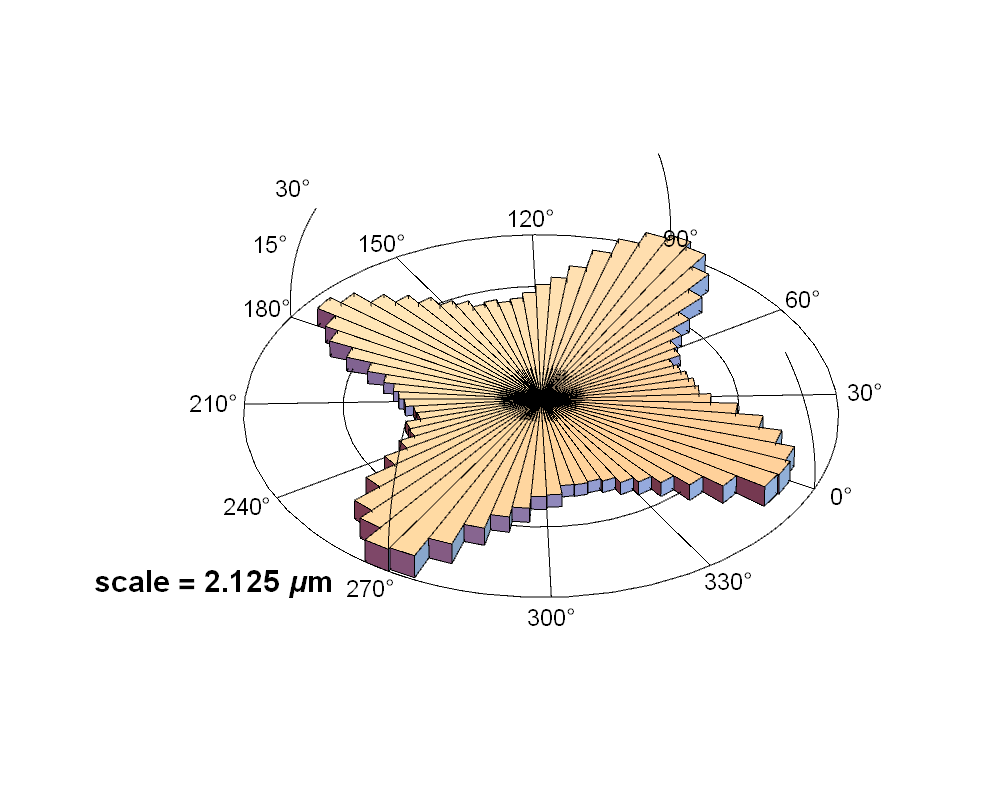

Supplement: Supplementary file 1 [file materials-13-03028-s001.zip › supplementary data/Multiscale curvature analysis/3D rosetta plots/microEDMed_17withlabel.tiff]

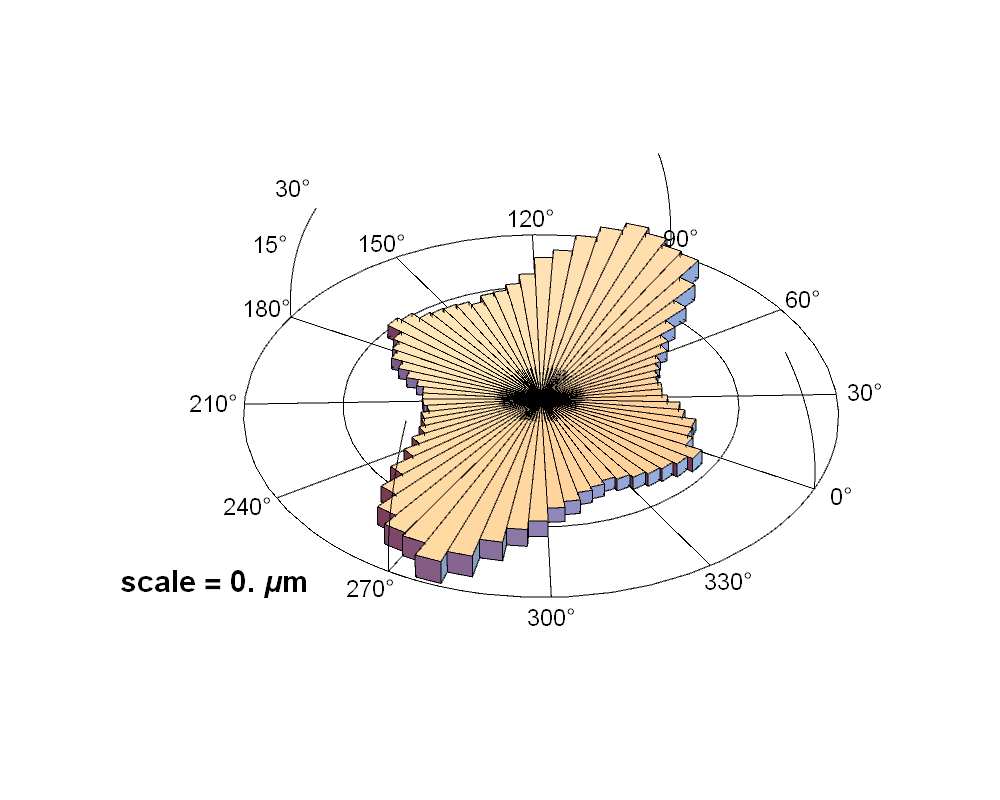

Supplement: Supplementary file 1 [file materials-13-03028-s001.zip › supplementary data/Multiscale curvature analysis/3D rosetta plots/microEDMed_1withlabel.tiff]

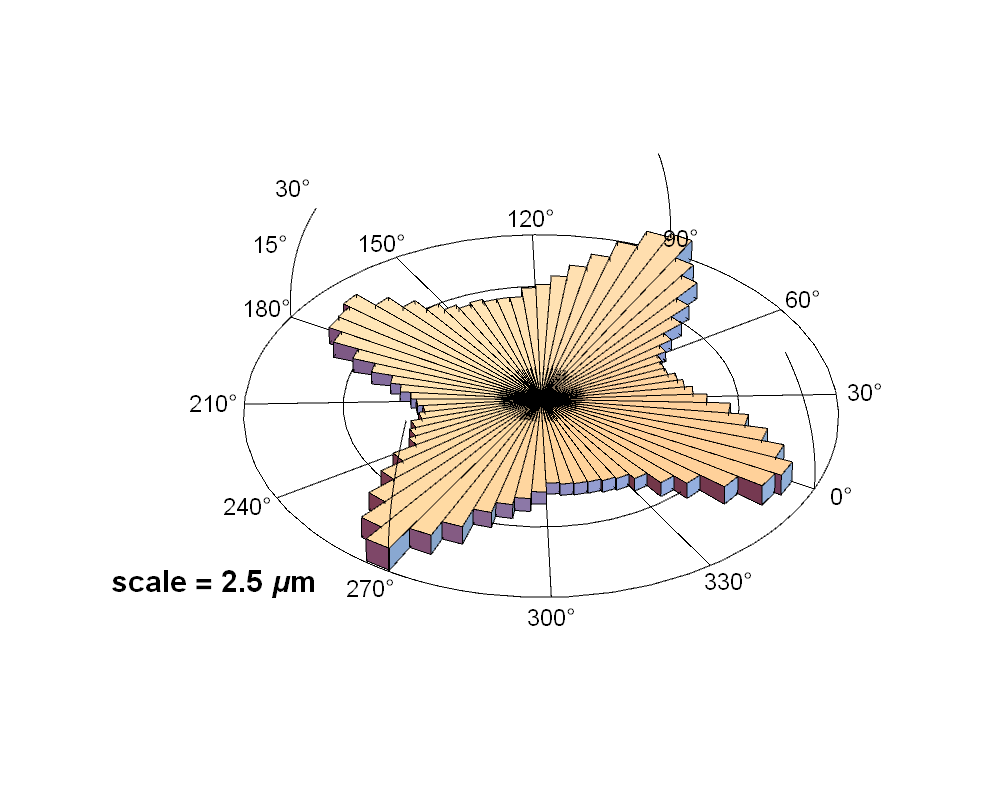

Supplement: Supplementary file 1 [file materials-13-03028-s001.zip › supplementary data/Multiscale curvature analysis/3D rosetta plots/microEDMed_20withlabel.tiff]

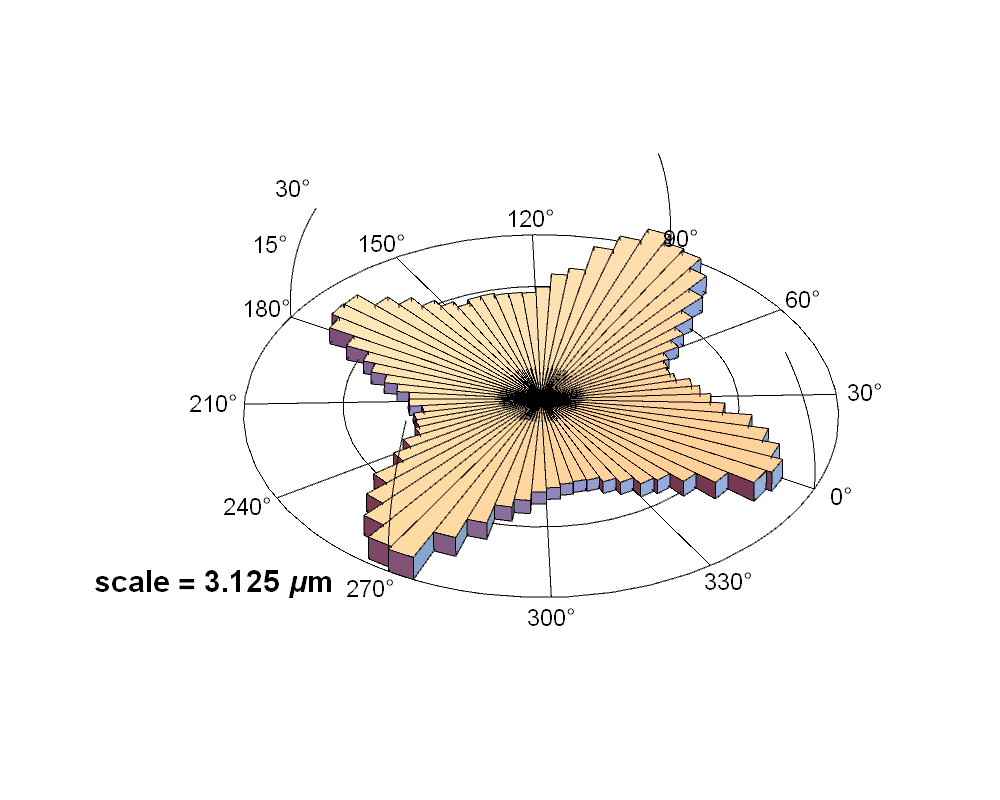

Supplement: Supplementary file 1 [file materials-13-03028-s001.zip › supplementary data/Multiscale curvature analysis/3D rosetta plots/microEDMed_25withlabel.tiff]

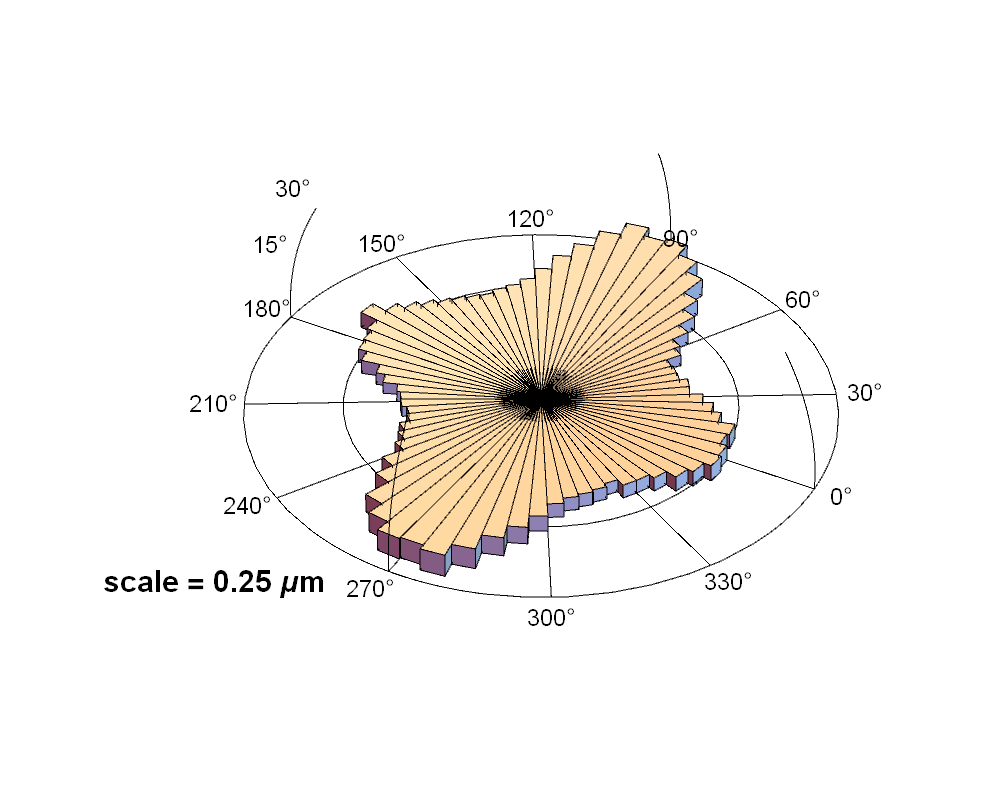

Supplement: Supplementary file 1 [file materials-13-03028-s001.zip › supplementary data/Multiscale curvature analysis/3D rosetta plots/microEDMed_2withlabel.tiff]

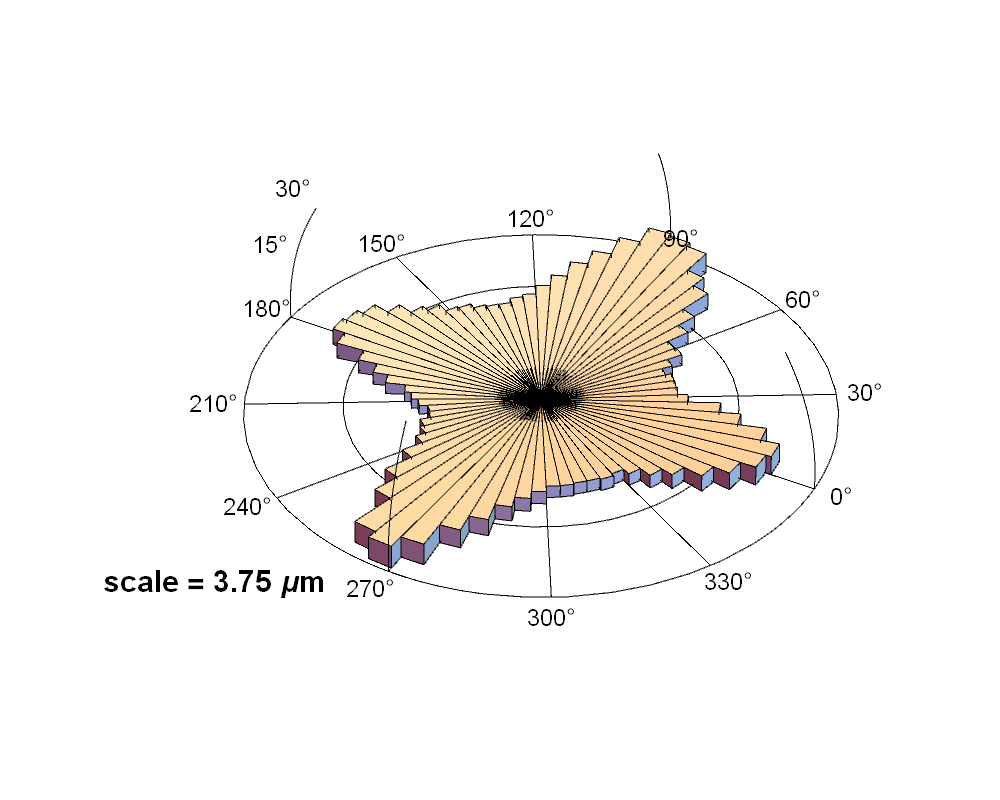

Supplement: Supplementary file 1 [file materials-13-03028-s001.zip › supplementary data/Multiscale curvature analysis/3D rosetta plots/microEDMed_30withlabel.tiff]

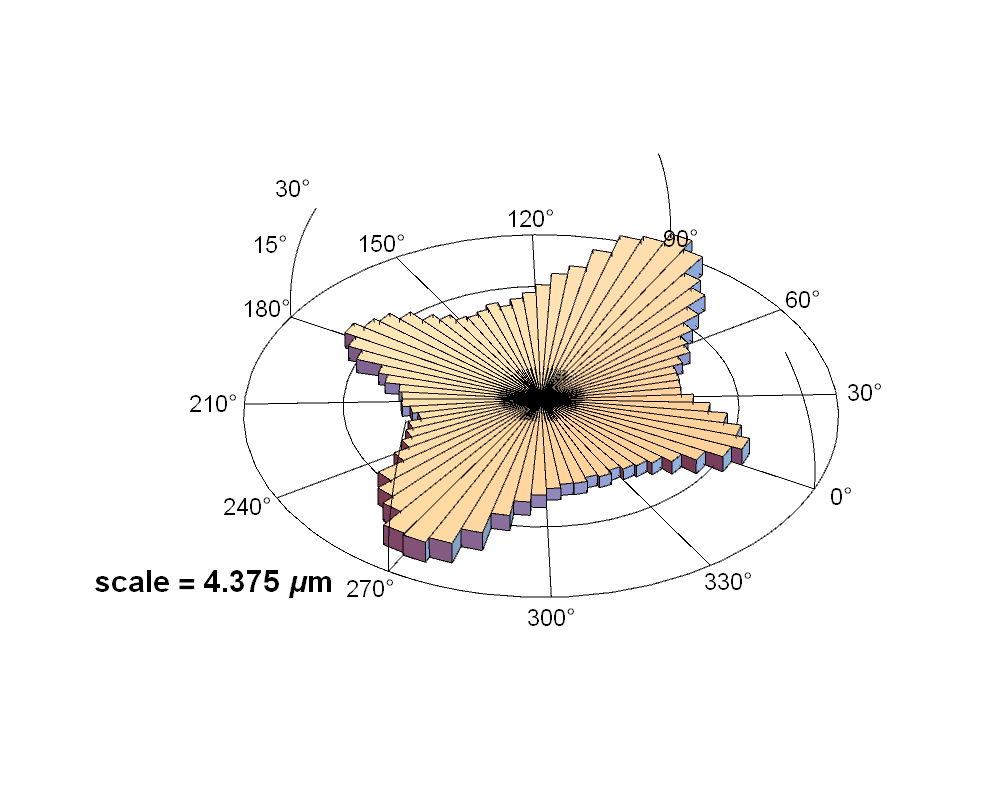

Supplement: Supplementary file 1 [file materials-13-03028-s001.zip › supplementary data/Multiscale curvature analysis/3D rosetta plots/microEDMed_35withlabel.tiff]

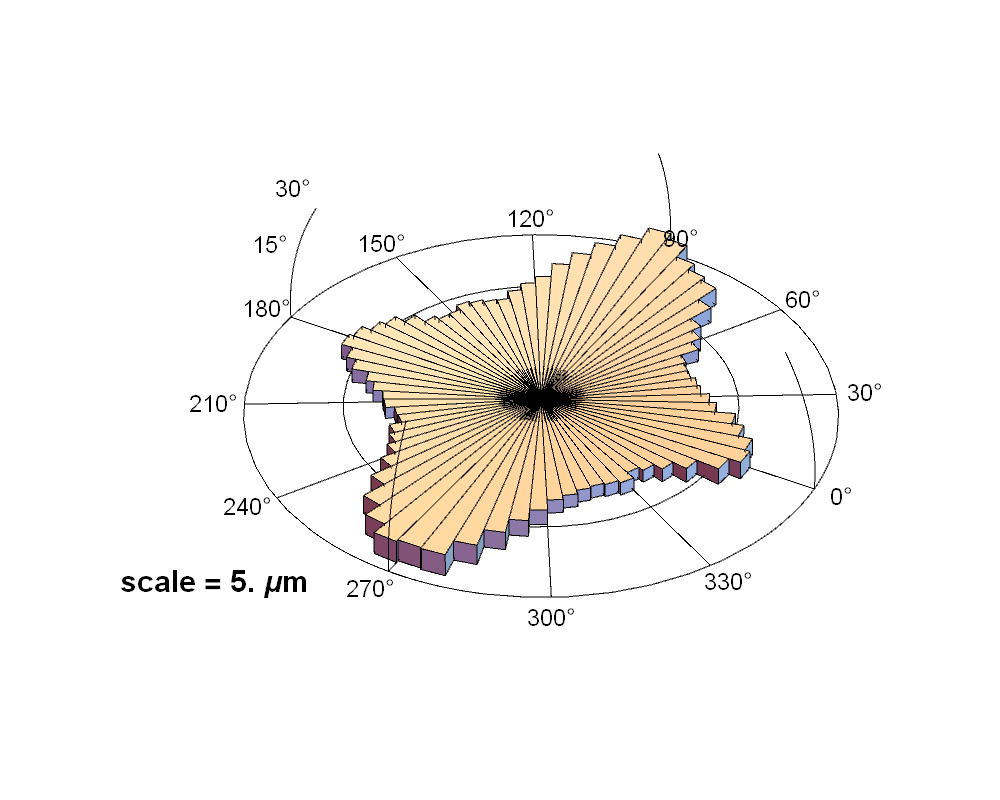

Supplement: Supplementary file 1 [file materials-13-03028-s001.zip › supplementary data/Multiscale curvature analysis/3D rosetta plots/microEDMed_40withlabel.tiff]

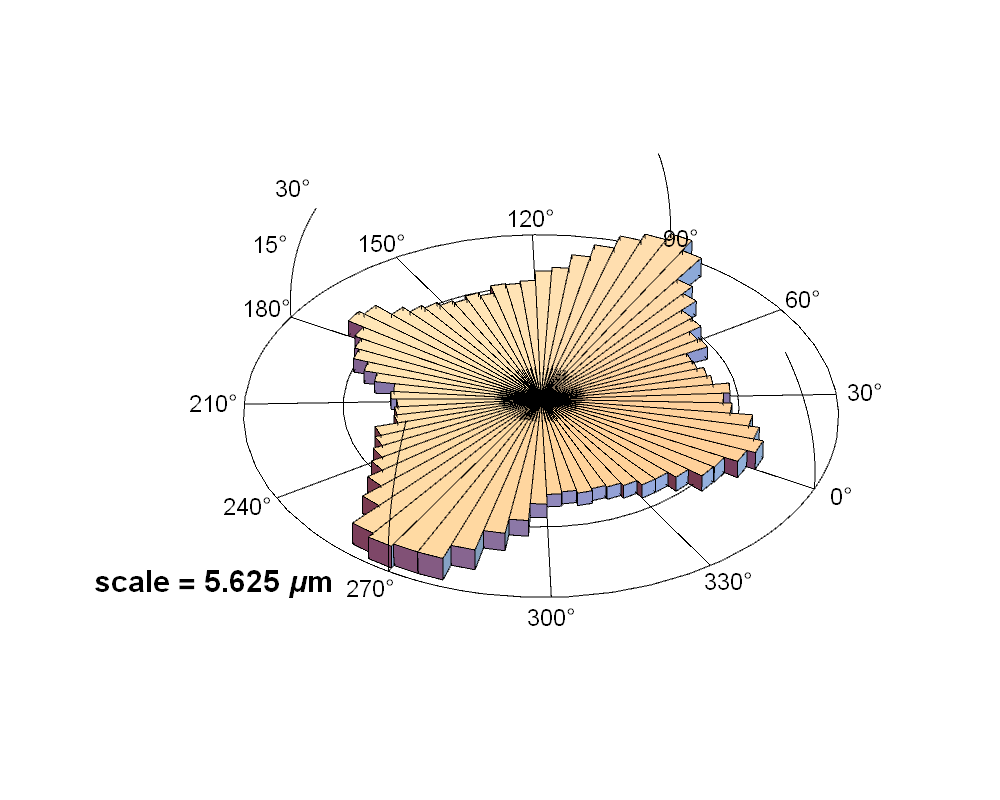

Supplement: Supplementary file 1 [file materials-13-03028-s001.zip › supplementary data/Multiscale curvature analysis/3D rosetta plots/microEDMed_45withlabel.tiff]

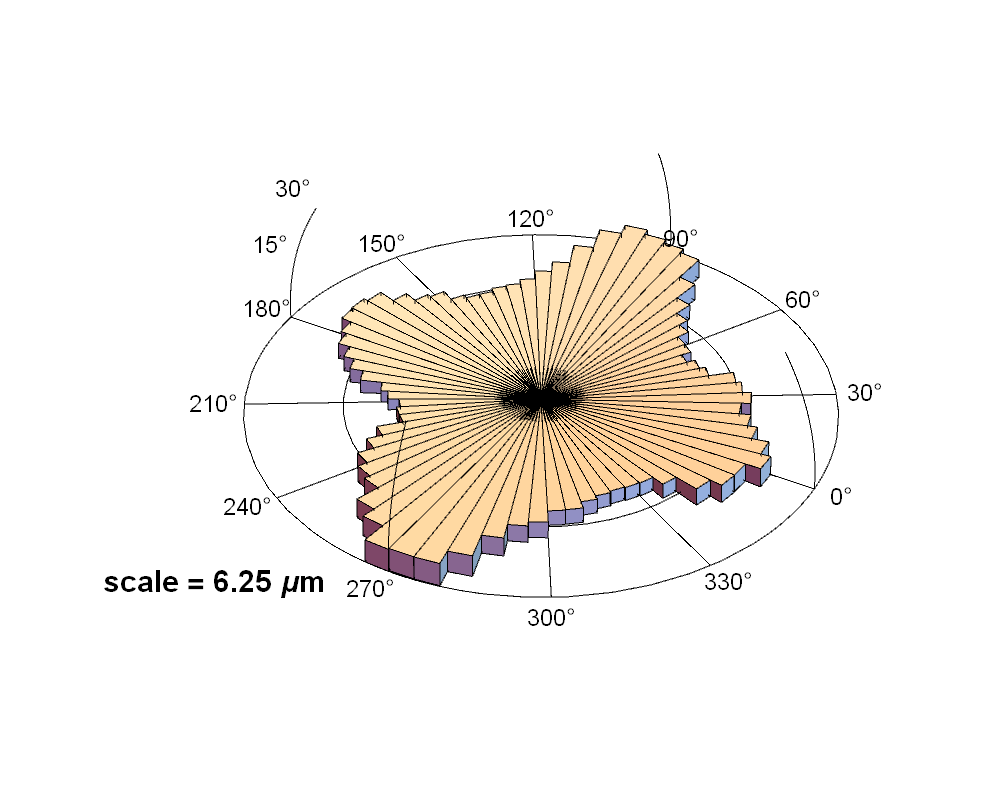

Supplement: Supplementary file 1 [file materials-13-03028-s001.zip › supplementary data/Multiscale curvature analysis/3D rosetta plots/microEDMed_50withlabel.tiff]

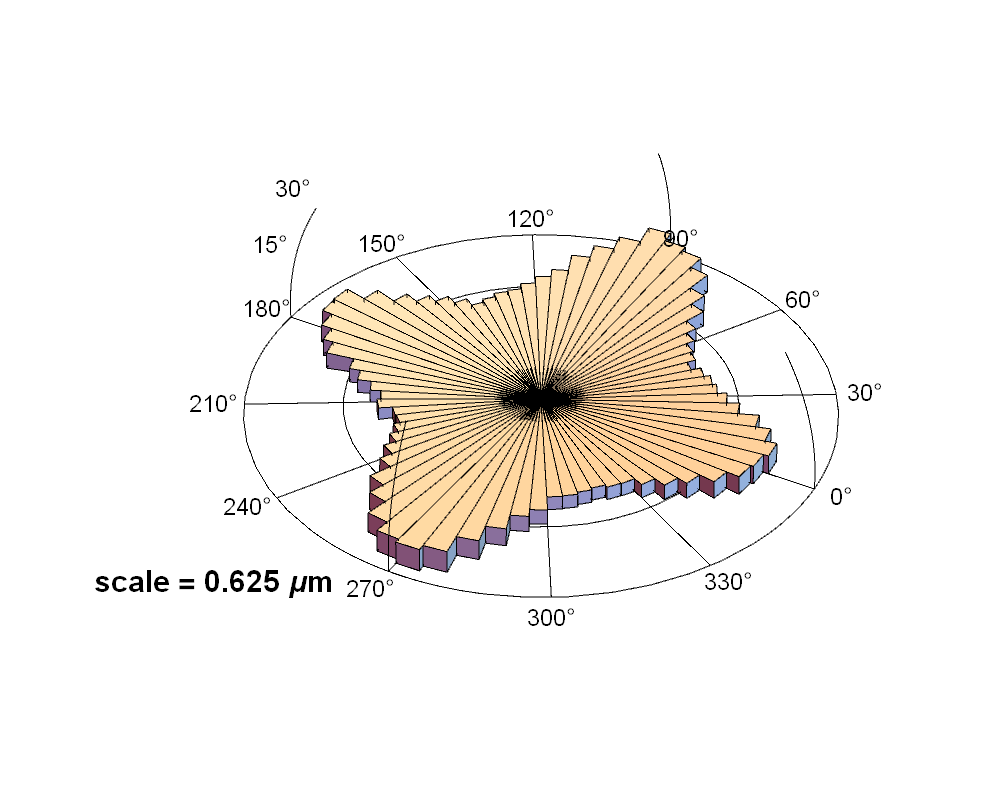

Supplement: Supplementary file 1 [file materials-13-03028-s001.zip › supplementary data/Multiscale curvature analysis/3D rosetta plots/microEDMed_5withlabel.tiff]

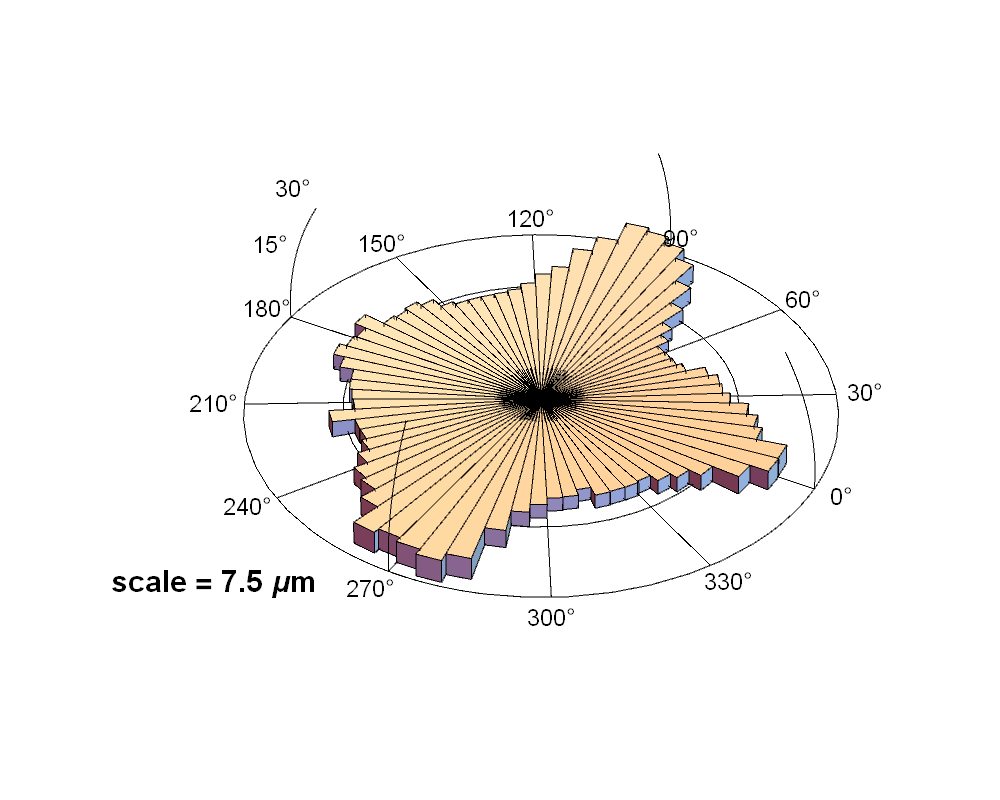

Supplement: Supplementary file 1 [file materials-13-03028-s001.zip › supplementary data/Multiscale curvature analysis/3D rosetta plots/microEDMed_60withlabel.tiff]

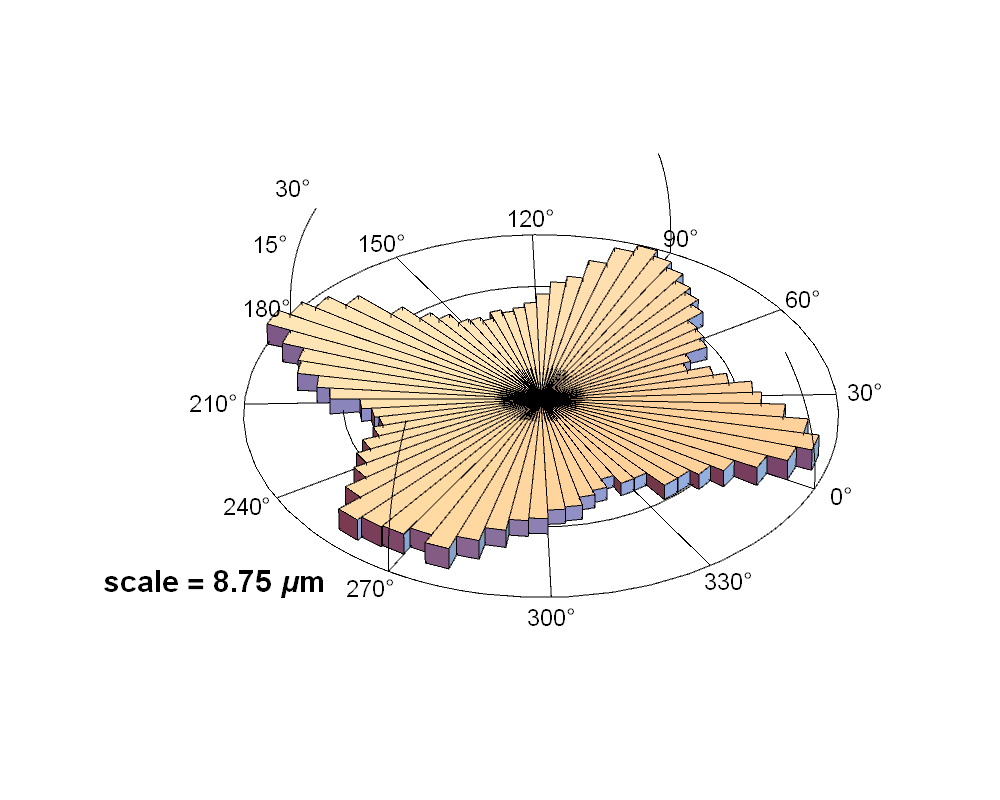

Supplement: Supplementary file 1 [file materials-13-03028-s001.zip › supplementary data/Multiscale curvature analysis/3D rosetta plots/microEDMed_70withlabel.tiff]

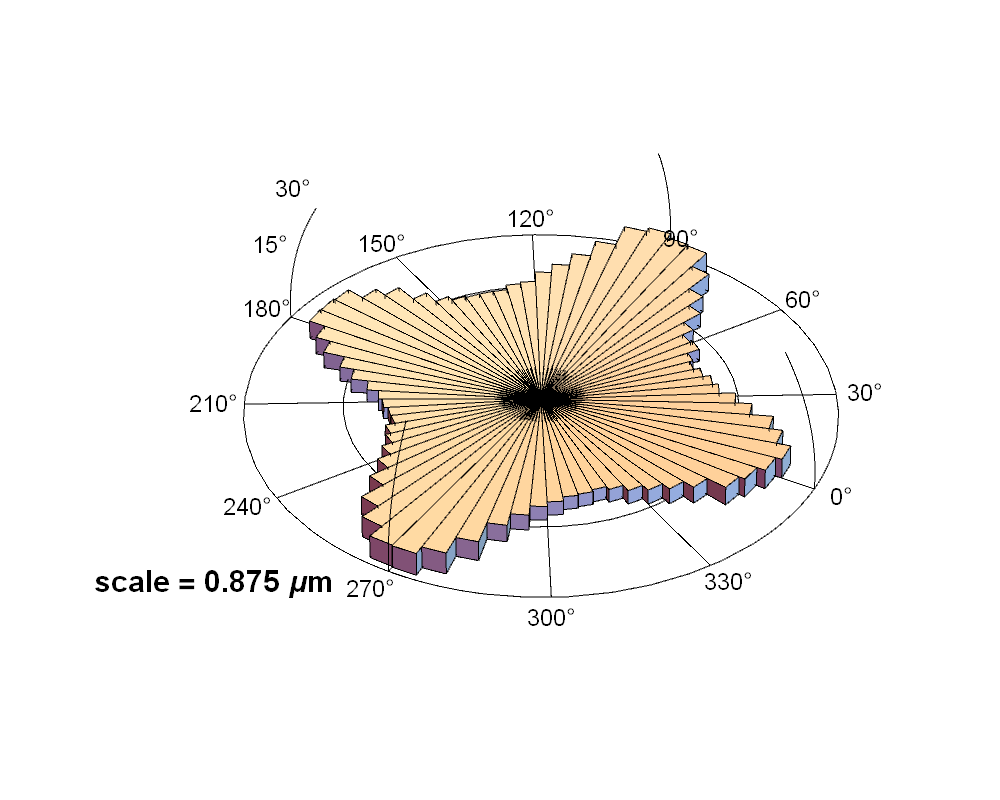

Supplement: Supplementary file 1 [file materials-13-03028-s001.zip › supplementary data/Multiscale curvature analysis/3D rosetta plots/microEDMed_7withlabel.tiff]

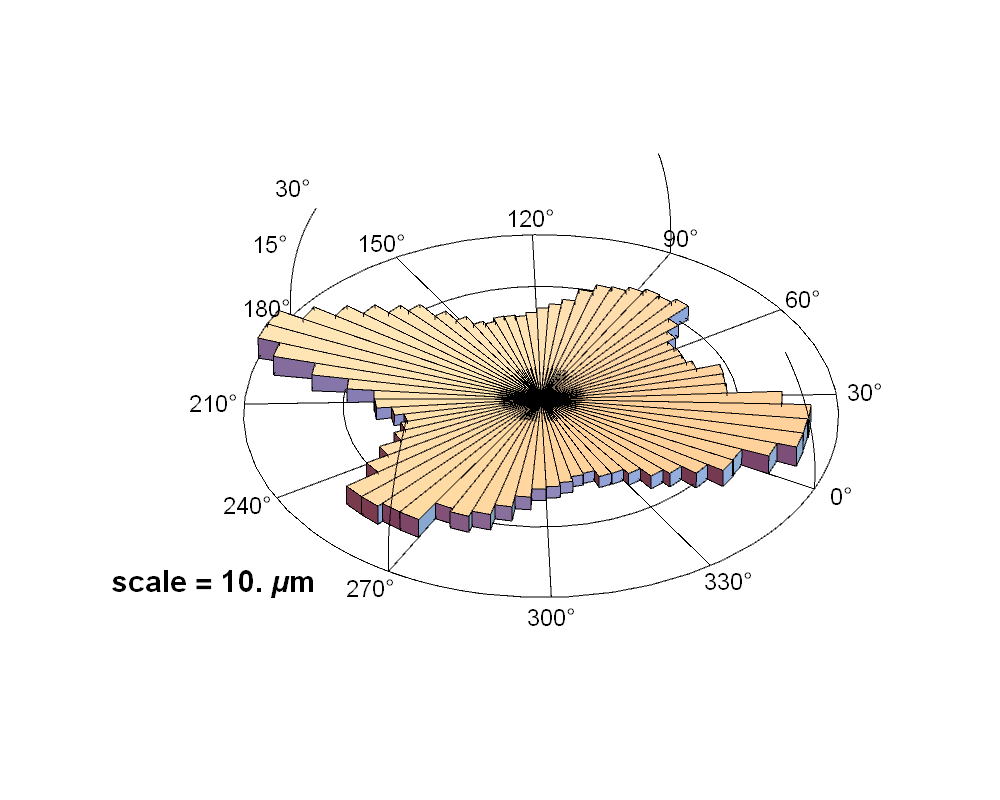

Supplement: Supplementary file 1 [file materials-13-03028-s001.zip › supplementary data/Multiscale curvature analysis/3D rosetta plots/microEDMed_80withlabel.tiff]

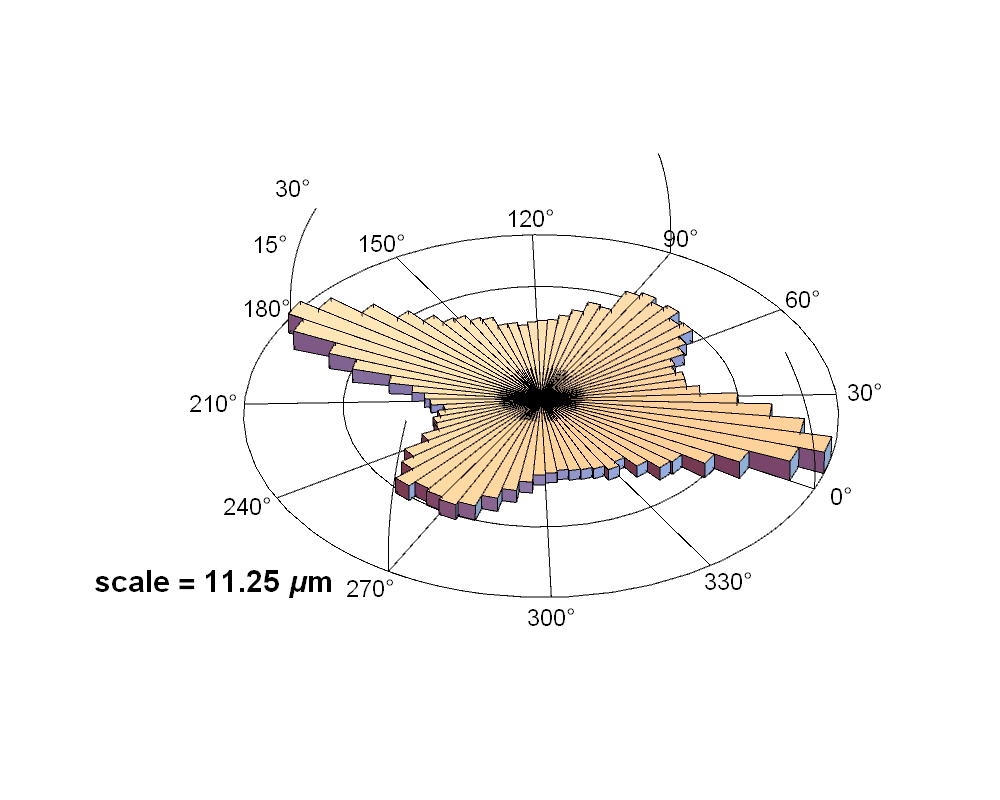

Supplement: Supplementary file 1 [file materials-13-03028-s001.zip › supplementary data/Multiscale curvature analysis/3D rosetta plots/microEDMed_90withlabel.tiff]

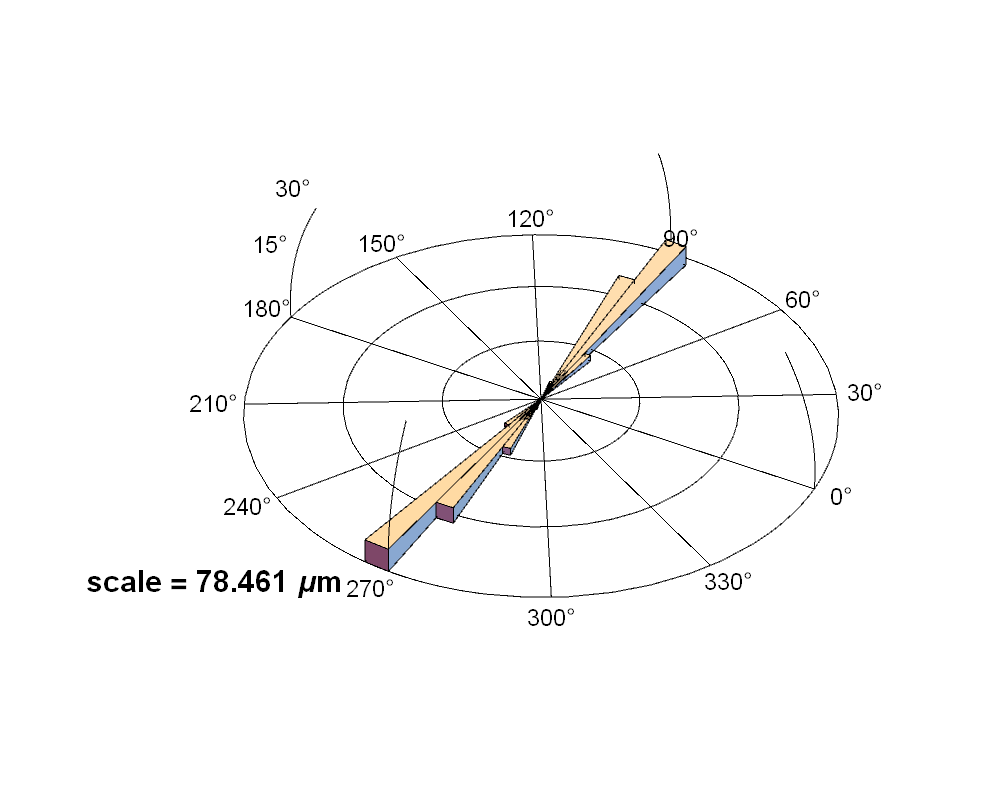

Supplement: Supplementary file 1 [file materials-13-03028-s001.zip › supplementary data/Multiscale curvature analysis/3D rosetta plots/MilledC_100withlabel.tiff]

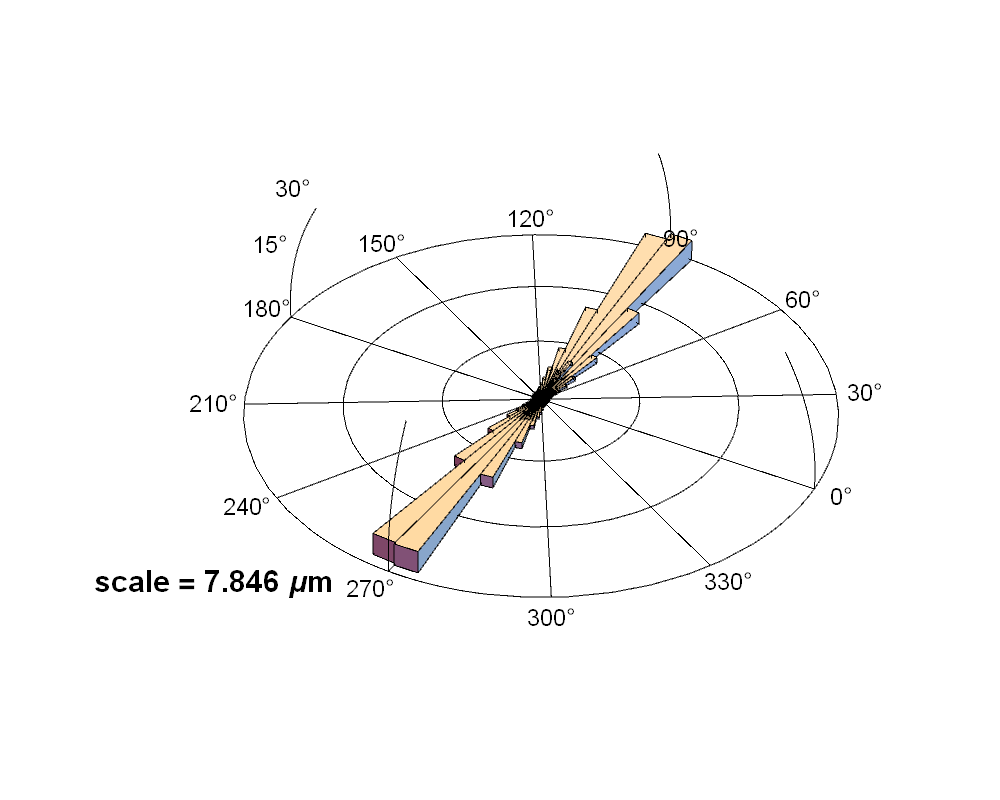

Supplement: Supplementary file 1 [file materials-13-03028-s001.zip › supplementary data/Multiscale curvature analysis/3D rosetta plots/MilledC_10withlabel.tiff]

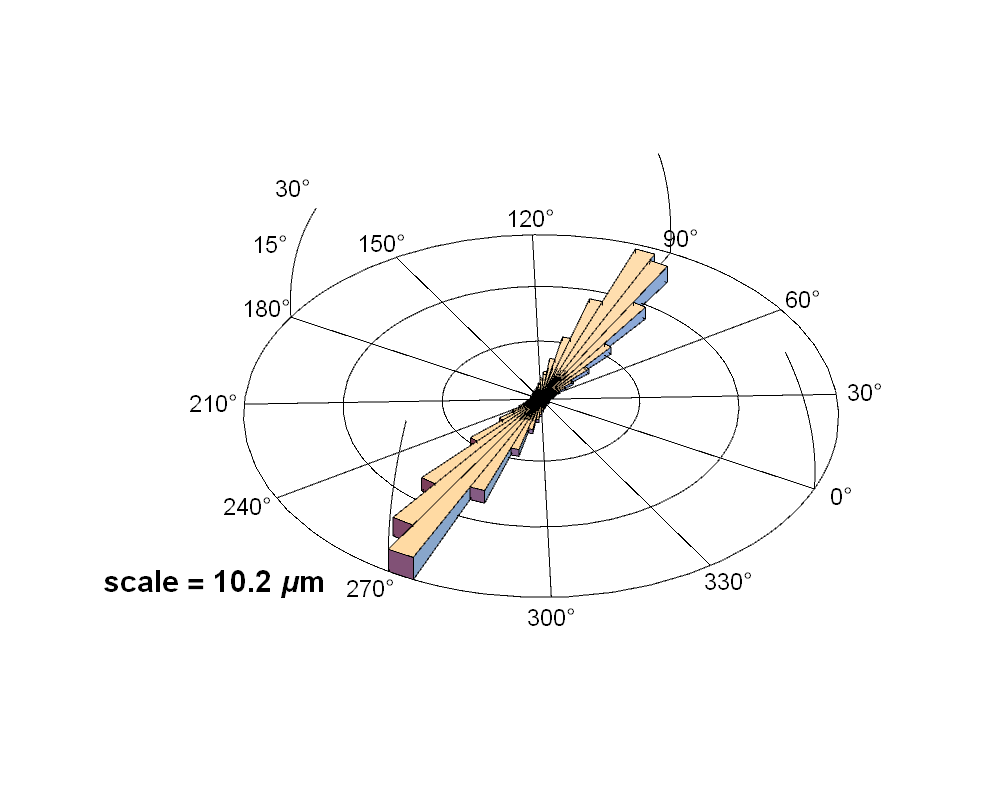

Supplement: Supplementary file 1 [file materials-13-03028-s001.zip › supplementary data/Multiscale curvature analysis/3D rosetta plots/MilledC_13withlabel.tiff]

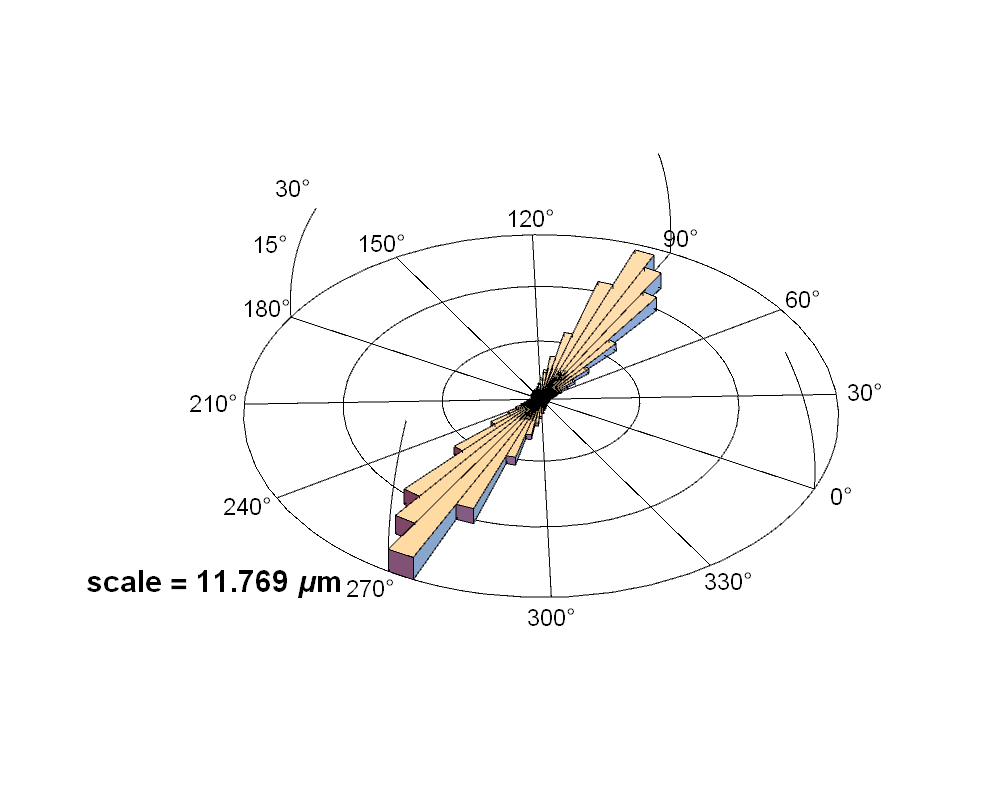

Supplement: Supplementary file 1 [file materials-13-03028-s001.zip › supplementary data/Multiscale curvature analysis/3D rosetta plots/MilledC_15withlabel.tiff]

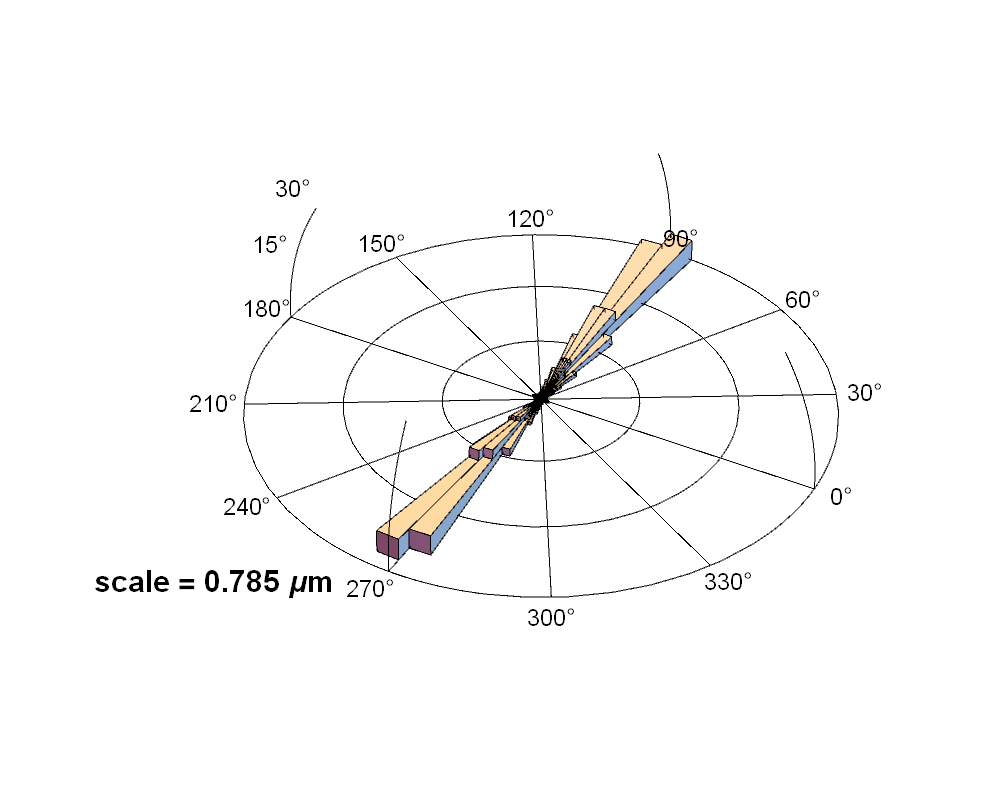

Supplement: Supplementary file 1 [file materials-13-03028-s001.zip › supplementary data/Multiscale curvature analysis/3D rosetta plots/MilledC_1withlabel.tiff]

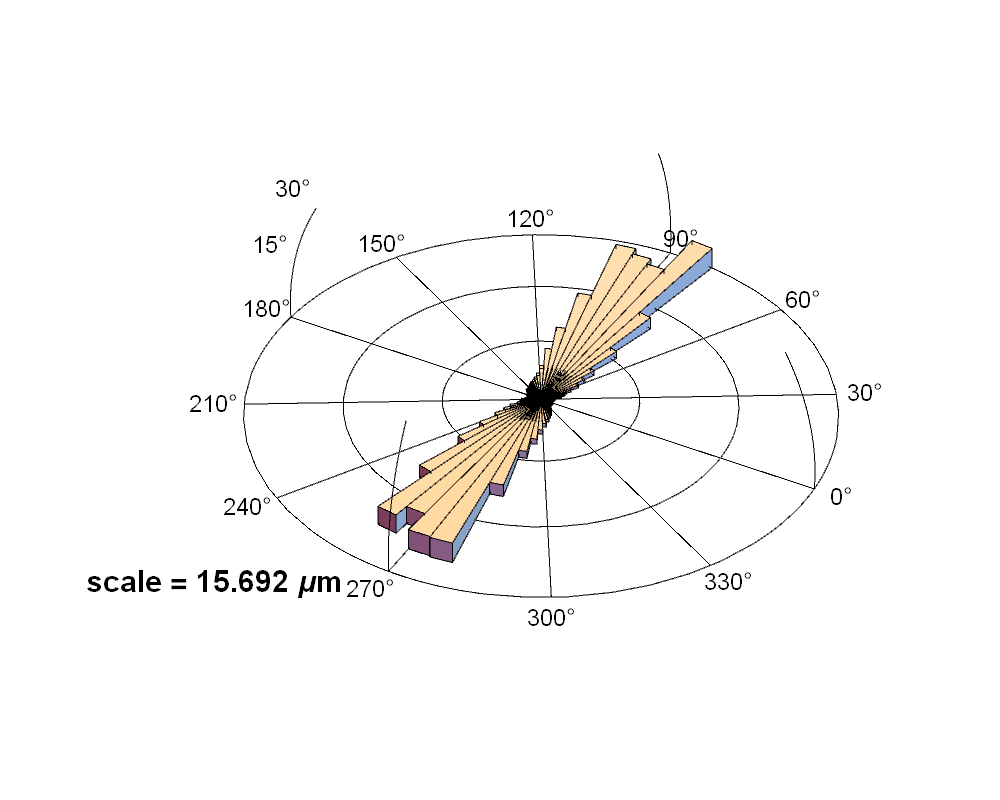

Supplement: Supplementary file 1 [file materials-13-03028-s001.zip › supplementary data/Multiscale curvature analysis/3D rosetta plots/MilledC_20withlabel.tiff]

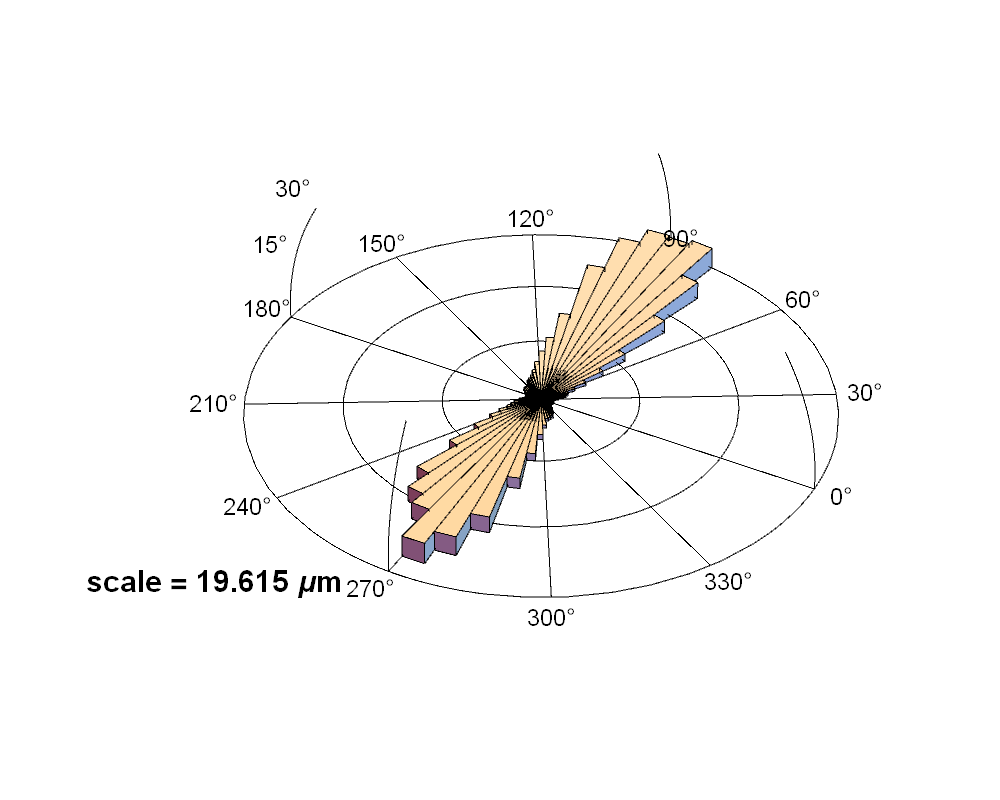

Supplement: Supplementary file 1 [file materials-13-03028-s001.zip › supplementary data/Multiscale curvature analysis/3D rosetta plots/MilledC_25withlabel.tiff]

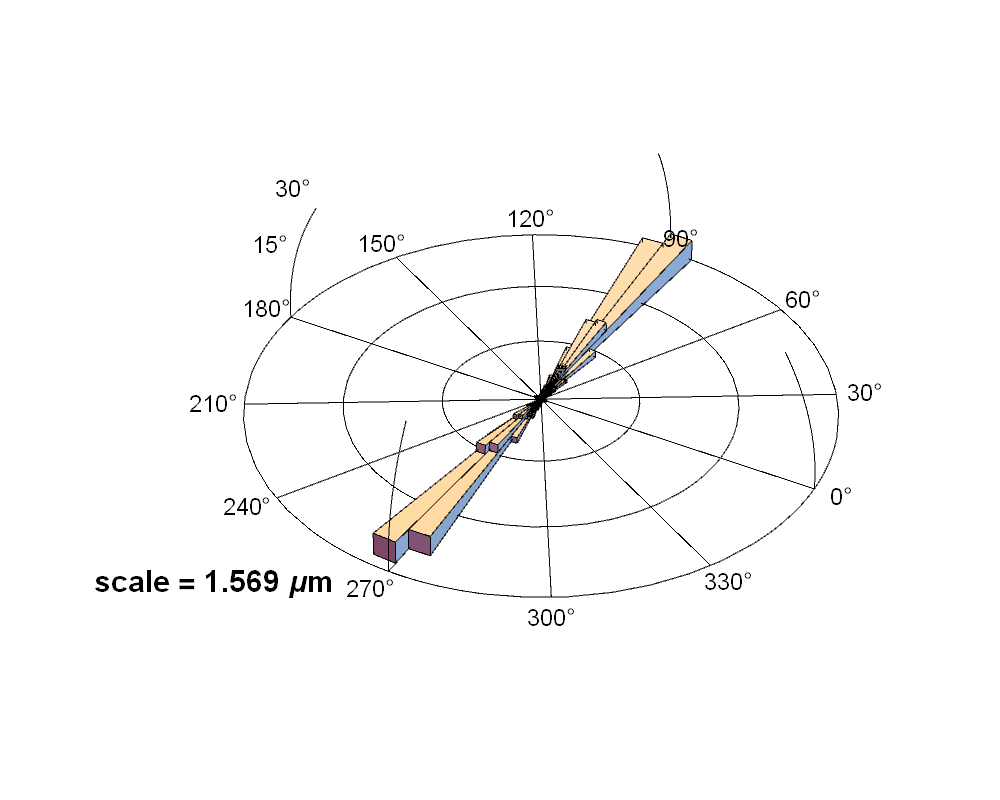

Supplement: Supplementary file 1 [file materials-13-03028-s001.zip › supplementary data/Multiscale curvature analysis/3D rosetta plots/MilledC_2withlabel.tiff]

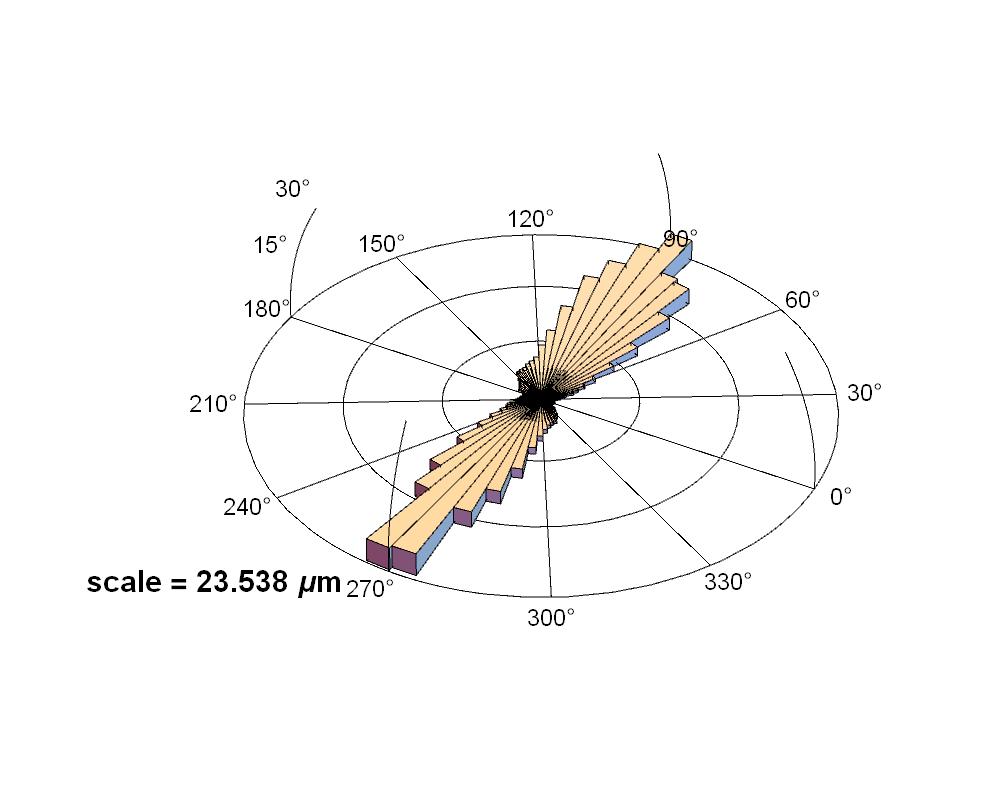

Supplement: Supplementary file 1 [file materials-13-03028-s001.zip › supplementary data/Multiscale curvature analysis/3D rosetta plots/MilledC_30withlabel.tiff]

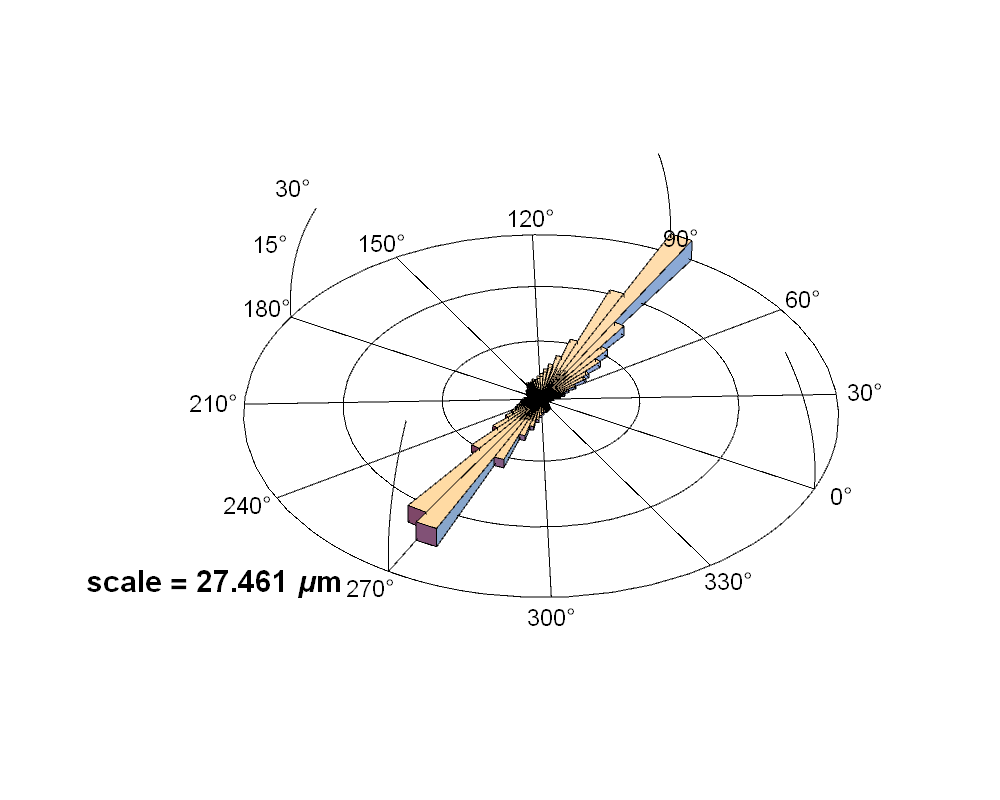

Supplement: Supplementary file 1 [file materials-13-03028-s001.zip › supplementary data/Multiscale curvature analysis/3D rosetta plots/MilledC_35withlabel.tiff]

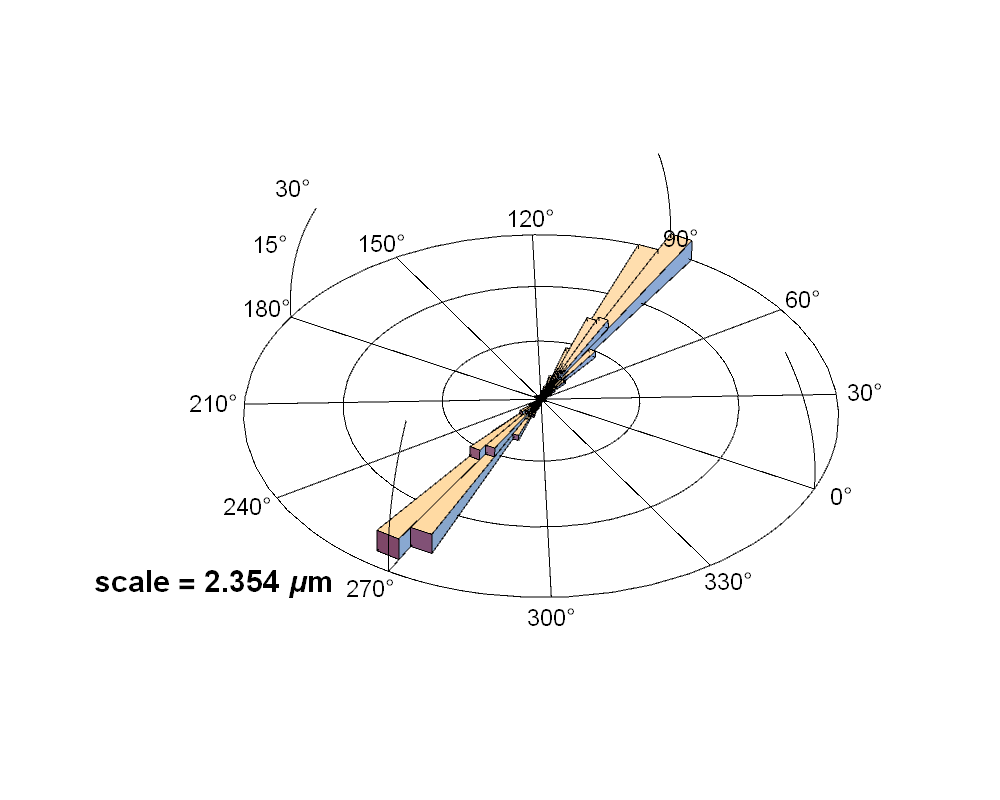

Supplement: Supplementary file 1 [file materials-13-03028-s001.zip › supplementary data/Multiscale curvature analysis/3D rosetta plots/MilledC_3withlabel.tiff]

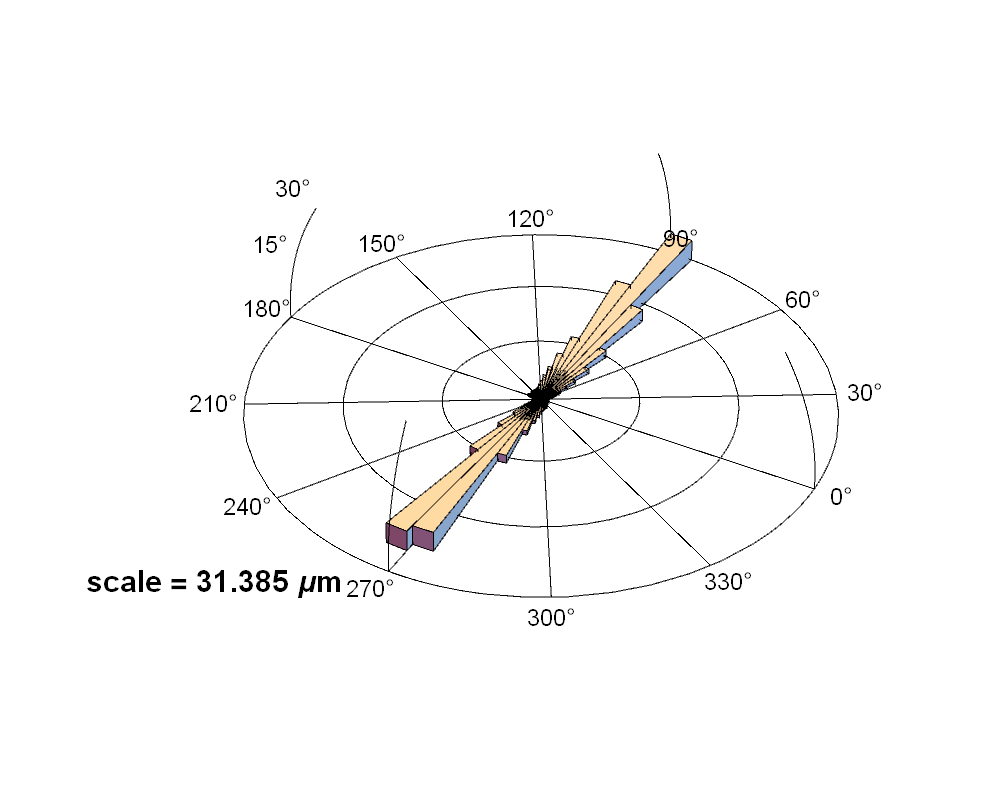

Supplement: Supplementary file 1 [file materials-13-03028-s001.zip › supplementary data/Multiscale curvature analysis/3D rosetta plots/MilledC_40withlabel.tiff]

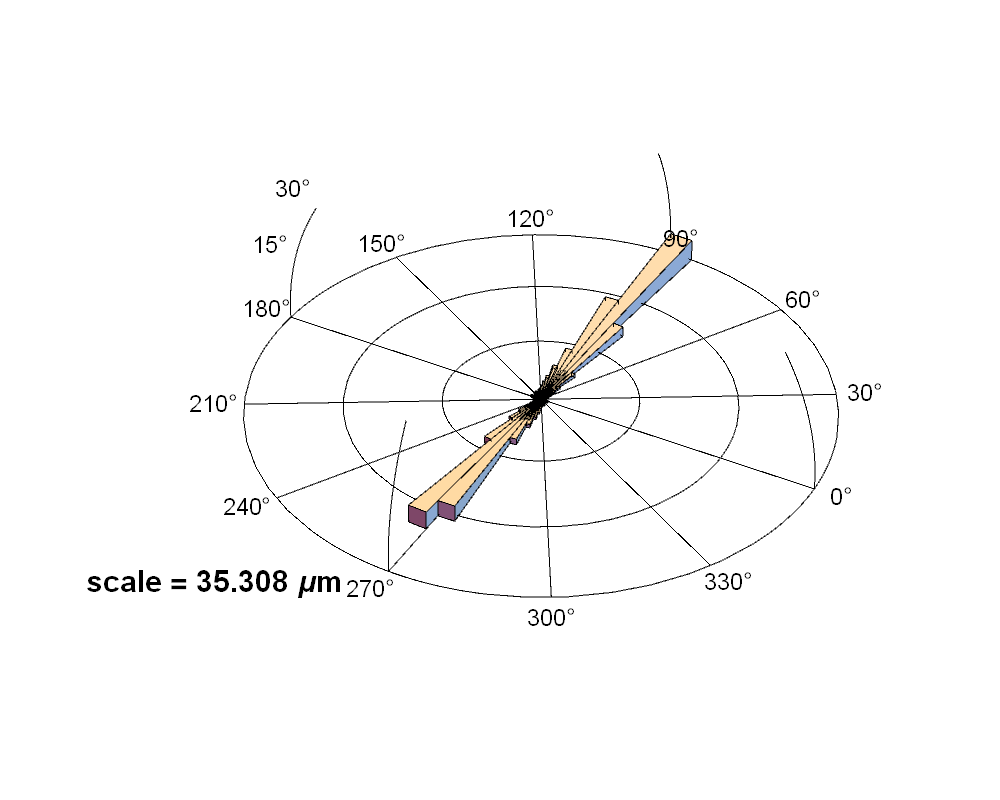

Supplement: Supplementary file 1 [file materials-13-03028-s001.zip › supplementary data/Multiscale curvature analysis/3D rosetta plots/MilledC_45withlabel.tiff]

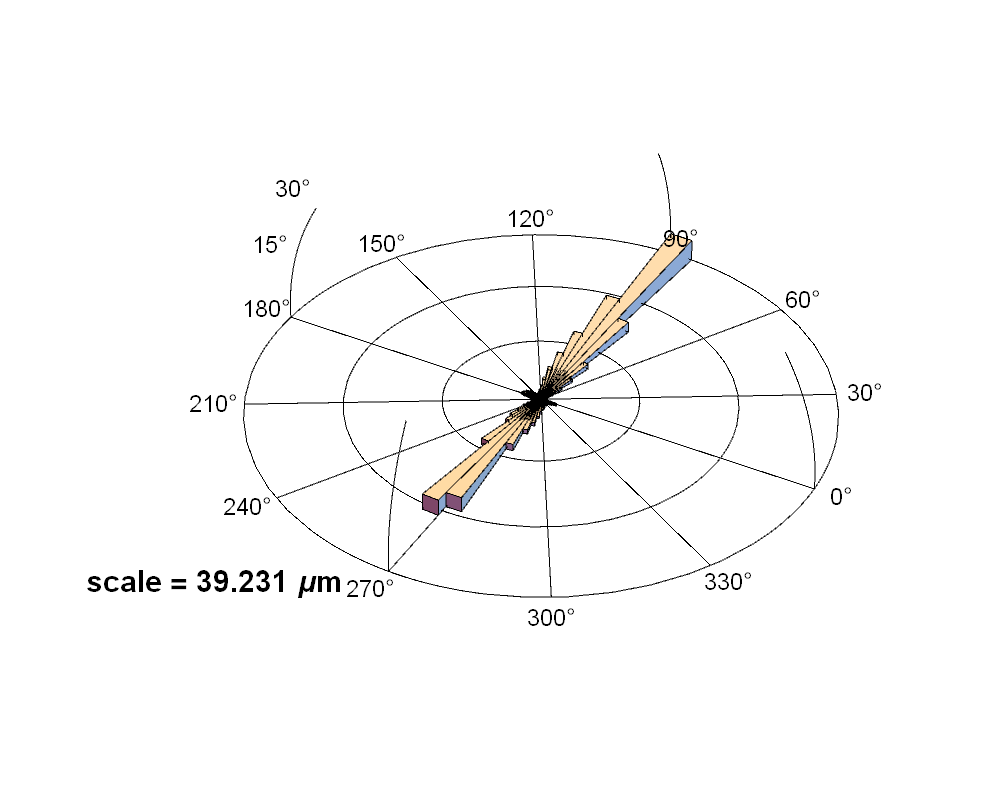

Supplement: Supplementary file 1 [file materials-13-03028-s001.zip › supplementary data/Multiscale curvature analysis/3D rosetta plots/MilledC_50withlabel.tiff]

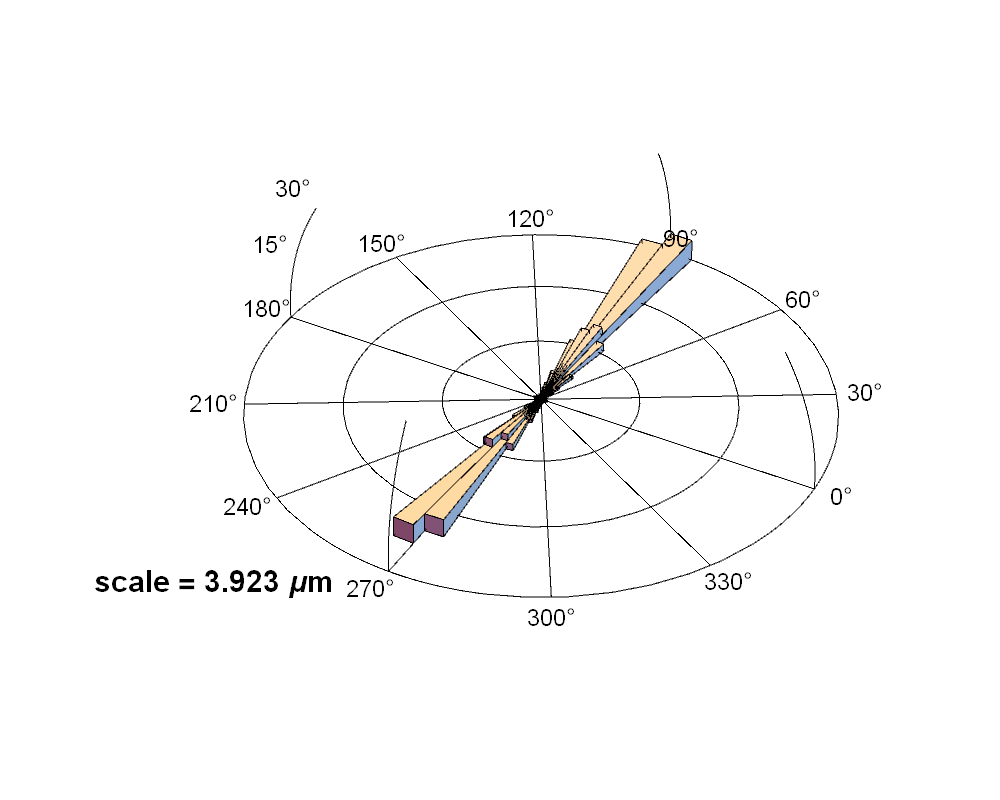

Supplement: Supplementary file 1 [file materials-13-03028-s001.zip › supplementary data/Multiscale curvature analysis/3D rosetta plots/MilledC_5withlabel.tiff]

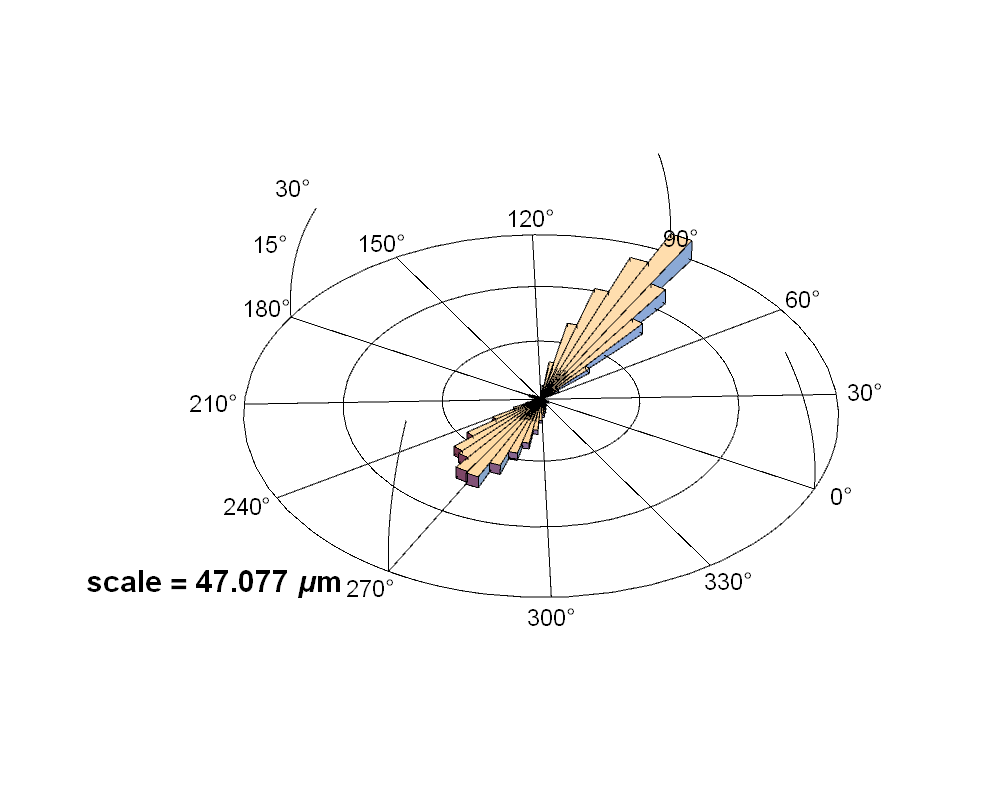

Supplement: Supplementary file 1 [file materials-13-03028-s001.zip › supplementary data/Multiscale curvature analysis/3D rosetta plots/MilledC_60withlabel.tiff]

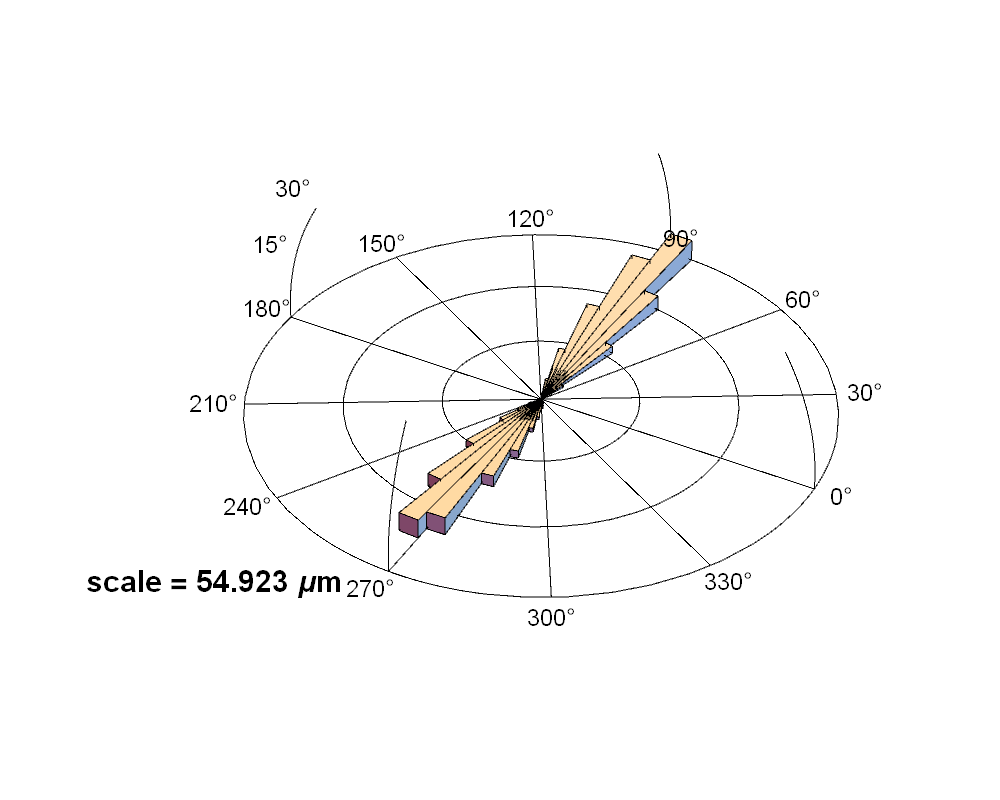

Supplement: Supplementary file 1 [file materials-13-03028-s001.zip › supplementary data/Multiscale curvature analysis/3D rosetta plots/MilledC_70withlabel.tiff]

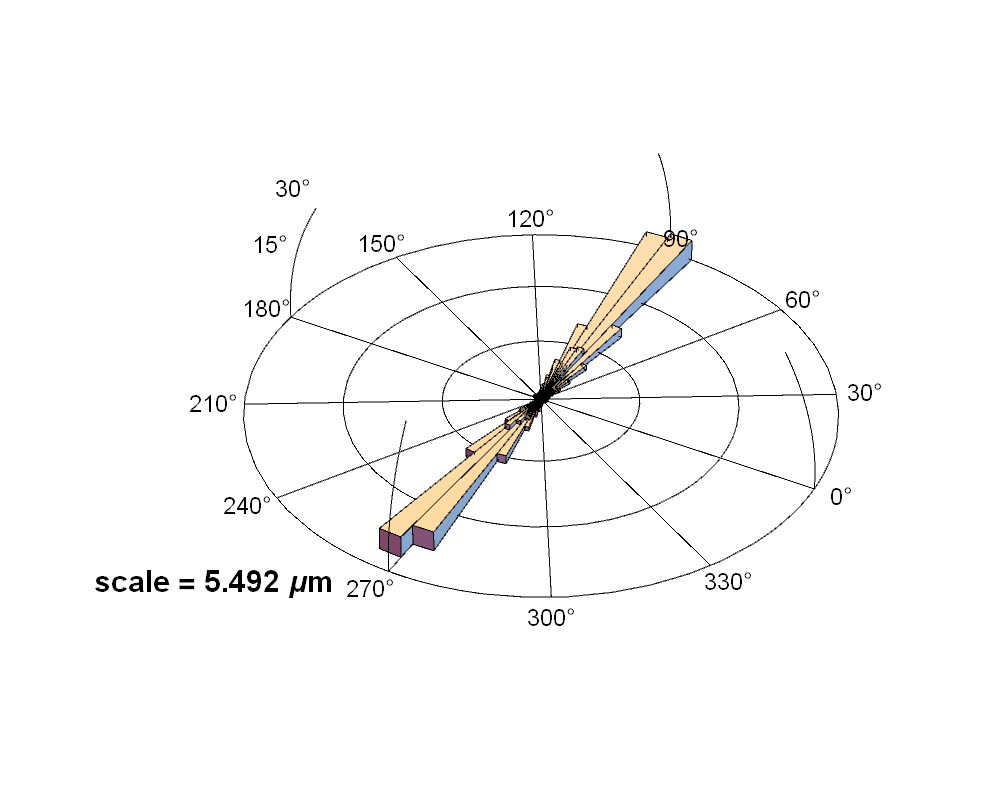

Supplement: Supplementary file 1 [file materials-13-03028-s001.zip › supplementary data/Multiscale curvature analysis/3D rosetta plots/MilledC_7withlabel.tiff]

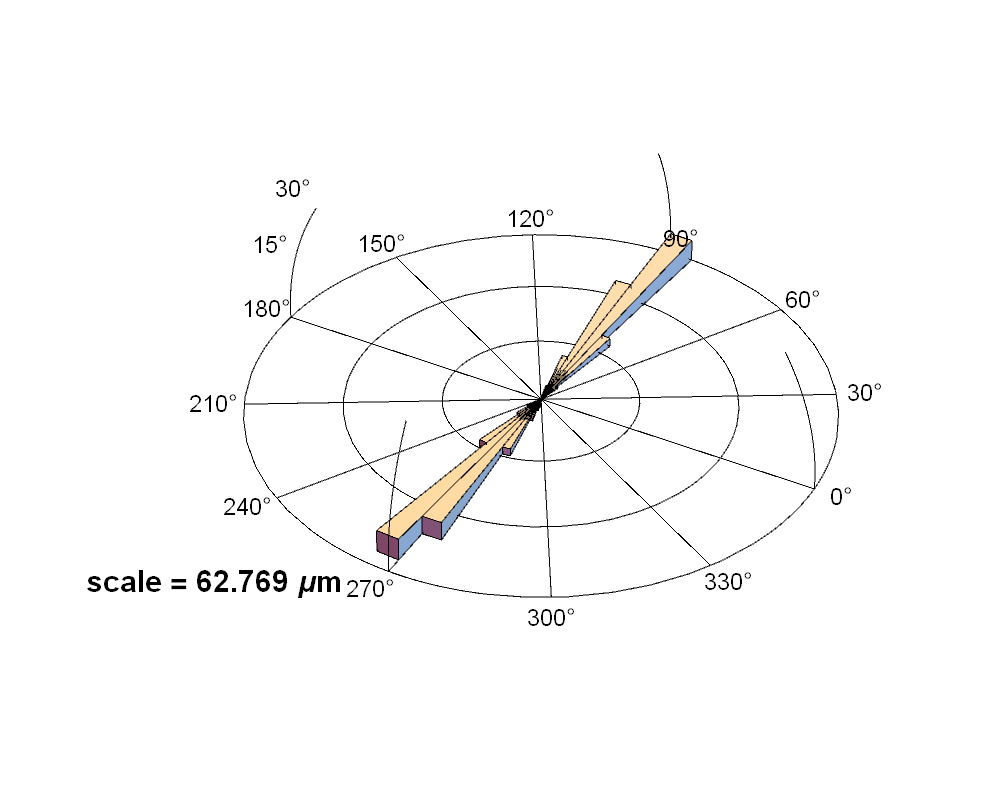

Supplement: Supplementary file 1 [file materials-13-03028-s001.zip › supplementary data/Multiscale curvature analysis/3D rosetta plots/MilledC_80withlabel.tiff]

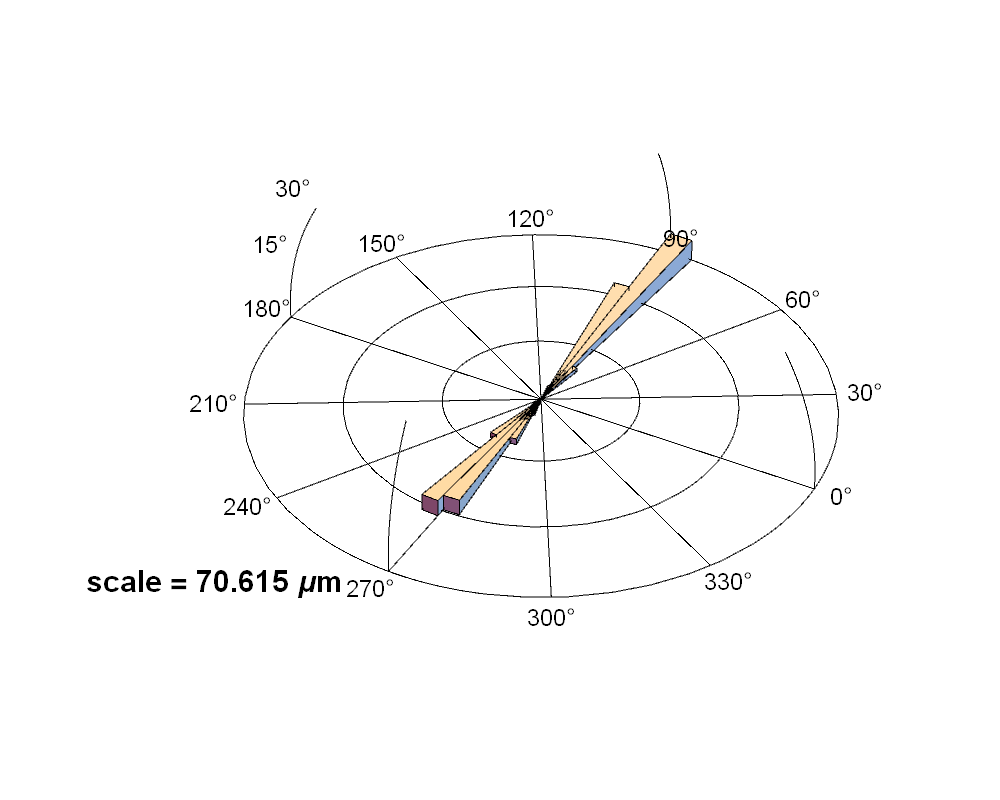

Supplement: Supplementary file 1 [file materials-13-03028-s001.zip › supplementary data/Multiscale curvature analysis/3D rosetta plots/MilledC_90withlabel.tiff]

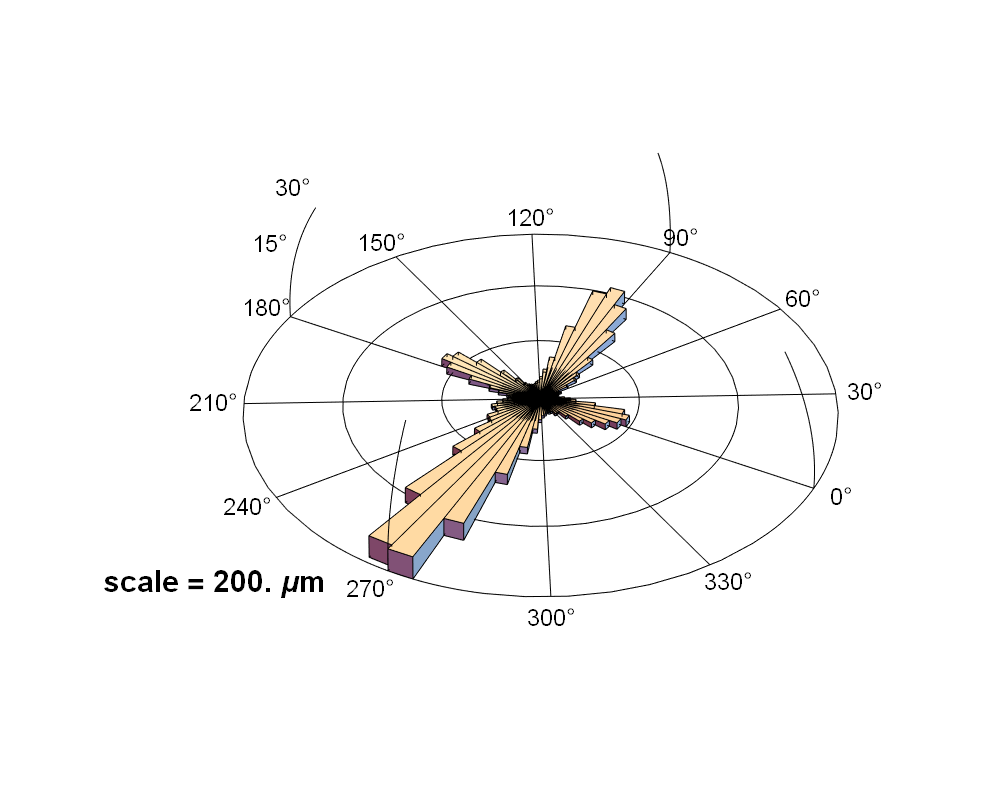

Supplement: Supplementary file 1 [file materials-13-03028-s001.zip › supplementary data/Multiscale curvature analysis/3D rosetta plots/MilledF_100withlabel.tiff]

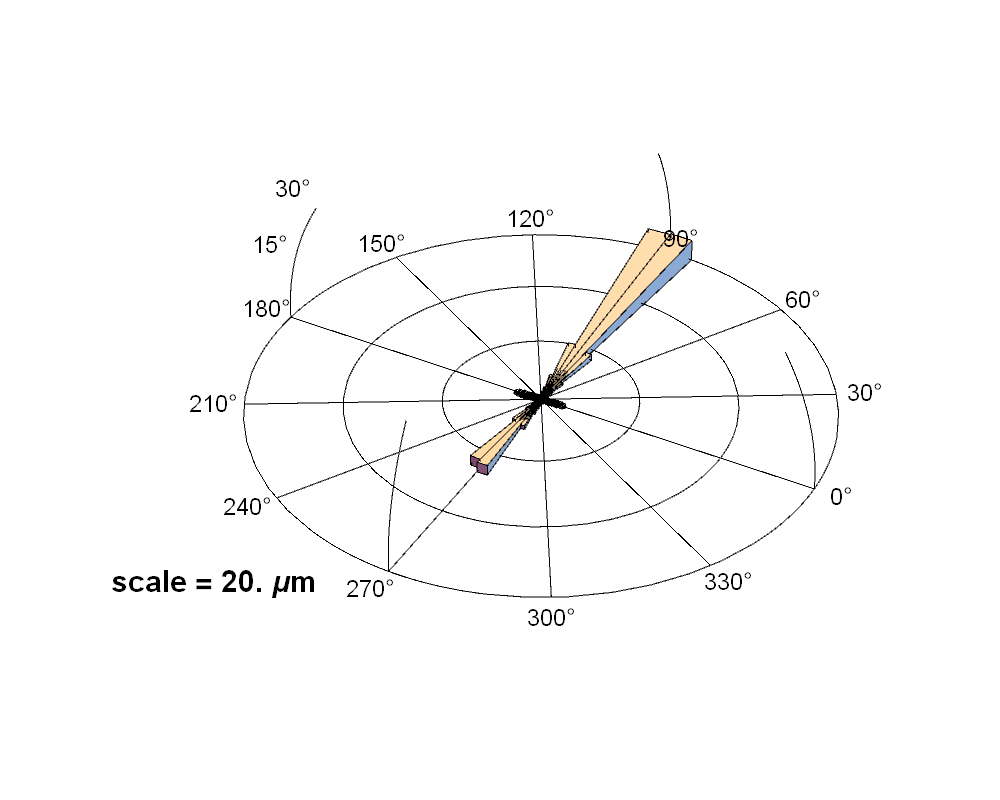

Supplement: Supplementary file 1 [file materials-13-03028-s001.zip › supplementary data/Multiscale curvature analysis/3D rosetta plots/MilledF_10withlabel.tiff]

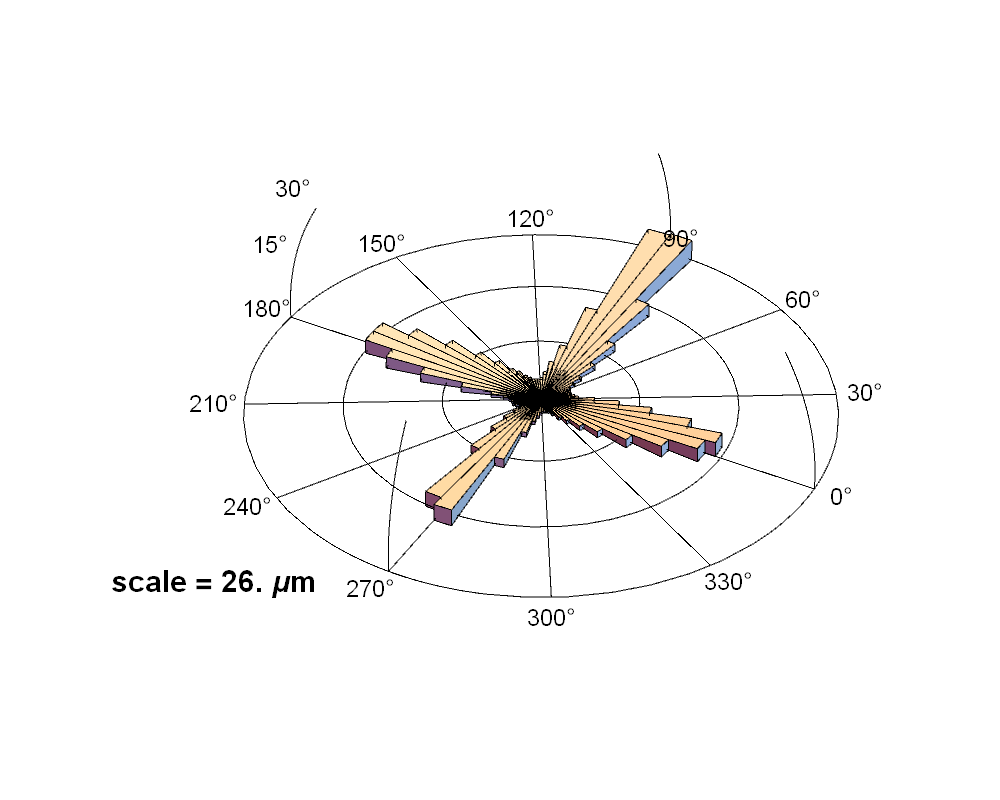

Supplement: Supplementary file 1 [file materials-13-03028-s001.zip › supplementary data/Multiscale curvature analysis/3D rosetta plots/MilledF_13withlabel.tiff]

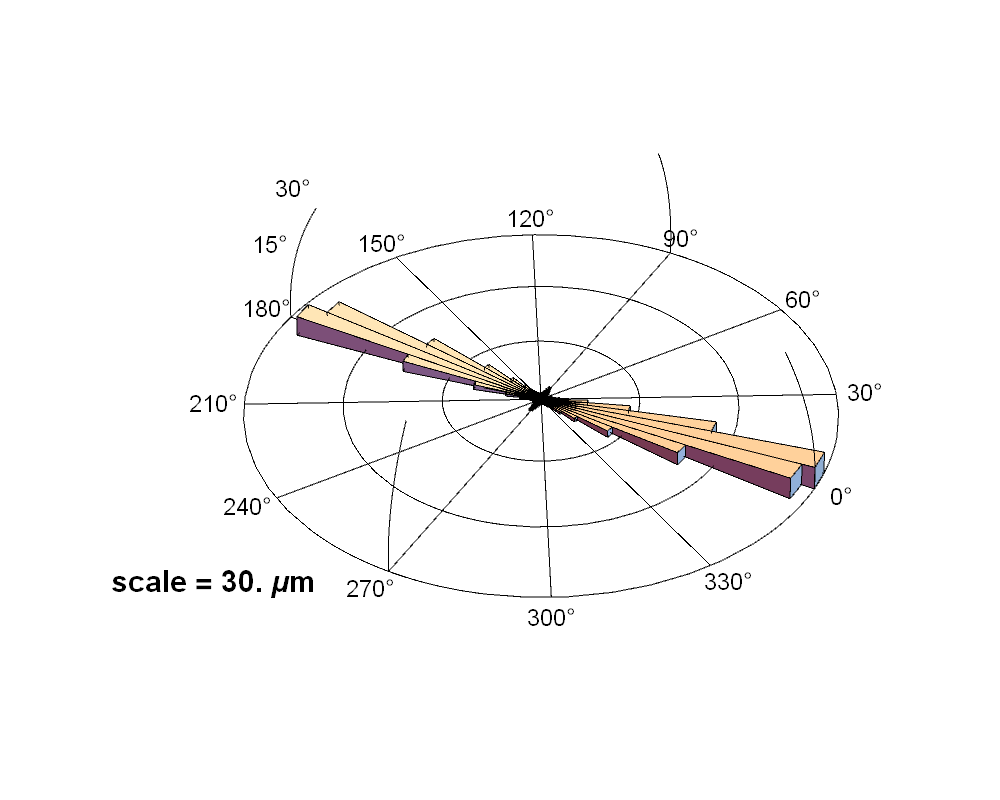

Supplement: Supplementary file 1 [file materials-13-03028-s001.zip › supplementary data/Multiscale curvature analysis/3D rosetta plots/MilledF_15withlabel.tiff]

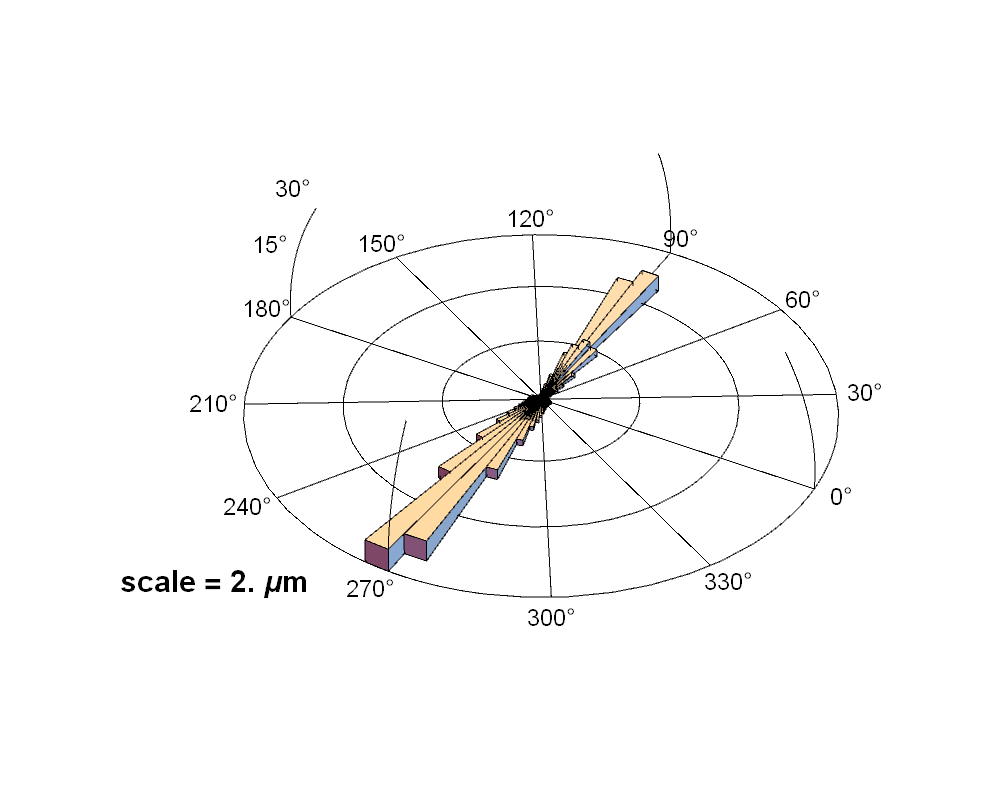

Supplement: Supplementary file 1 [file materials-13-03028-s001.zip › supplementary data/Multiscale curvature analysis/3D rosetta plots/MilledF_1withlabel.tiff]

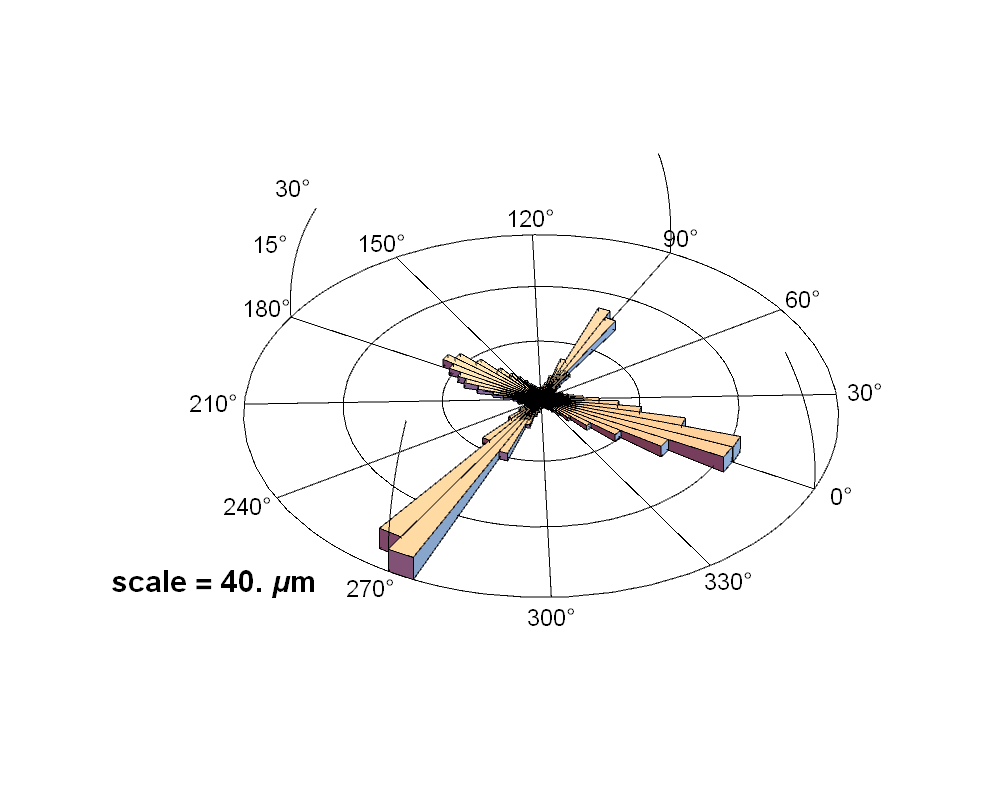

Supplement: Supplementary file 1 [file materials-13-03028-s001.zip › supplementary data/Multiscale curvature analysis/3D rosetta plots/MilledF_20withlabel.tiff]

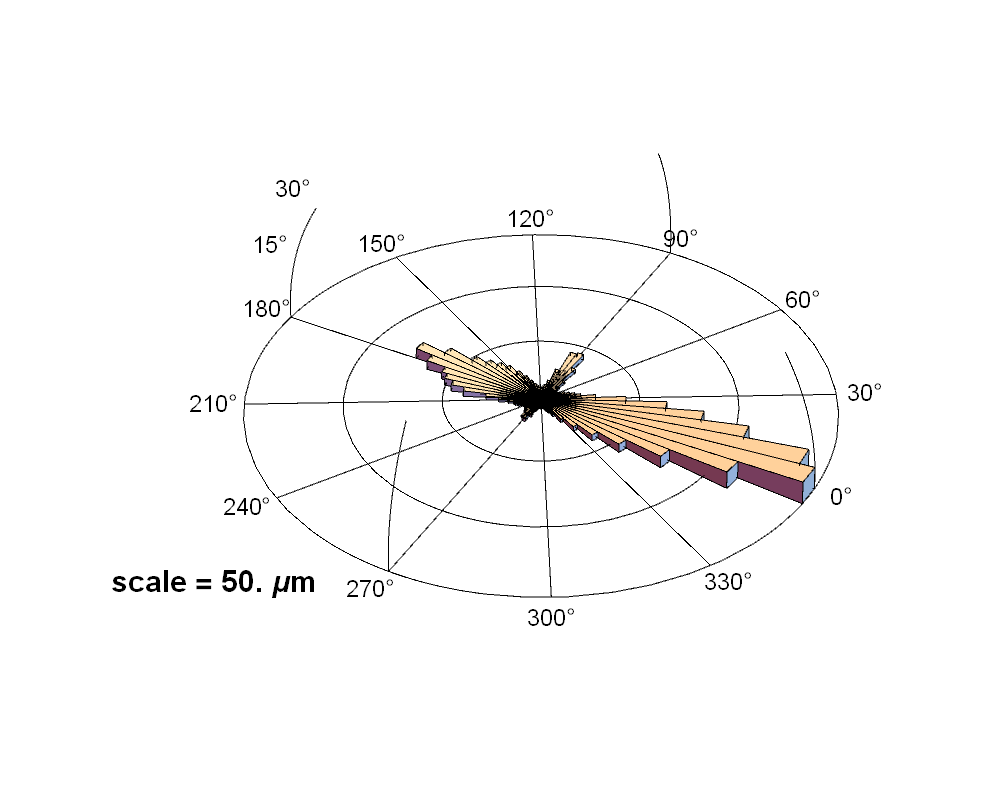

Supplement: Supplementary file 1 [file materials-13-03028-s001.zip › supplementary data/Multiscale curvature analysis/3D rosetta plots/MilledF_25withlabel.tiff]

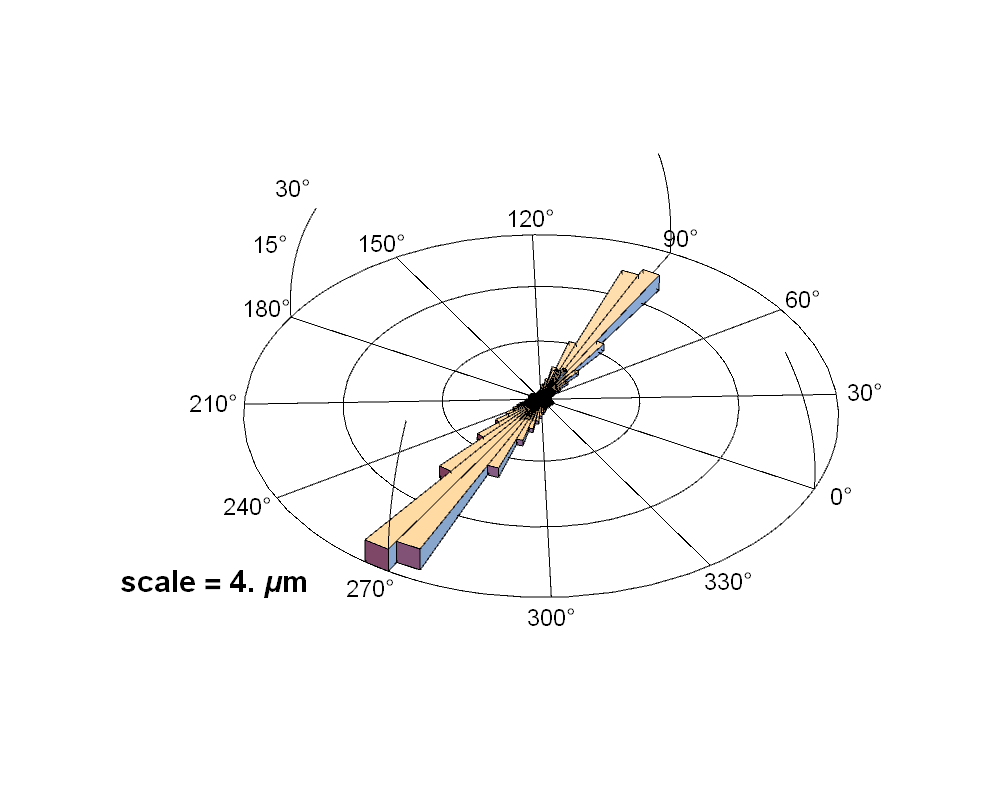

Supplement: Supplementary file 1 [file materials-13-03028-s001.zip › supplementary data/Multiscale curvature analysis/3D rosetta plots/MilledF_2withlabel.tiff]

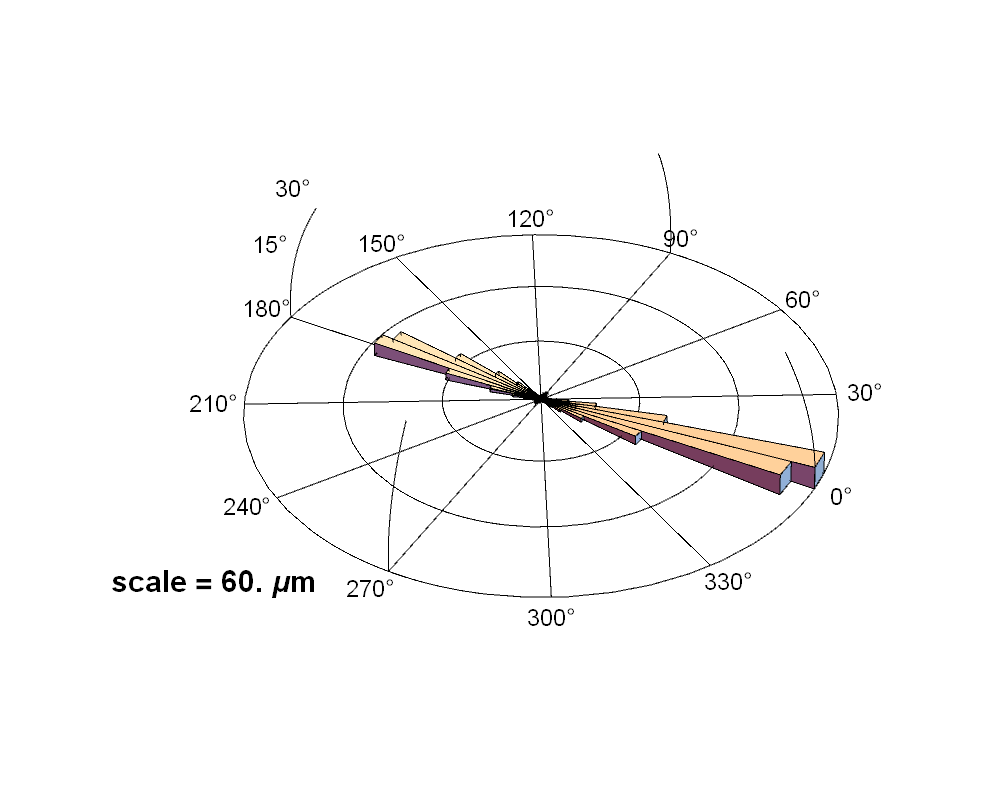

Supplement: Supplementary file 1 [file materials-13-03028-s001.zip › supplementary data/Multiscale curvature analysis/3D rosetta plots/MilledF_30withlabel.tiff]

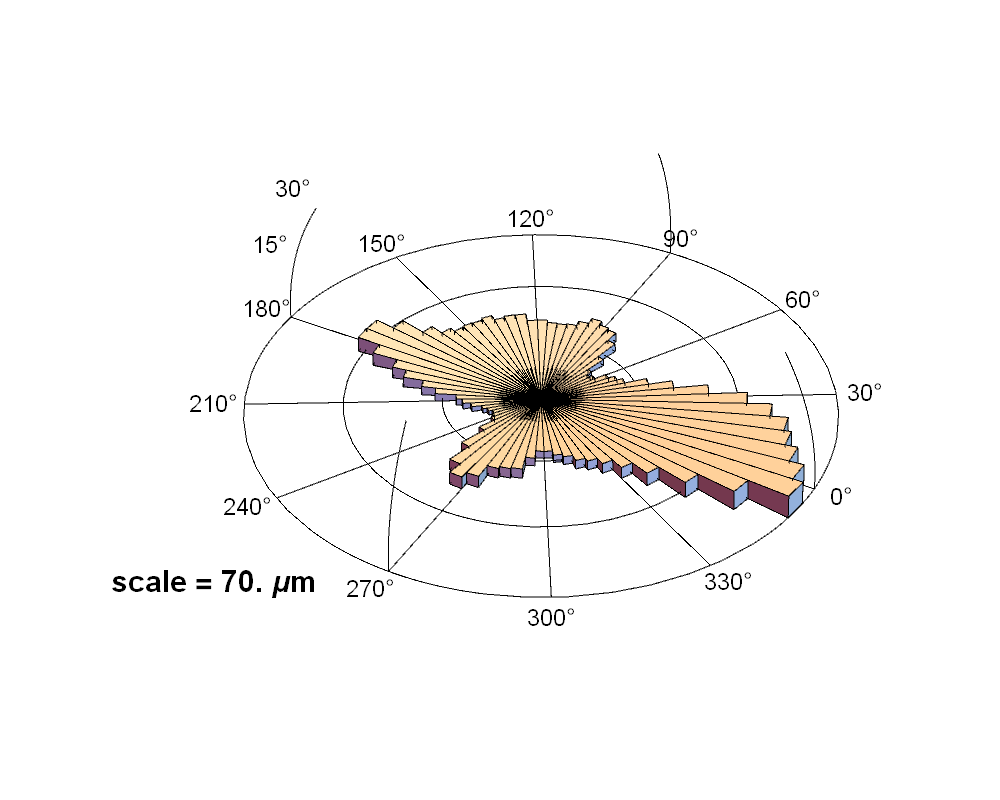

Supplement: Supplementary file 1 [file materials-13-03028-s001.zip › supplementary data/Multiscale curvature analysis/3D rosetta plots/MilledF_35withlabel.tiff]

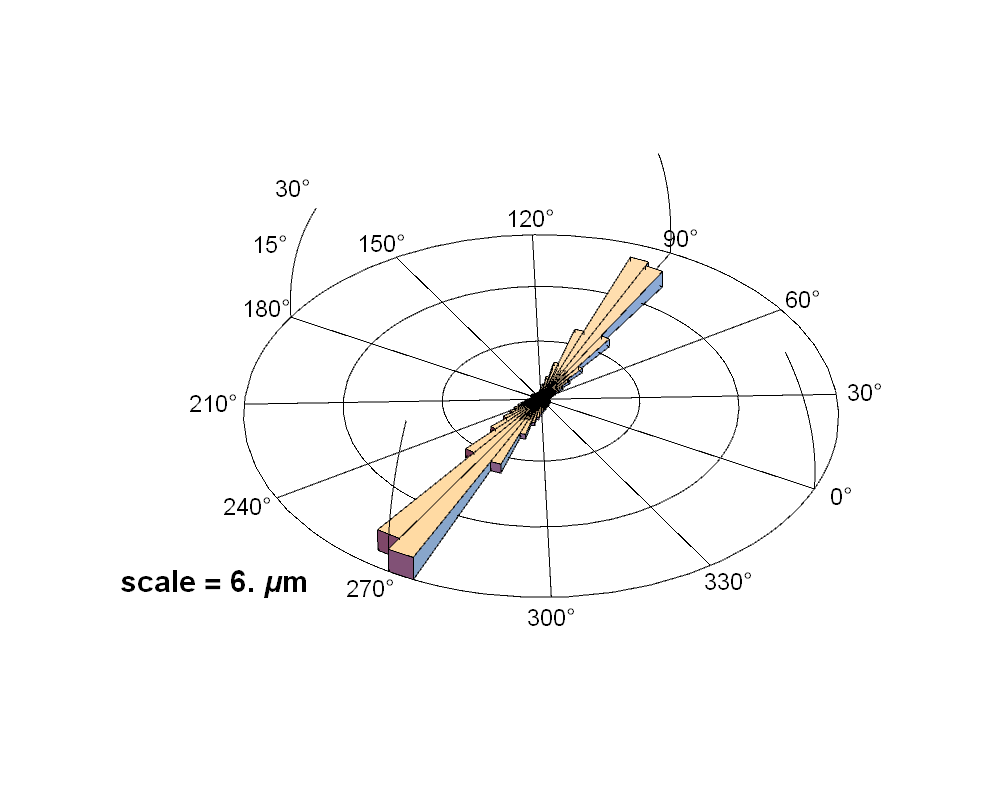

Supplement: Supplementary file 1 [file materials-13-03028-s001.zip › supplementary data/Multiscale curvature analysis/3D rosetta plots/MilledF_3withlabel.tiff]

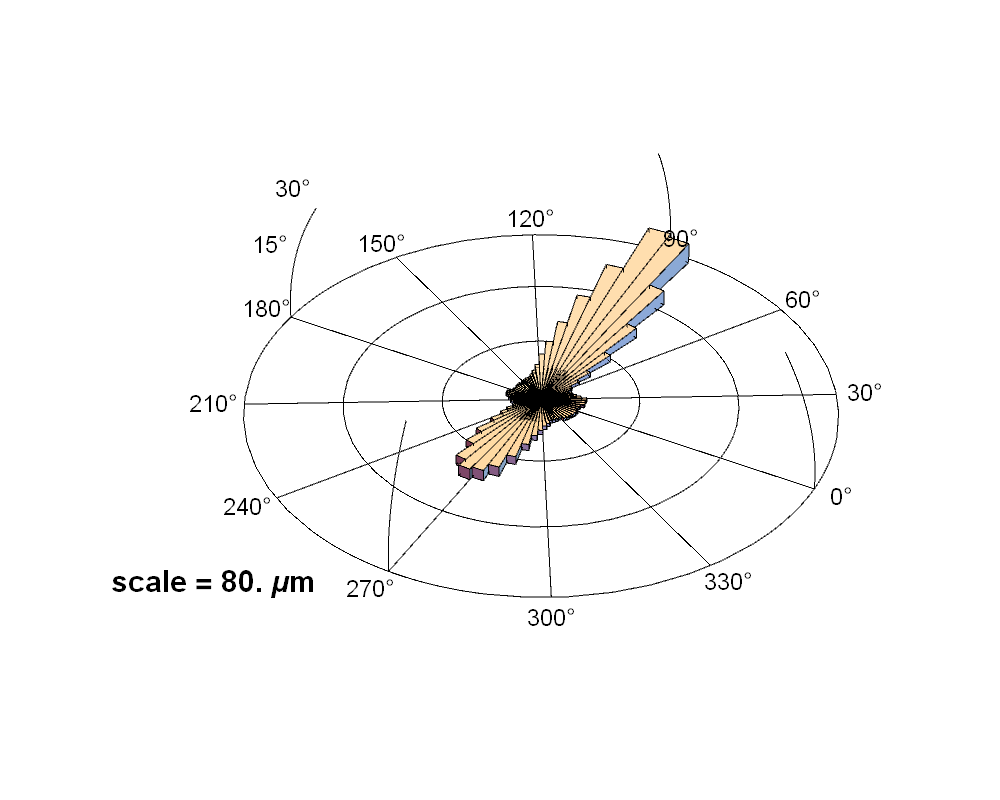

Supplement: Supplementary file 1 [file materials-13-03028-s001.zip › supplementary data/Multiscale curvature analysis/3D rosetta plots/MilledF_40withlabel.tiff]
