# Supplementary figures and images for: Multiscale Characterizations of Surface Anisotropies (part 1 of 2)
Source: Materials (Basel). 2020 Jul 7;13(13):3028. doi: 10.3390/ma13133028 (PMC7372363; doi:10.3390/ma13133028)

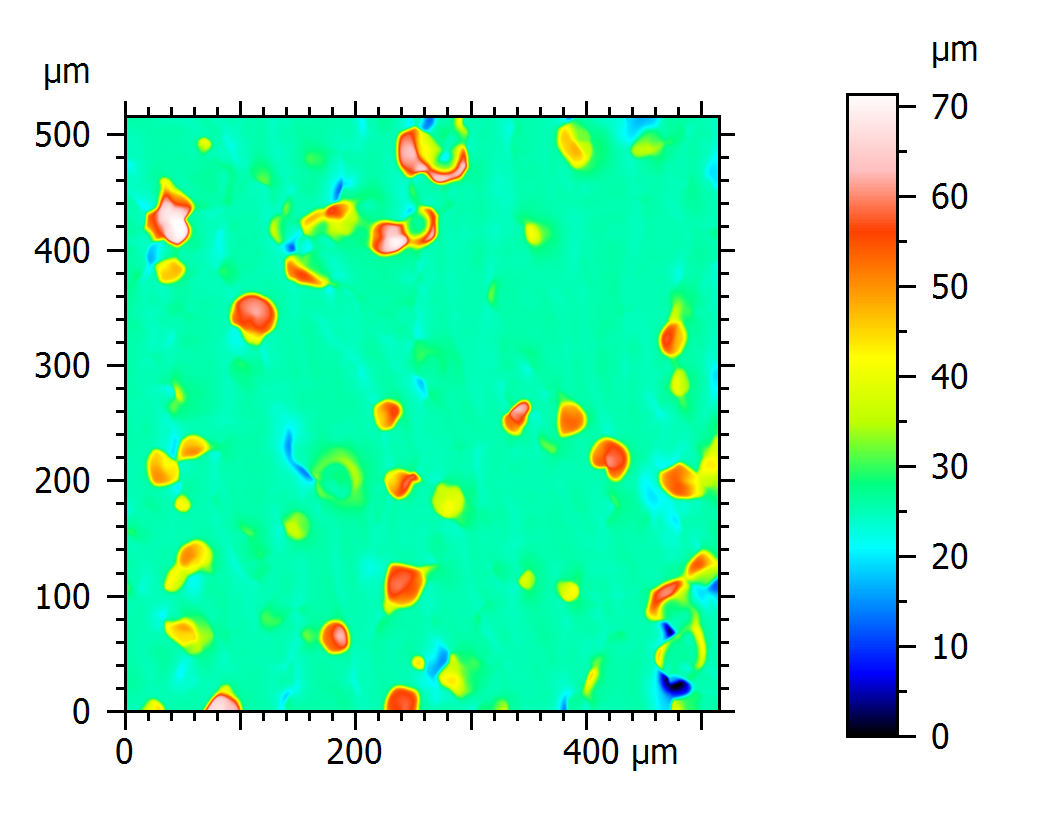

Supplement: Supplementary file 1 [file materials-13-03028-s001.zip › supplementary data/Bandpass filtering/2d_images_filtered_surfaces/L-PBFed_Number=10_CentralWavelength=24.png]

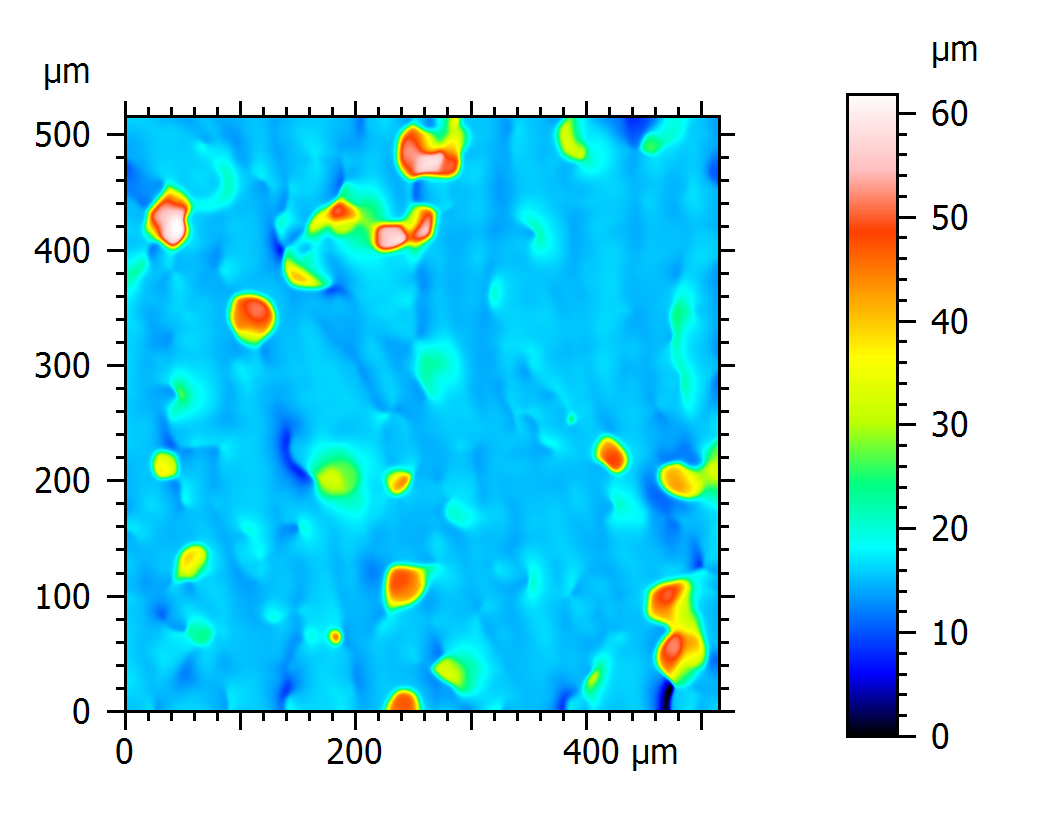

Supplement: Supplementary file 1 [file materials-13-03028-s001.zip › supplementary data/Bandpass filtering/2d_images_filtered_surfaces/L-PBFed_Number=11_CentralWavelength=36.png]

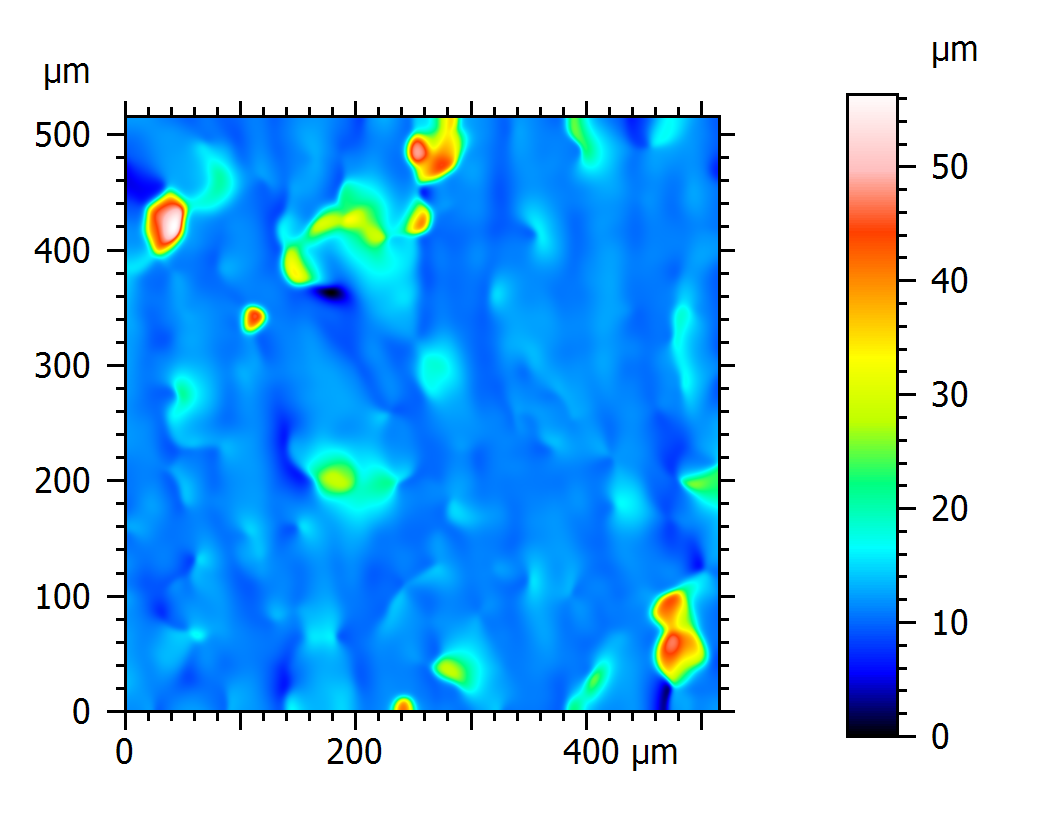

Supplement: Supplementary file 1 [file materials-13-03028-s001.zip › supplementary data/Bandpass filtering/2d_images_filtered_surfaces/L-PBFed_Number=12_CentralWavelength=48.png]

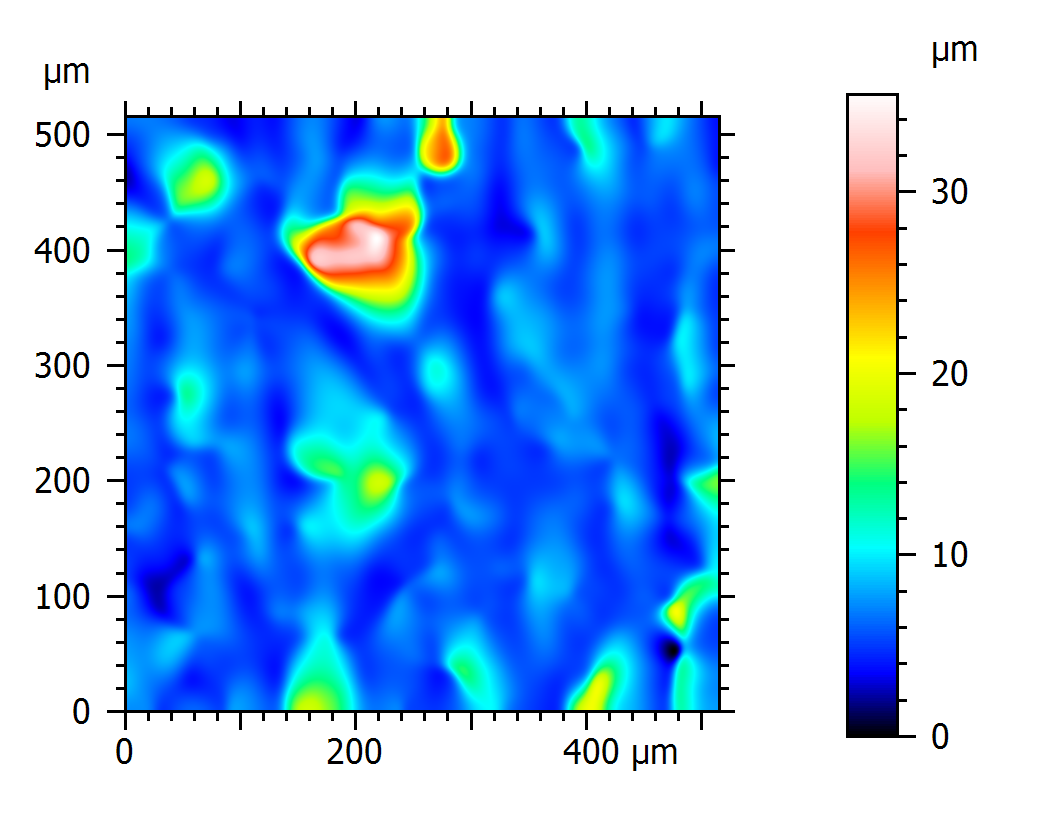

Supplement: Supplementary file 1 [file materials-13-03028-s001.zip › supplementary data/Bandpass filtering/2d_images_filtered_surfaces/L-PBFed_Number=13_CentralWavelength=72.png]

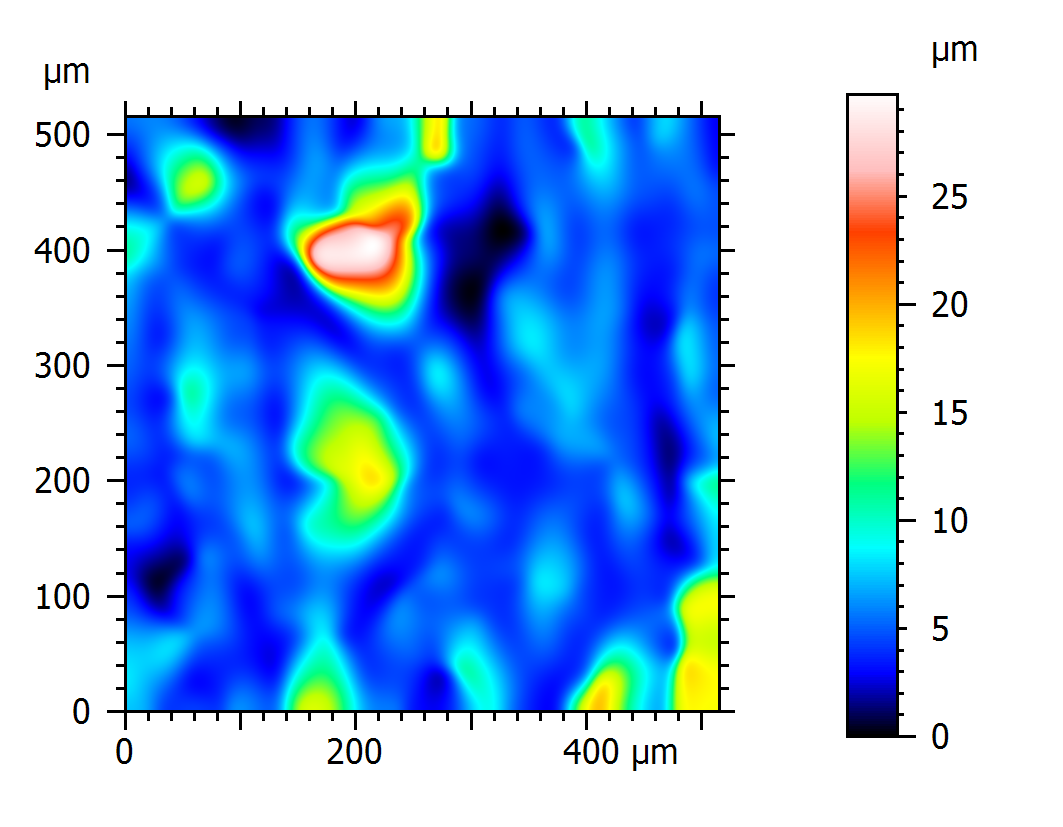

Supplement: Supplementary file 1 [file materials-13-03028-s001.zip › supplementary data/Bandpass filtering/2d_images_filtered_surfaces/L-PBFed_Number=14_CentralWavelength=96.png]

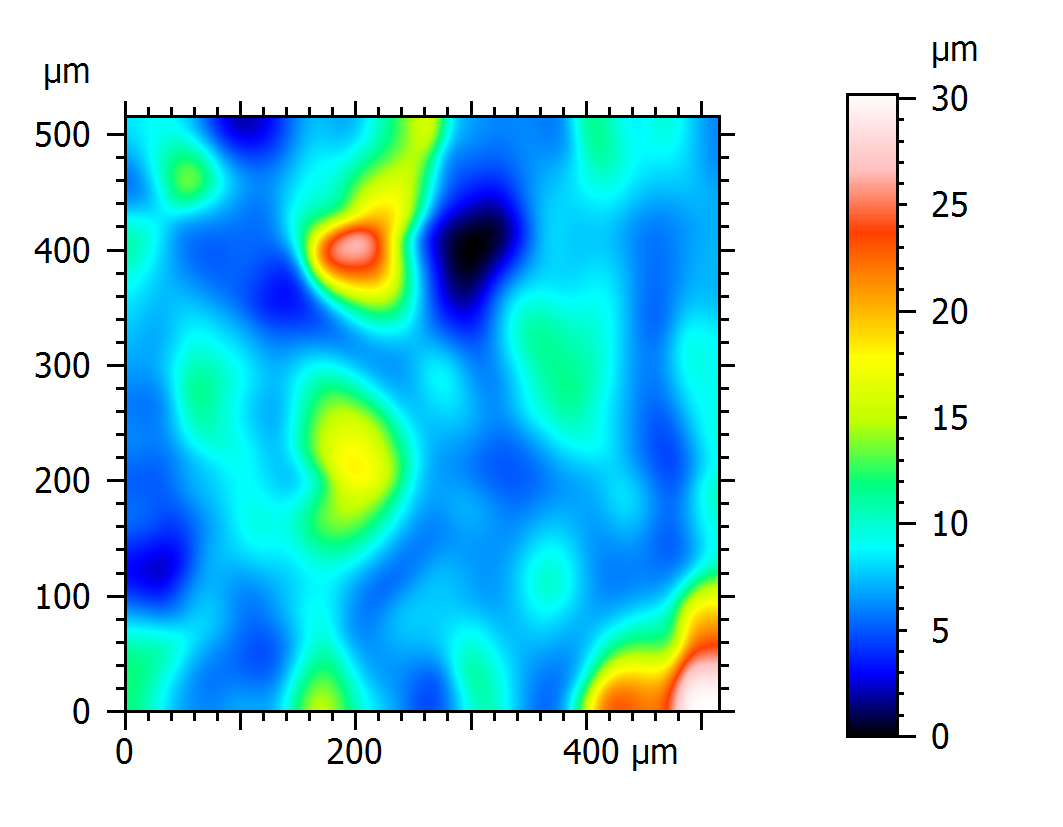

Supplement: Supplementary file 1 [file materials-13-03028-s001.zip › supplementary data/Bandpass filtering/2d_images_filtered_surfaces/L-PBFed_Number=15_CentralWavelength=144.png]

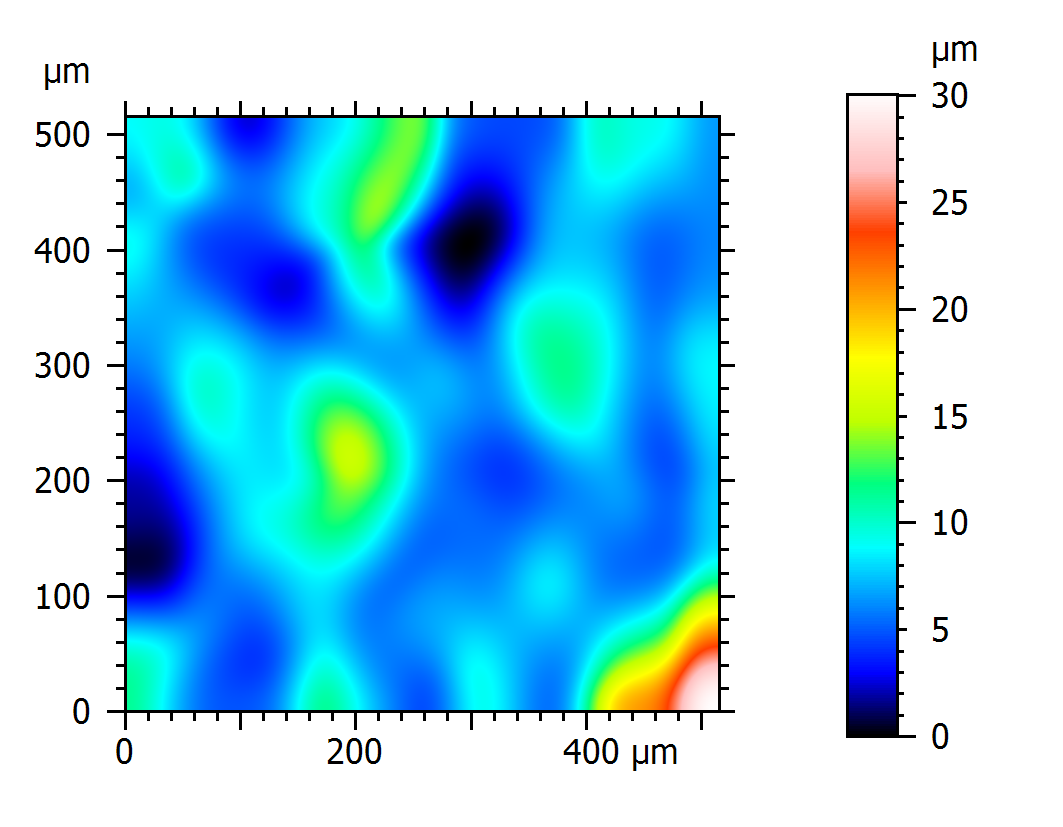

Supplement: Supplementary file 1 [file materials-13-03028-s001.zip › supplementary data/Bandpass filtering/2d_images_filtered_surfaces/L-PBFed_Number=16_CentralWavelength=192.png]

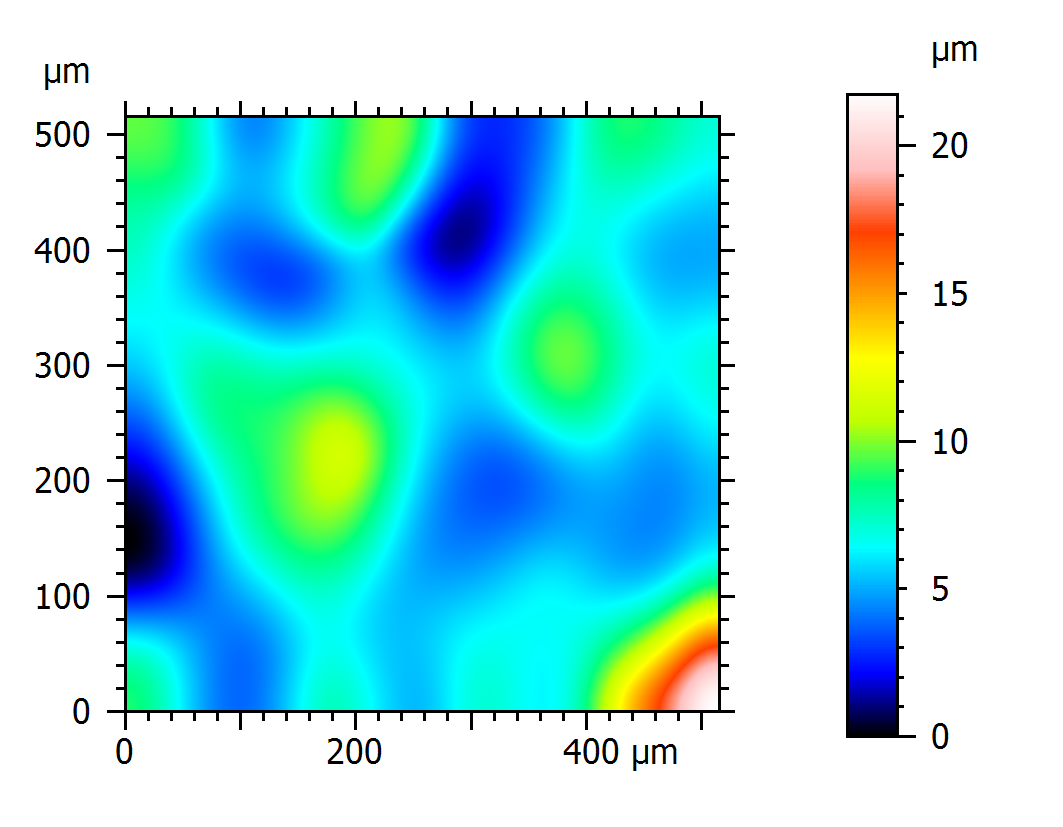

Supplement: Supplementary file 1 [file materials-13-03028-s001.zip › supplementary data/Bandpass filtering/2d_images_filtered_surfaces/L-PBFed_Number=17_CentralWavelength=288.png]

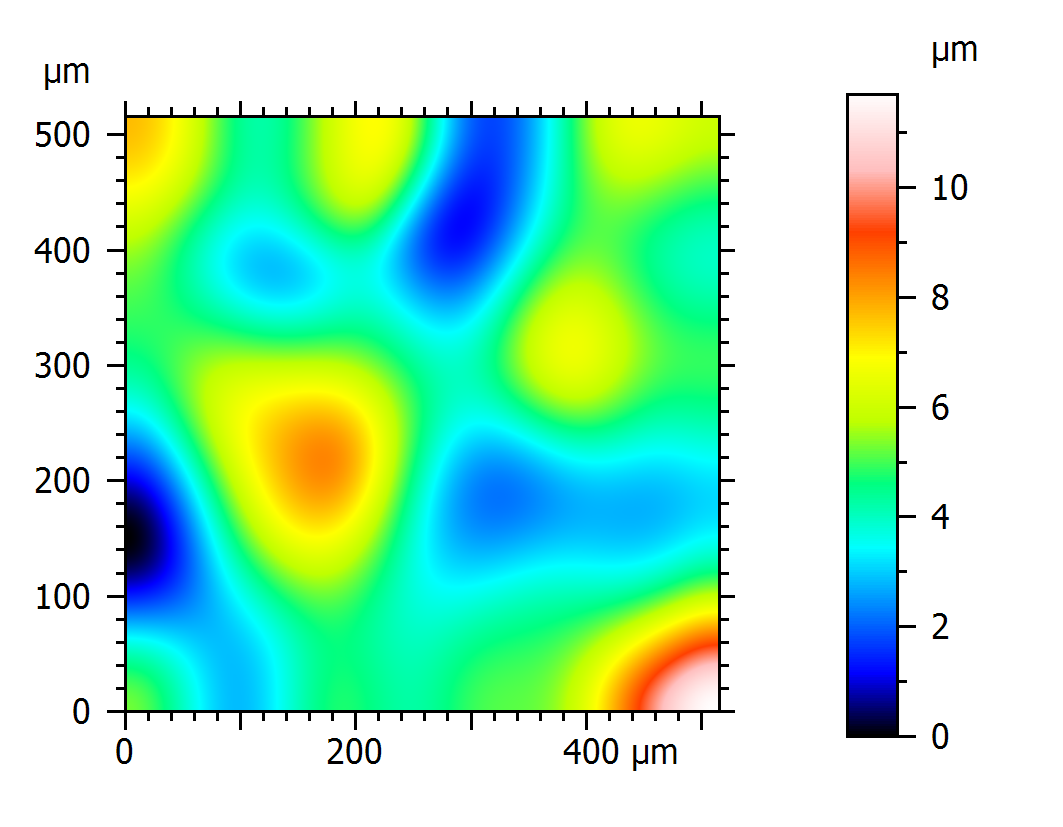

Supplement: Supplementary file 1 [file materials-13-03028-s001.zip › supplementary data/Bandpass filtering/2d_images_filtered_surfaces/L-PBFed_Number=18_CentralWavelength=384.png]

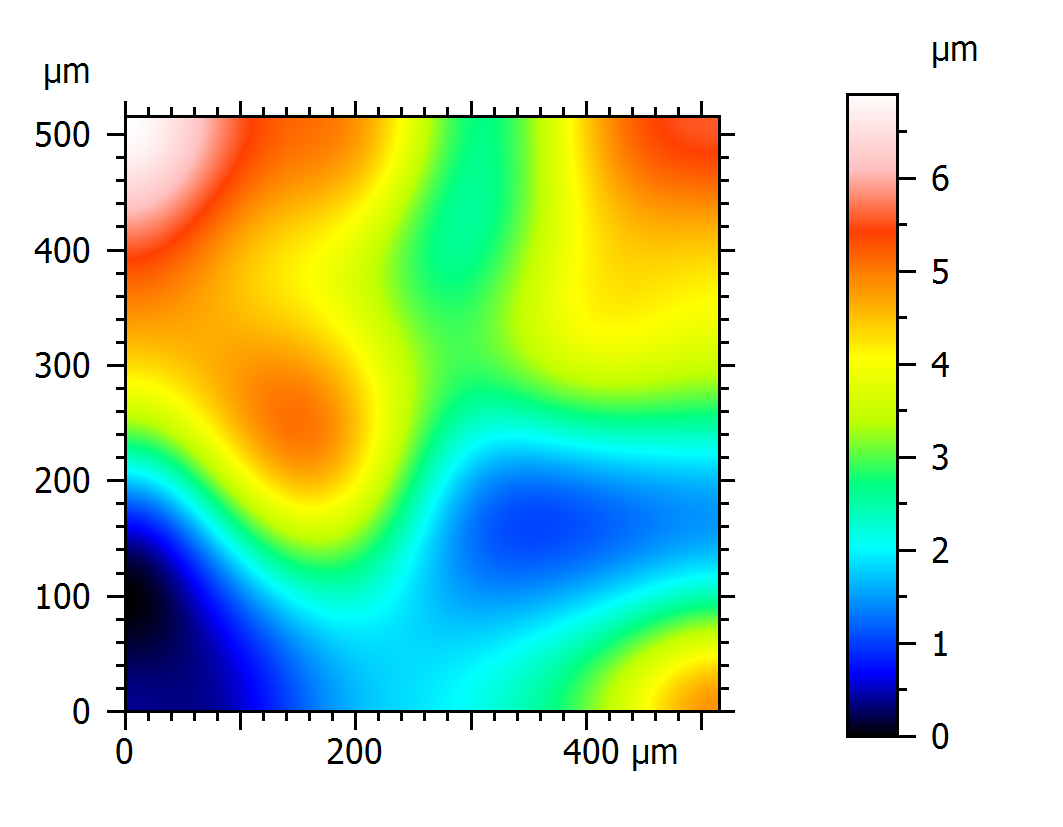

Supplement: Supplementary file 1 [file materials-13-03028-s001.zip › supplementary data/Bandpass filtering/2d_images_filtered_surfaces/L-PBFed_Number=19_CentralWavelength=576.png]

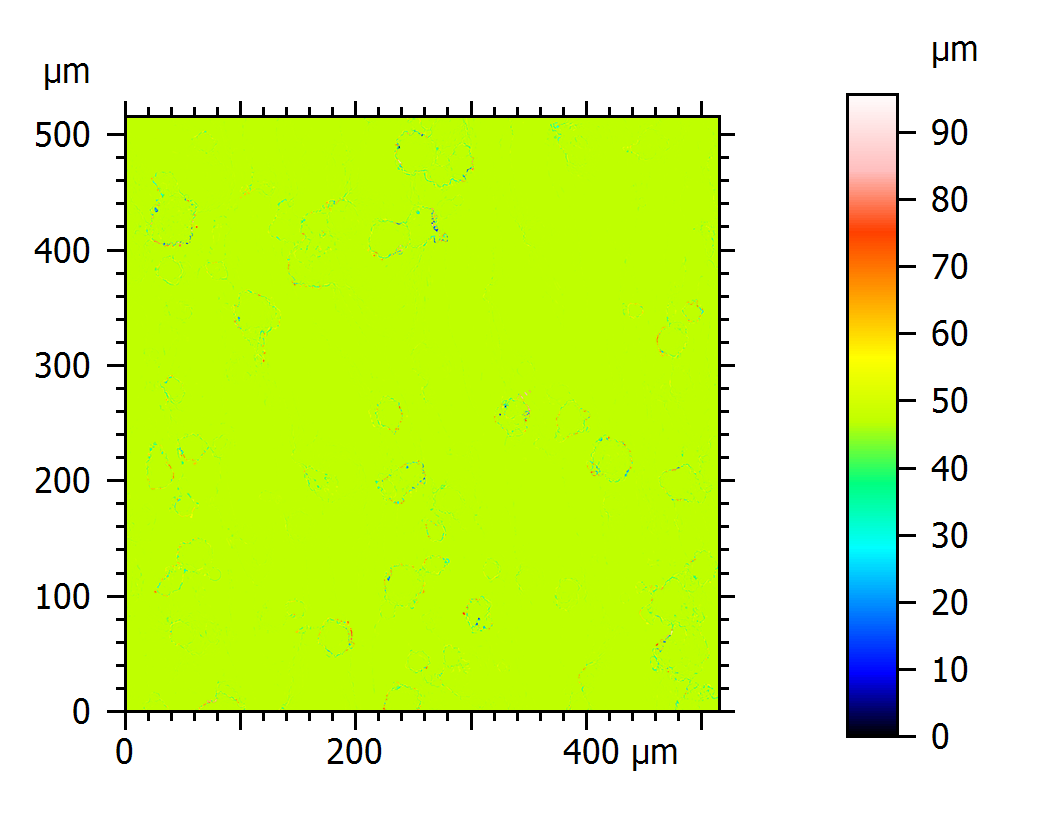

Supplement: Supplementary file 1 [file materials-13-03028-s001.zip › supplementary data/Bandpass filtering/2d_images_filtered_surfaces/L-PBFed_Number=1_CentralWavelength=1.125.png]

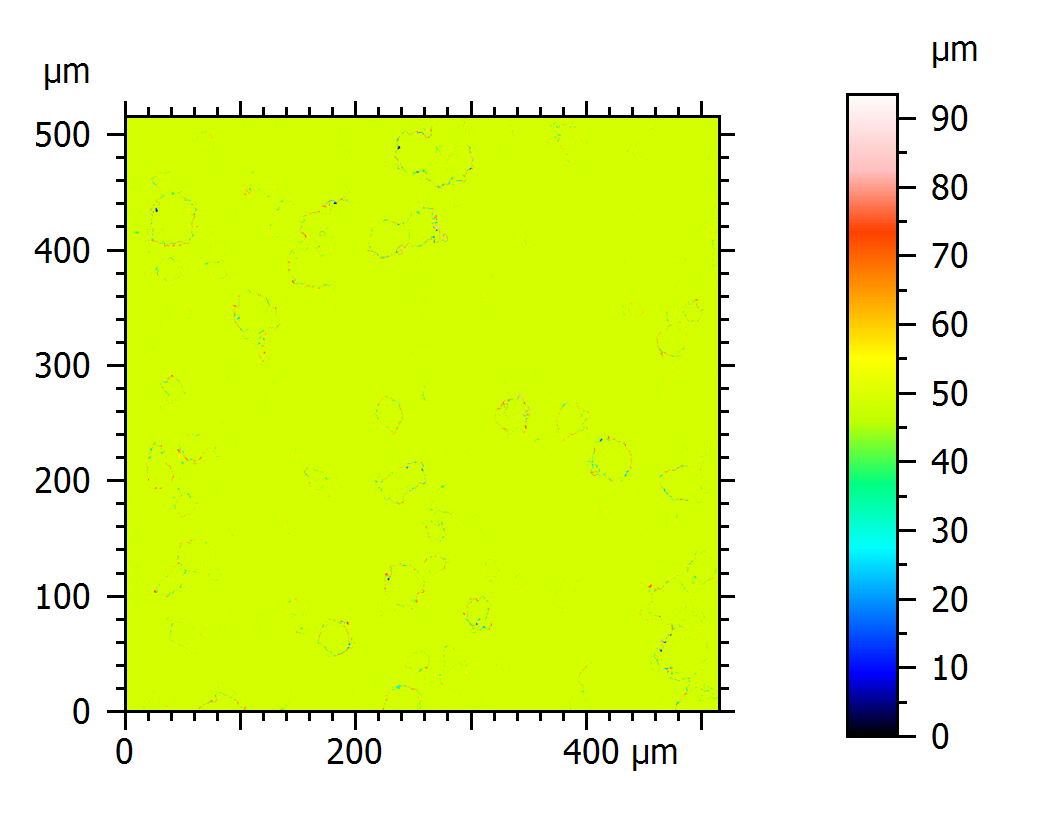

Supplement: Supplementary file 1 [file materials-13-03028-s001.zip › supplementary data/Bandpass filtering/2d_images_filtered_surfaces/L-PBFed_Number=2_CentralWavelength=1.5.png]

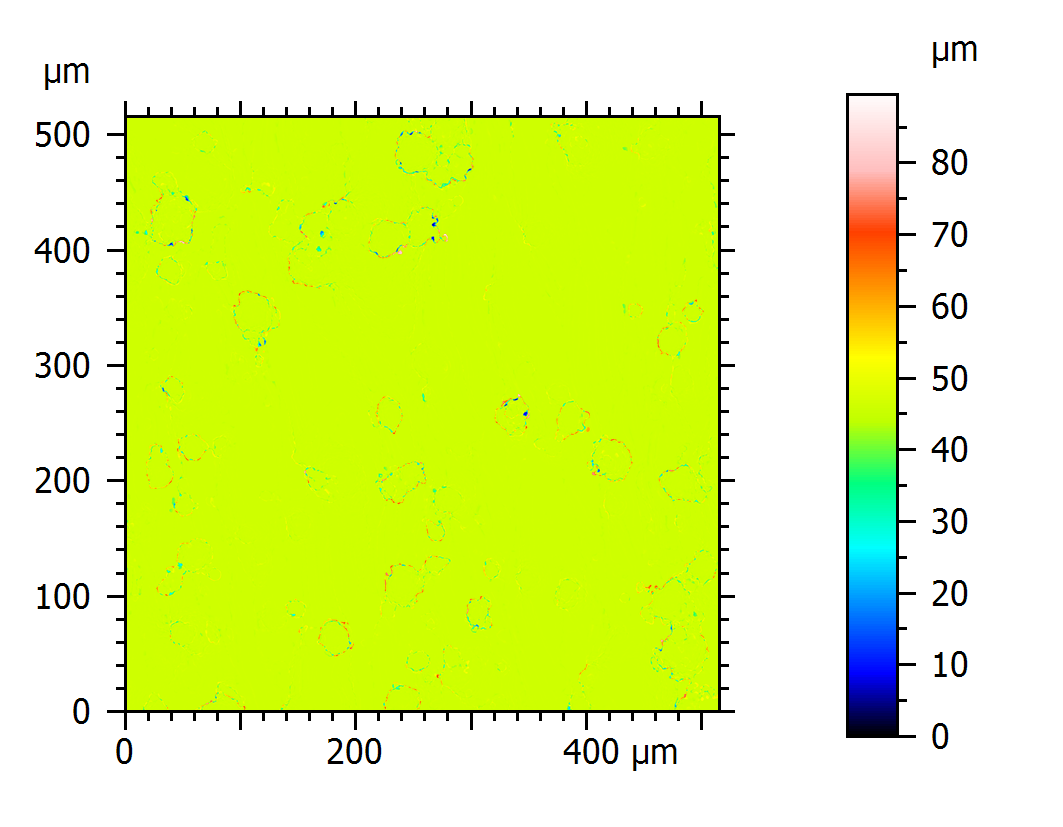

Supplement: Supplementary file 1 [file materials-13-03028-s001.zip › supplementary data/Bandpass filtering/2d_images_filtered_surfaces/L-PBFed_Number=3_CentralWavelength=2.25.png]

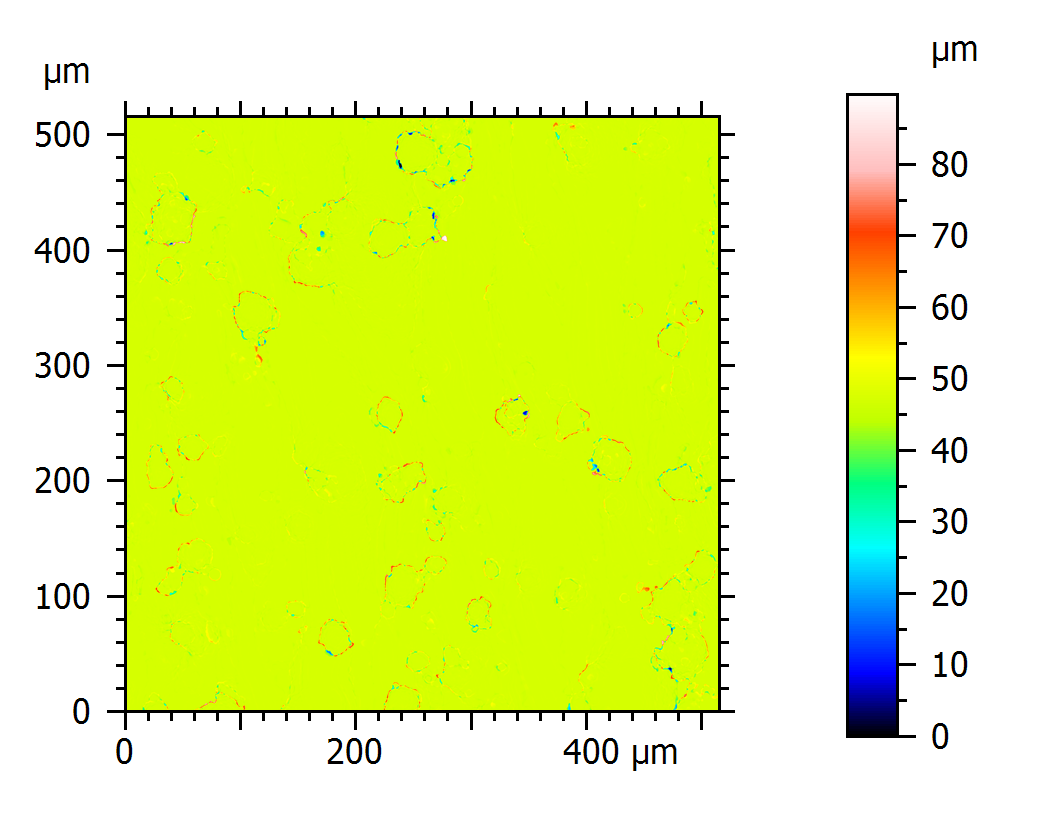

Supplement: Supplementary file 1 [file materials-13-03028-s001.zip › supplementary data/Bandpass filtering/2d_images_filtered_surfaces/L-PBFed_Number=4_CentralWavelength=3.png]

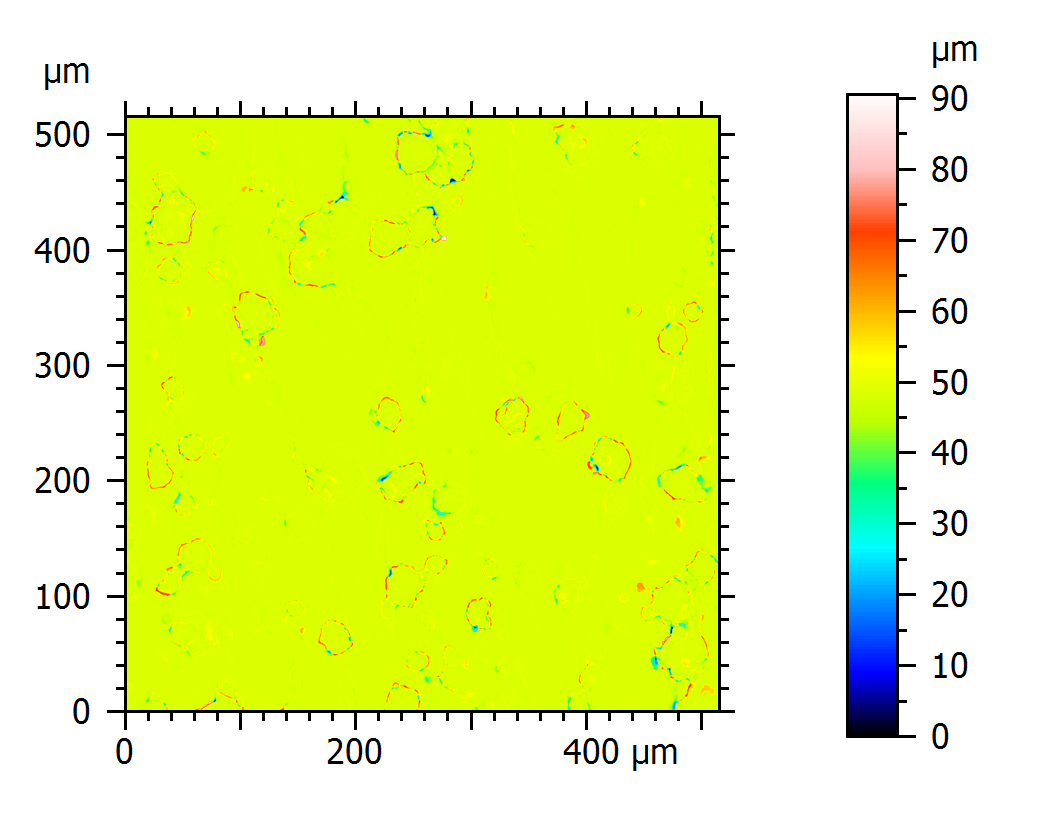

Supplement: Supplementary file 1 [file materials-13-03028-s001.zip › supplementary data/Bandpass filtering/2d_images_filtered_surfaces/L-PBFed_Number=5_CentralWavelength=4.5.png]

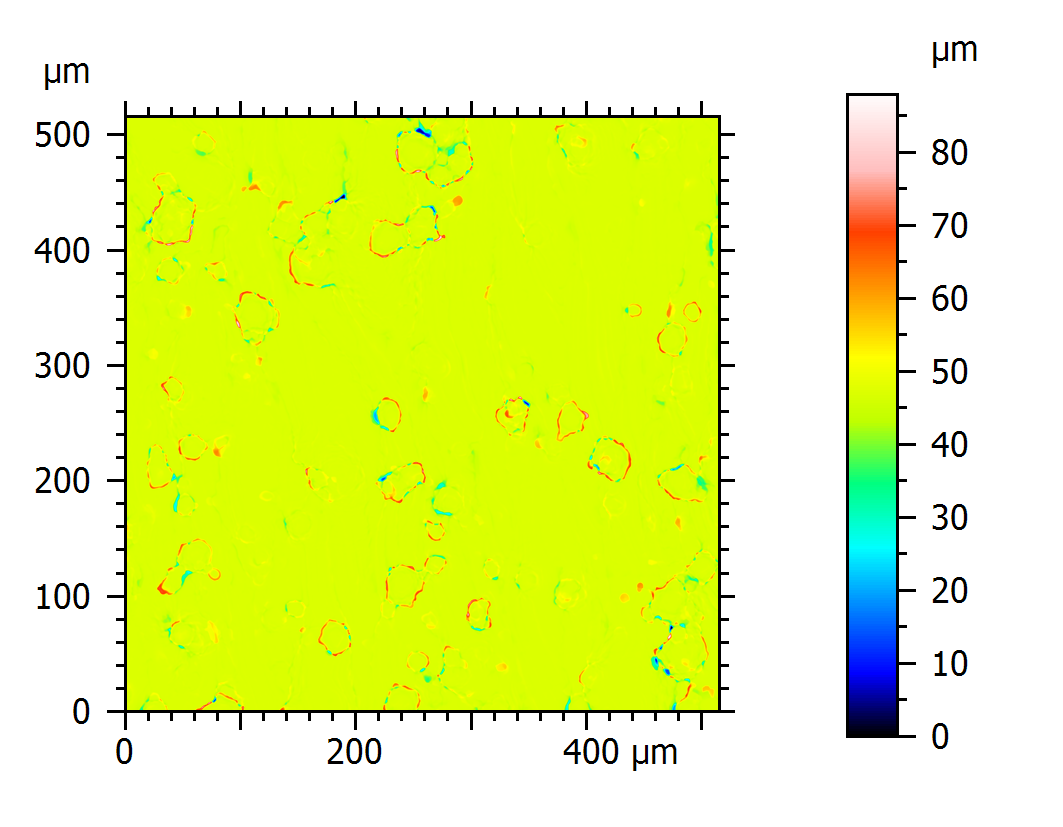

Supplement: Supplementary file 1 [file materials-13-03028-s001.zip › supplementary data/Bandpass filtering/2d_images_filtered_surfaces/L-PBFed_Number=6_CentralWavelength=6.png]

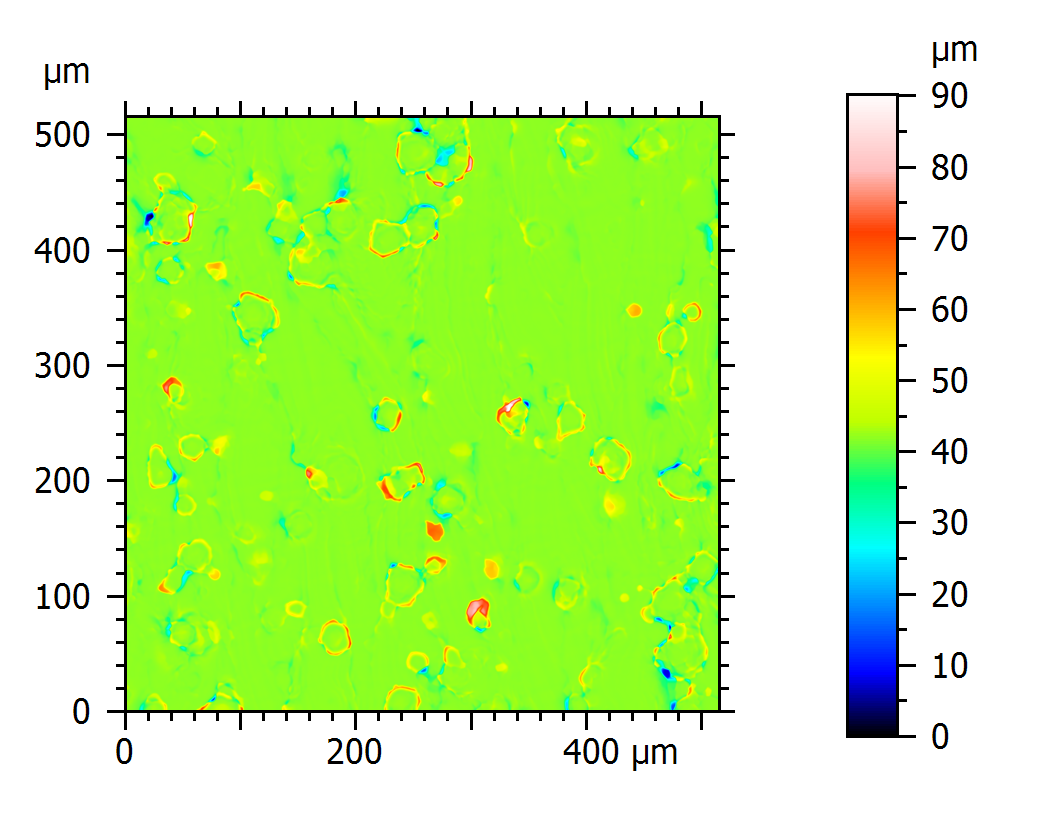

Supplement: Supplementary file 1 [file materials-13-03028-s001.zip › supplementary data/Bandpass filtering/2d_images_filtered_surfaces/L-PBFed_Number=7_CentralWavelength=9.png]

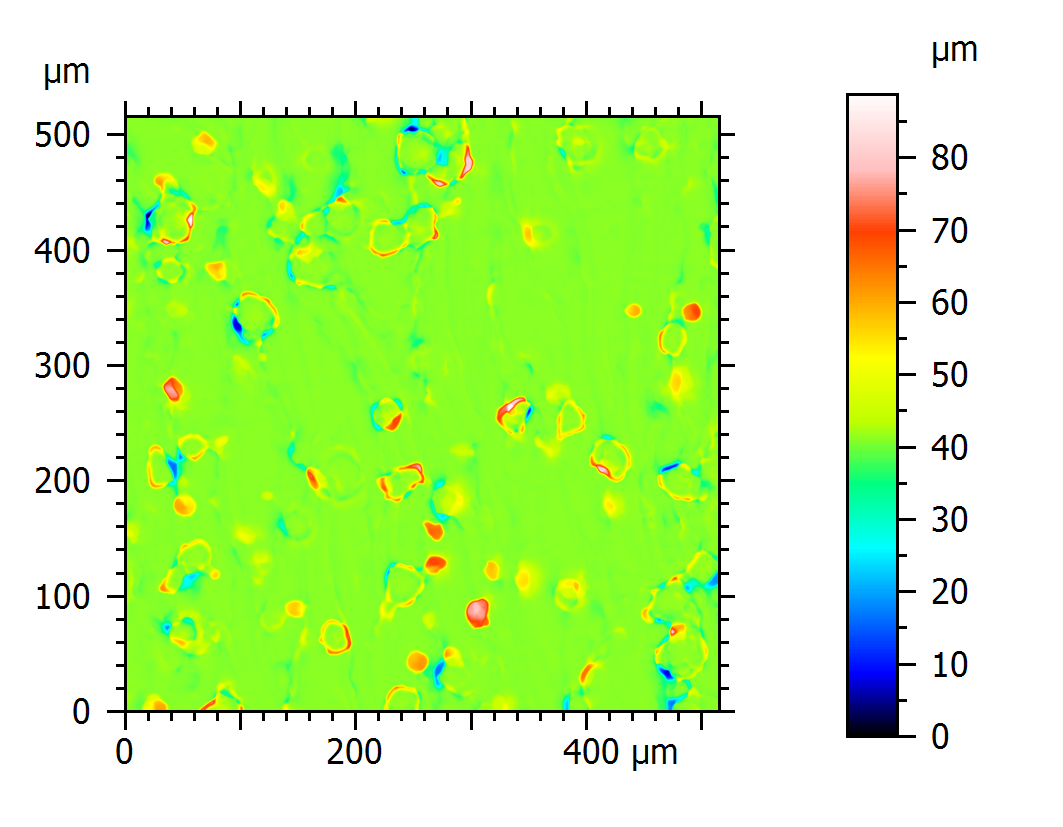

Supplement: Supplementary file 1 [file materials-13-03028-s001.zip › supplementary data/Bandpass filtering/2d_images_filtered_surfaces/L-PBFed_Number=8_CentralWavelength=12.png]

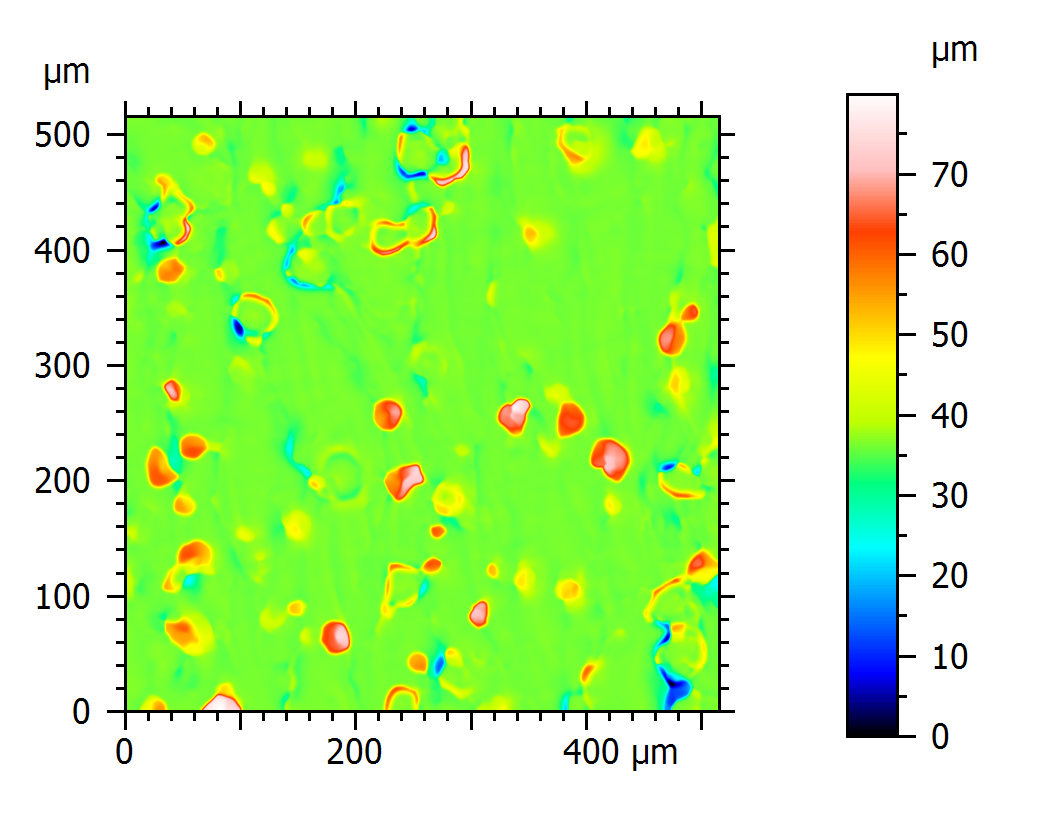

Supplement: Supplementary file 1 [file materials-13-03028-s001.zip › supplementary data/Bandpass filtering/2d_images_filtered_surfaces/L-PBFed_Number=9_CentralWavelength=18.png]

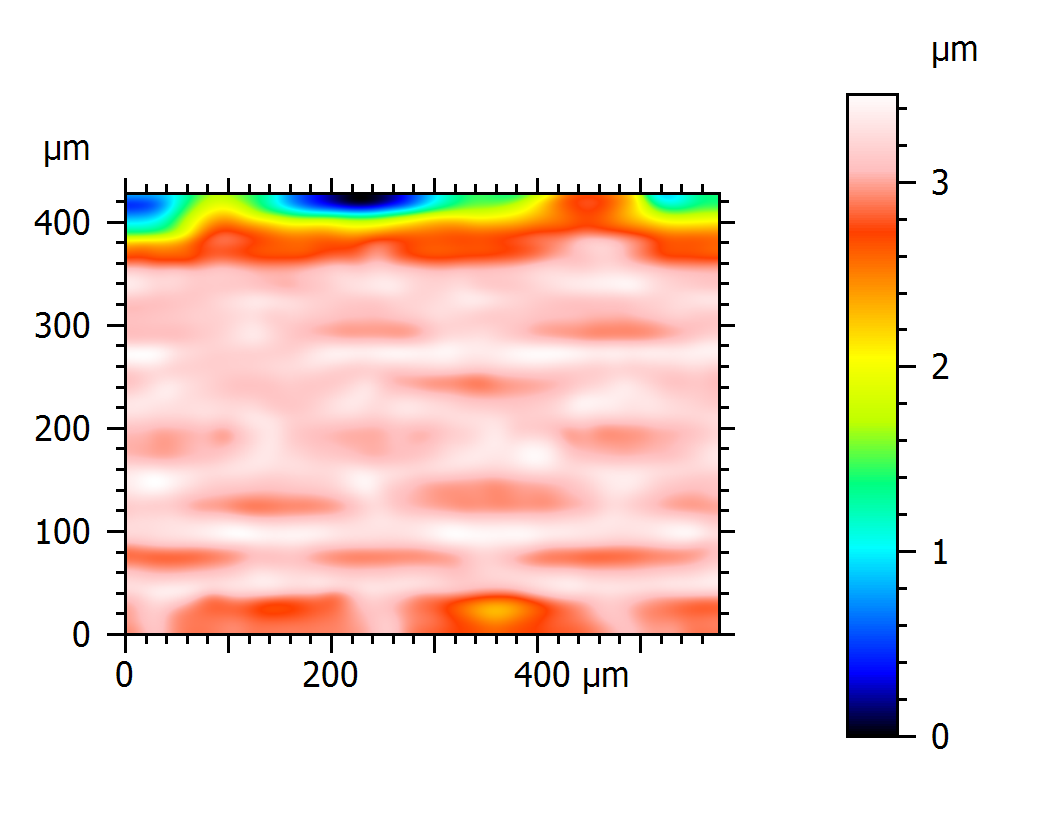

Supplement: Supplementary file 1 [file materials-13-03028-s001.zip › supplementary data/Bandpass filtering/2d_images_filtered_surfaces/MilledC_Number=10_CentralWavelength=72.png]

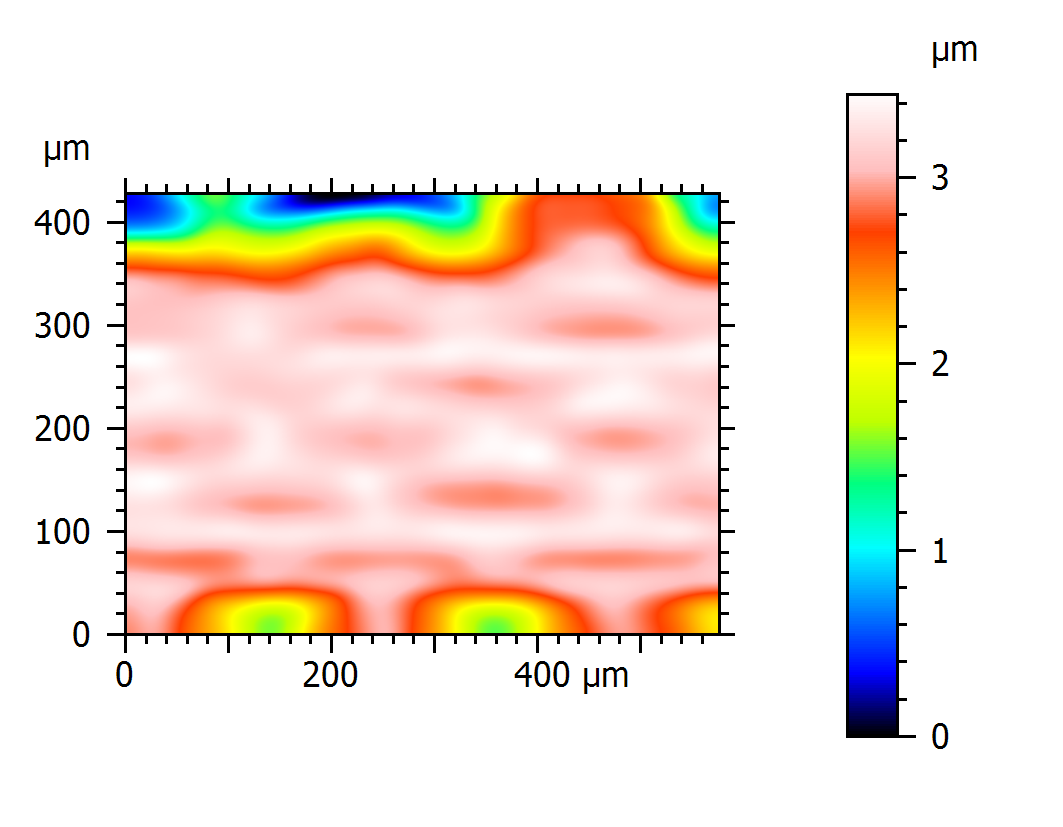

Supplement: Supplementary file 1 [file materials-13-03028-s001.zip › supplementary data/Bandpass filtering/2d_images_filtered_surfaces/MilledC_Number=11_CentralWavelength=96.png]

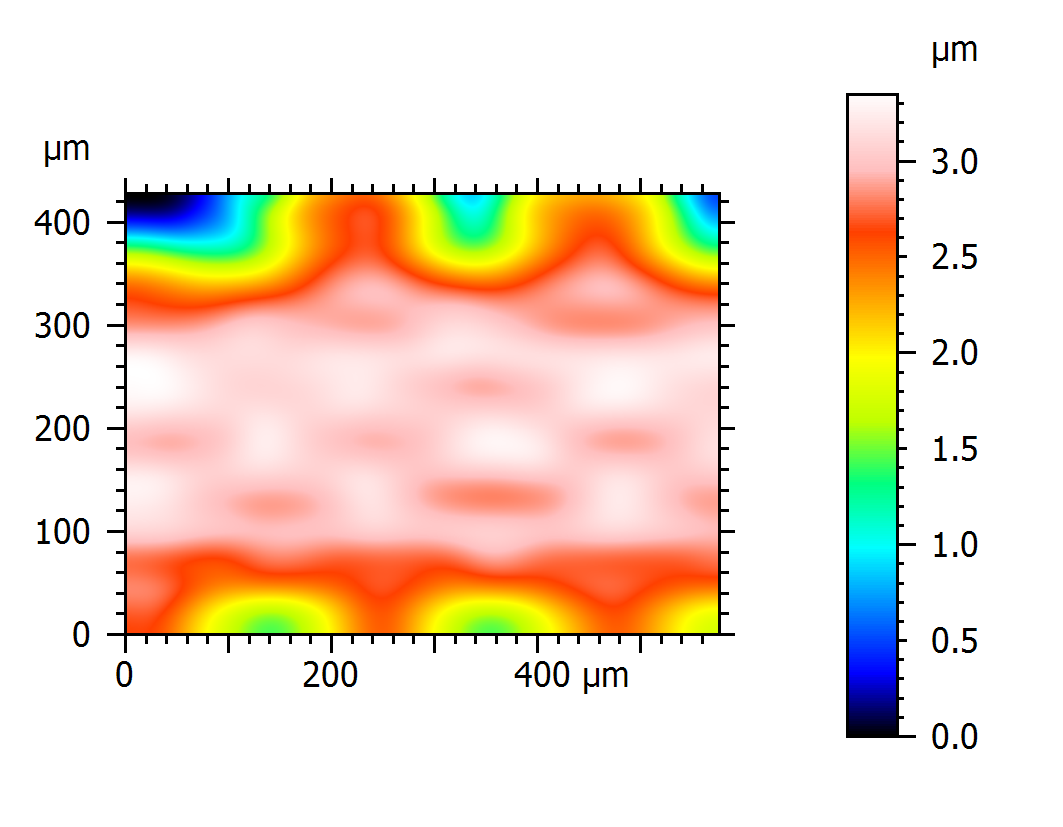

Supplement: Supplementary file 1 [file materials-13-03028-s001.zip › supplementary data/Bandpass filtering/2d_images_filtered_surfaces/MilledC_Number=12_CentralWavelength=144.png]

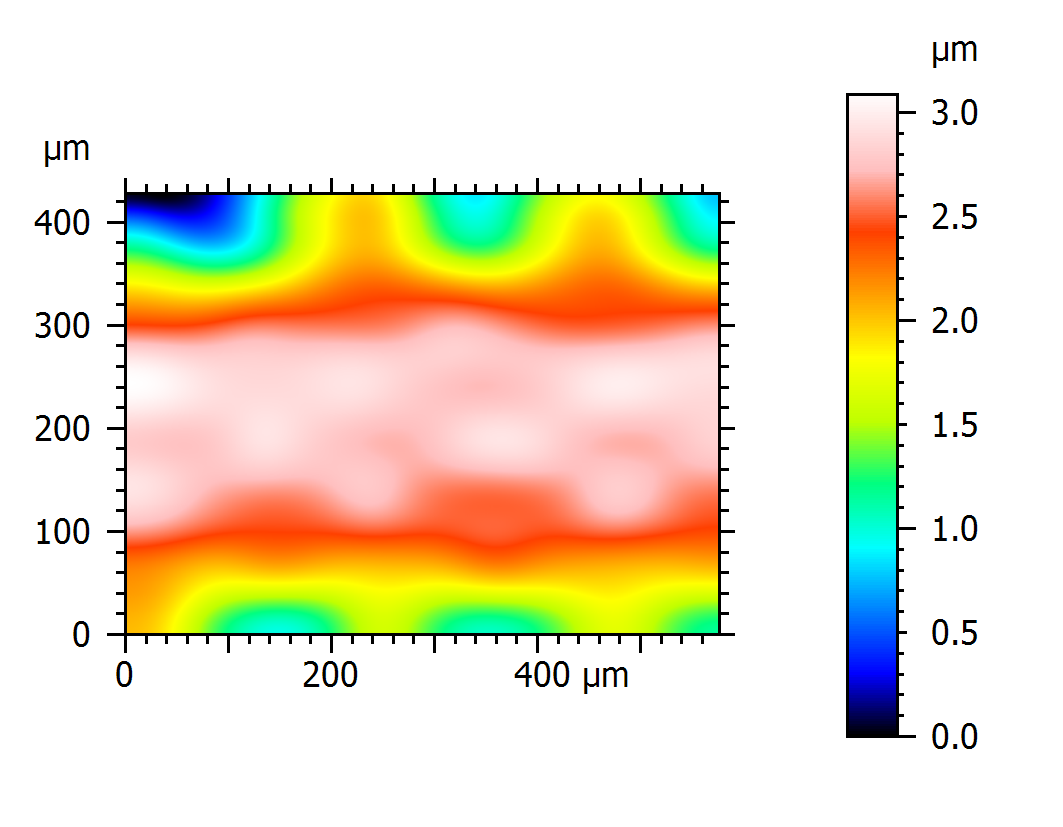

Supplement: Supplementary file 1 [file materials-13-03028-s001.zip › supplementary data/Bandpass filtering/2d_images_filtered_surfaces/MilledC_Number=13_CentralWavelength=192.png]

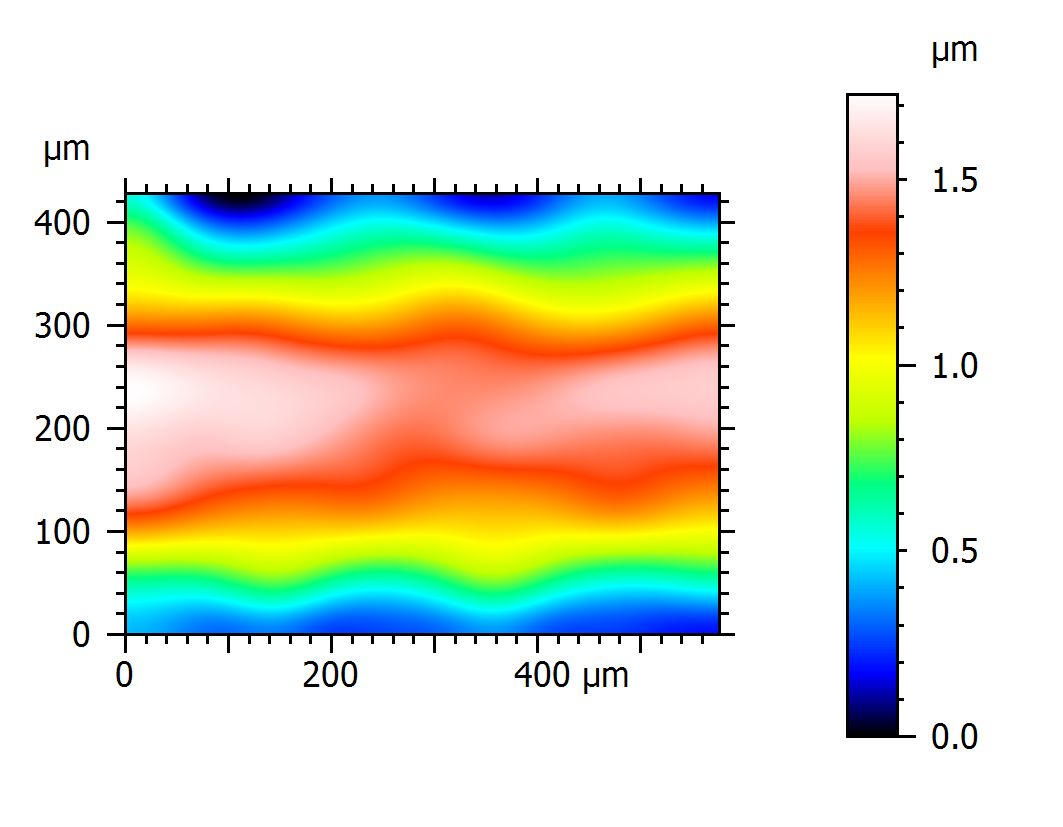

Supplement: Supplementary file 1 [file materials-13-03028-s001.zip › supplementary data/Bandpass filtering/2d_images_filtered_surfaces/MilledC_Number=14_CentralWavelength=270.png]

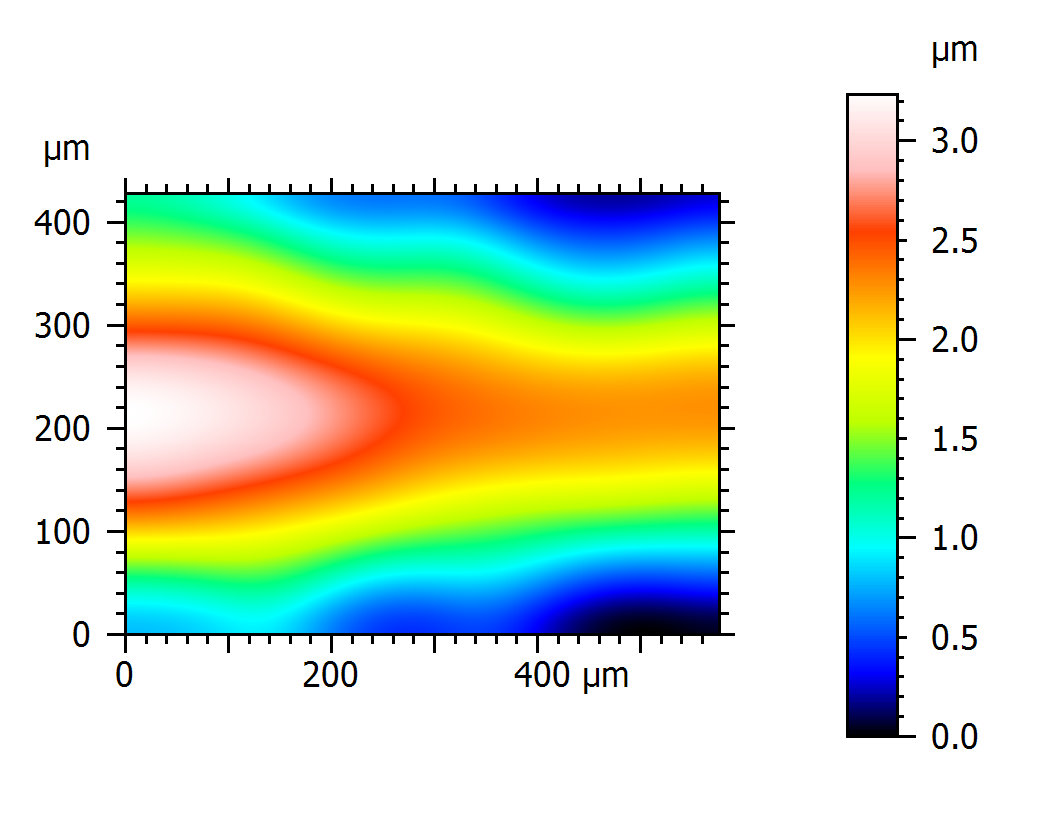

Supplement: Supplementary file 1 [file materials-13-03028-s001.zip › supplementary data/Bandpass filtering/2d_images_filtered_surfaces/MilledC_Number=15_CentralWavelength=384.png]

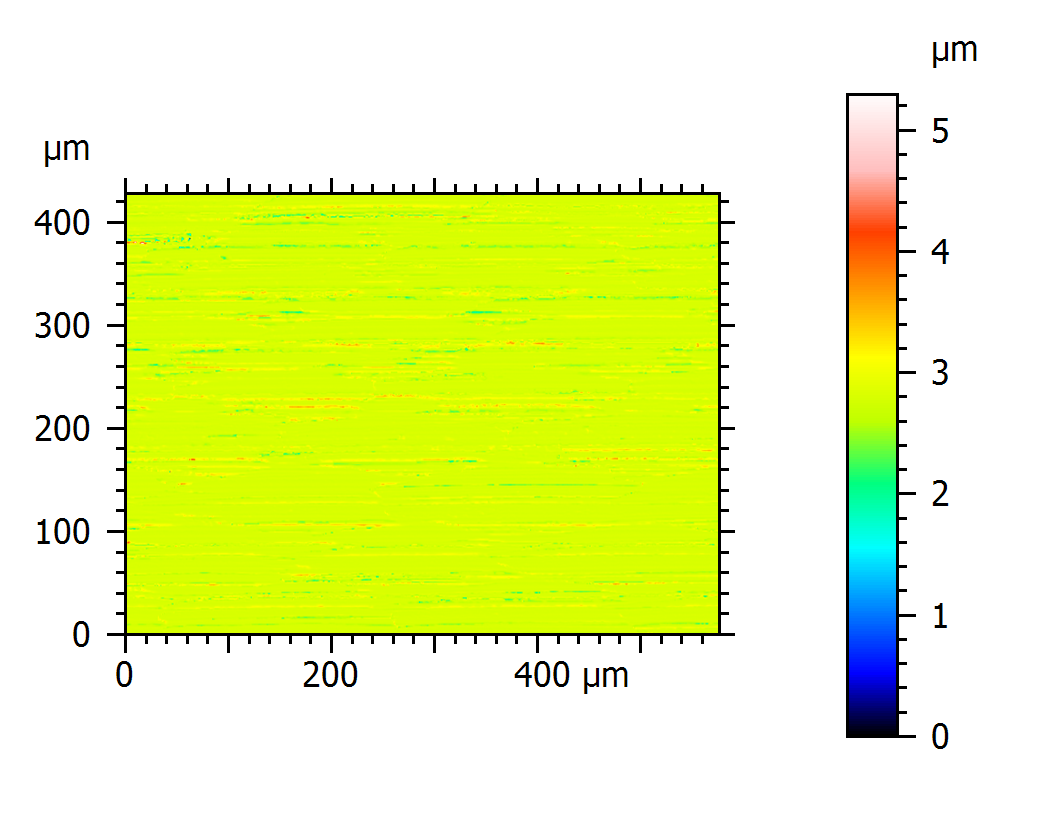

Supplement: Supplementary file 1 [file materials-13-03028-s001.zip › supplementary data/Bandpass filtering/2d_images_filtered_surfaces/MilledC_Number=1_CentralWavelength=3.png]

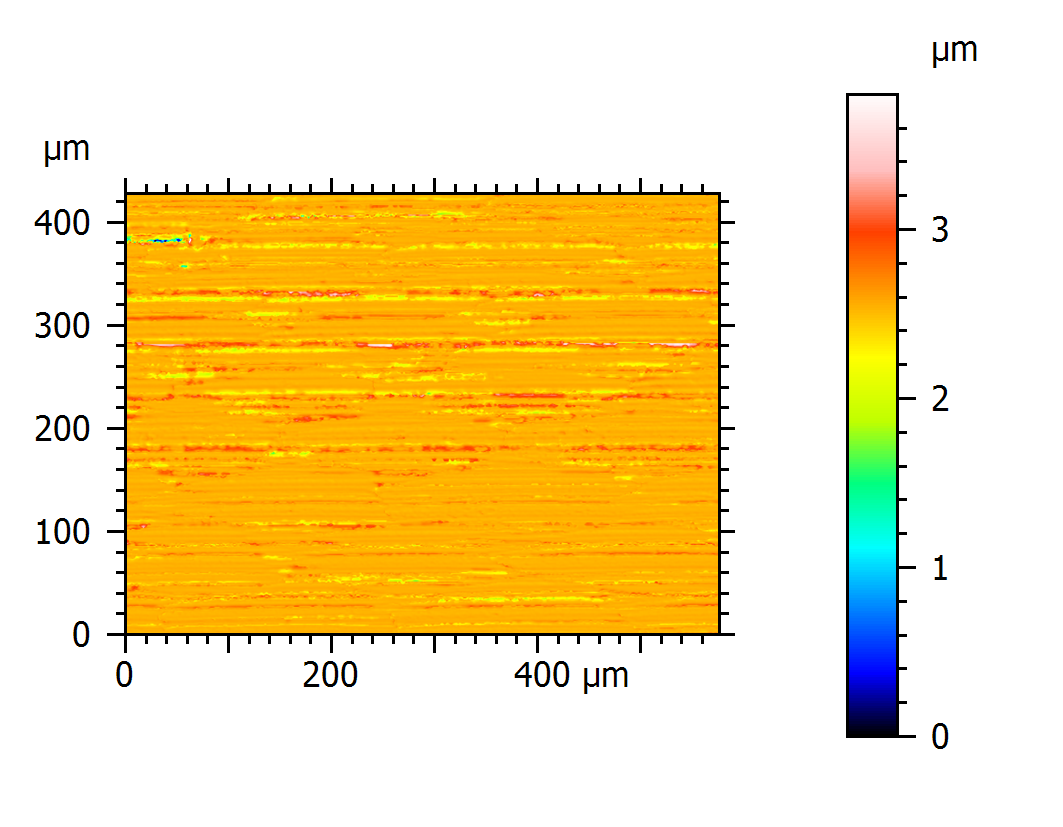

Supplement: Supplementary file 1 [file materials-13-03028-s001.zip › supplementary data/Bandpass filtering/2d_images_filtered_surfaces/MilledC_Number=2_CentralWavelength=4.5.png]

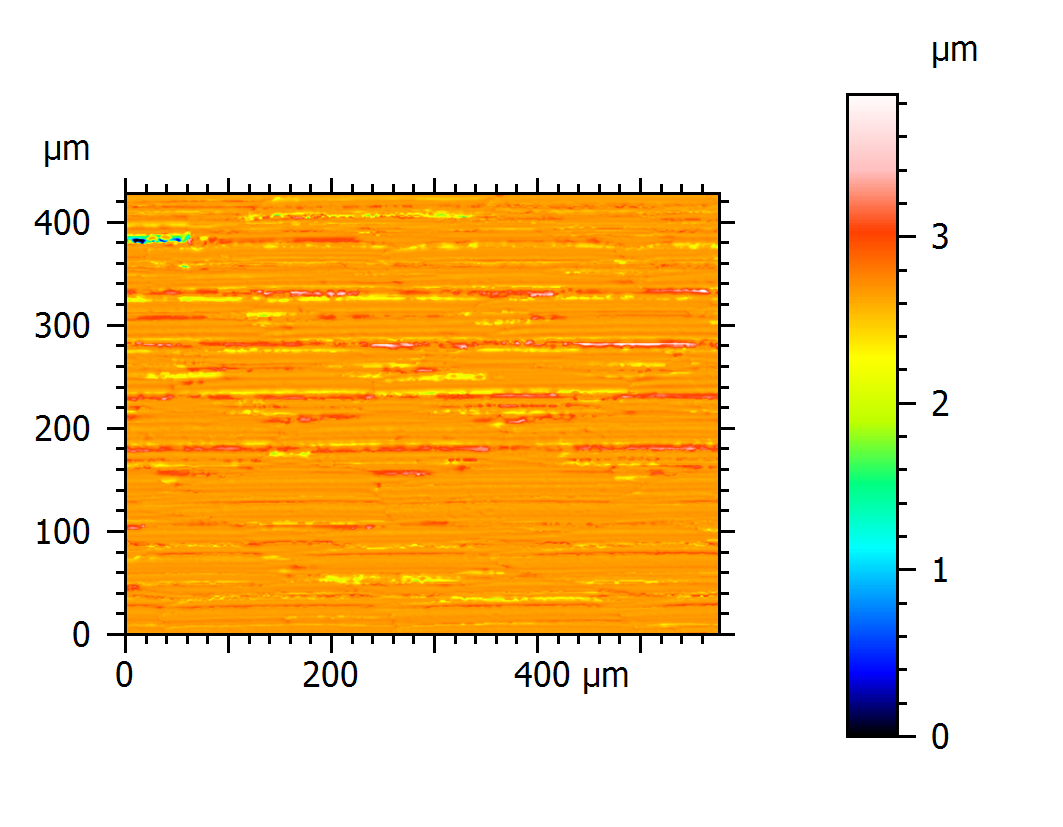

Supplement: Supplementary file 1 [file materials-13-03028-s001.zip › supplementary data/Bandpass filtering/2d_images_filtered_surfaces/MilledC_Number=3_CentralWavelength=6.png]

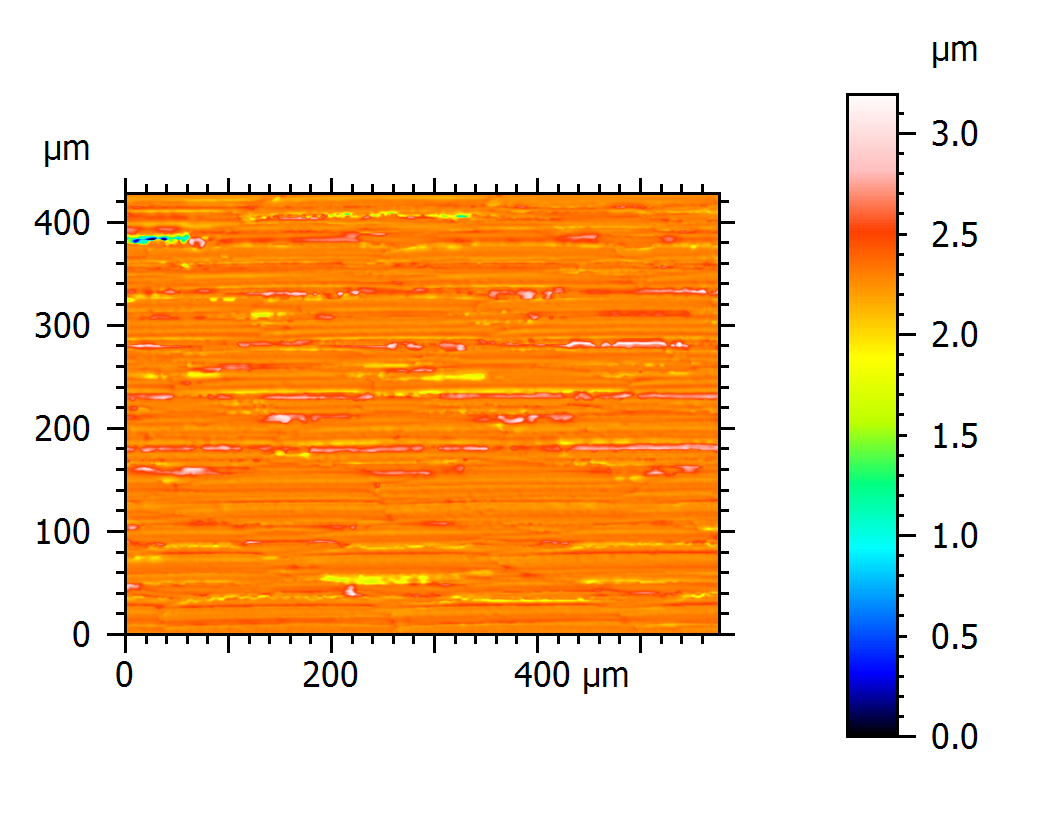

Supplement: Supplementary file 1 [file materials-13-03028-s001.zip › supplementary data/Bandpass filtering/2d_images_filtered_surfaces/MilledC_Number=4_CentralWavelength=9.png]

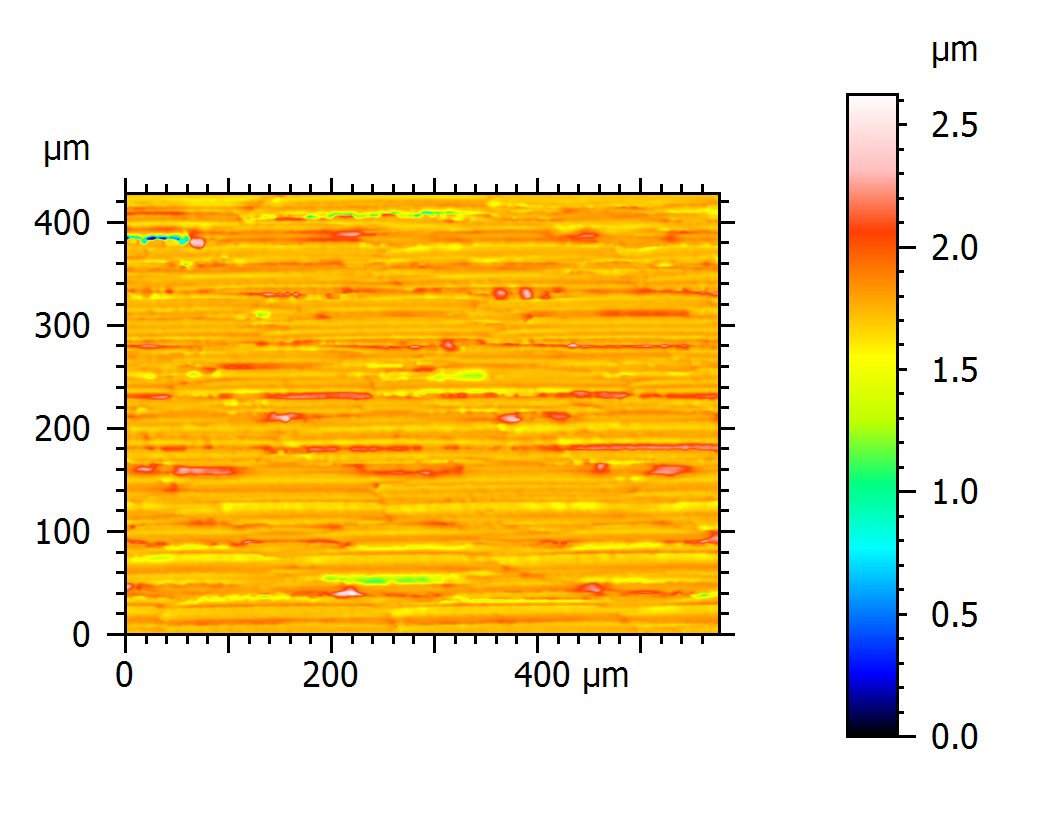

Supplement: Supplementary file 1 [file materials-13-03028-s001.zip › supplementary data/Bandpass filtering/2d_images_filtered_surfaces/MilledC_Number=5_CentralWavelength=12.png]

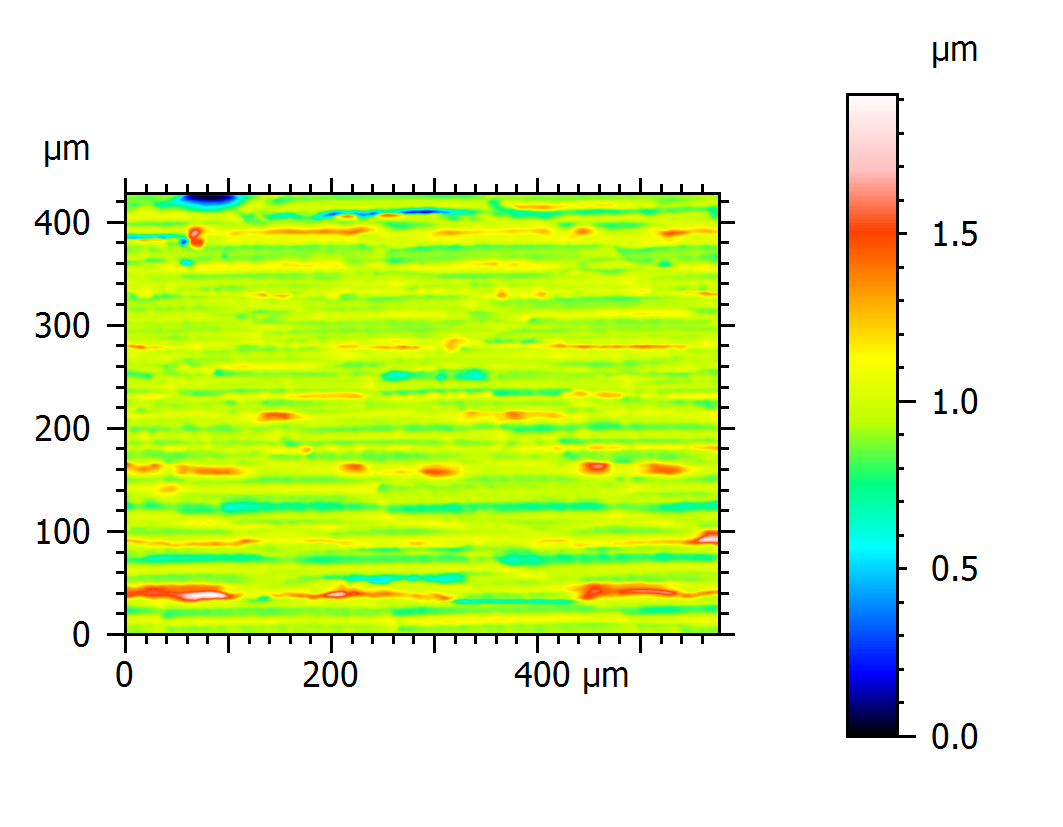

Supplement: Supplementary file 1 [file materials-13-03028-s001.zip › supplementary data/Bandpass filtering/2d_images_filtered_surfaces/MilledC_Number=6_CentralWavelength=18.png]

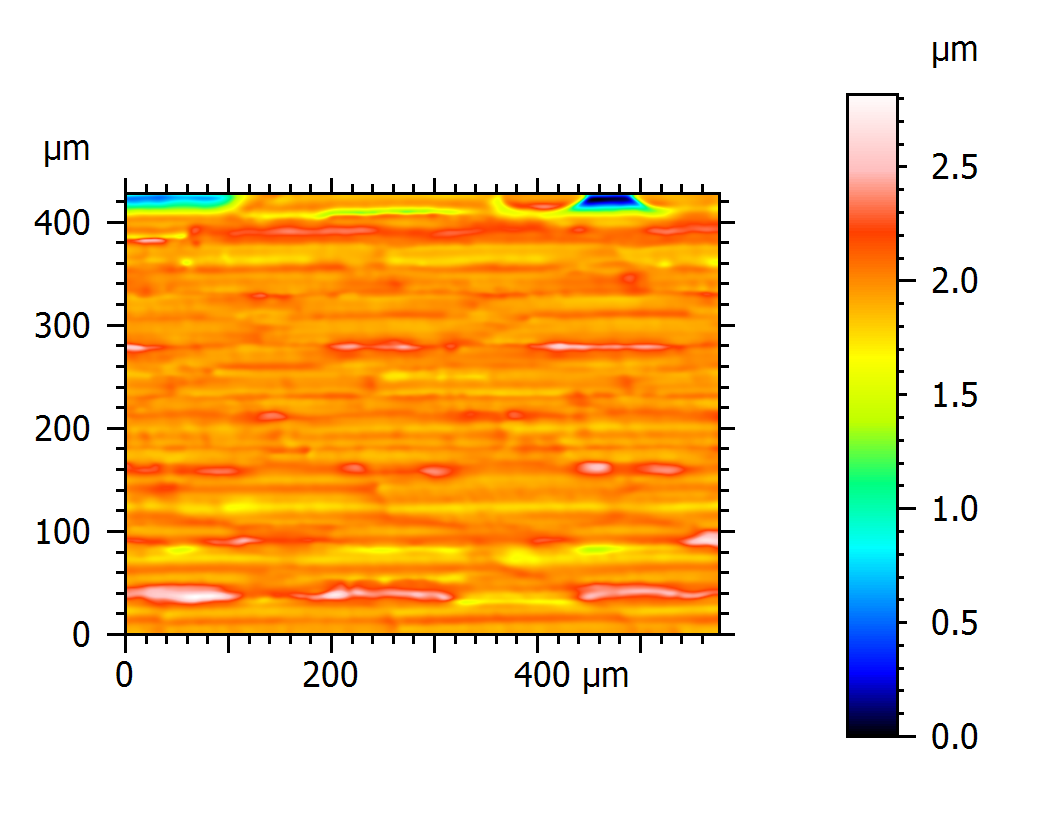

Supplement: Supplementary file 1 [file materials-13-03028-s001.zip › supplementary data/Bandpass filtering/2d_images_filtered_surfaces/MilledC_Number=7_CentralWavelength=24.png]

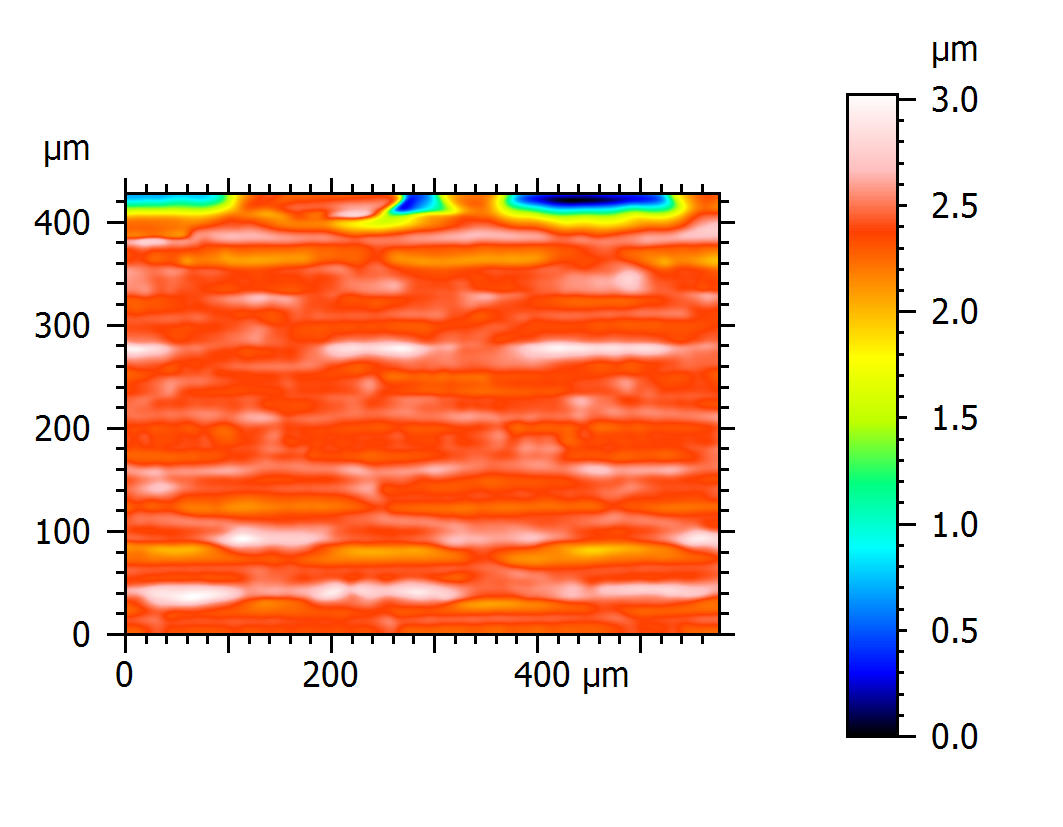

Supplement: Supplementary file 1 [file materials-13-03028-s001.zip › supplementary data/Bandpass filtering/2d_images_filtered_surfaces/MilledC_Number=8_CentralWavelength=36.png]

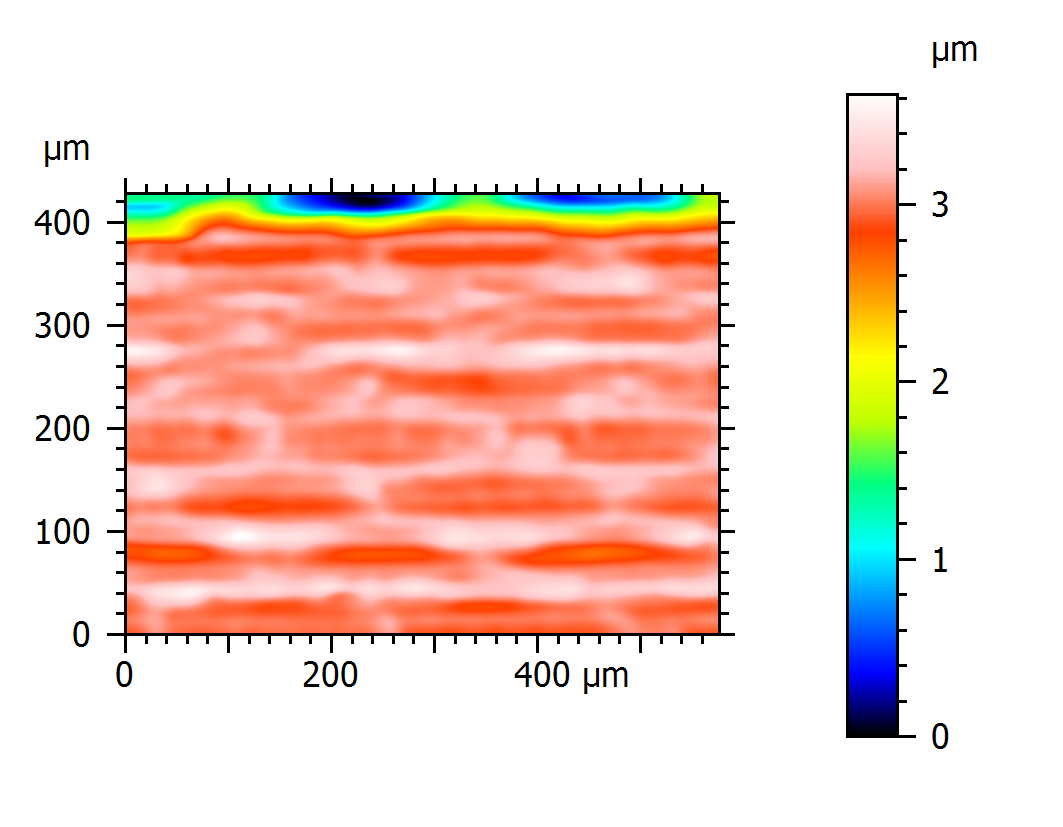

Supplement: Supplementary file 1 [file materials-13-03028-s001.zip › supplementary data/Bandpass filtering/2d_images_filtered_surfaces/MilledC_Number=9_CentralWavelength=48.png]

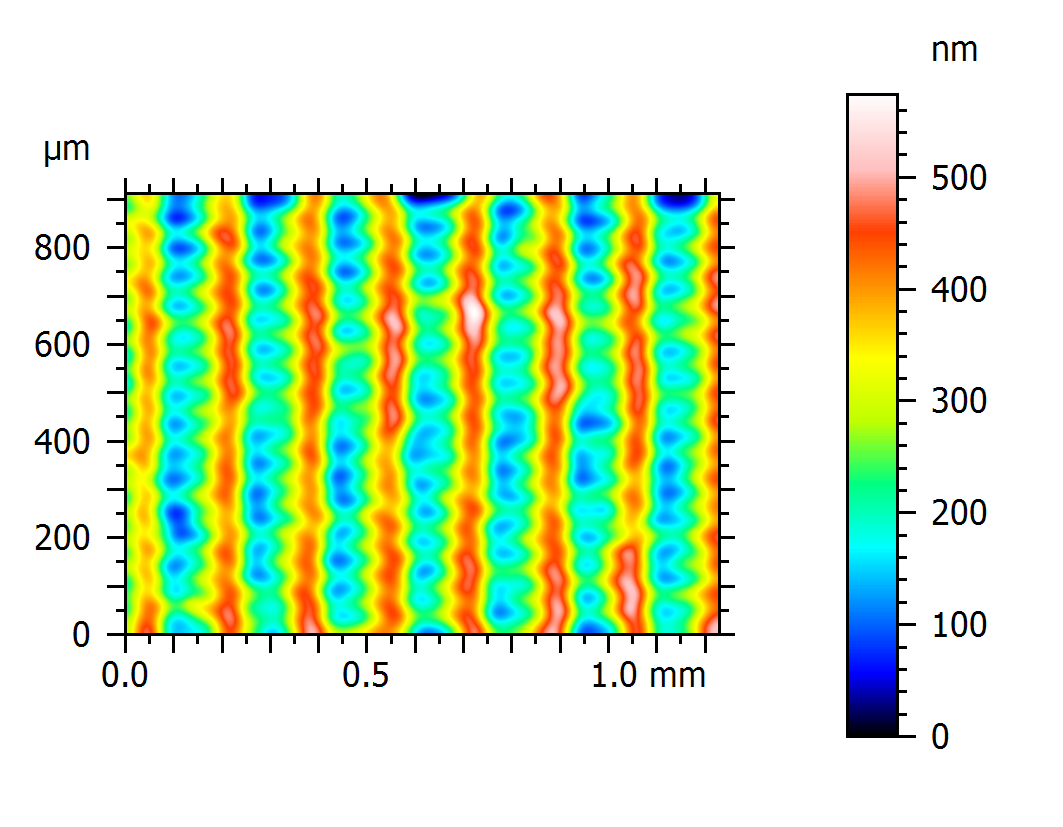

Supplement: Supplementary file 1 [file materials-13-03028-s001.zip › supplementary data/Bandpass filtering/2d_images_filtered_surfaces/MilledF_Number=10_CentralWavelength=144.png]

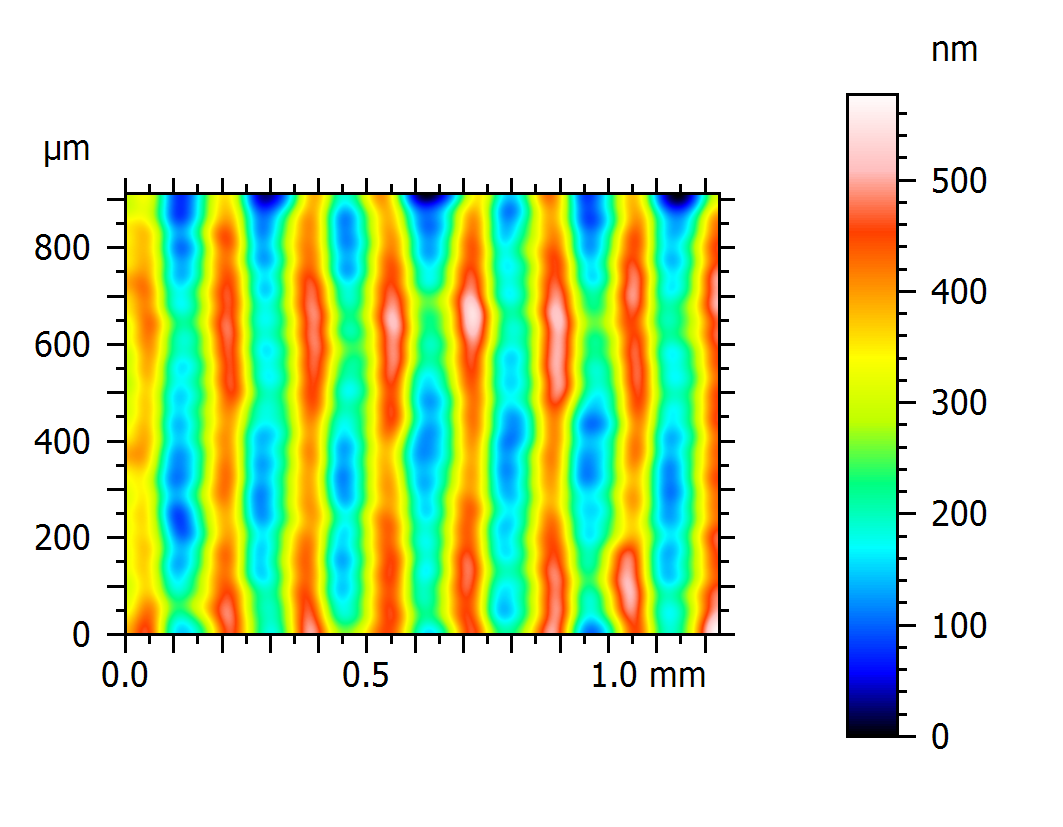

Supplement: Supplementary file 1 [file materials-13-03028-s001.zip › supplementary data/Bandpass filtering/2d_images_filtered_surfaces/MilledF_Number=11_CentralWavelength=192.png]

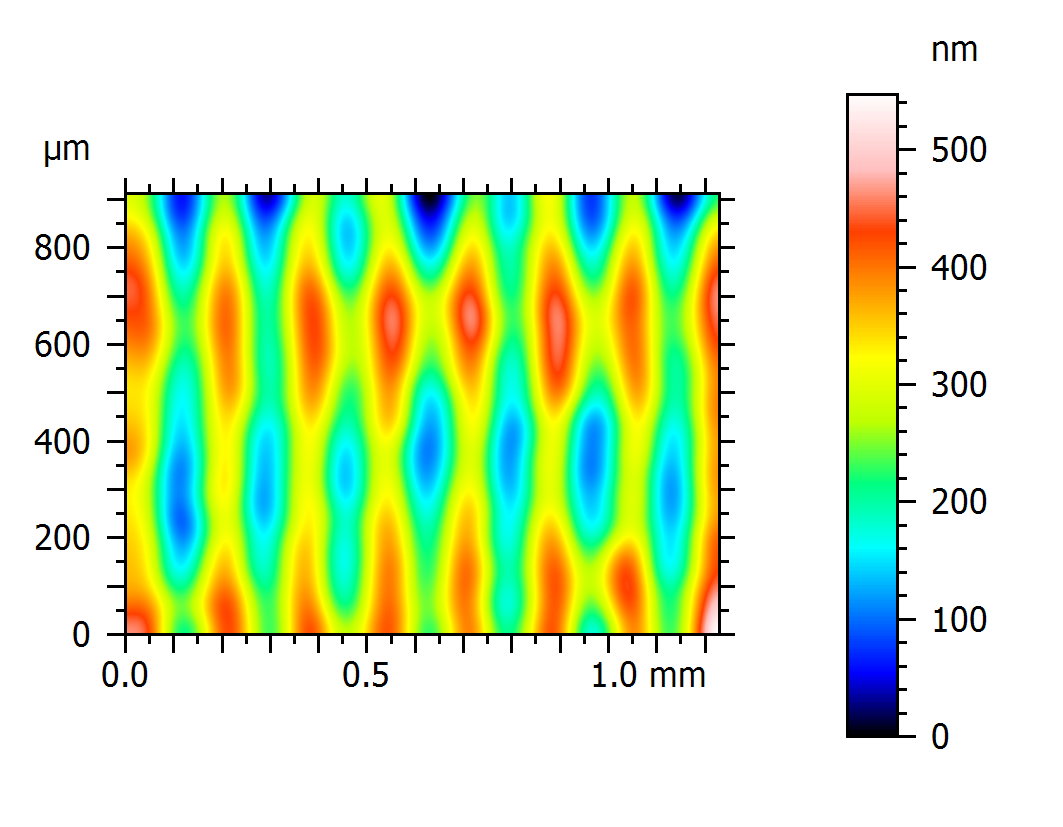

Supplement: Supplementary file 1 [file materials-13-03028-s001.zip › supplementary data/Bandpass filtering/2d_images_filtered_surfaces/MilledF_Number=12_CentralWavelength=270.png]

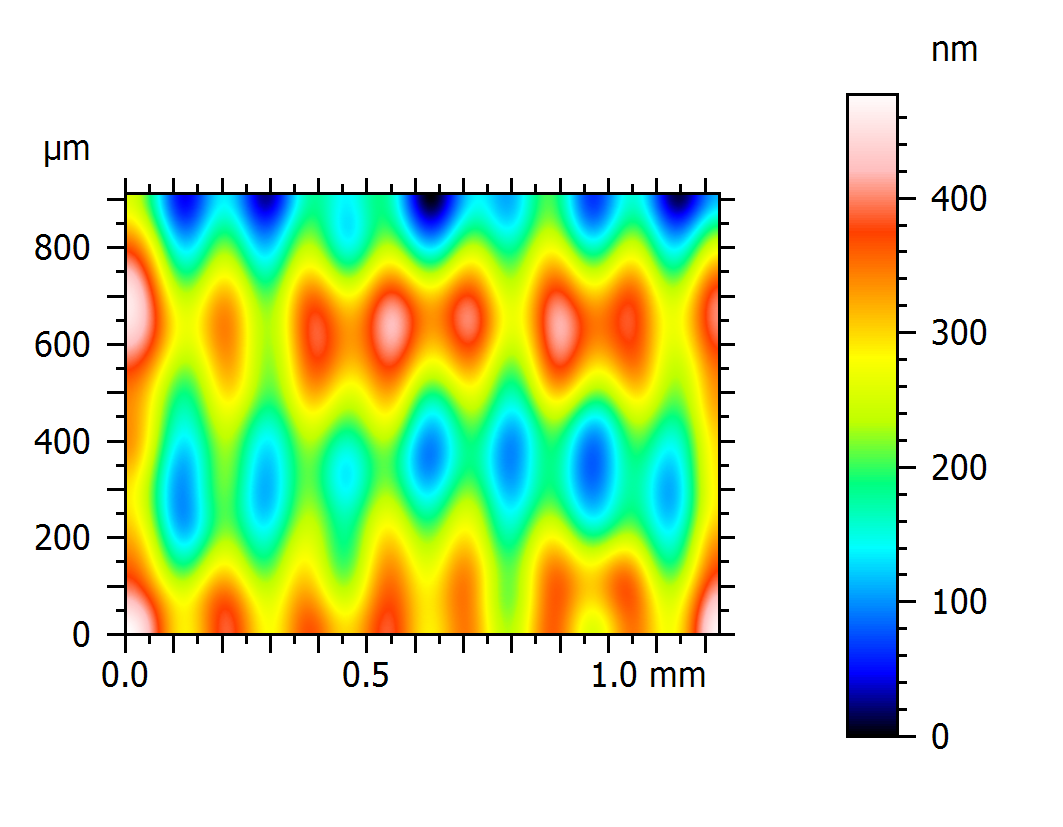

Supplement: Supplementary file 1 [file materials-13-03028-s001.zip › supplementary data/Bandpass filtering/2d_images_filtered_surfaces/MilledF_Number=13_CentralWavelength=384.png]

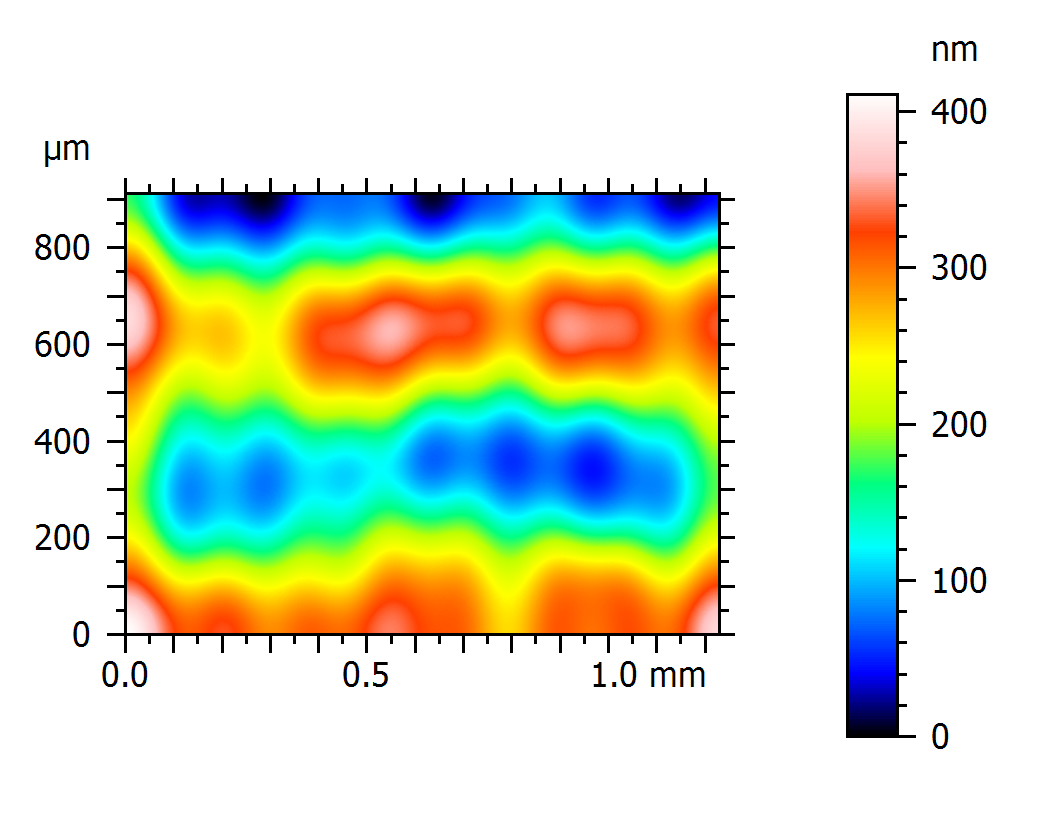

Supplement: Supplementary file 1 [file materials-13-03028-s001.zip › supplementary data/Bandpass filtering/2d_images_filtered_surfaces/MilledF_Number=14_CentralWavelength=522.png]

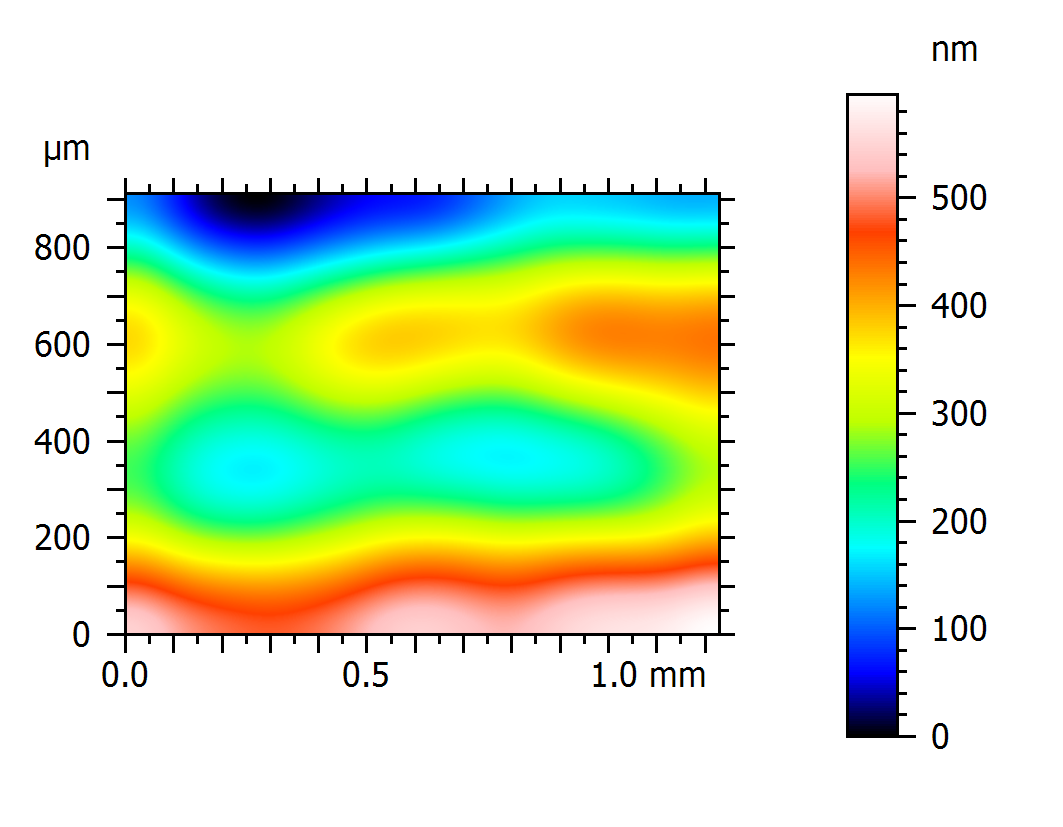

Supplement: Supplementary file 1 [file materials-13-03028-s001.zip › supplementary data/Bandpass filtering/2d_images_filtered_surfaces/MilledF_Number=15_CentralWavelength=768.png]

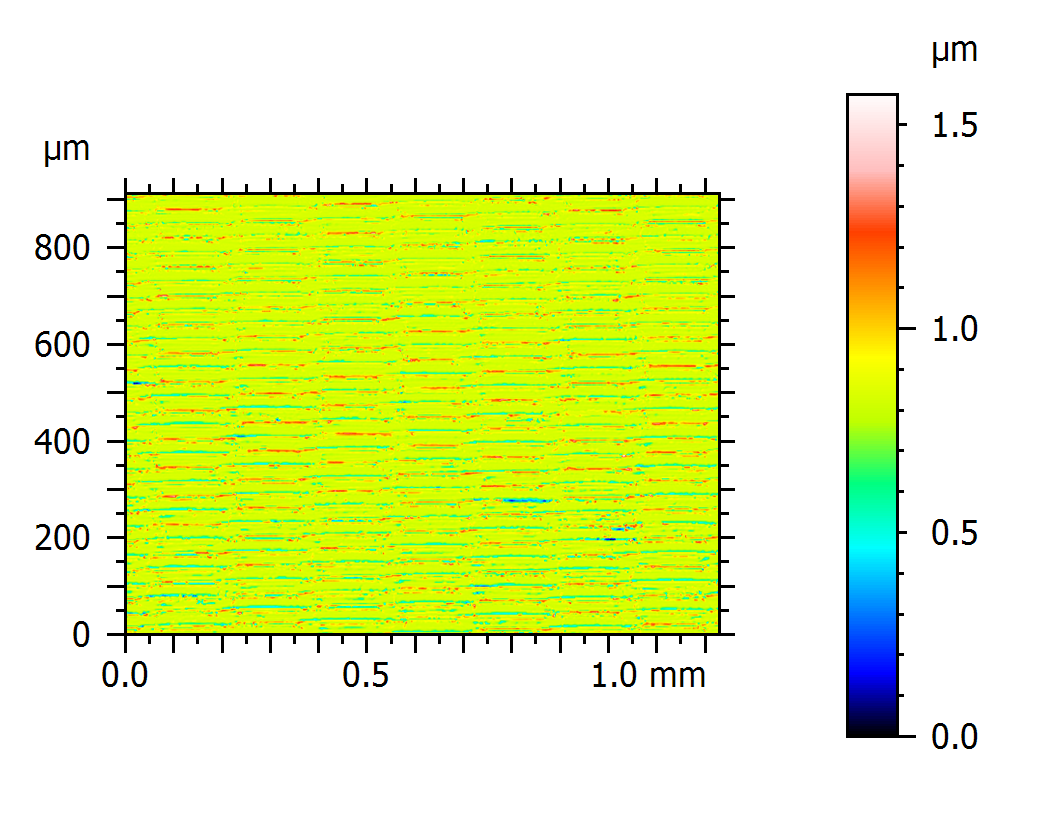

Supplement: Supplementary file 1 [file materials-13-03028-s001.zip › supplementary data/Bandpass filtering/2d_images_filtered_surfaces/MilledF_Number=1_CentralWavelength=6.png]

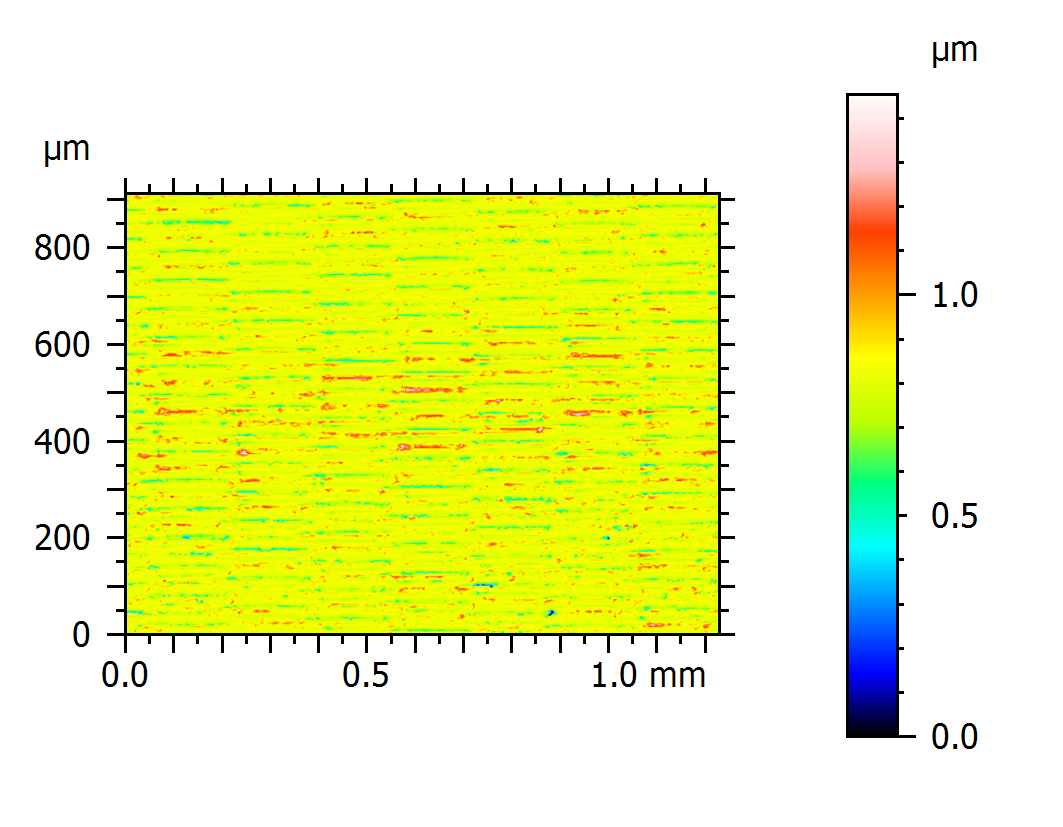

Supplement: Supplementary file 1 [file materials-13-03028-s001.zip › supplementary data/Bandpass filtering/2d_images_filtered_surfaces/MilledF_Number=2_CentralWavelength=9.png]

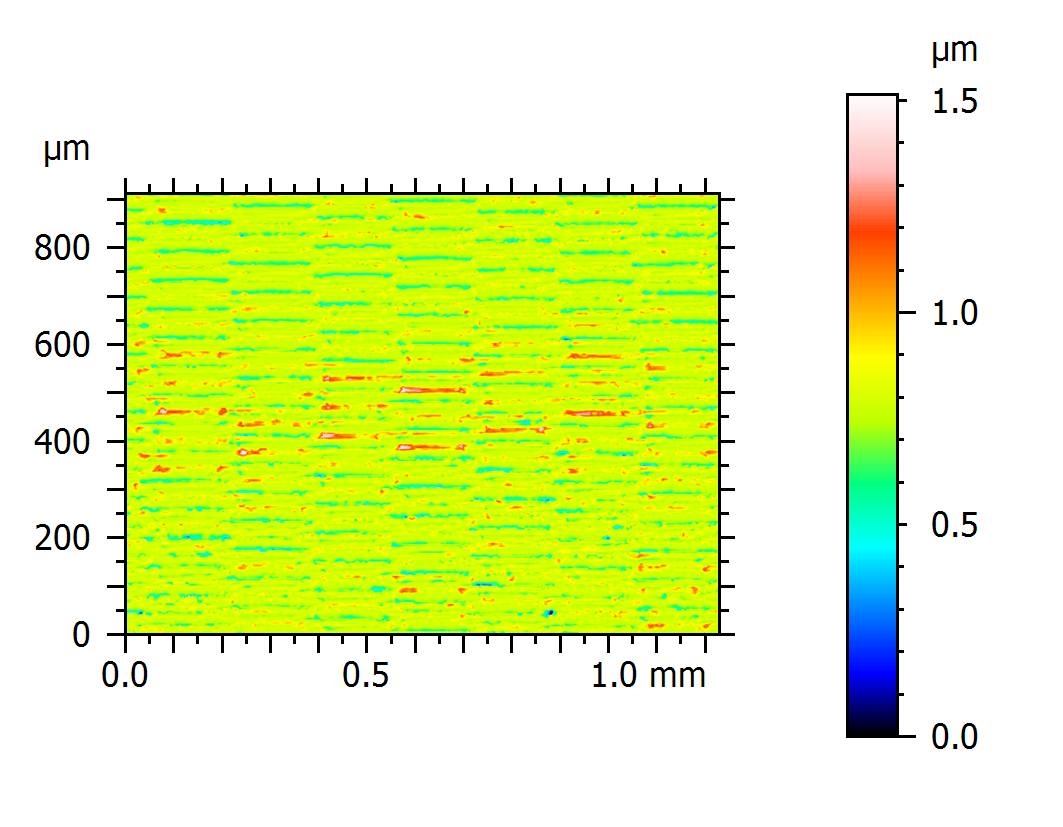

Supplement: Supplementary file 1 [file materials-13-03028-s001.zip › supplementary data/Bandpass filtering/2d_images_filtered_surfaces/MilledF_Number=3_CentralWavelength=12.png]

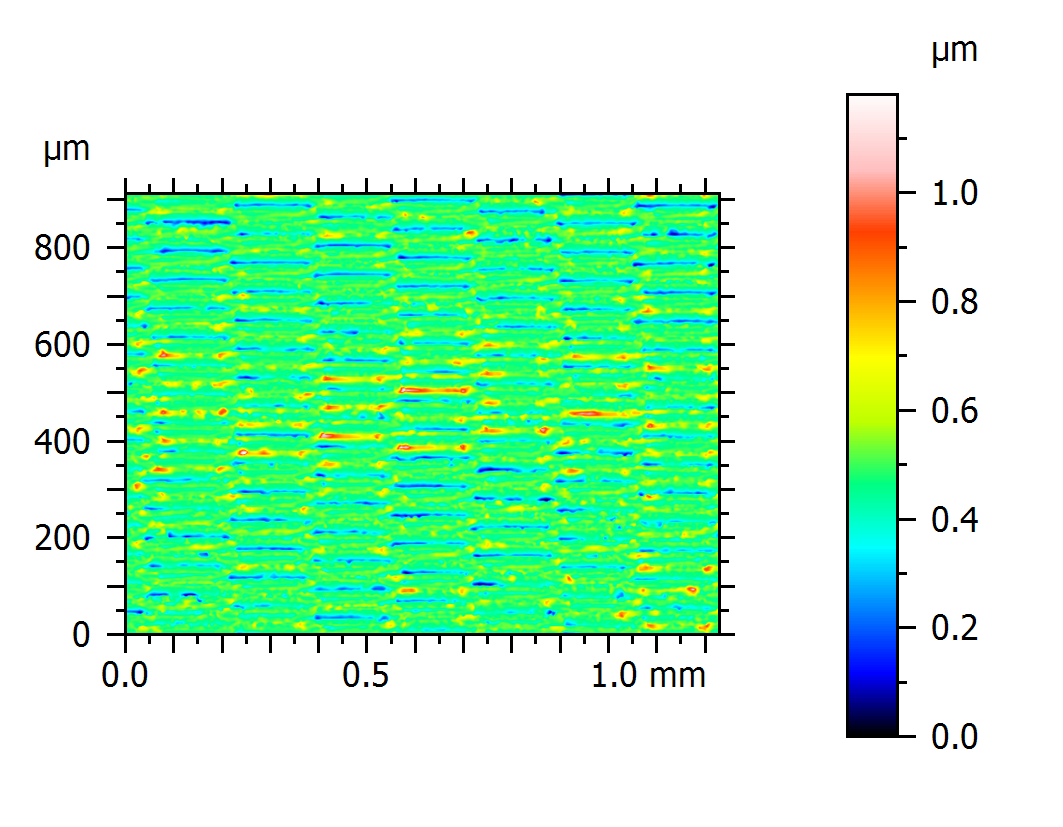

Supplement: Supplementary file 1 [file materials-13-03028-s001.zip › supplementary data/Bandpass filtering/2d_images_filtered_surfaces/MilledF_Number=4_CentralWavelength=18.png]

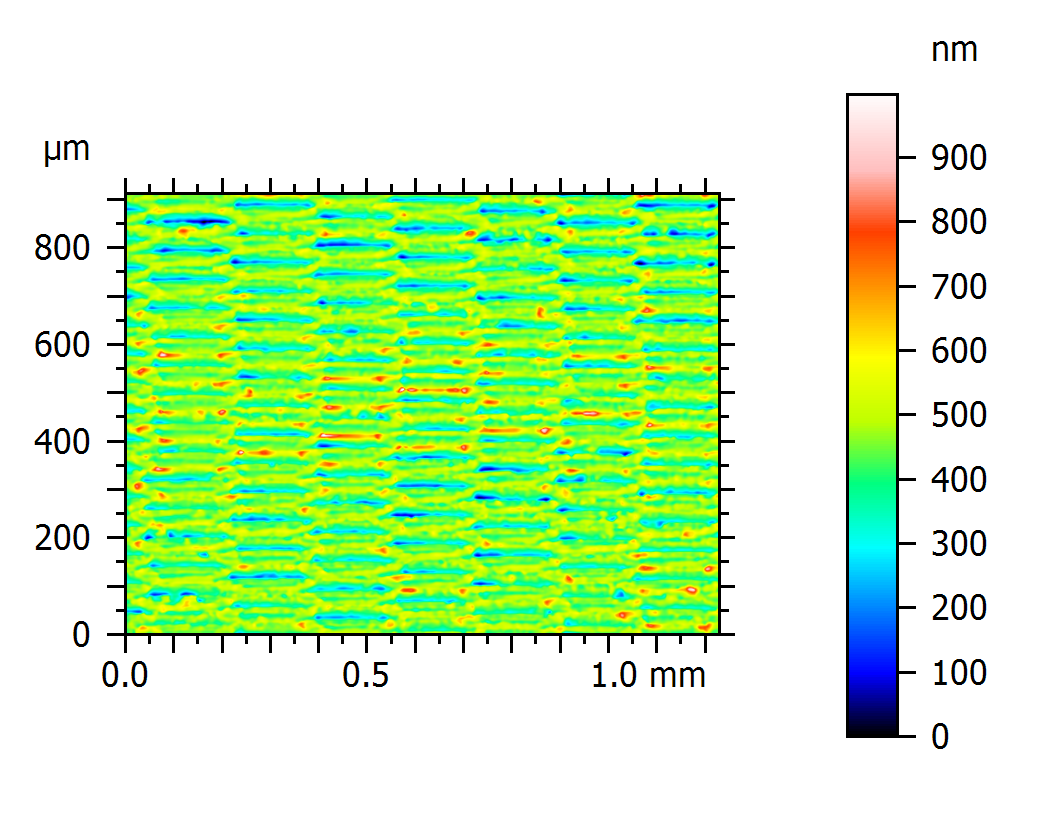

Supplement: Supplementary file 1 [file materials-13-03028-s001.zip › supplementary data/Bandpass filtering/2d_images_filtered_surfaces/MilledF_Number=5_CentralWavelength=24.png]

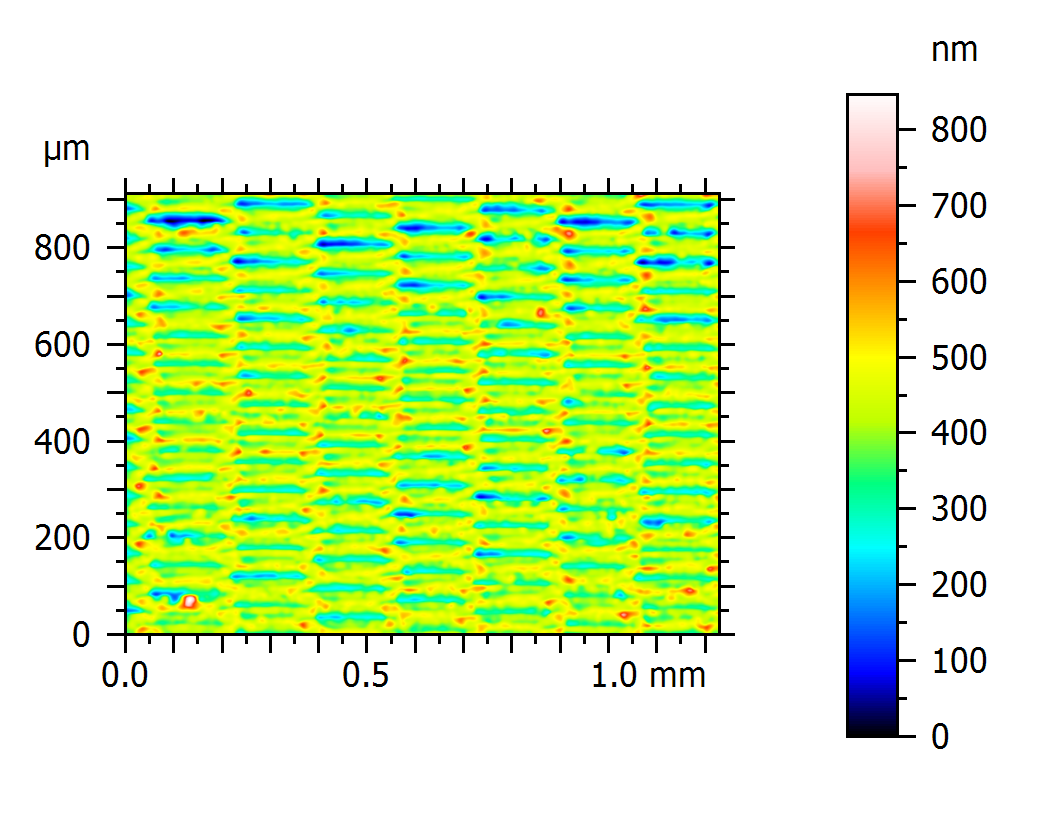

Supplement: Supplementary file 1 [file materials-13-03028-s001.zip › supplementary data/Bandpass filtering/2d_images_filtered_surfaces/MilledF_Number=6_CentralWavelength=36.png]

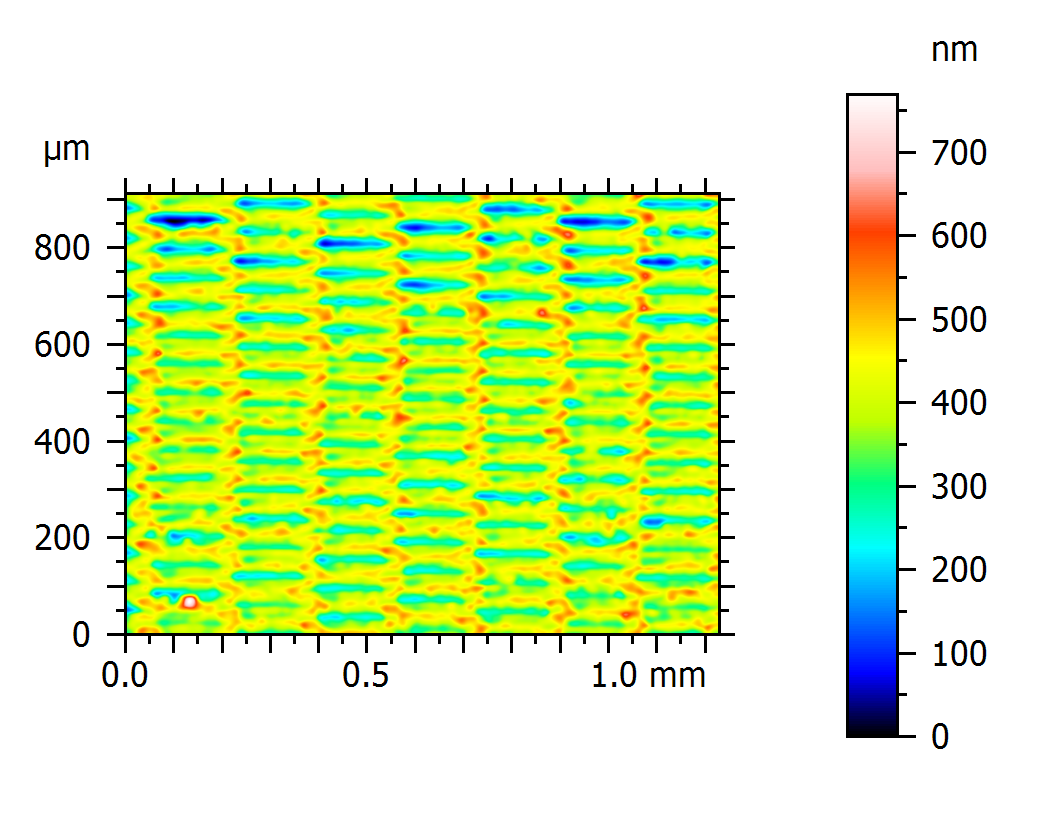

Supplement: Supplementary file 1 [file materials-13-03028-s001.zip › supplementary data/Bandpass filtering/2d_images_filtered_surfaces/MilledF_Number=7_CentralWavelength=48.png]

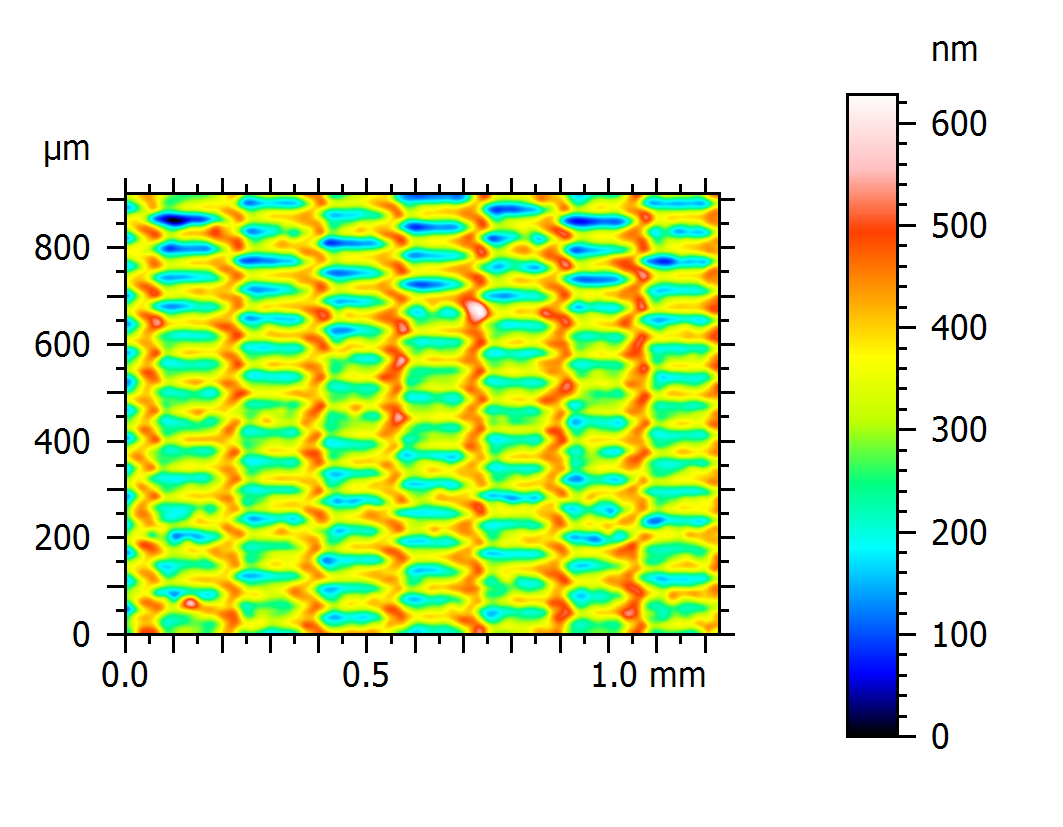

Supplement: Supplementary file 1 [file materials-13-03028-s001.zip › supplementary data/Bandpass filtering/2d_images_filtered_surfaces/MilledF_Number=8_CentralWavelength=72.png]

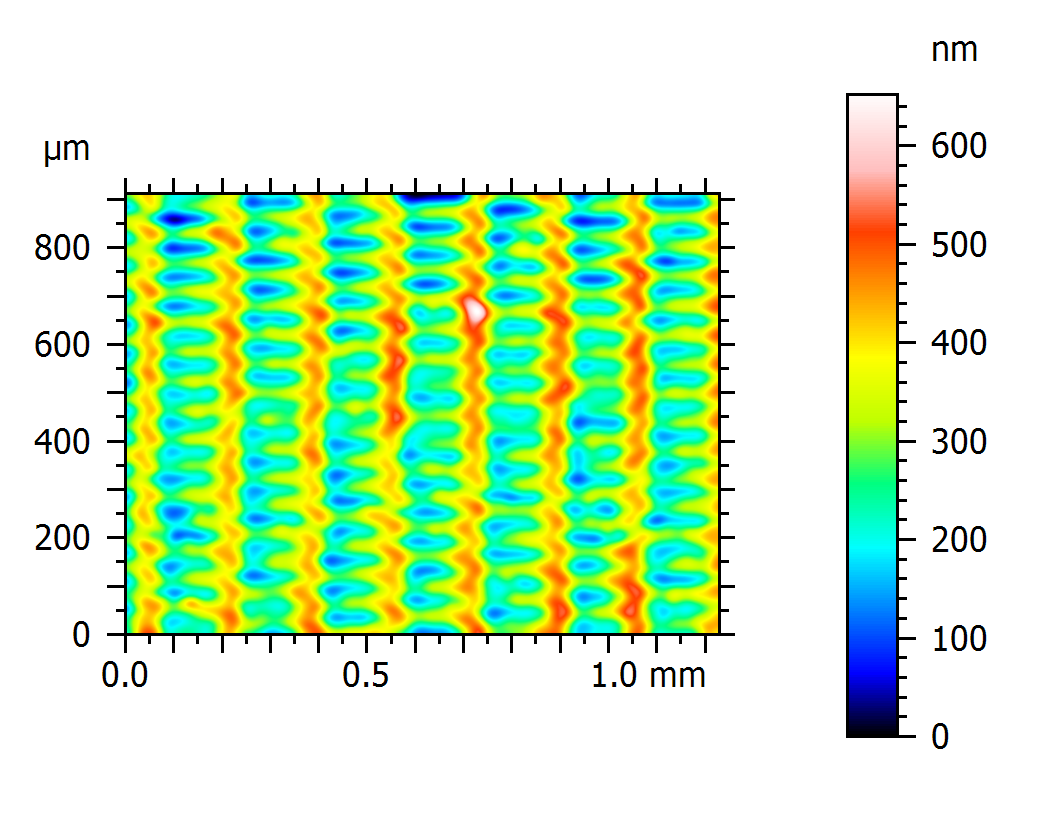

Supplement: Supplementary file 1 [file materials-13-03028-s001.zip › supplementary data/Bandpass filtering/2d_images_filtered_surfaces/MilledF_Number=9_CentralWavelength=96.png]

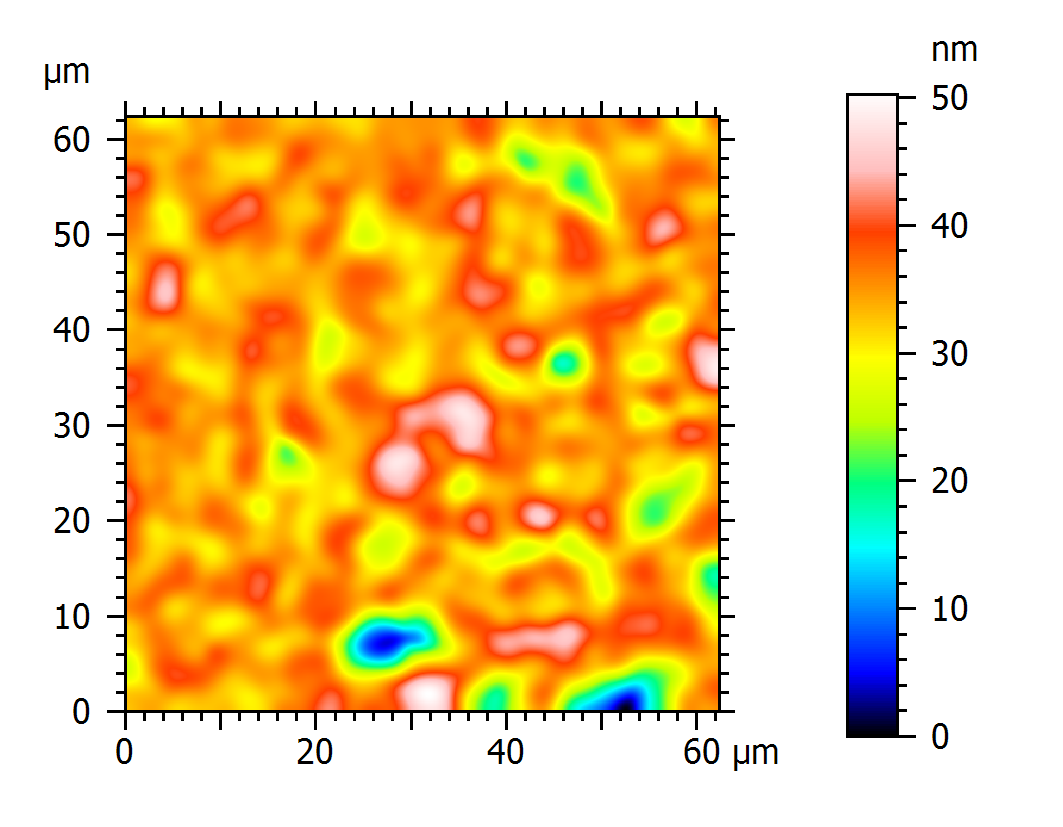

Supplement: Supplementary file 1 [file materials-13-03028-s001.zip › supplementary data/Bandpass filtering/2d_images_filtered_surfaces/μEDMed_Number=10_CentralWavelength=9.png]

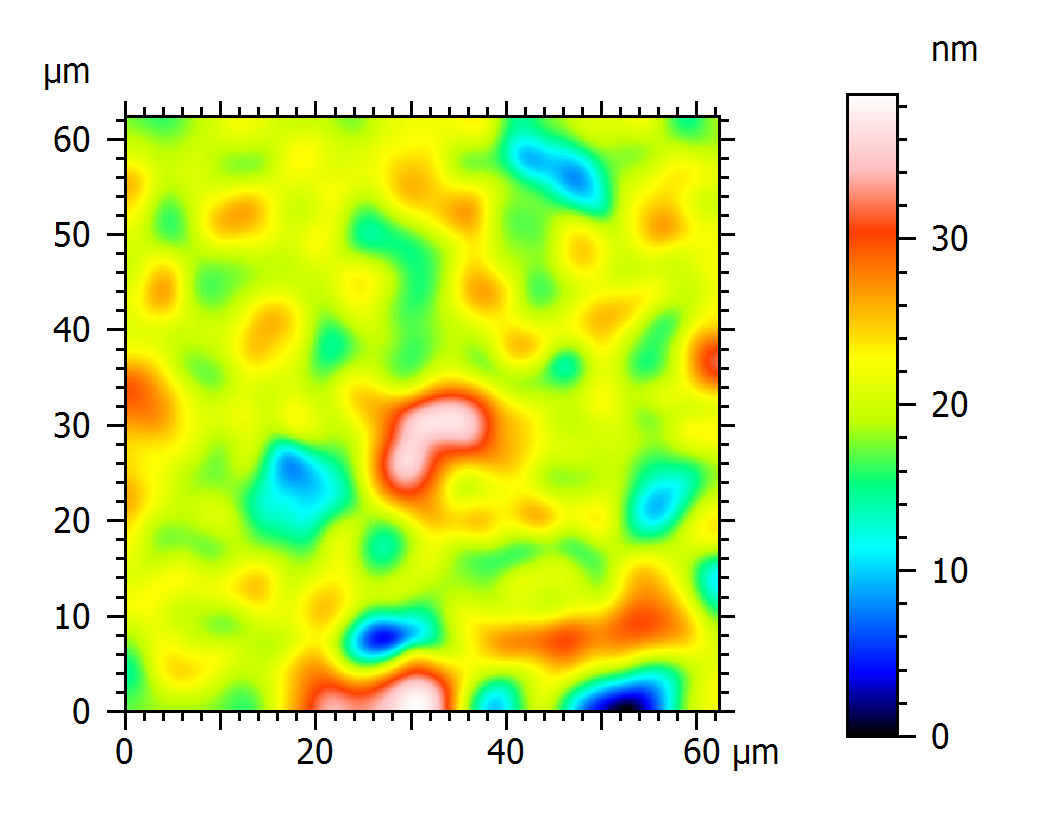

Supplement: Supplementary file 1 [file materials-13-03028-s001.zip › supplementary data/Bandpass filtering/2d_images_filtered_surfaces/μEDMed_Number=11_CentralWavelength=13.5.png]

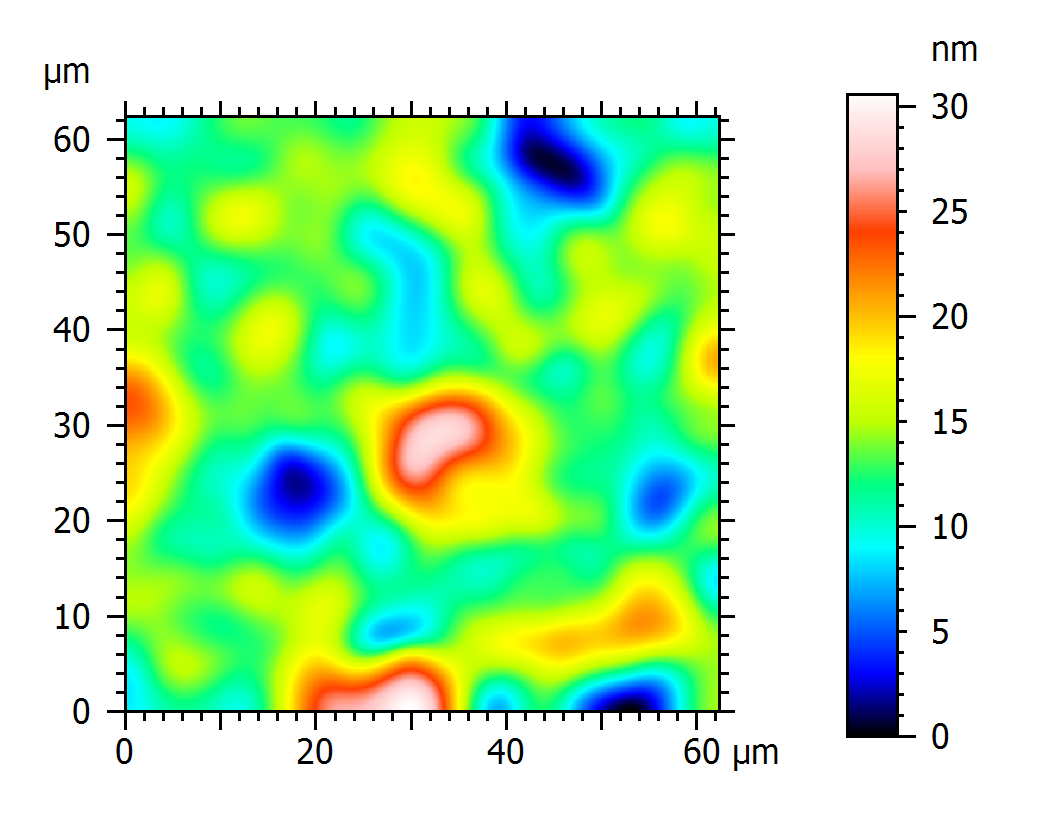

Supplement: Supplementary file 1 [file materials-13-03028-s001.zip › supplementary data/Bandpass filtering/2d_images_filtered_surfaces/μEDMed_Number=12_CentralWavelength=18.png]

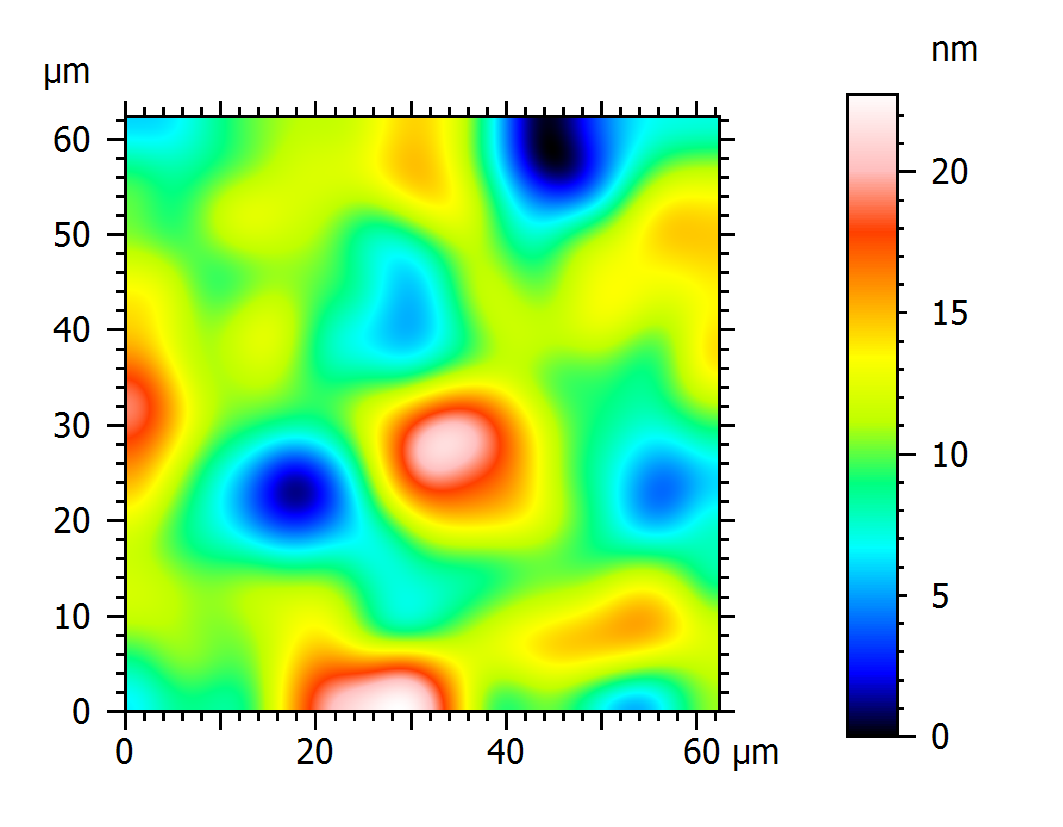

Supplement: Supplementary file 1 [file materials-13-03028-s001.zip › supplementary data/Bandpass filtering/2d_images_filtered_surfaces/μEDMed_Number=13_CentralWavelength=27.png]

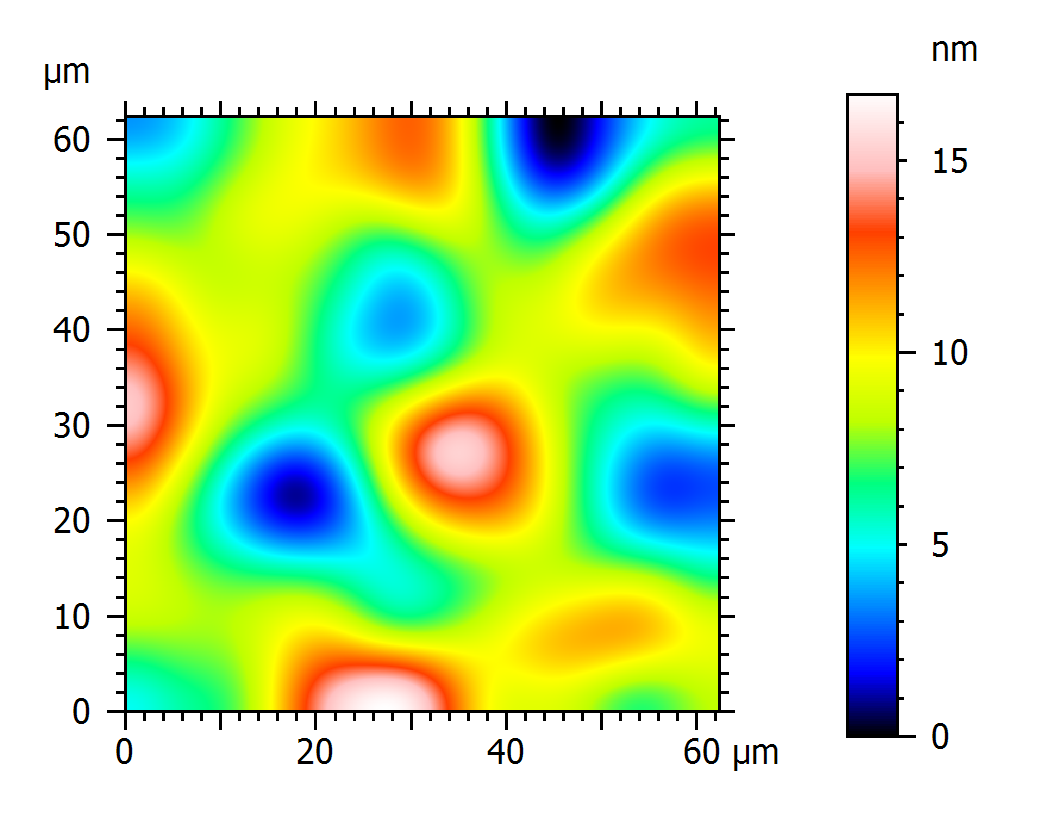

Supplement: Supplementary file 1 [file materials-13-03028-s001.zip › supplementary data/Bandpass filtering/2d_images_filtered_surfaces/μEDMed_Number=14_CentralWavelength=36.png]

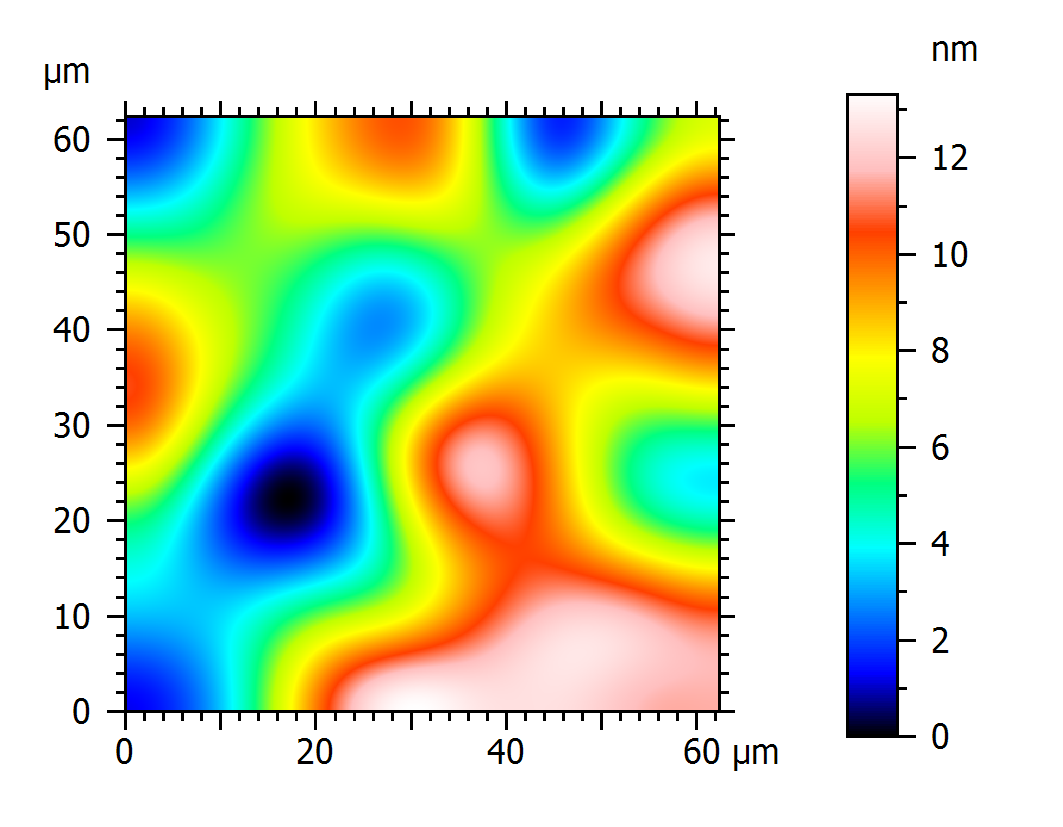

Supplement: Supplementary file 1 [file materials-13-03028-s001.zip › supplementary data/Bandpass filtering/2d_images_filtered_surfaces/μEDMed_Number=15_CentralWavelength=48.png]

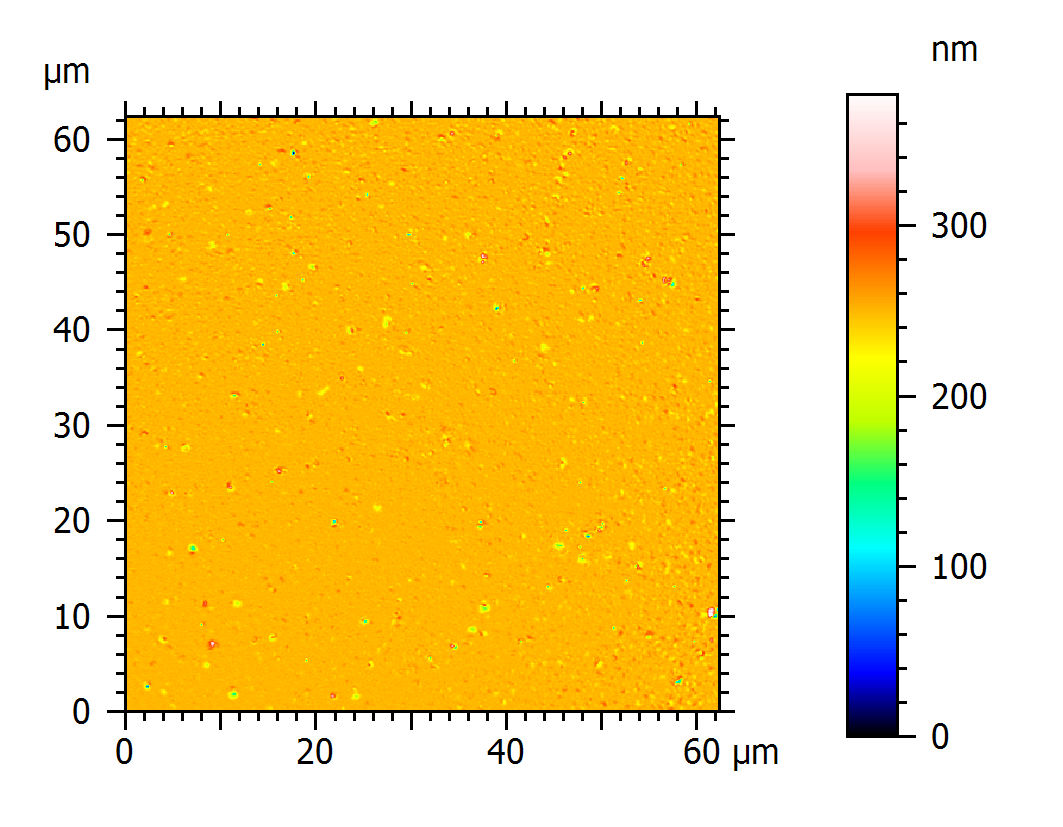

Supplement: Supplementary file 1 [file materials-13-03028-s001.zip › supplementary data/Bandpass filtering/2d_images_filtered_surfaces/μEDMed_Number=1_CentralWavelength=0.421875.png]

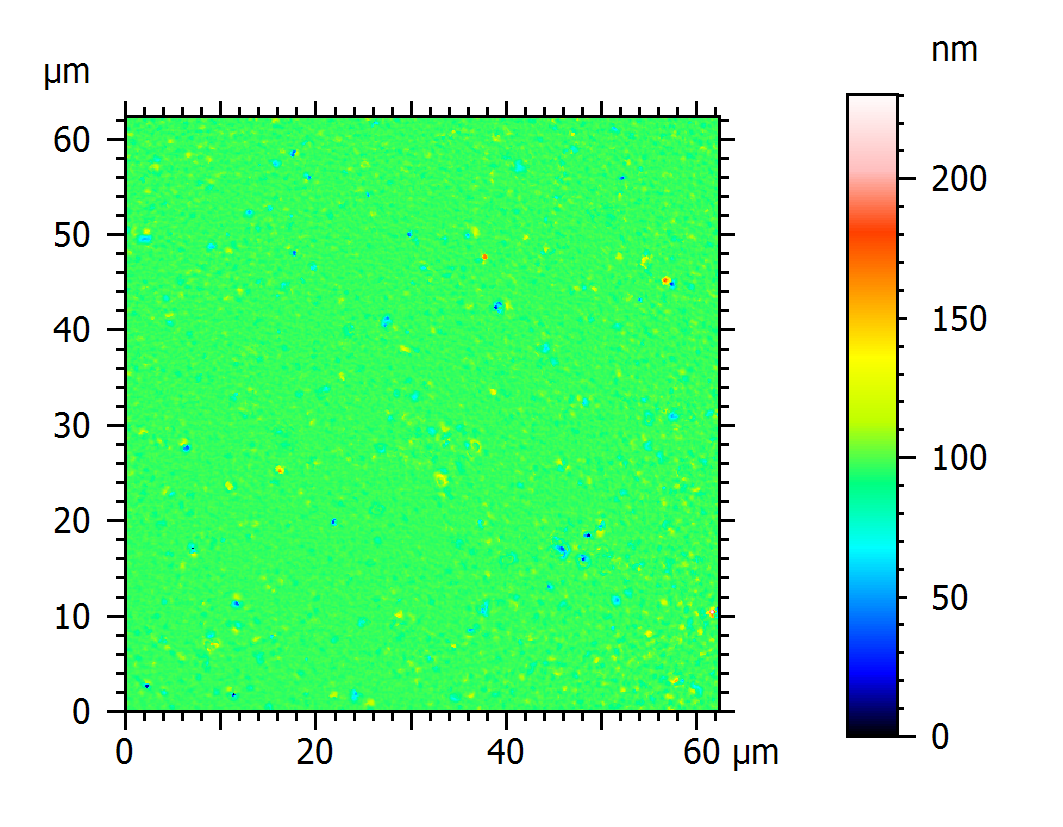

Supplement: Supplementary file 1 [file materials-13-03028-s001.zip › supplementary data/Bandpass filtering/2d_images_filtered_surfaces/μEDMed_Number=2_CentralWavelength=0.5625.png]

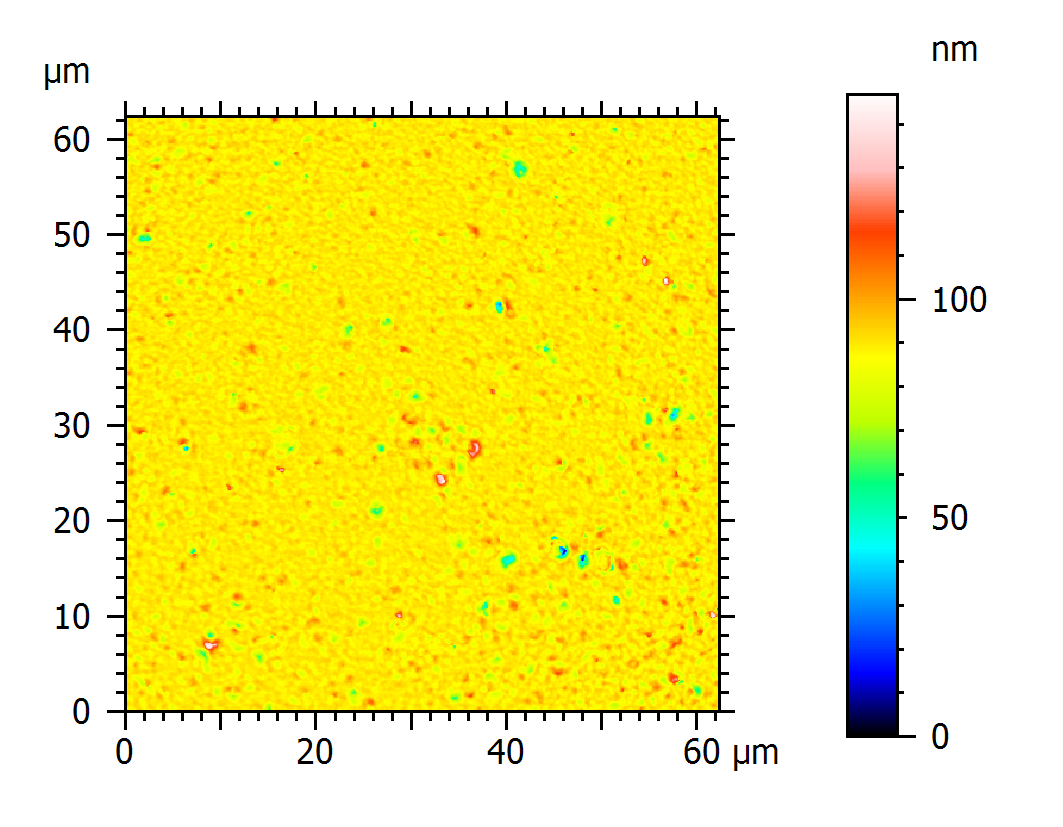

Supplement: Supplementary file 1 [file materials-13-03028-s001.zip › supplementary data/Bandpass filtering/2d_images_filtered_surfaces/μEDMed_Number=3_CentralWavelength=0.84375.png]

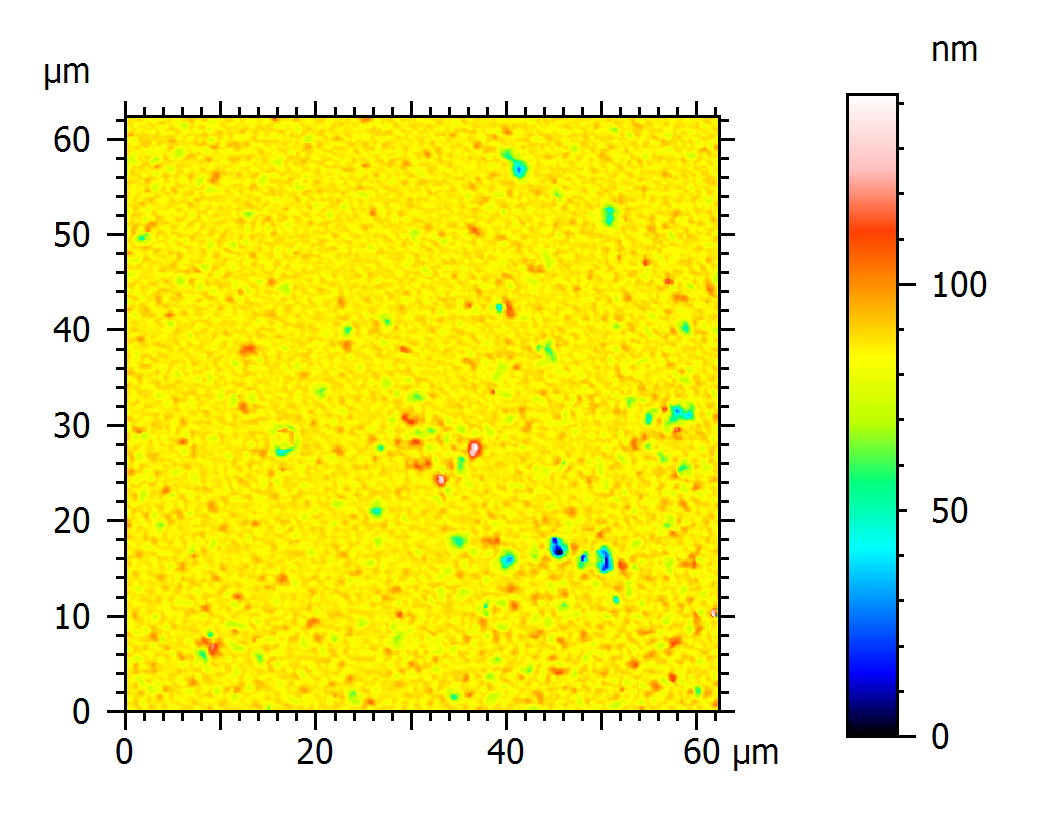

Supplement: Supplementary file 1 [file materials-13-03028-s001.zip › supplementary data/Bandpass filtering/2d_images_filtered_surfaces/μEDMed_Number=4_CentralWavelength=1.125.png]

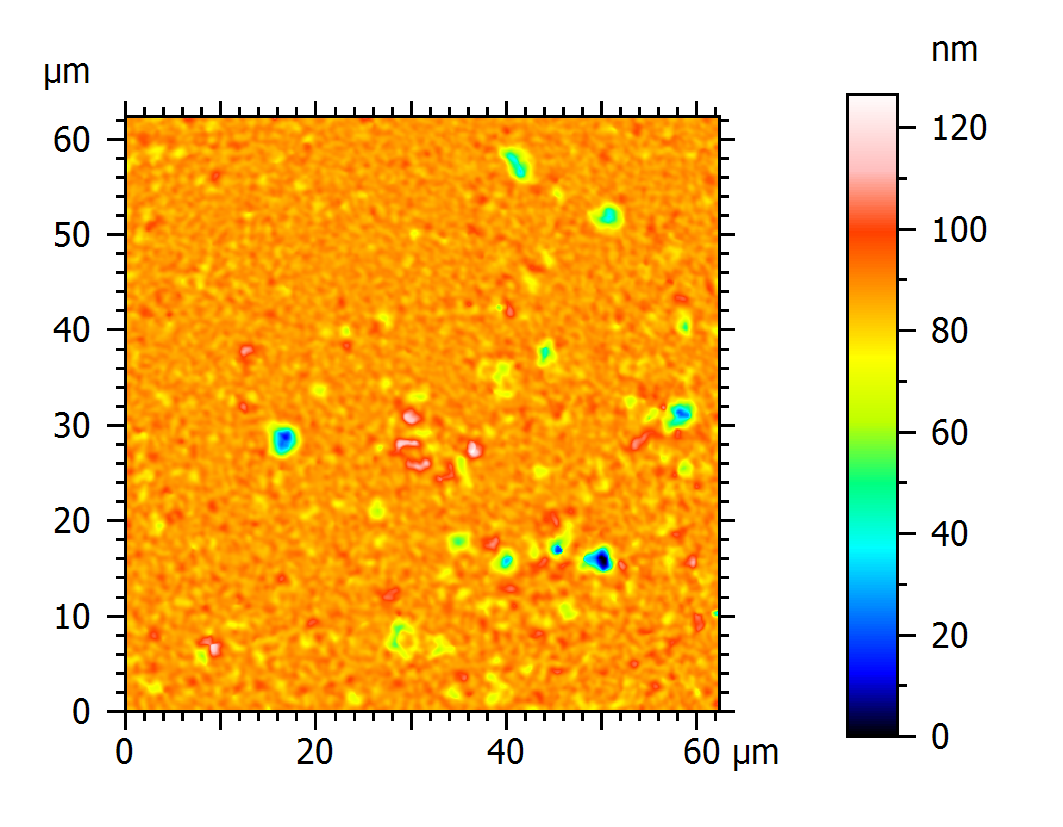

Supplement: Supplementary file 1 [file materials-13-03028-s001.zip › supplementary data/Bandpass filtering/2d_images_filtered_surfaces/μEDMed_Number=5_CentralWavelength=1.6875.png]

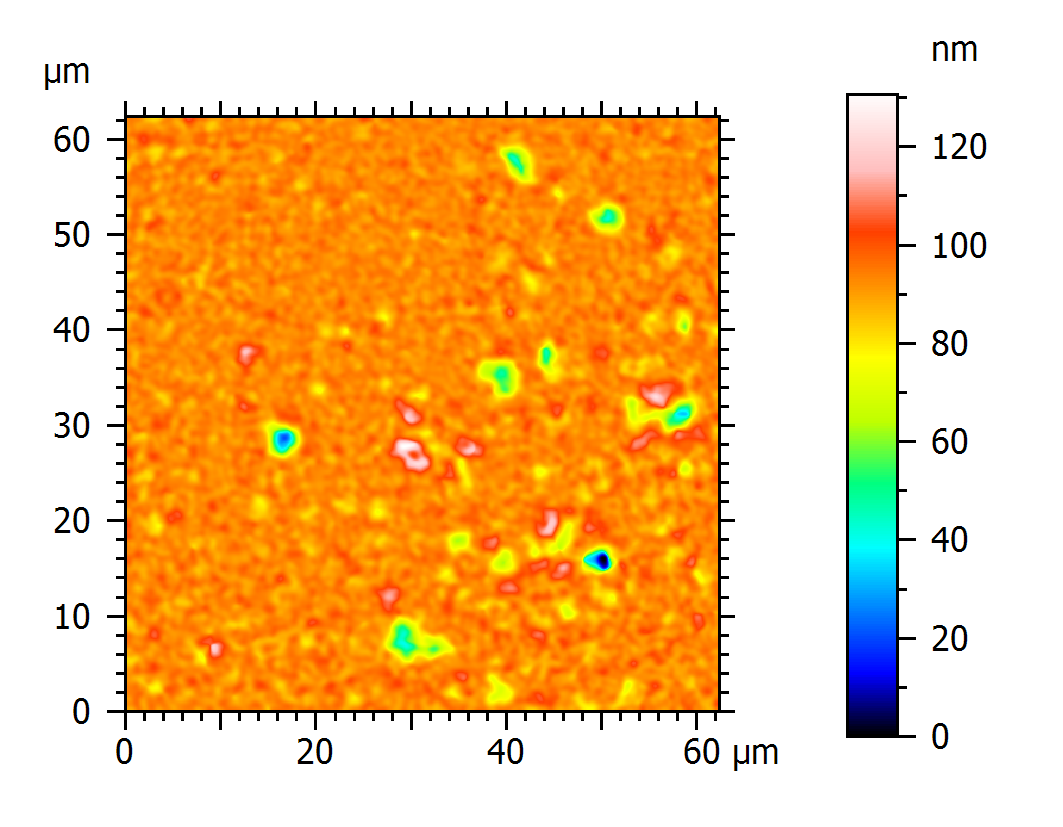

Supplement: Supplementary file 1 [file materials-13-03028-s001.zip › supplementary data/Bandpass filtering/2d_images_filtered_surfaces/μEDMed_Number=6_CentralWavelength=2.25.png]

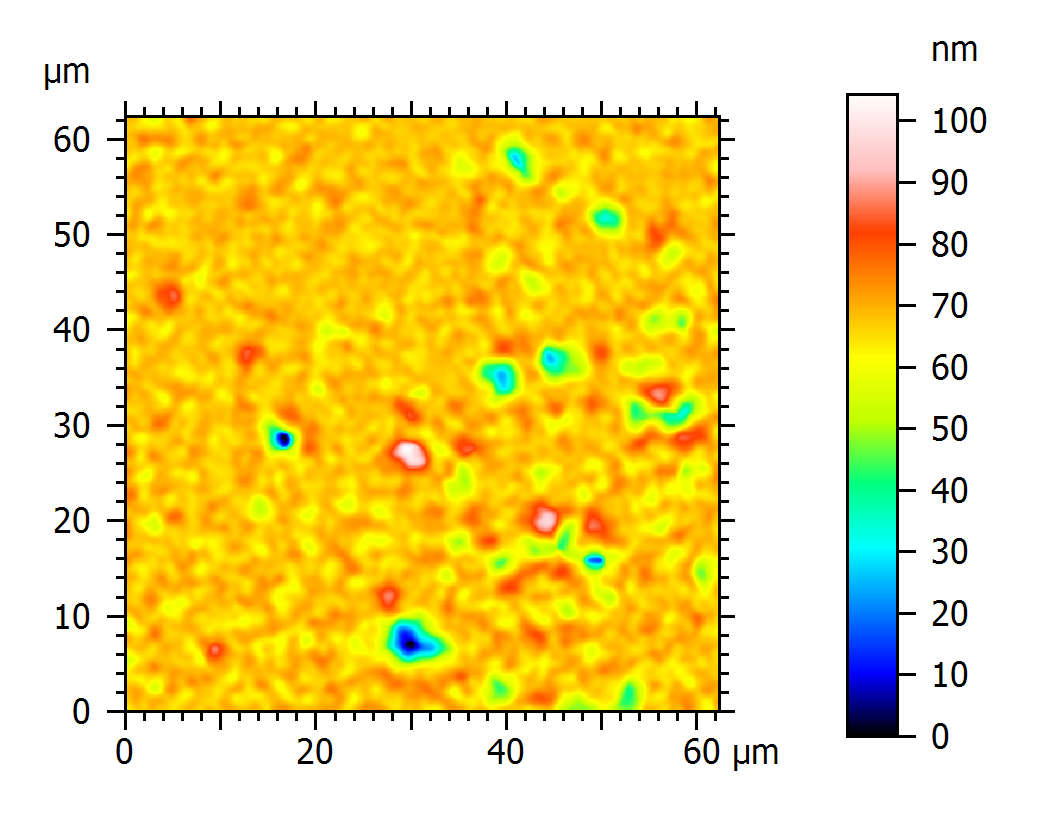

Supplement: Supplementary file 1 [file materials-13-03028-s001.zip › supplementary data/Bandpass filtering/2d_images_filtered_surfaces/μEDMed_Number=7_CentralWavelength=3.375.png]

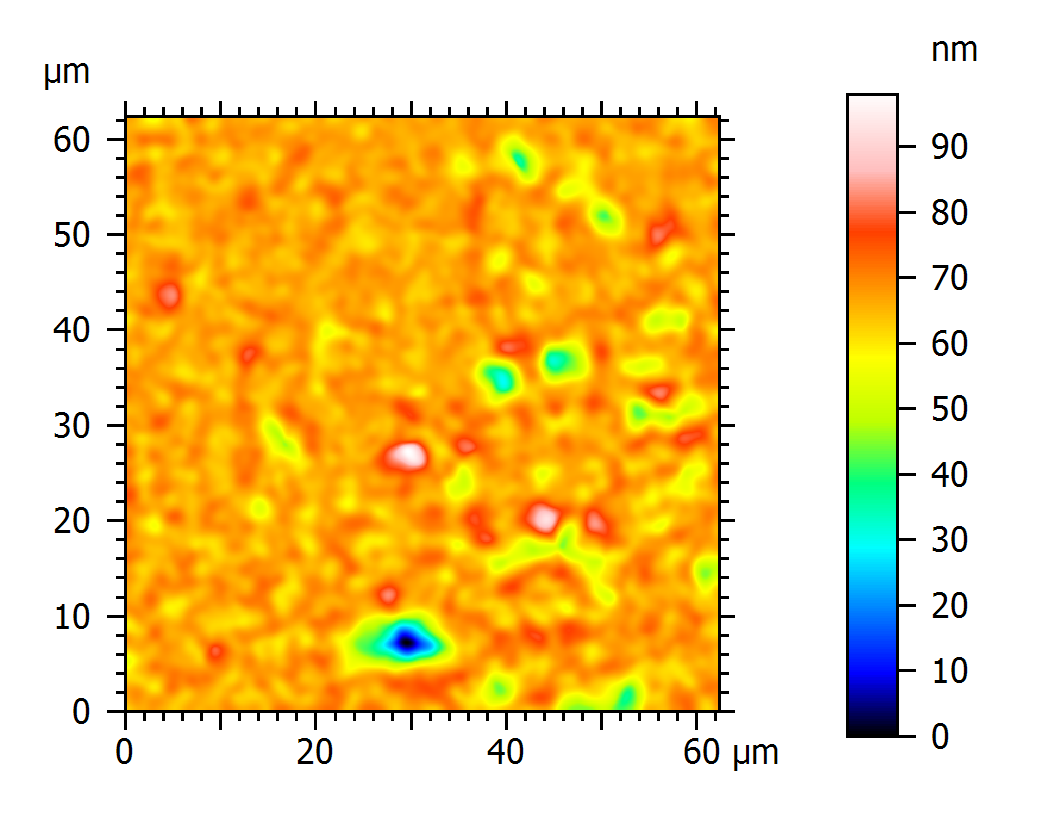

Supplement: Supplementary file 1 [file materials-13-03028-s001.zip › supplementary data/Bandpass filtering/2d_images_filtered_surfaces/μEDMed_Number=8_CentralWavelength=4.5.png]

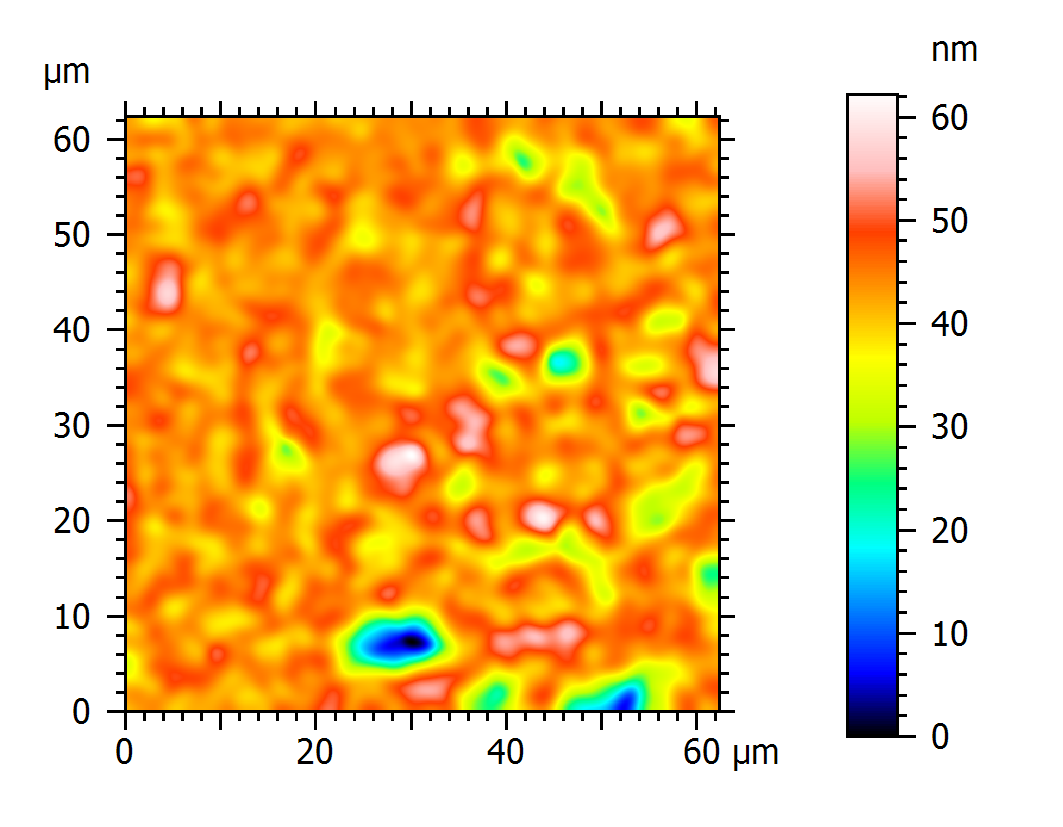

Supplement: Supplementary file 1 [file materials-13-03028-s001.zip › supplementary data/Bandpass filtering/2d_images_filtered_surfaces/μEDMed_Number=9_CentralWavelength=6.75.png]

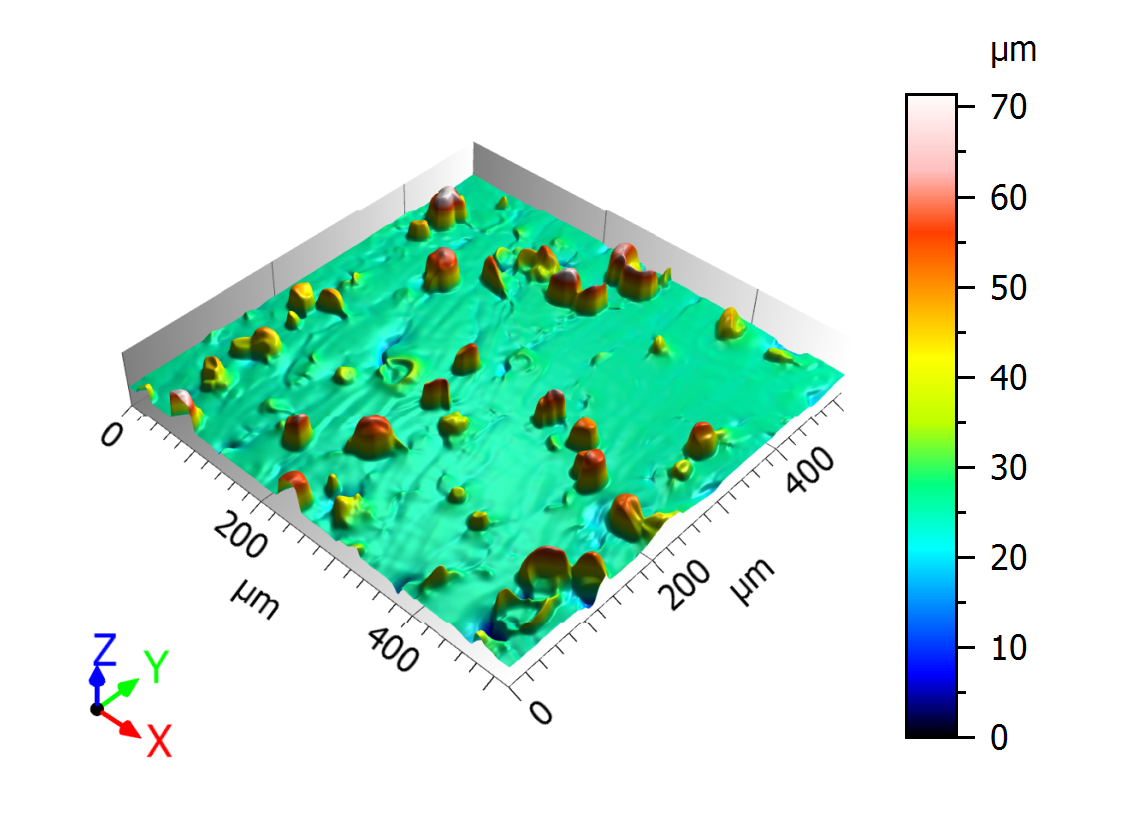

Supplement: Supplementary file 1 [file materials-13-03028-s001.zip › supplementary data/Bandpass filtering/3d_images_filtered_surfaces/L-PBFed_Number=10_CentralWavelength=24.png]

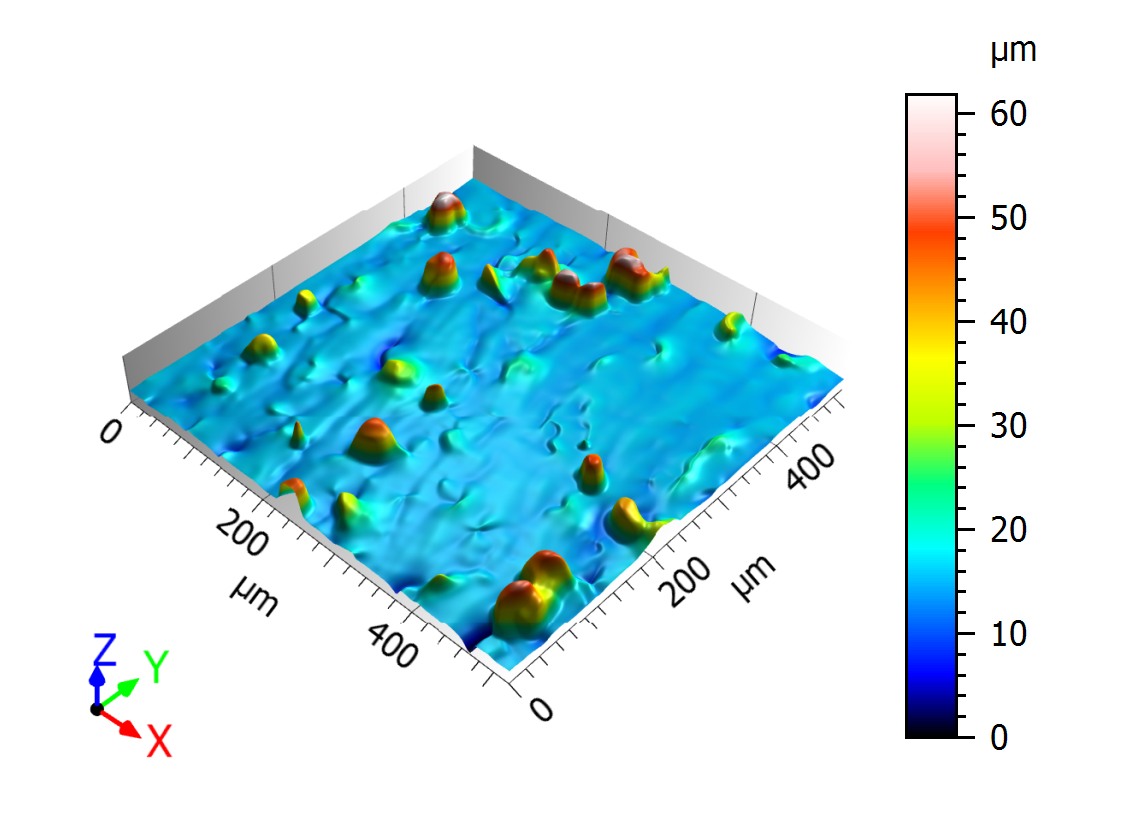

Supplement: Supplementary file 1 [file materials-13-03028-s001.zip › supplementary data/Bandpass filtering/3d_images_filtered_surfaces/L-PBFed_Number=11_CentralWavelength=36.png]

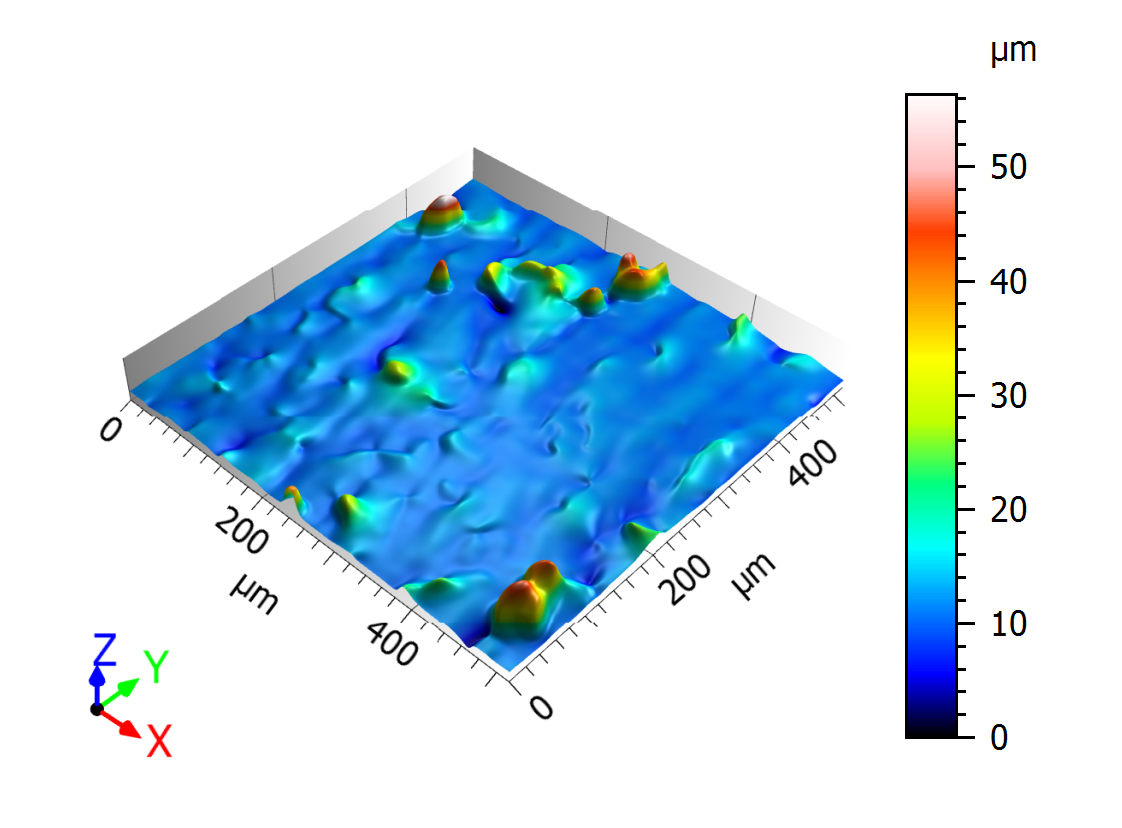

Supplement: Supplementary file 1 [file materials-13-03028-s001.zip › supplementary data/Bandpass filtering/3d_images_filtered_surfaces/L-PBFed_Number=12_CentralWavelength=48.png]

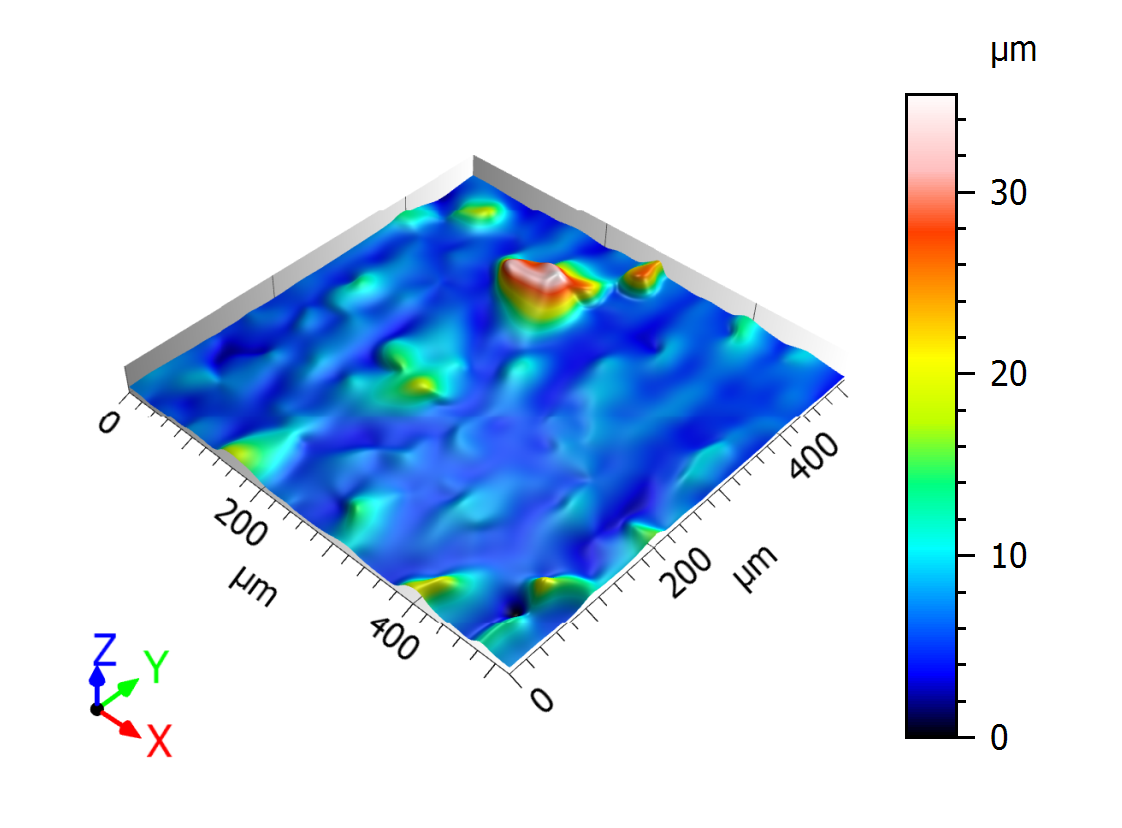

Supplement: Supplementary file 1 [file materials-13-03028-s001.zip › supplementary data/Bandpass filtering/3d_images_filtered_surfaces/L-PBFed_Number=13_CentralWavelength=72.png]

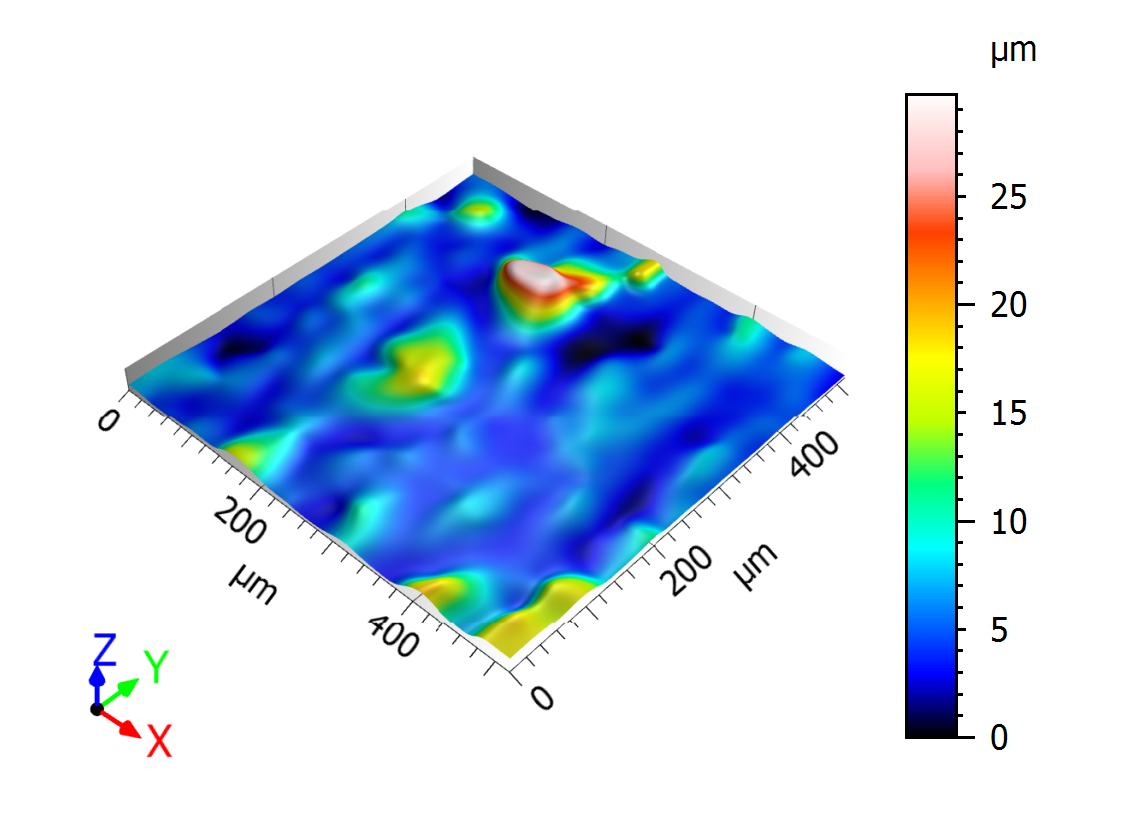

Supplement: Supplementary file 1 [file materials-13-03028-s001.zip › supplementary data/Bandpass filtering/3d_images_filtered_surfaces/L-PBFed_Number=14_CentralWavelength=96.png]

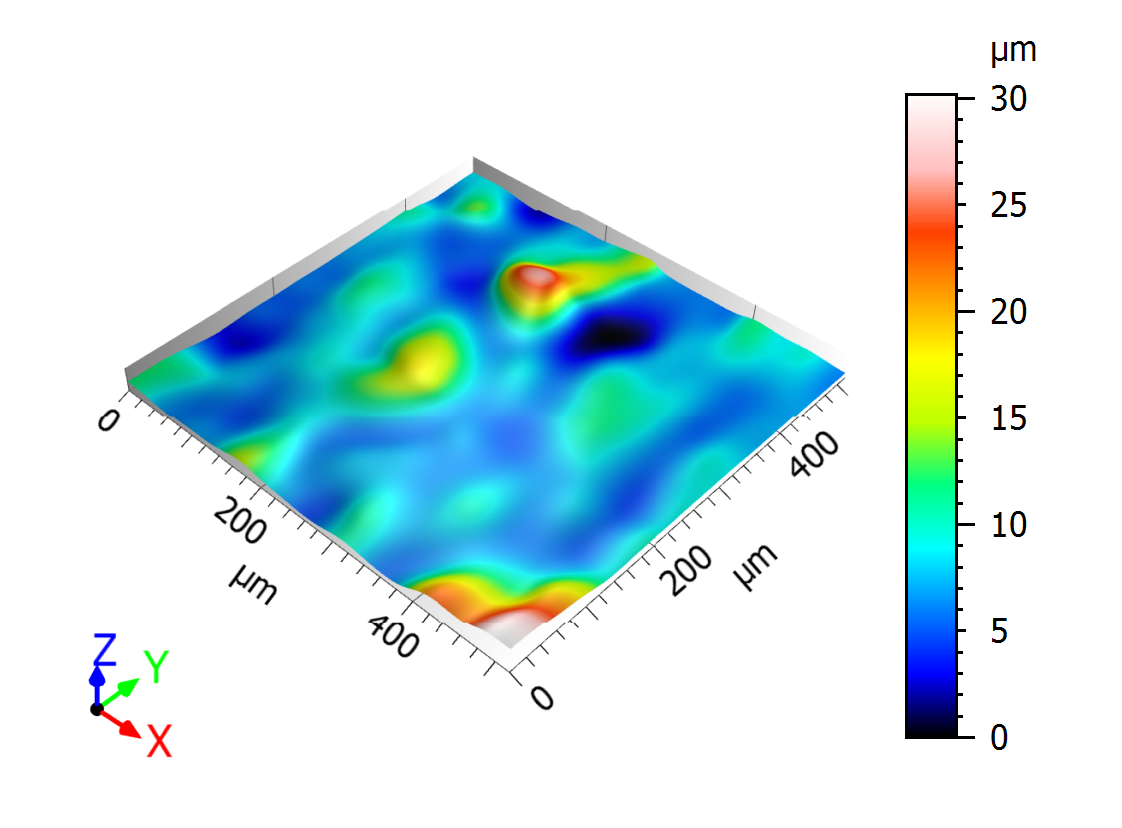

Supplement: Supplementary file 1 [file materials-13-03028-s001.zip › supplementary data/Bandpass filtering/3d_images_filtered_surfaces/L-PBFed_Number=15_CentralWavelength=144.png]

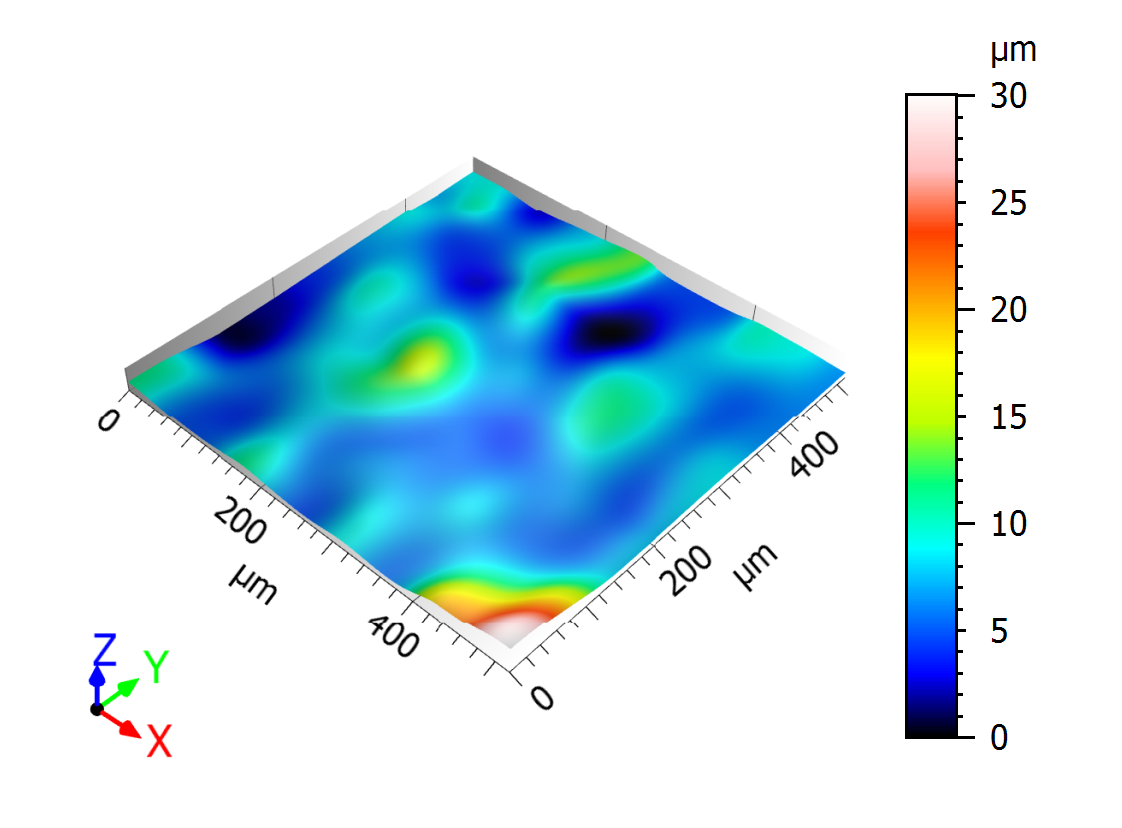

Supplement: Supplementary file 1 [file materials-13-03028-s001.zip › supplementary data/Bandpass filtering/3d_images_filtered_surfaces/L-PBFed_Number=16_CentralWavelength=192.png]

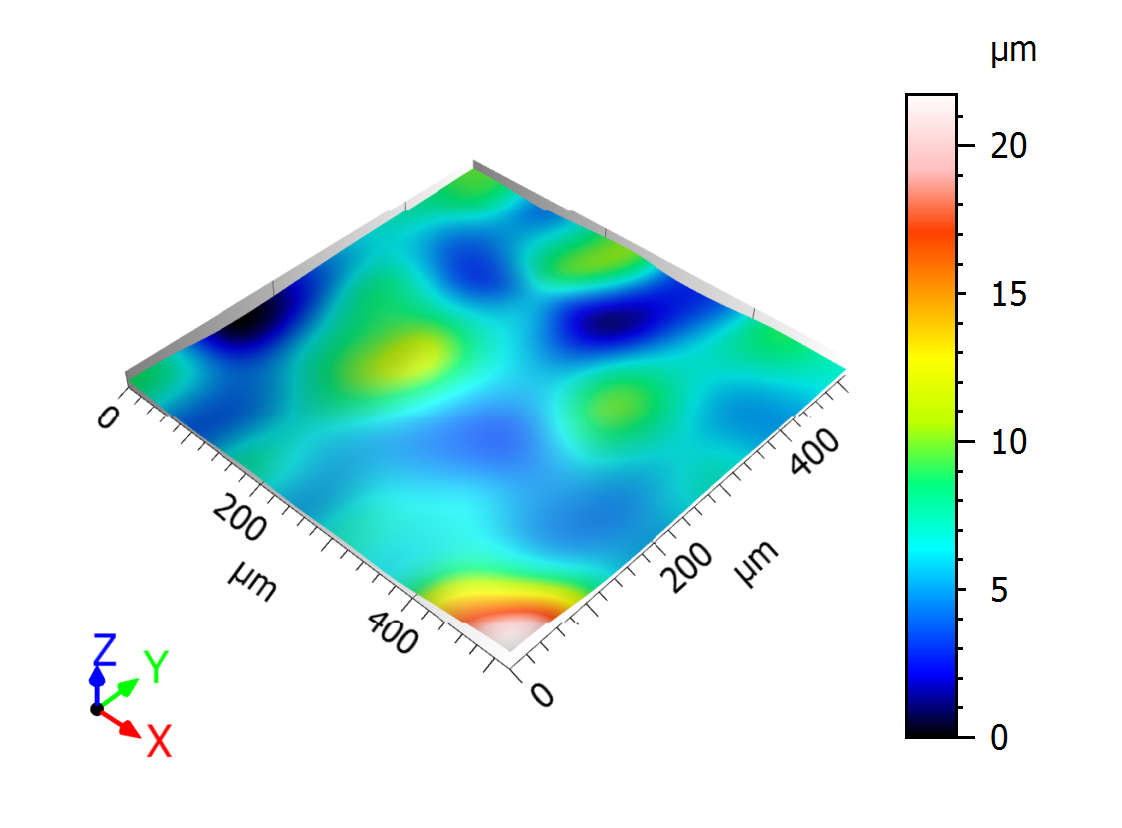

Supplement: Supplementary file 1 [file materials-13-03028-s001.zip › supplementary data/Bandpass filtering/3d_images_filtered_surfaces/L-PBFed_Number=17_CentralWavelength=288.png]

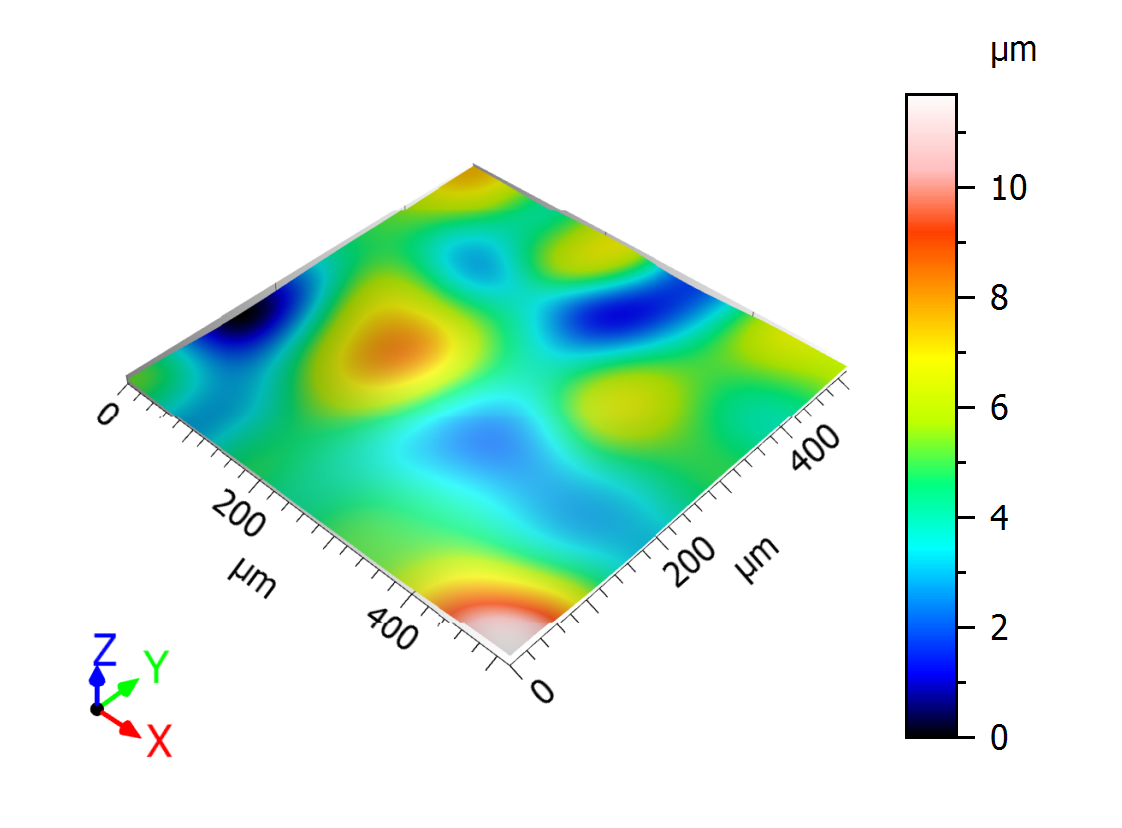

Supplement: Supplementary file 1 [file materials-13-03028-s001.zip › supplementary data/Bandpass filtering/3d_images_filtered_surfaces/L-PBFed_Number=18_CentralWavelength=384.png]

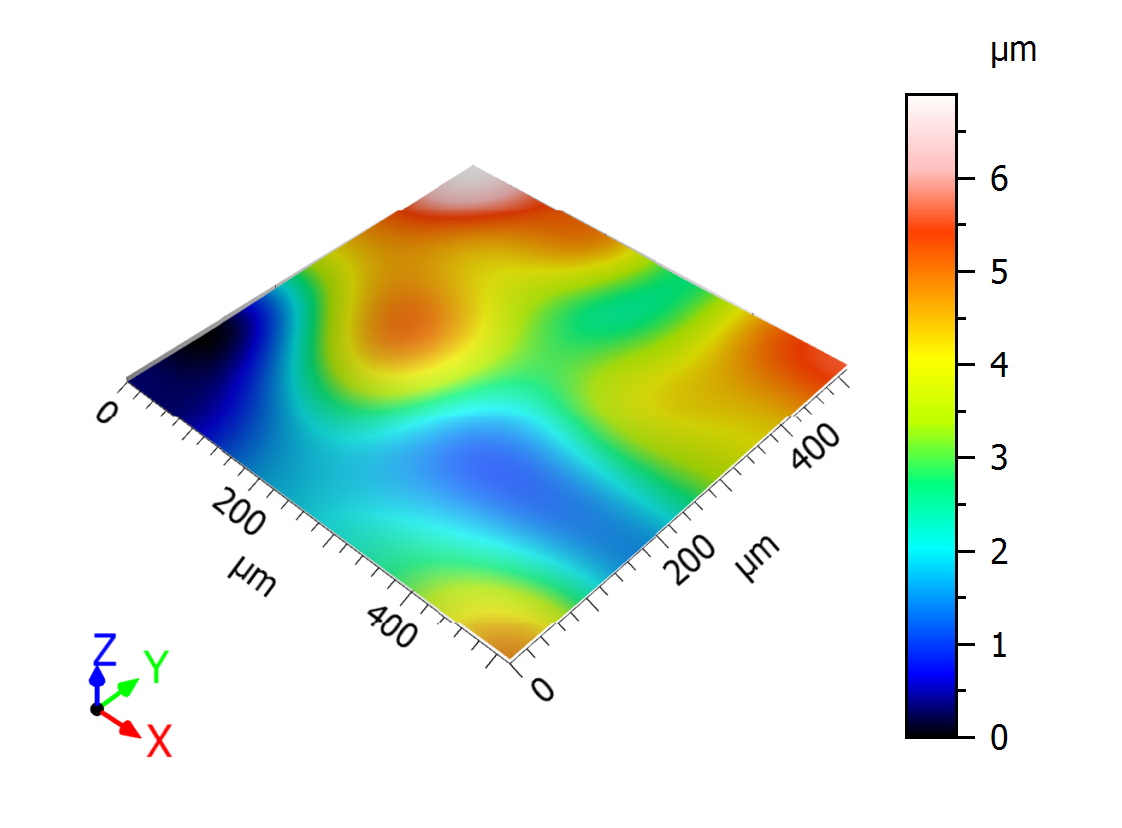

Supplement: Supplementary file 1 [file materials-13-03028-s001.zip › supplementary data/Bandpass filtering/3d_images_filtered_surfaces/L-PBFed_Number=19_CentralWavelength=576.png]

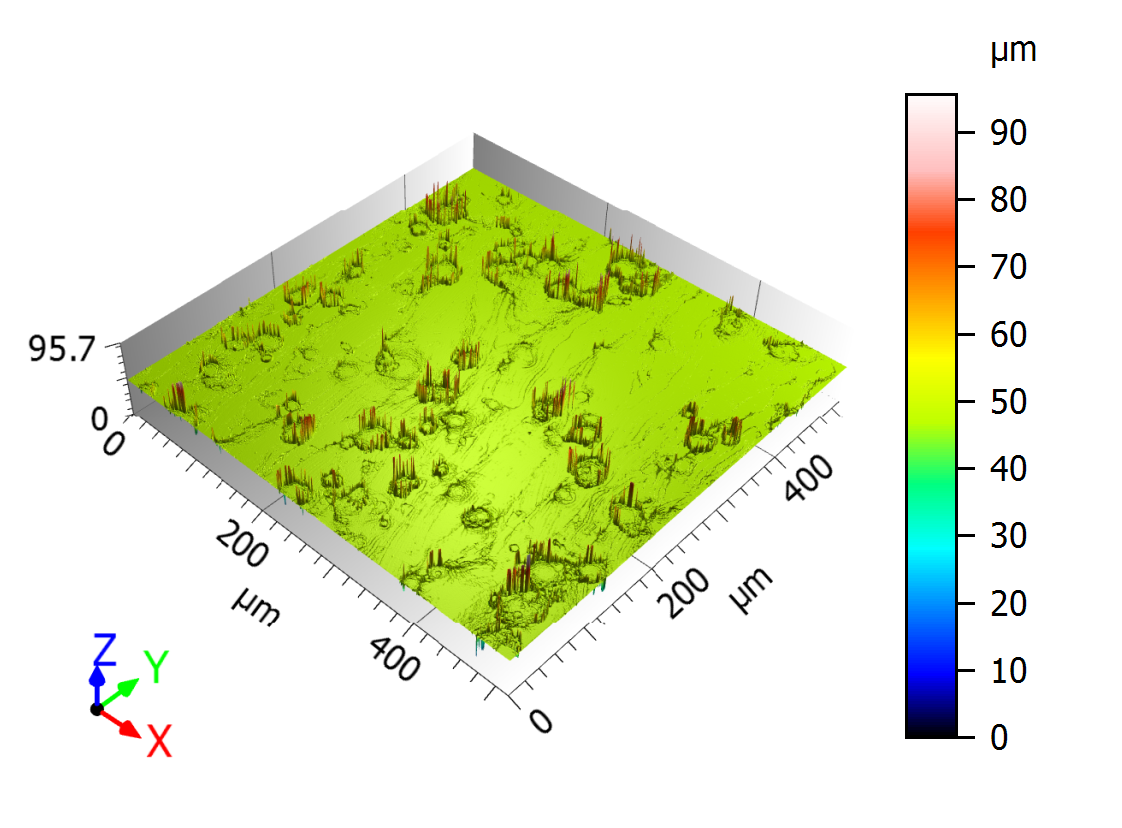

Supplement: Supplementary file 1 [file materials-13-03028-s001.zip › supplementary data/Bandpass filtering/3d_images_filtered_surfaces/L-PBFed_Number=1_CentralWavelength=1.125.png]

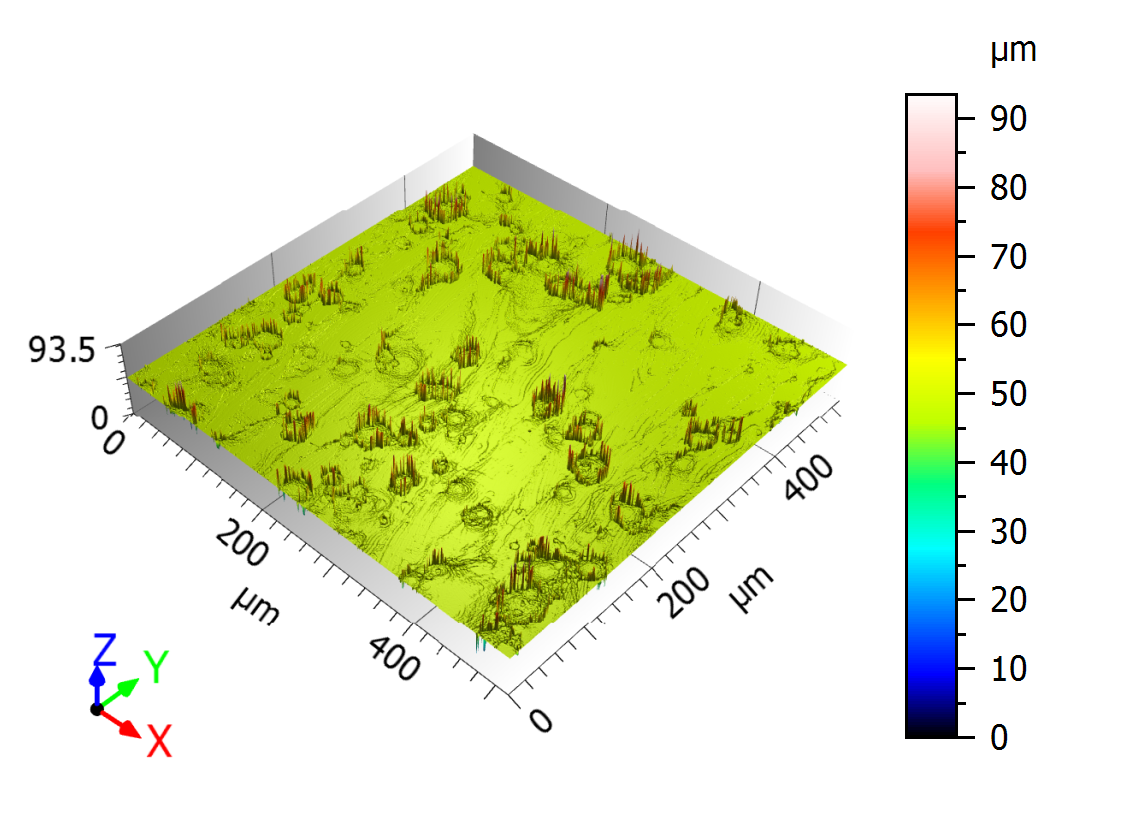

Supplement: Supplementary file 1 [file materials-13-03028-s001.zip › supplementary data/Bandpass filtering/3d_images_filtered_surfaces/L-PBFed_Number=2_CentralWavelength=1.5.png]

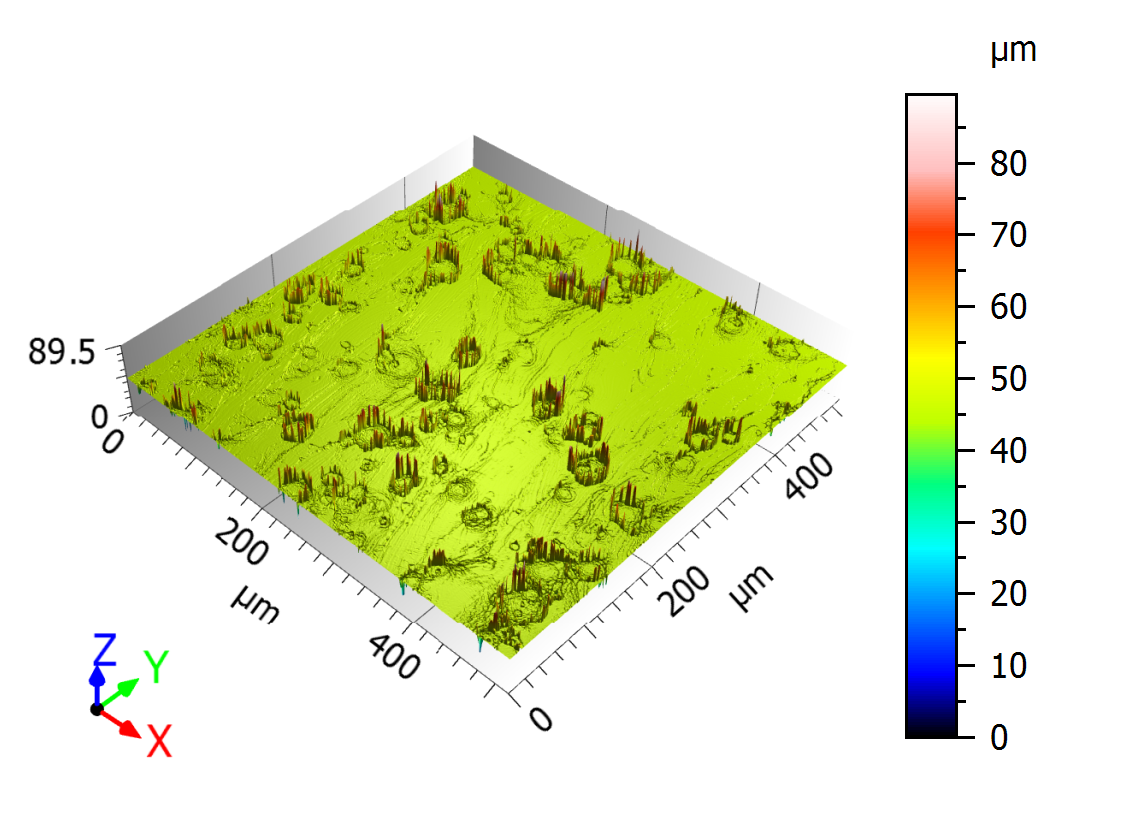

Supplement: Supplementary file 1 [file materials-13-03028-s001.zip › supplementary data/Bandpass filtering/3d_images_filtered_surfaces/L-PBFed_Number=3_CentralWavelength=2.25.png]

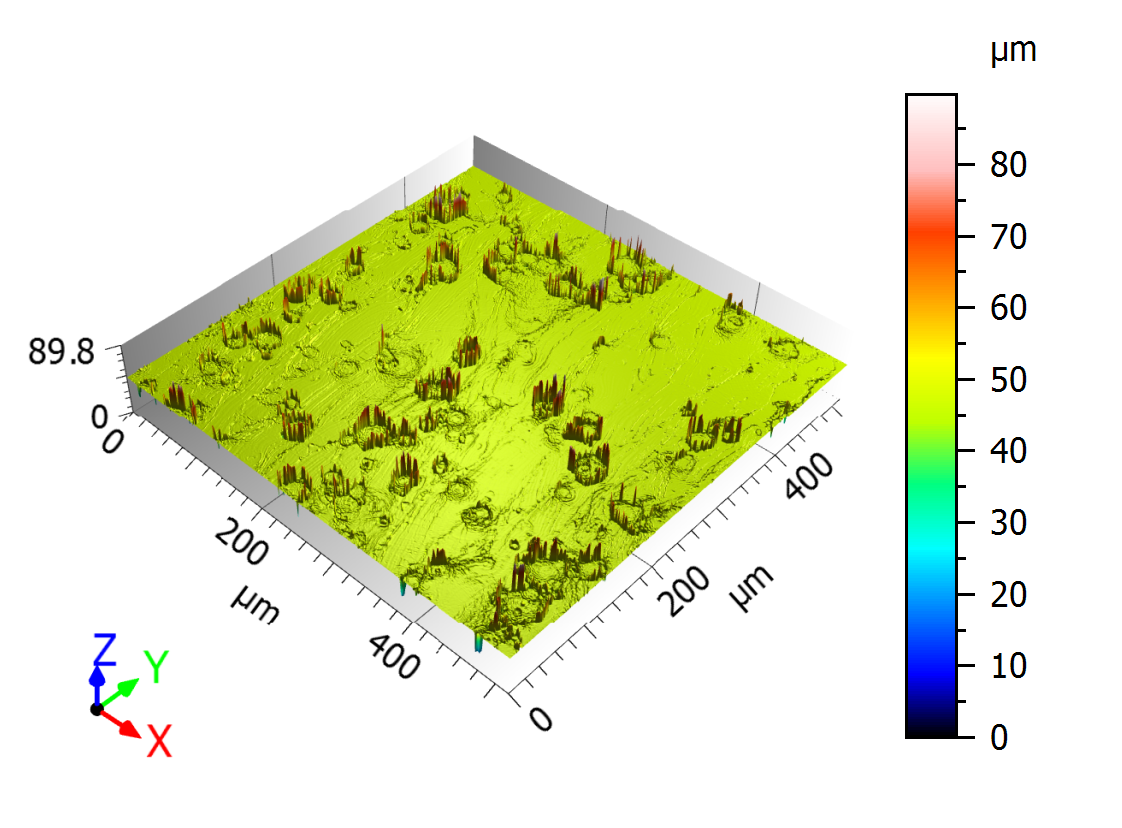

Supplement: Supplementary file 1 [file materials-13-03028-s001.zip › supplementary data/Bandpass filtering/3d_images_filtered_surfaces/L-PBFed_Number=4_CentralWavelength=3.png]

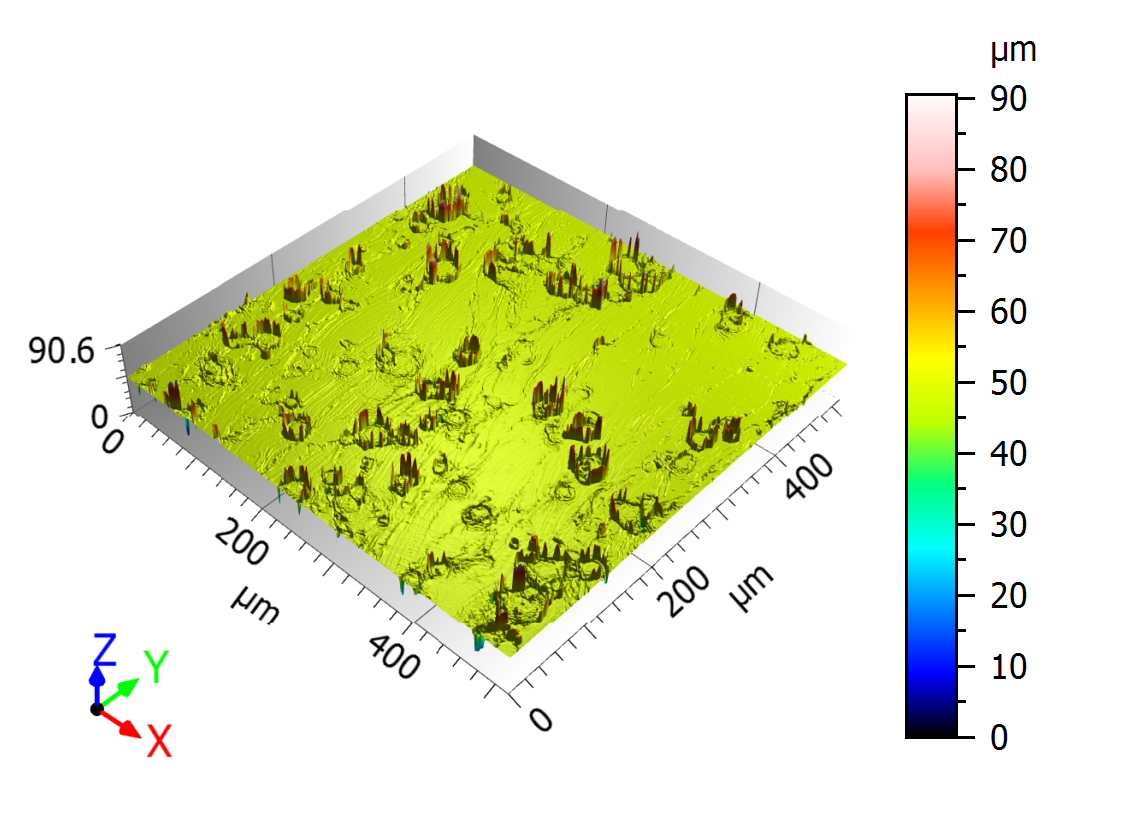

Supplement: Supplementary file 1 [file materials-13-03028-s001.zip › supplementary data/Bandpass filtering/3d_images_filtered_surfaces/L-PBFed_Number=5_CentralWavelength=4.5.png]

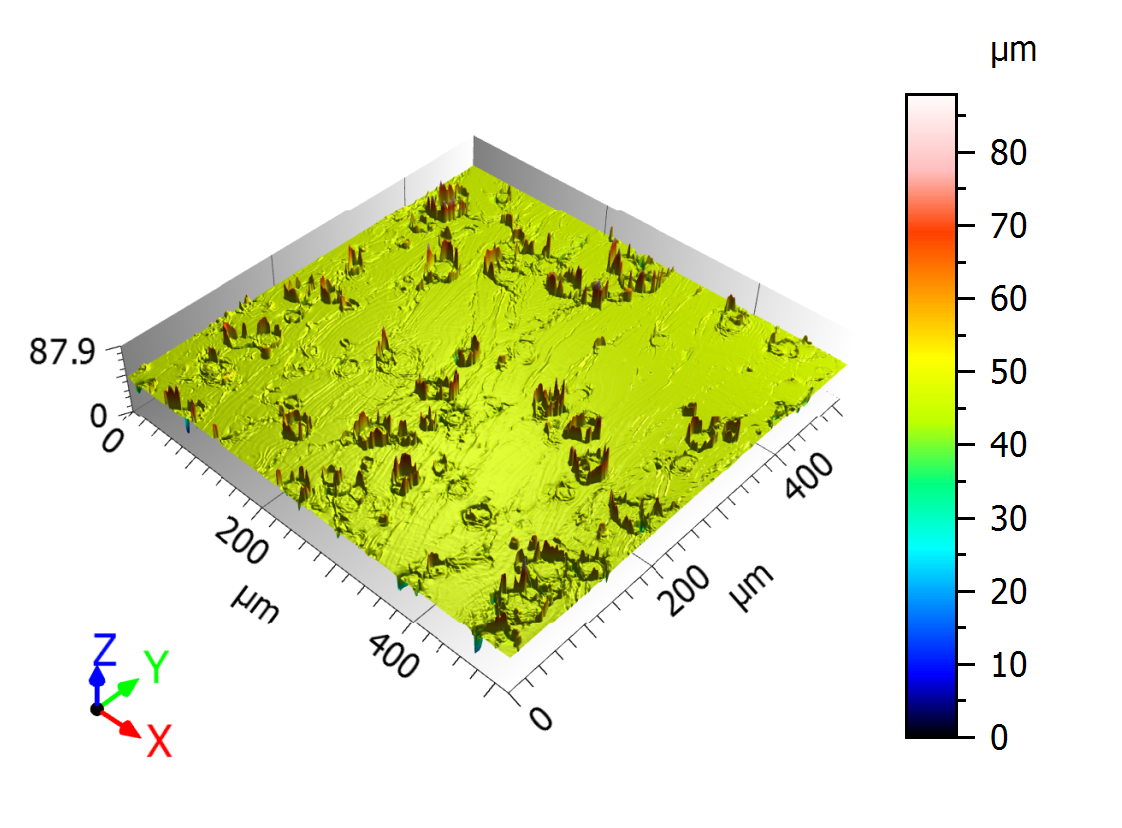

Supplement: Supplementary file 1 [file materials-13-03028-s001.zip › supplementary data/Bandpass filtering/3d_images_filtered_surfaces/L-PBFed_Number=6_CentralWavelength=6.png]

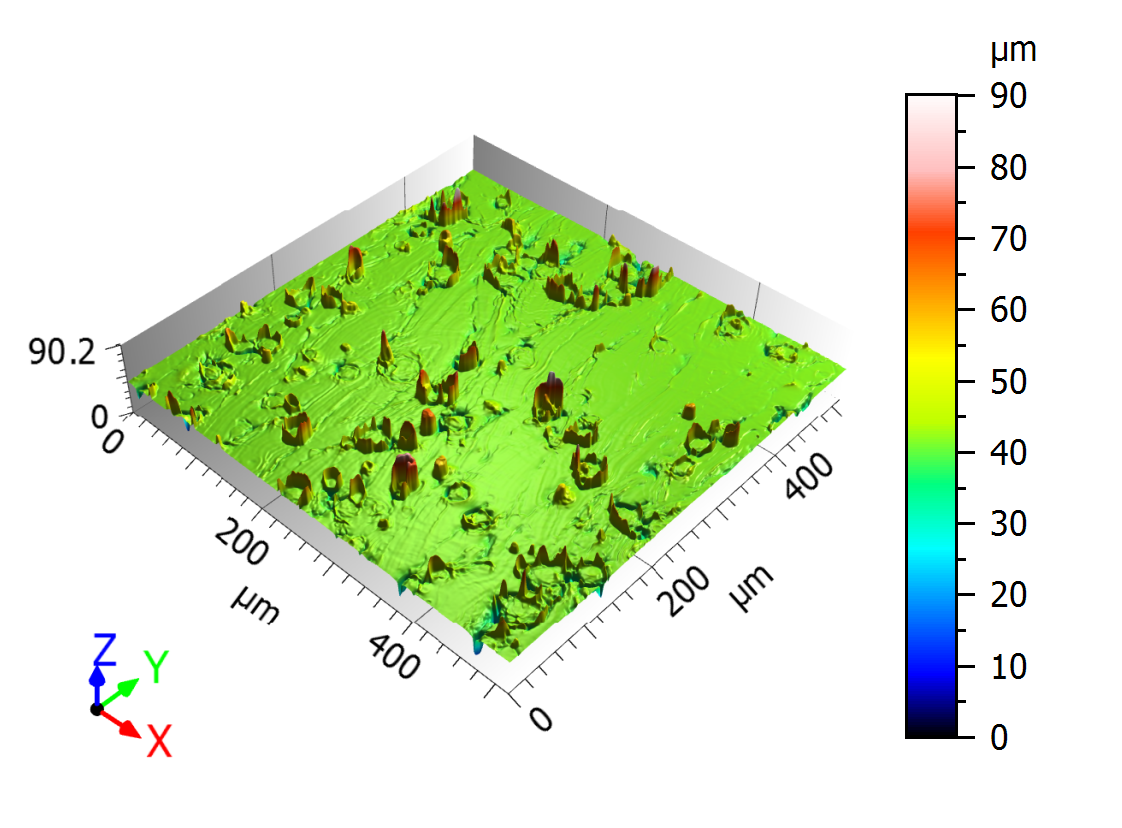

Supplement: Supplementary file 1 [file materials-13-03028-s001.zip › supplementary data/Bandpass filtering/3d_images_filtered_surfaces/L-PBFed_Number=7_CentralWavelength=9.png]

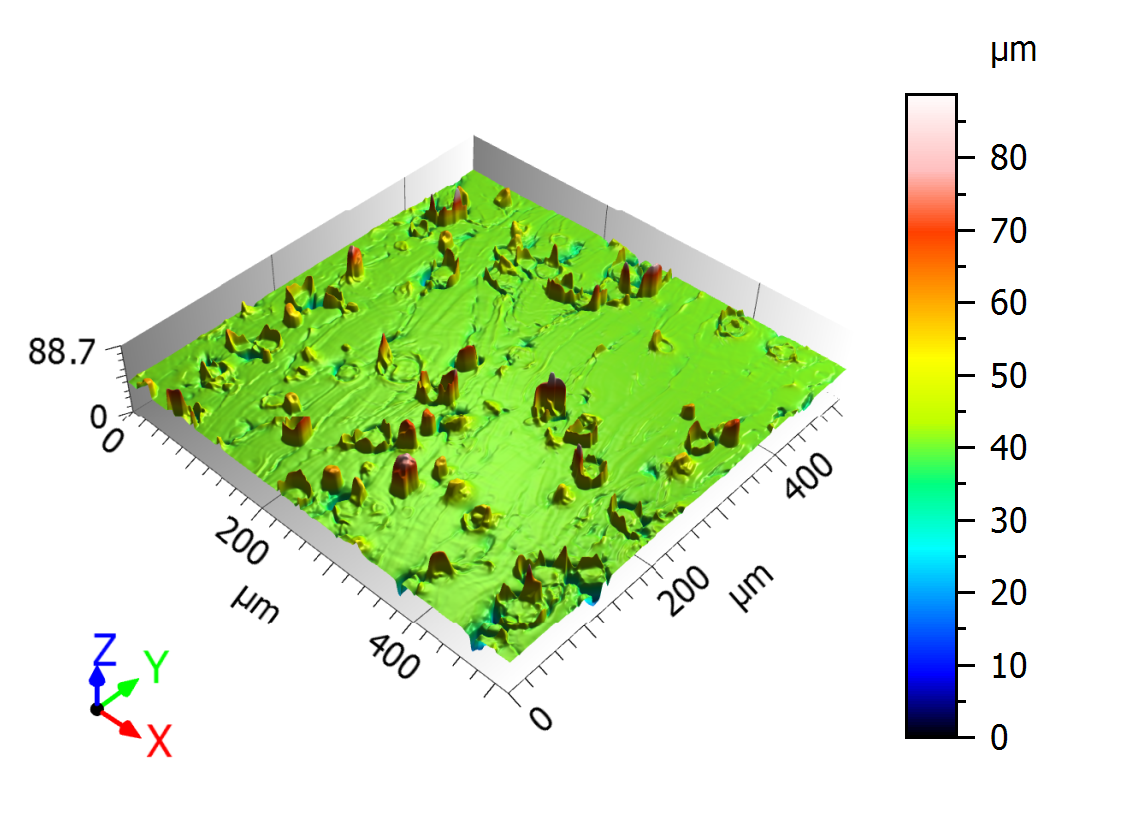

Supplement: Supplementary file 1 [file materials-13-03028-s001.zip › supplementary data/Bandpass filtering/3d_images_filtered_surfaces/L-PBFed_Number=8_CentralWavelength=12.png]

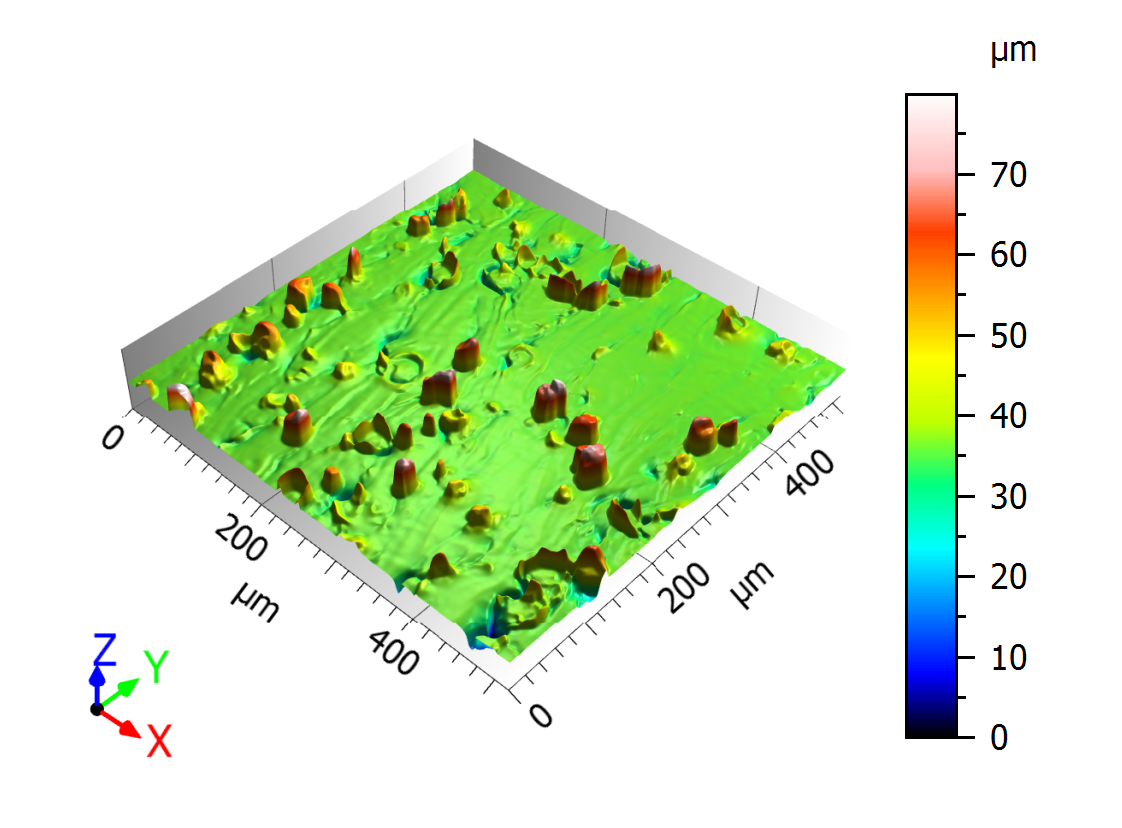

Supplement: Supplementary file 1 [file materials-13-03028-s001.zip › supplementary data/Bandpass filtering/3d_images_filtered_surfaces/L-PBFed_Number=9_CentralWavelength=18.png]

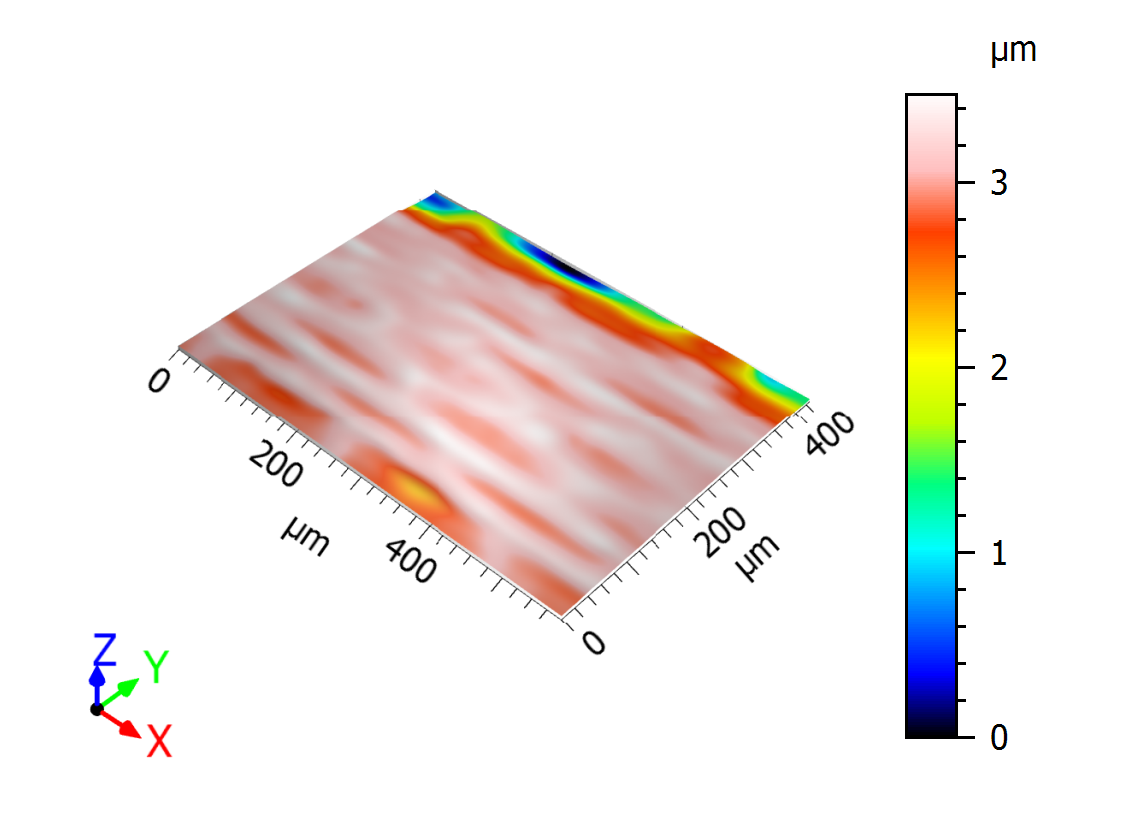

Supplement: Supplementary file 1 [file materials-13-03028-s001.zip › supplementary data/Bandpass filtering/3d_images_filtered_surfaces/MilledC_Number=10_CentralWavelength=72.png]

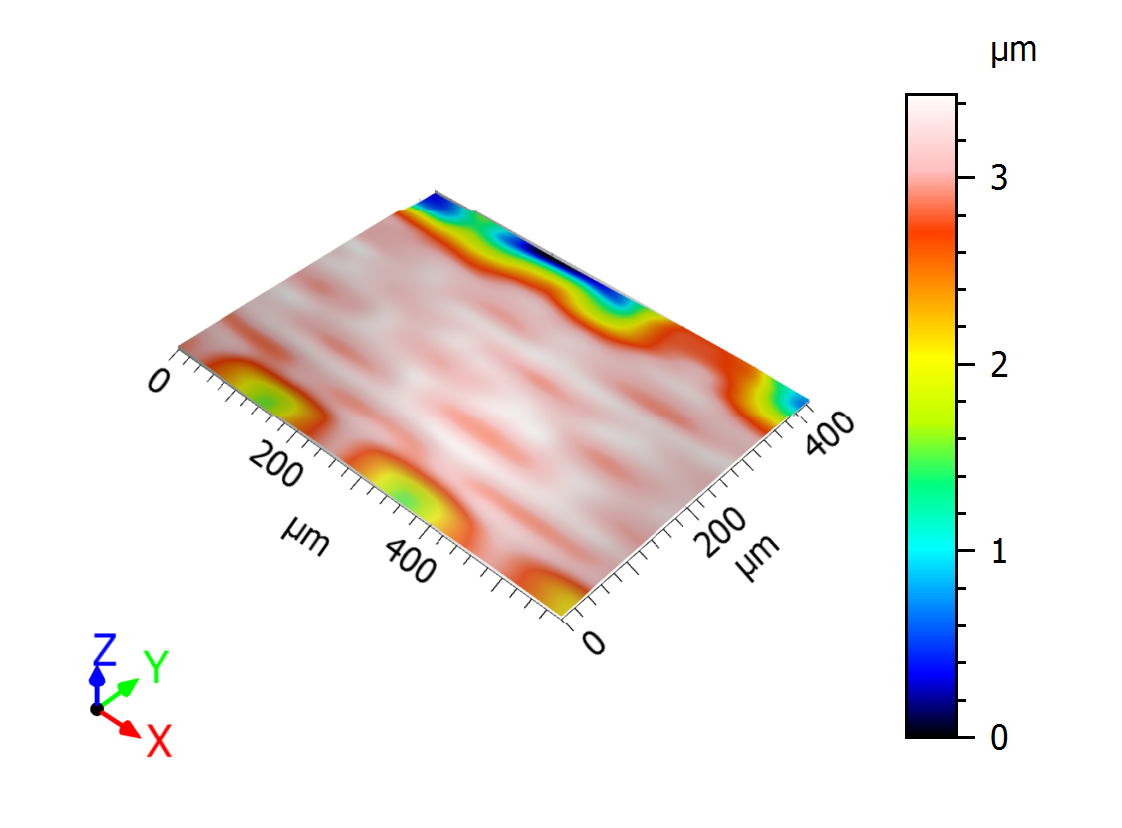

Supplement: Supplementary file 1 [file materials-13-03028-s001.zip › supplementary data/Bandpass filtering/3d_images_filtered_surfaces/MilledC_Number=11_CentralWavelength=96.png]

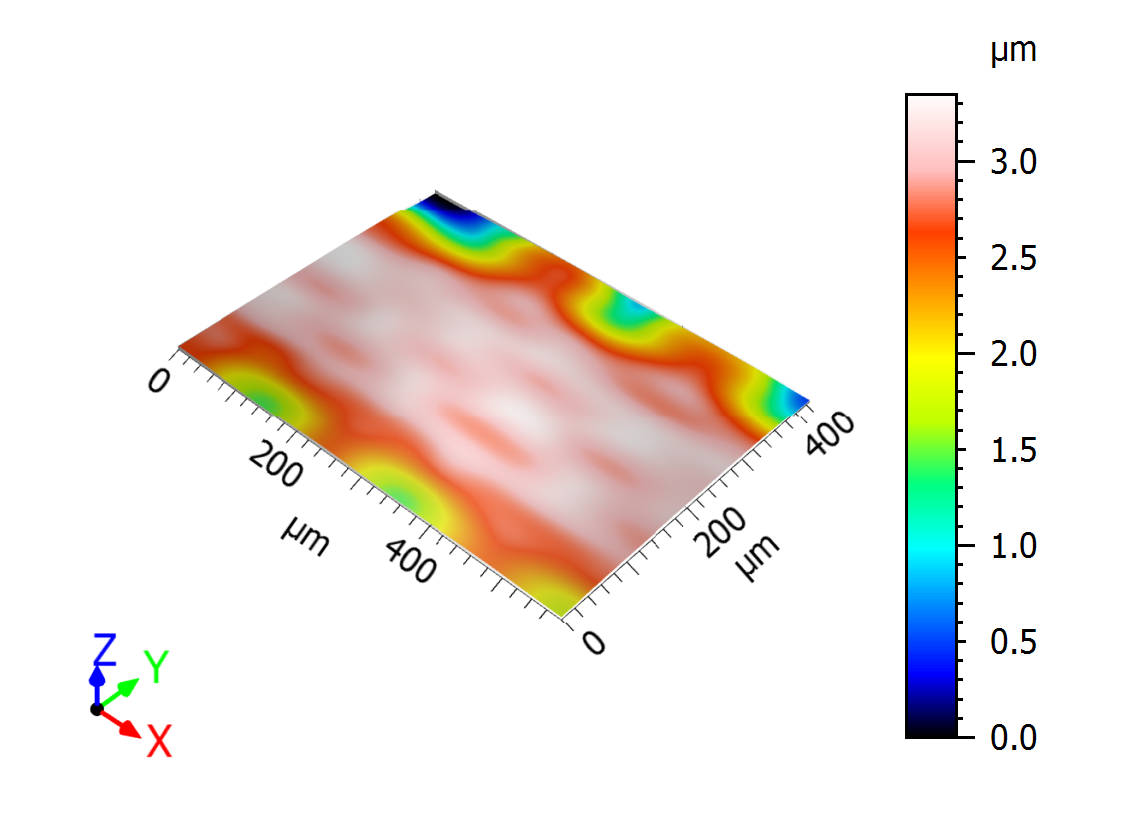

Supplement: Supplementary file 1 [file materials-13-03028-s001.zip › supplementary data/Bandpass filtering/3d_images_filtered_surfaces/MilledC_Number=12_CentralWavelength=144.png]

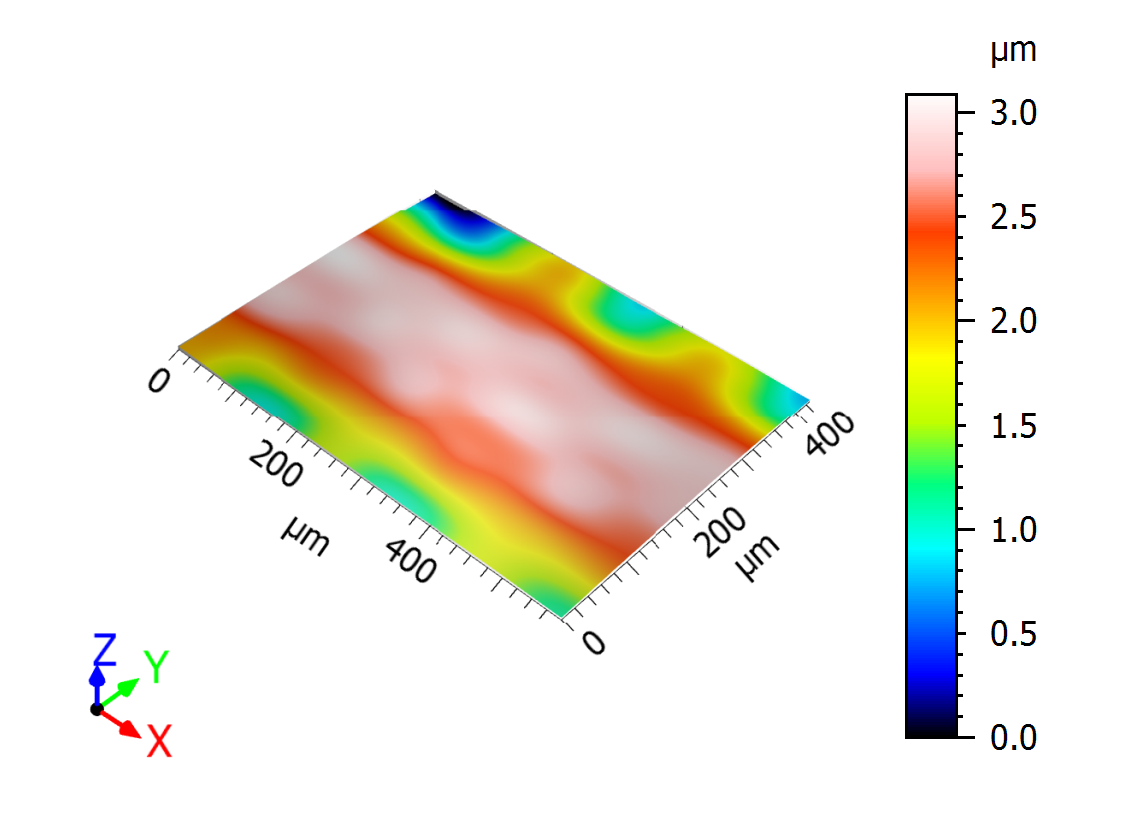

Supplement: Supplementary file 1 [file materials-13-03028-s001.zip › supplementary data/Bandpass filtering/3d_images_filtered_surfaces/MilledC_Number=13_CentralWavelength=192.png]

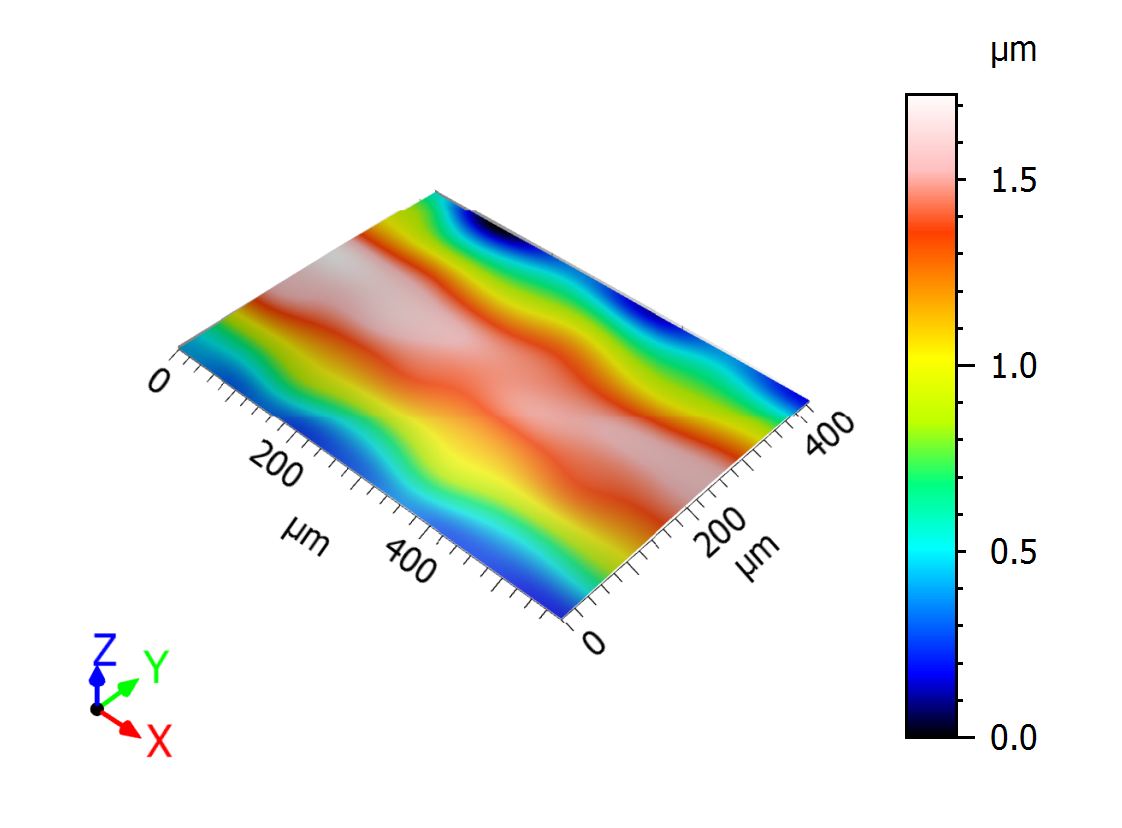

Supplement: Supplementary file 1 [file materials-13-03028-s001.zip › supplementary data/Bandpass filtering/3d_images_filtered_surfaces/MilledC_Number=14_CentralWavelength=270.png]

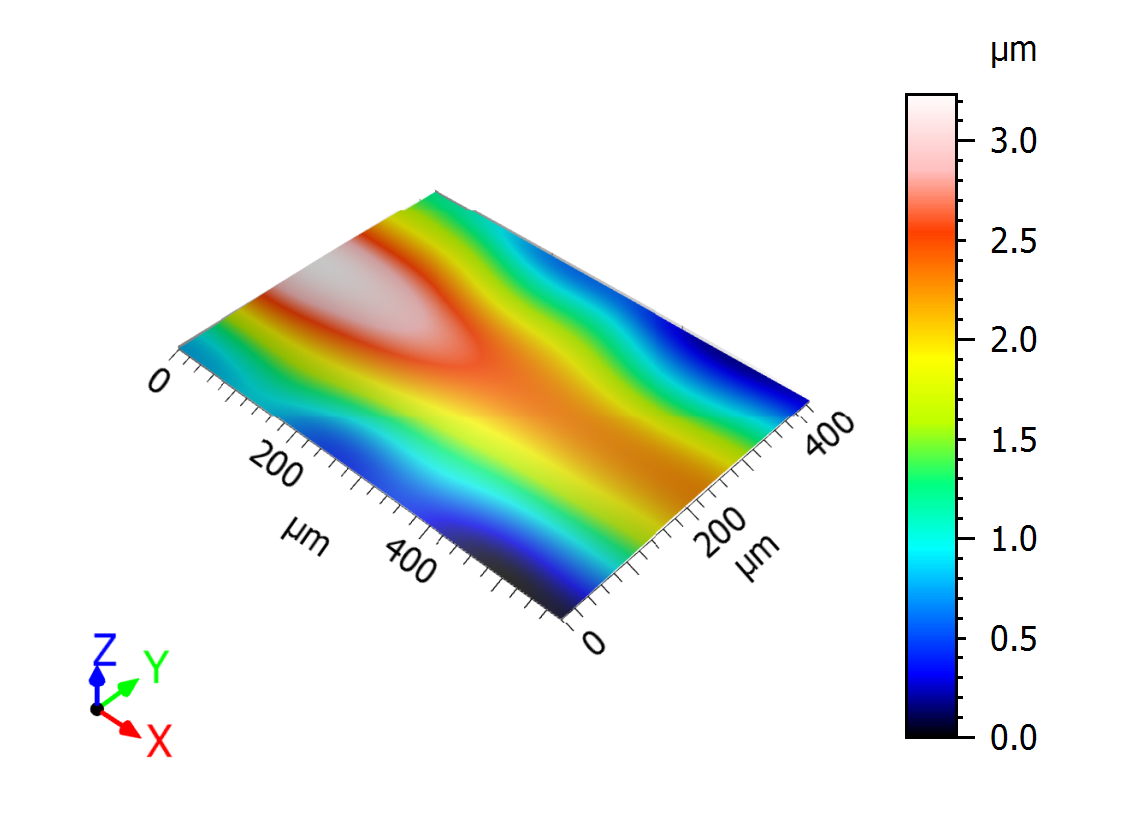

Supplement: Supplementary file 1 [file materials-13-03028-s001.zip › supplementary data/Bandpass filtering/3d_images_filtered_surfaces/MilledC_Number=15_CentralWavelength=384.png]

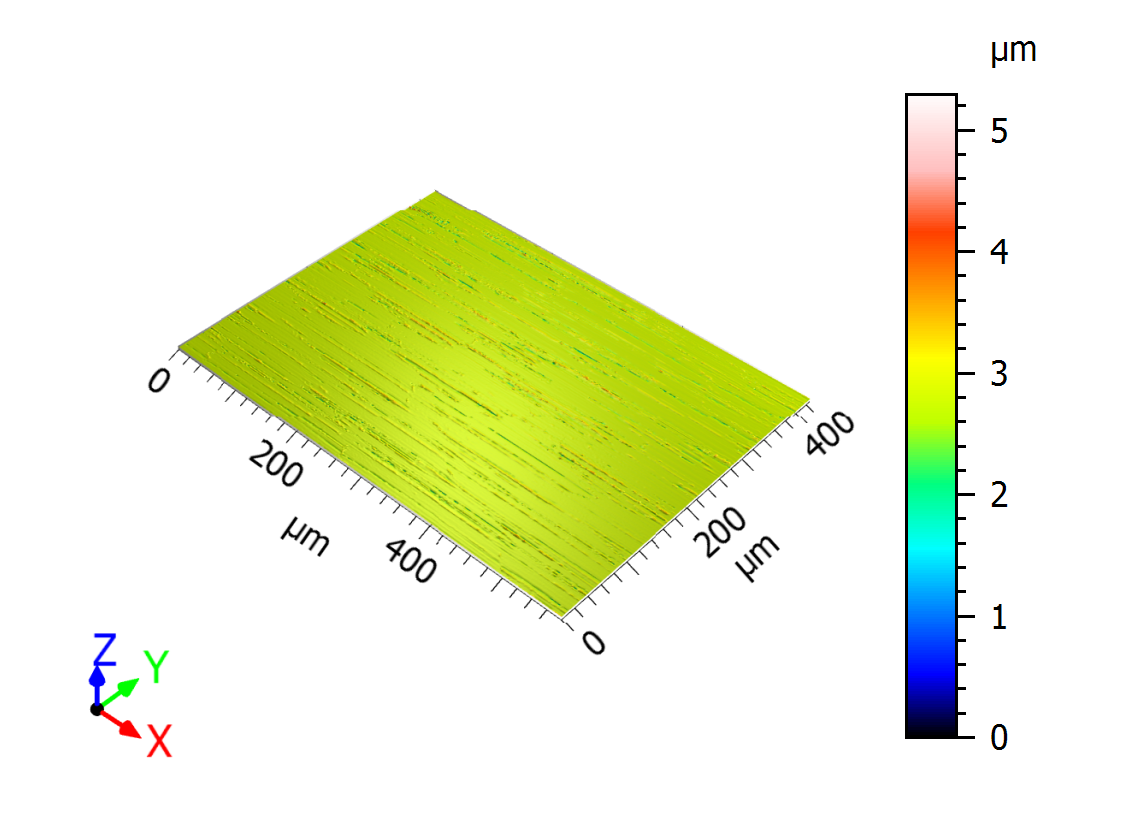

Supplement: Supplementary file 1 [file materials-13-03028-s001.zip › supplementary data/Bandpass filtering/3d_images_filtered_surfaces/MilledC_Number=1_CentralWavelength=3.png]

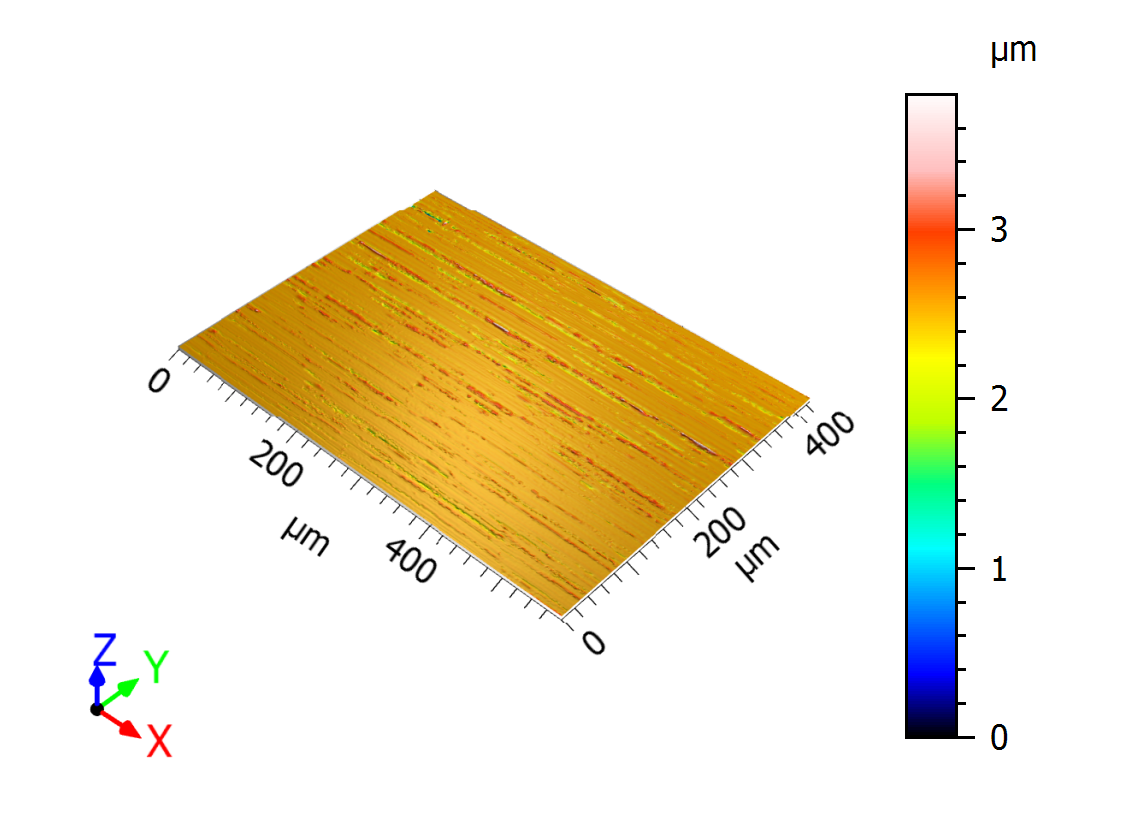

Supplement: Supplementary file 1 [file materials-13-03028-s001.zip › supplementary data/Bandpass filtering/3d_images_filtered_surfaces/MilledC_Number=2_CentralWavelength=4.5.png]

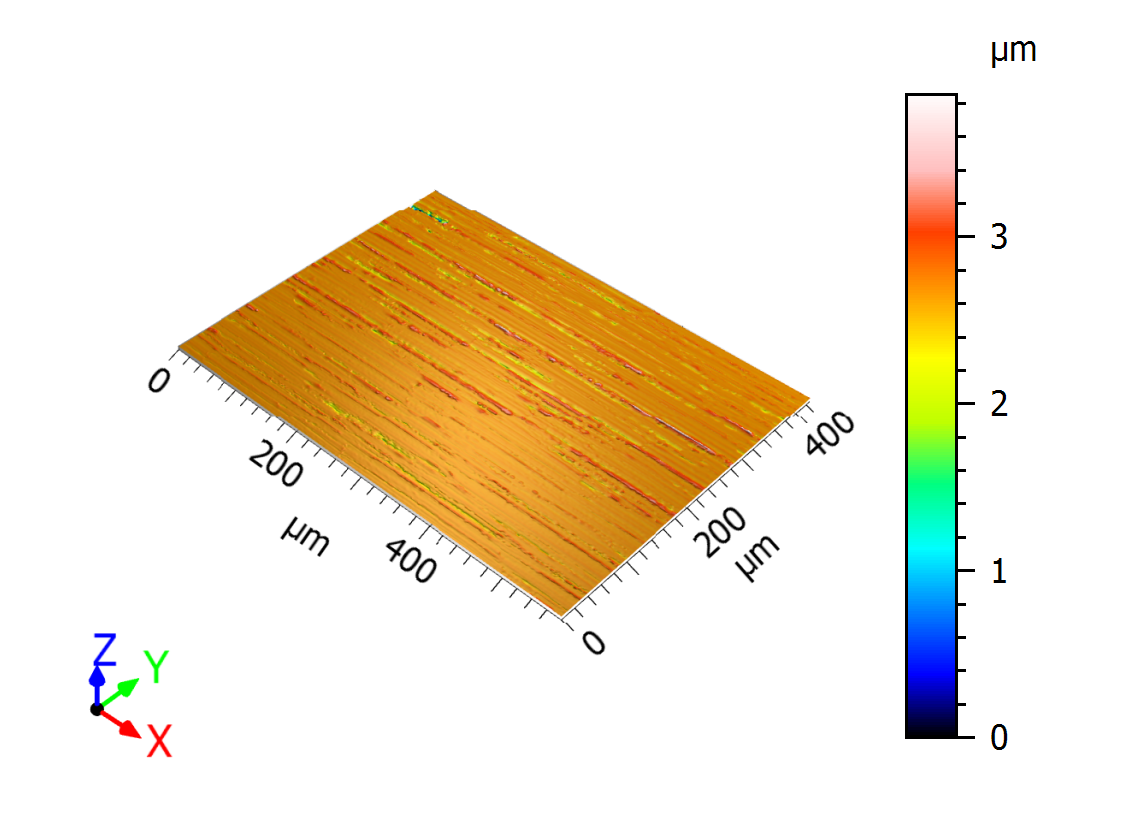

Supplement: Supplementary file 1 [file materials-13-03028-s001.zip › supplementary data/Bandpass filtering/3d_images_filtered_surfaces/MilledC_Number=3_CentralWavelength=6.png]

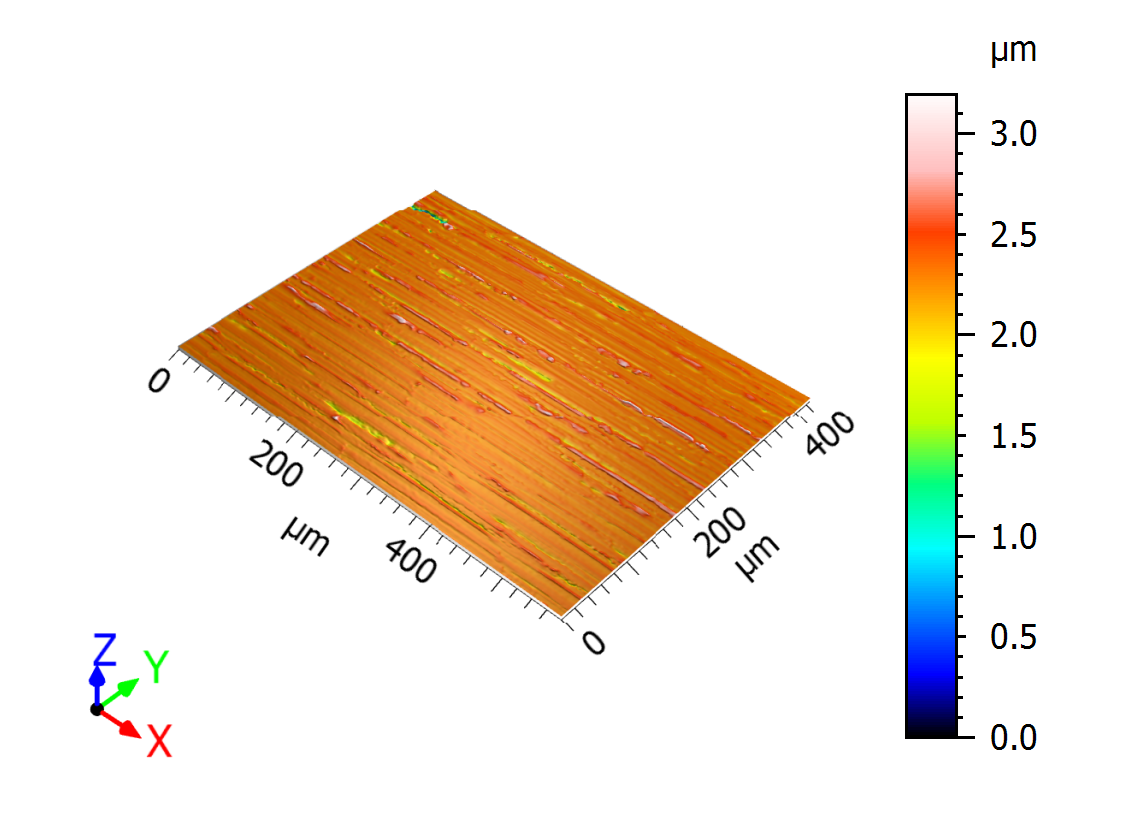

Supplement: Supplementary file 1 [file materials-13-03028-s001.zip › supplementary data/Bandpass filtering/3d_images_filtered_surfaces/MilledC_Number=4_CentralWavelength=9.png]

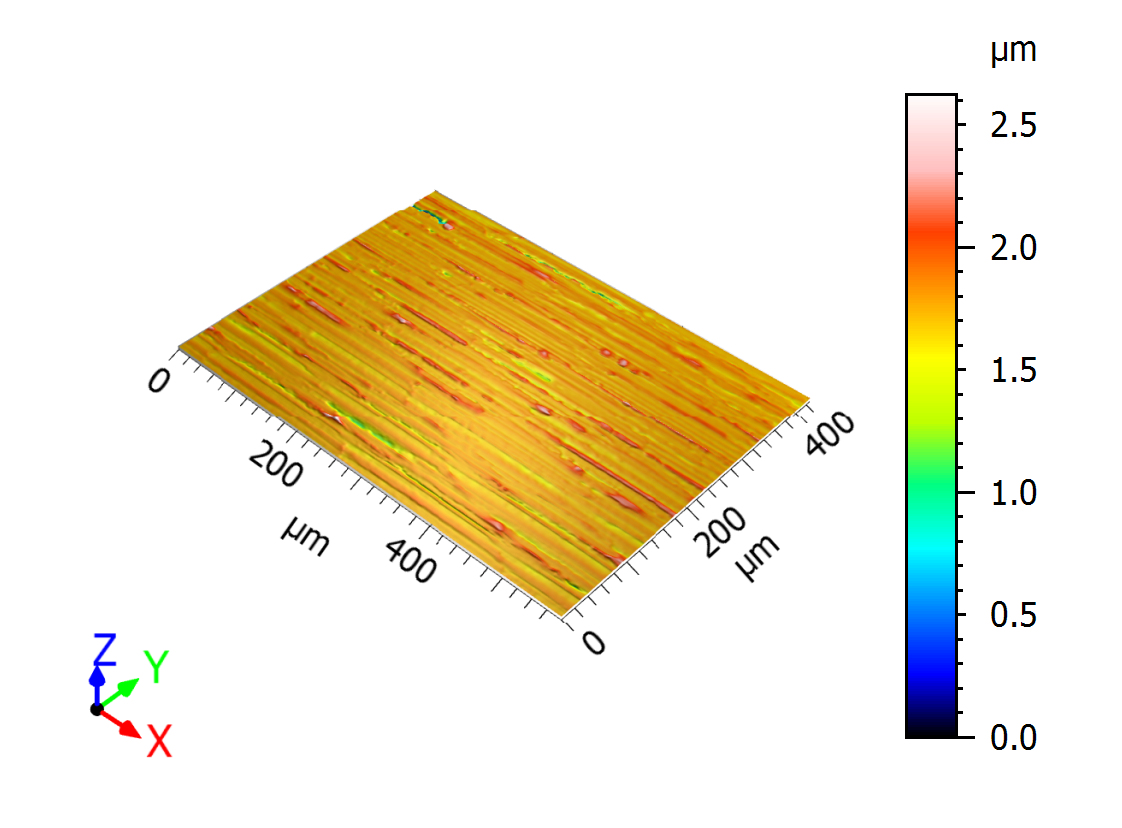

Supplement: Supplementary file 1 [file materials-13-03028-s001.zip › supplementary data/Bandpass filtering/3d_images_filtered_surfaces/MilledC_Number=5_CentralWavelength=12.png]

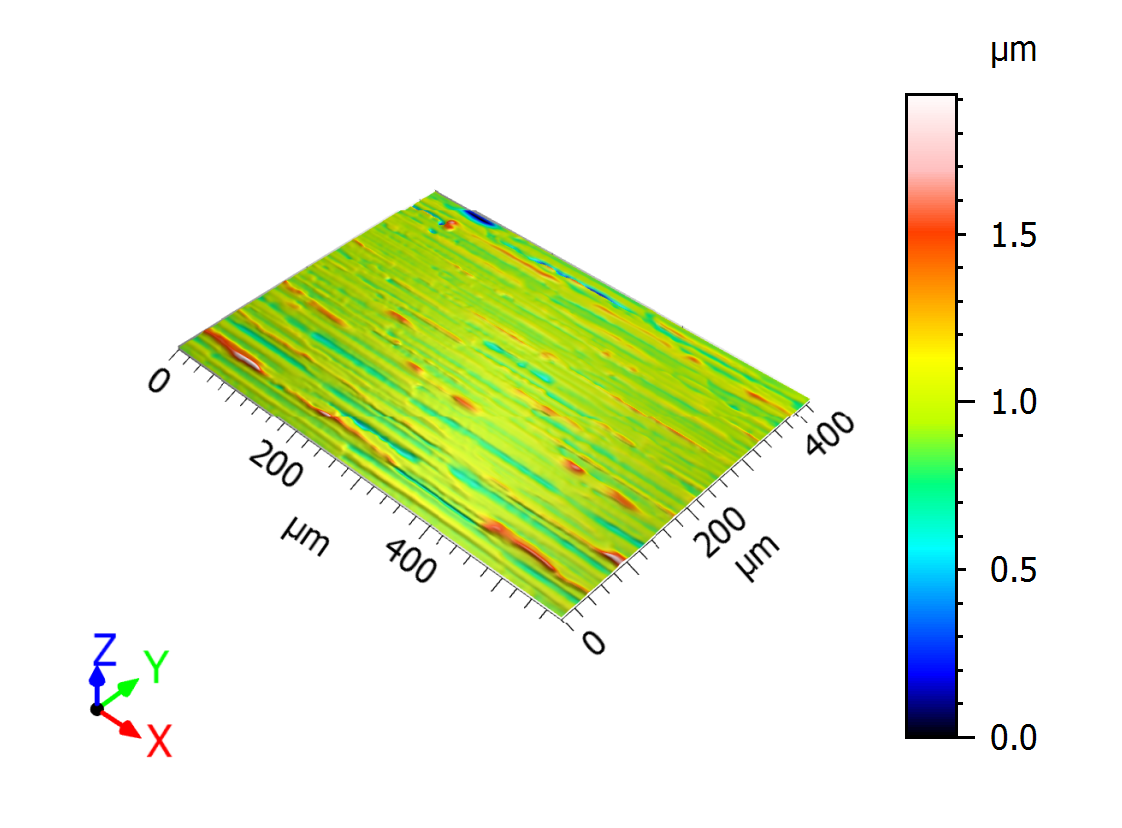

Supplement: Supplementary file 1 [file materials-13-03028-s001.zip › supplementary data/Bandpass filtering/3d_images_filtered_surfaces/MilledC_Number=6_CentralWavelength=18.png]

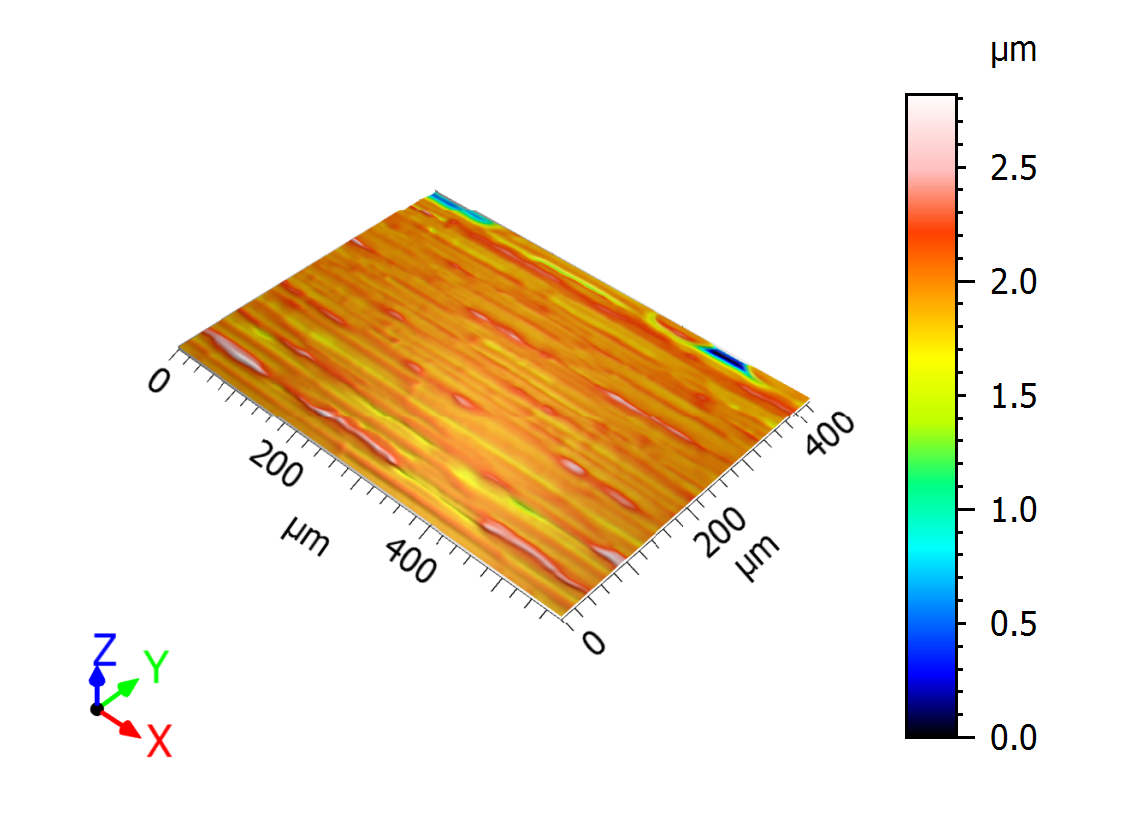

Supplement: Supplementary file 1 [file materials-13-03028-s001.zip › supplementary data/Bandpass filtering/3d_images_filtered_surfaces/MilledC_Number=7_CentralWavelength=24.png]

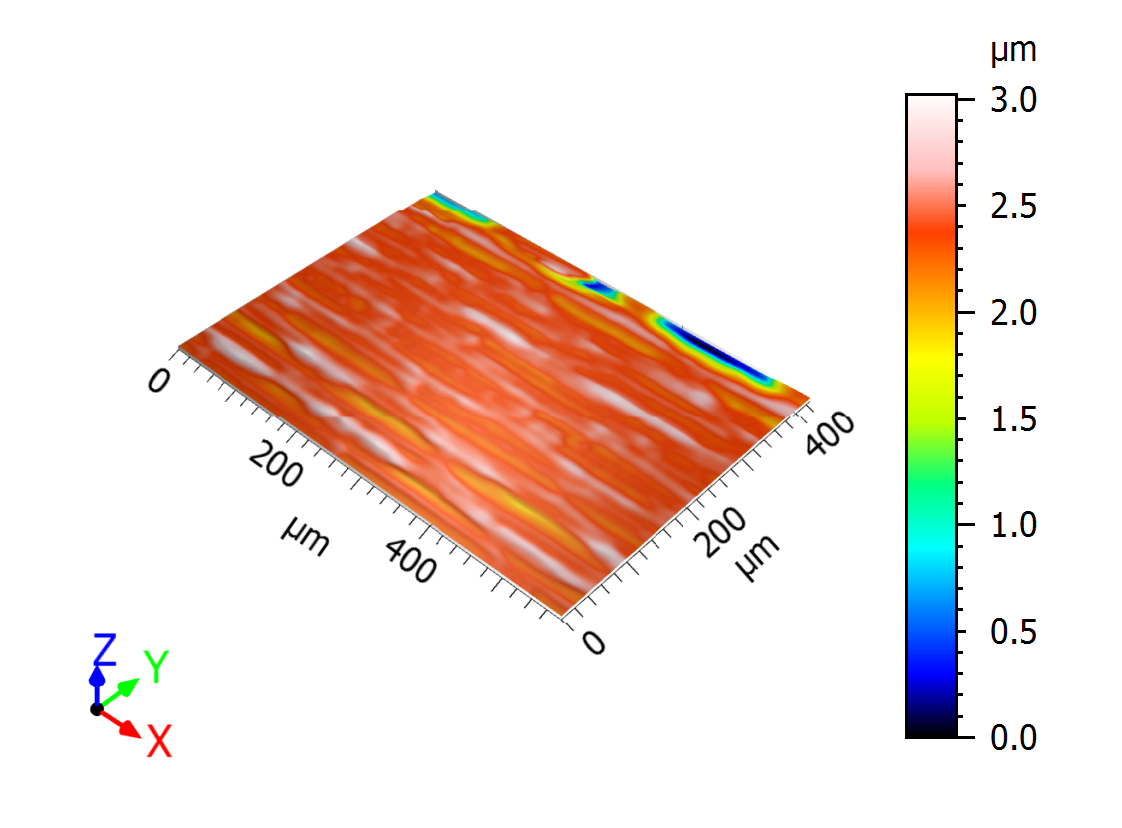

Supplement: Supplementary file 1 [file materials-13-03028-s001.zip › supplementary data/Bandpass filtering/3d_images_filtered_surfaces/MilledC_Number=8_CentralWavelength=36.png]

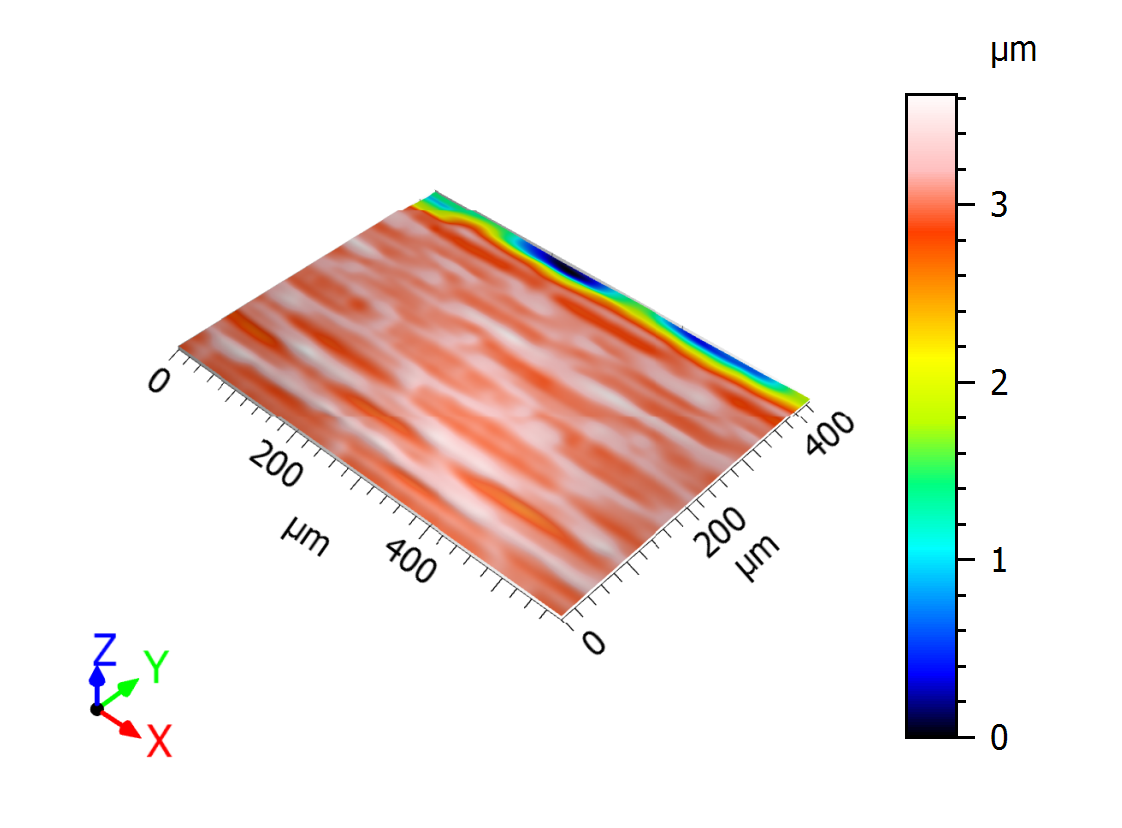

Supplement: Supplementary file 1 [file materials-13-03028-s001.zip › supplementary data/Bandpass filtering/3d_images_filtered_surfaces/MilledC_Number=9_CentralWavelength=48.png]

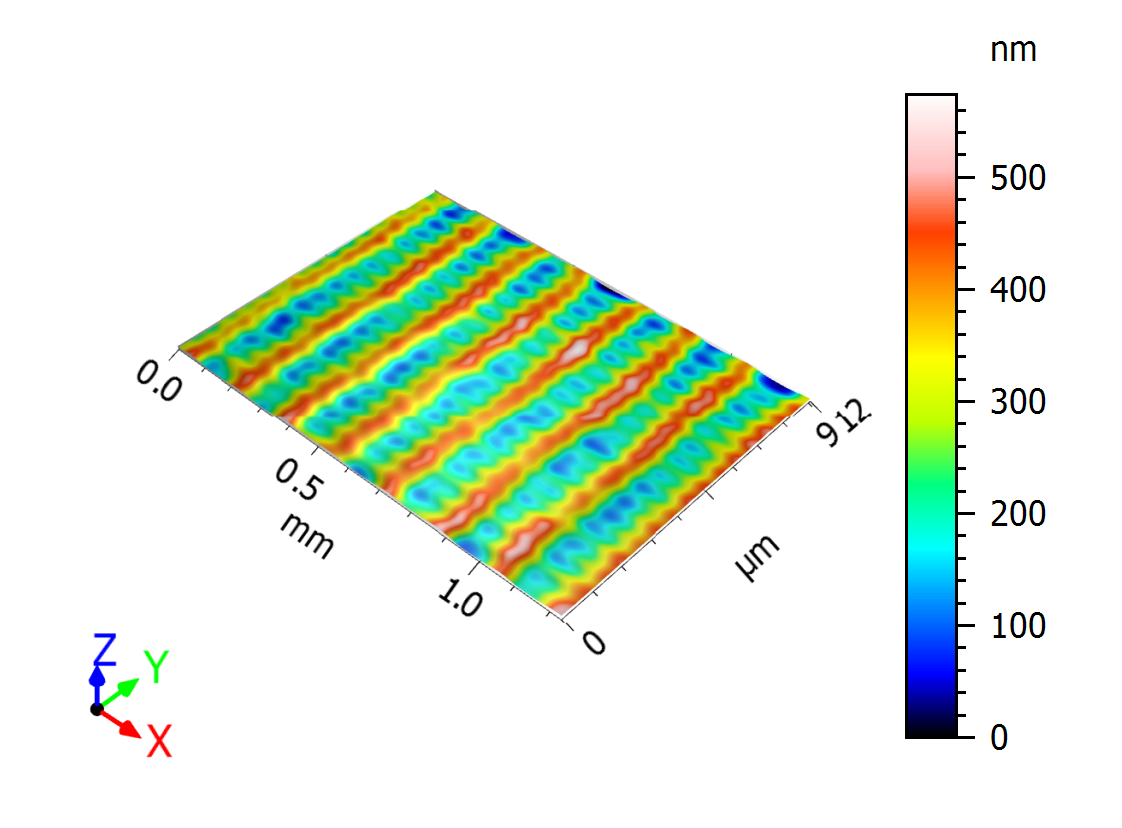

Supplement: Supplementary file 1 [file materials-13-03028-s001.zip › supplementary data/Bandpass filtering/3d_images_filtered_surfaces/MilledF_Number=10_CentralWavelength=144.png]

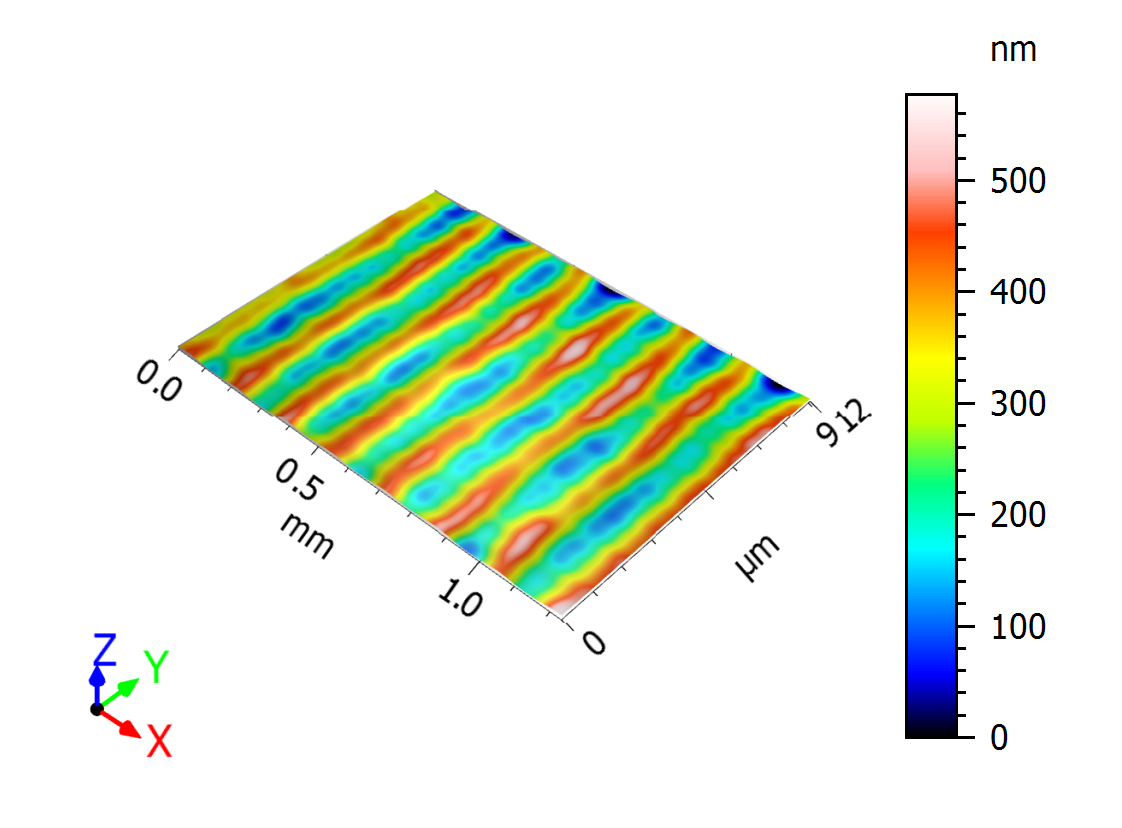

Supplement: Supplementary file 1 [file materials-13-03028-s001.zip › supplementary data/Bandpass filtering/3d_images_filtered_surfaces/MilledF_Number=11_CentralWavelength=192.png]
